# Palaeogenomics of Upper Palaeolithic to Neolithic European hunter-gatherers

**DOI:** 10.1038/s41586-023-05726-0

**Published:** 2023-03-01

**Authors:** Cosimo Posth, He Yu, Ayshin Ghalichi, Hélène Rougier, Isabelle Crevecoeur, Yilei Huang, Harald Ringbauer, Adam B. Rohrlach, Kathrin Nägele, Vanessa Villalba-Mouco, Rita Radzeviciute, Tiago Ferraz, Alexander Stoessel, Rezeda Tukhbatova, Dorothée G. Drucker, Martina Lari, Alessandra Modi, Stefania Vai, Tina Saupe, Christiana L. Scheib, Giulio Catalano, Luca Pagani, Sahra Talamo, Helen Fewlass, Laurent Klaric, André Morala, Mathieu Rué, Stéphane Madelaine, Laurent Crépin, Jean-Baptiste Caverne, Emmy Bocaege, Stefano Ricci, Francesco Boschin, Priscilla Bayle, Bruno Maureille, Foni Le Brun-Ricalens, Jean-Guillaume Bordes, Gregorio Oxilia, Eugenio Bortolini, Olivier Bignon-Lau, Grégory Debout, Michel Orliac, Antoine Zazzo, Vitale Sparacello, Elisabetta Starnini, Luca Sineo, Johannes van der Plicht, Laure Pecqueur, Gildas Merceron, Géraldine Garcia, Jean-Michel Leuvrey, Coralie Bay Garcia, Asier Gómez-Olivencia, Marta Połtowicz-Bobak, Dariusz Bobak, Mona Le Luyer, Paul Storm, Claudia Hoffmann, Jacek Kabaciński, Tatiana Filimonova, Svetlana Shnaider, Natalia Berezina, Borja González-Rabanal, Manuel R. González Morales, Ana B. Marín-Arroyo, Belén López, Carmen Alonso-Llamazares, Annamaria Ronchitelli, Caroline Polet, Ivan Jadin, Nicolas Cauwe, Joaquim Soler, Neus Coromina, Isaac Rufí, Richard Cottiaux, Geoffrey Clark, Lawrence G. Straus, Marie-Anne Julien, Silvia Renhart, Dorothea Talaa, Stefano Benazzi, Matteo Romandini, Luc Amkreutz, Hervé Bocherens, Christoph Wißing, Sébastien Villotte, Javier Fernández-López de Pablo, Magdalena Gómez-Puche, Marco Aurelio Esquembre-Bebia, Pierre Bodu, Liesbeth Smits, Bénédicte Souffi, Rimantas Jankauskas, Justina Kozakaitė, Christophe Cupillard, Hartmut Benthien, Kurt Wehrberger, Ralf W. Schmitz, Susanne C. Feine, Tim Schüler, Corinne Thevenet, Dan Grigorescu, Friedrich Lüth, Andreas Kotula, Henny Piezonka, Franz Schopper, Jiří Svoboda, Sandra Sázelová, Andrey Chizhevsky, Aleksandr Khokhlov, Nicholas J. Conard, Frédérique Valentin, Katerina Harvati, Patrick Semal, Bettina Jungklaus, Alexander Suvorov, Rick Schulting, Vyacheslav Moiseyev, Kristiina Mannermaa, Alexandra Buzhilova, Thomas Terberger, David Caramelli, Eveline Altena, Wolfgang Haak, Johannes Krause

**Affiliations:** 1 Archaeo- and Palaeogenetics, Institute for Archaeological Sciences, Department of Geosciences, University of Tübingen, Tübingen, Germany; 2 Senckenberg Centre for Human Evolution and Palaeoenvironment at the University of Tübingen, Tübingen, Germany; 3 Department of Archaeogenetics, Max Planck Institute for Evolutionary Anthropology, Leipzig, Germany; 4 State Key Laboratory of Protein and Plant Gene Research, School of Life Sciences, Peking University, Beijing, China; 5 Department of Anthropology, California State University Northridge, Northridge, CA USA; 6 Université de Bordeaux, CNRS, MC, PACEA UMR 5199, Pessac, France; 7 School of Mathematical Sciences, University of Adelaide, Adelaide, South Australia Australia; 8 Instituto Universitario de Investigación en Ciencias Ambientales de Aragón, IUCA-Aragosaurus, Zaragoza, Spain; 9 Department of Archaeogenetics, Max Planck Institute for the Science of Human History, Jena, Germany; 10 Institute of Zoology and Evolutionary Research, University of Jena, Jena, Germany; 11 Center of Excellence ‘Archaeometry’, Kazan Federal University, Kazan, Russia; 12 Department of Biology, University of Florence, Florence, Italy; 13 Estonian Biocentre, Institute of Genomics, University of Tartu, Tartu, Estonia; 14 St John’s College, University of Cambridge, Cambridge, UK; 15 Department of Biological, Chemical and Pharmaceutical Sciences and Technologies, University of Palermo, Palermo, Italy; 16 Department of Biology, University of Padova, Padova, Italy; 17 Department of Chemistry G. Ciamician, Alma Mater Studiorum, University of Bologna, Bologna, Italy; 18 Department of Human Evolution, Max Planck Institute for Evolutionary Anthropology, Leipzig, Germany; 19 UMR 8068 CNRS, TEMPS—Technologie et Ethnologie des Mondes Préhistoriques, Nanterre Cedex, France; 20 Musée National de Préhistoire, Les Eyzies de Tayac, France; 21 Paléotime, Villard-de-Lans, France; 22 UMR 5140 CNRS, Archéologie des Sociétés Méditerranéennes, Université Paul-Valéry, Montpellier, France; 23 UMR 7194, Histoire Naturelle de l’Homme Préhistorique (HNHP), Département Homme et Environnement, Muséum National d’Histoire Naturelle, CNRS, UPVD, Paris, France; 24 Association APRAGE (Approches pluridisciplinaires de recherche archéologique du Grand-Est), Besançon, France; 25 Inrap GE, Metz, France; 26 Skeletal Biology Research Centre, School of Anthropology and Conservation, University of Kent, Canterbury, UK; 27 Dipartimento di Scienze Fisiche, della Terra e dell’Ambiente, U.R. Preistoria e Antropologia, Università degli Studi di Siena, Siena, Italy; 28 Accademia dei Fisiocritici, Siena, Italy; 29 Centro Studi sul Quaternario ODV, Sansepolcro, Italy; 30 Institut National de Recherches Archéologiques, Bertrange, Luxembourg; 31 Department of Cultural Heritage, University of Bologna, Ravenna, Italy; 32 Human Ecology and Archaeology (HUMANE), Department of Archaeology and Anthropology, Institució Milà i Fontanals de Investigación en Humanidades, Consejo Superior de Investigaciones Científicas (IMF - CSIC), Barcelona, Spain; 33 UMR 7209—Archéozoologie et Archéobotanique-Sociétés, Pratiques et Environnements, Muséum National d’Histoire Naturelle, Paris, France; 34 Dipartimento di Scienze Della Vita e Dell’Ambiente, Sezione di Neuroscienze e Antropologia, Università Degli Studi di Cagliari, Cittadella Monserrato, Cagliari, Italy; 35 Dipartimento di Civiltà e Forme Del Sapere, Università di Pisa, Pisa, Italy; 36 Center for Isotope Research, Groningen University, Groningen, The Netherlands; 37 Inrap CIF, Croissy-Beaubourg, France; 38 UMR 7206 Éco-Anthropologie, Équipe ABBA. CNRS, MNHN, Université de Paris Cité, Musée de l’Homme, Paris, France; 39 PALEVOPRIM Lab UMR 7262 CNRS-INEE, University of Poitiers, Poitiers, France; 40 Centre de Valorisation des Collections Scientifiques, Université de Poitiers, Mignaloux Beauvoir, France; 41 Musées de Poitiers–Ville de Poitiers, Poitiers, France; 42 Departamento de Geología, Facultad de Ciencia y Tecnología, Universidad del País Vasco/Euskal Herriko Unibertsitatea (UPV/EHU), Leioa, Spain; 43 Sociedad de Ciencias Aranzadi, Donostia-San Sebastian, Spain; 44 Centro UCM-ISCIII de Investigación sobre Evolución y Comportamiento Humanos, Madrid, Spain; 45 Institute of Archaeology, University of Rzeszów, Rzeszów, Poland; 46 Foundation for Rzeszów Archaeological Centre, Rzeszów, Poland; 47 Center for Genomic Medicine, Massachusetts General Hospital, Boston, MA USA; 48 Department of Psychiatry, Harvard Medical School, Boston, MA USA; 49 Groninger Instituut voor Archeologie, Groningen University, Groningen, The Netherlands; 50 Stralsund Museum, Stralsund, Germany; 51 Institute of Archaeology and Ethnology, Polish Academy of Science, Poznań, Poland; 52 Institute of History, Archaeology and Ethnography, Dushanbe, Tajikistan; 53 ArchaeoZOOlogy in Siberia and Central Asia—ZooSCAn, CNRS–IAET SB RAS International Research Laboratory, IRL 2013, Institute of Archaeology SB RAS, Novosibirsk, Russia; 54 Research Institute and Museum of Anthropology, Moscow State University, Moscow, Russia; 55 Grupo de I+D+i EVOADAPTA (Evolución Humana y Adaptaciones durante la Prehistoria) Departamento de Ciencias Históricas, Universidad de Cantabria, Santander, Spain; 56 Instituto Internacional de Investigaciones Prehistóricas de Cantabria (IIIPC), Universidad de Cantabria-Gobierno de Cantabria-Banco Santander, Santander, Spain; 57 Departamento de Biología de Organismos y Sistemas, Universidad de Oviedo, Oviedo, Spain; 58 Quaternary Environments and Humans, OD Earth and History of Life, Royal Belgian Institute of Natural Sciences, Brussels, Belgium; 59 Musées Royaux d’Art et d’Histoire, Bruxelles, Belgium; 60 Institute of Historical Research, University of Girona, Catalonia, Spain; 61 INRAP/UMR 8215 Trajectoires 21, Paris, France; 62 School of Human Evolution and Social Change, Arizona State University, Tempe, AZ USA; 63 Department of Anthropology, University of New Mexico, Albuquerque, NM USA; 64 GéoArchPal-GéoArchÉon, Viéville sous-les-Cotes, France; 65 Archäologie & Münzkabinett, Universalmuseum Joanneum, Graz, Austria; 66 Museum ‘Das Dorf des Welan’, Wöllersdorf-Steinabrückl, Austria; 67 Pradis Cave Museum, Clauzetto, Italy; 68 Department of Humanities, University of Ferrara, Ferrara, Italy; 69 National Museum of Antiquities, Leiden, The Netherlands; 70 Faculty of Archaeology, Leiden University, Leiden, The Netherlands; 71 Biogeology, Department of Geosciences, University of Tübingen, Tübingen, Germany; 72 Unité de Recherches Art, Archéologie Patrimoine, Université de Liège, Liège, Belgium; 73 I.U. de Investigación en Arqueología y Patrimonio Histórico, University of Alicante, Sant Vicent del Raspeig, Alicante, Spain; 74 Arpa Patrimonio S. L., Alicante, Spain; 75 Amsterdam Centre of Ancient Studies and Archaeology, University of Amsterdam, Amsterdam, The Netherlands; 76 Department of Anatomy, Histology and Anthropology, Faculty of Medicine, Vilnius University, Vilnius, Lithuania; 77 Service Régional de l’Archéologie de Bourgogne-Franche-Comté, Besançon Cedex, France; 78 Laboratoire de Chrono-Environnement, UMR 6249 du CNRS, UFR des Sciences et Techniques, Besançon Cedex, France; 79 Weyhe, Germany; 80 Ulm, Germany; 81 LVR-LandesMuseum Bonn, Bonn, Germany; 82 Institute of Pre- and Protohistory, University of Tübingen, Tübingen, Germany; 83 Department of Archeological Sciences, Thuringian State Office for Monuments Preservation and Archeology, Weimar, Germany; 84 University of Bucharest, Faculty of Geology and Geophysics, Department of Geology, Bucharest, Romania; 85 Institute for Advanced Studies in Levant Culture and Civilization, Bucharest, Romania; 86 German Archaeological Institute, Berlin, Germany; 87 Brandenburg Authorities for Heritage Management and Archaeological State Museum, Zossen, Germany; 88 Institute for Pre- and Protohistory, Kiel University, Kiel, Germany; 89 Institute of Archeology at Brno, Czech Academy of Sciences, Centre for Palaeolithic and Paleoanthropology, Brno, Czechia; 90 Institute of Archaeology, Academy of Sciences of the Republic of Tatarstan, Kazan, Russia; 91 Samara State University of Social Sciences and Education, Samara, Russia; 92 Early Prehistory and Quaternary Ecology, Department of Geosciences, University of Tübingen, Tübingen, Germany; 93 Paleoanthropology, Institute for Archaeological Sciences, Department of Geosciences, University of Tübingen, Tübingen, Germany; 94 DFG Centre for Advanced Studies ‘Words, Bones, Genes, Tools’, University of Tübingen, Tübingen, Germany; 95 Royal Belgian Institute of Natural Sciences, Brussels, Belgium; 96 Anthropologie-Büro, Berlin, Germany; 97 Institute of Archaeology Russian, Academy of Sciences, Moscow, Russia; 98 School of Archaeology, University of Oxford, Oxford, UK; 99 Peter the Great Museum of Anthropology and Ethnography (Kunstkamera), Russian Academy of Sciences, Saint Petersburg, Russia; 100 Department of Cultures, University of Helsinki, Helsinki, Finland; 101 Seminar for Pre- and Protohistory, Göttingen University, Göttingen, Germany; 102 Lower Saxony State Service for Cultural Heritage, Hannover, Germany; 103 Department of Human Genetics, Leiden University Medical Center, Leiden, The Netherlands

**Keywords:** Archaeology, Population genetics, Biological anthropology, Evolutionary genetics

## Abstract

Modern humans have populated Europe for more than 45,000 years^1,2^. Our knowledge of the genetic relatedness and structure of ancient hunter-gatherers is however limited, owing to the scarceness and poor molecular preservation of human remains from that period^3^. Here we analyse 356 ancient hunter-gatherer genomes, including new genomic data for 116 individuals from 14 countries in western and central Eurasia, spanning between 35,000 and 5,000 years ago. We identify a genetic ancestry profile in individuals associated with Upper Palaeolithic Gravettian assemblages from western Europe that is distinct from contemporaneous groups related to this archaeological culture in central and southern Europe^4^, but resembles that of preceding individuals associated with the Aurignacian culture. This ancestry profile survived during the Last Glacial Maximum (25,000 to 19,000 years ago) in human populations from southwestern Europe associated with the Solutrean culture, and with the following Magdalenian culture that re-expanded northeastward after the Last Glacial Maximum. Conversely, we reveal a genetic turnover in southern Europe suggesting a local replacement of human groups around the time of the Last Glacial Maximum, accompanied by a north-to-south dispersal of populations associated with the Epigravettian culture. From at least 14,000 years ago, an ancestry related to this culture spread from the south across the rest of Europe, largely replacing the Magdalenian-associated gene pool. After a period of limited admixture that spanned the beginning of the Mesolithic, we find genetic interactions between western and eastern European hunter-gatherers, who were also characterized by marked differences in phenotypically relevant variants.

## Abstract (web summary)

Combined analysis of new genomic data from 116 ancient hunter-gatherer individuals together with previously published data provides insights into the genetic structure and demographic shifts of west Eurasian forager populations over a period of 30,000 years.

## Main

Modern humans left sub-Saharan Africa at least 60 thousand years ago (ka), and during their initial expansion into Eurasia, they genetically mixed with Neanderthals, resulting in 2–3% Neanderthal ancestry in the majority of present-day non-African populations^5^. Genomic data have shown that modern humans were present in western Eurasia^1,2^ at least 45 ka. Some of those early groups from more than 40 ka further admixed with Neanderthals, as shown by signals of recent introgression in individuals from Bacho Kiro in Bulgaria—associated with an Initial Upper Palaeolithic (IUP) archaeological culture—and from Peştera cu Oase in Romania^2,6^. Other individuals from that period, such as Zlatý kůň from Czechia and Ust’Ishim from Russia, do not carry significantly more Neanderthal ancestry than other non-African groups^1,7^, indicating differential interactions between Neanderthals and early modern humans during their initial expansions across Eurasia. Surprisingly, however, none of those pre-40 ka individuals left substantial traces in the genetic makeup of present-day Eurasian populations^1,2,6,7^. The oldest genomes carrying ancestries that derive primarily from the lineage leading to present-day Europeans are Kostenki 14 (from 37 ka, with uncertain archaeological association from western Russia), Goyet Q116-1 (35 ka, Aurignacian-associated from Belgium) and Bacho Kiro 1653 (35 ka, probably Aurignacian-associated from Bulgaria)^2,4,8^. These data suggest that the genetic ancestries identified in the pre-40 ka individuals analysed so far went largely extinct or were assimilated by subsequent expansions^1,9^. The Kostenki genetic signature (related to the Kostenki 14 genome, and hereafter referred to as the Kostenki cluster or ancestry) contributed to the later Věstonice genetic cluster (hereafter, Věstonice cluster or ancestry), named after the Dolní Věstonice site in Czechia^4^. This genetic signature is shared among individuals associated with the archaeologically defined Gravettian culture (33–26 ka) in central and southern Europe and seemingly disappeared after the Last Glacial Maximum^4^ (LGM). However, the genetic profile of contemporaneous Gravettian-associated individuals from western Europe remains unknown, as is their contribution to populations after the LGM. Known to have been the coldest phase of the last Ice Age, the LGM is considered to have caused a demographic decline in large parts of Europe^10^, with populations retracting to southern latitudes as attested—for example—by the contemporaneity of the Solutrean culture (24–19 ka) in the Iberian peninsula and southern France. Other proposed climatic refugia for human survival during this period are the Italian peninsula, the Balkans and the southeastern European Plain, but the actual genetic contribution of populations from these regions to post-LGM Europeans is highly debated^11–13^.

After the LGM, a genetic component distantly linked to the Goyet Q116-1 individual from Belgium dated to 35 ka—named GoyetQ2 ancestry (hereafter, GoyetQ2 cluster or ancestry)—reappeared in individuals from southwestern and central Europe associated with the Magdalenian culture (19–14 ka from Iberia to eastern Europe across central Europe) and in an admixed form in subsequent Final Palaeolithic and Mesolithic hunter-gatherers^4,14^, but the geographic extension of this ancestry is still unclear. Instead, in southern Europe, a distinct hunter-gatherer genetic profile was found as early as 17 ka in individuals associated with the Epigravettian culture^15^ (24–12 ka, from the Italian peninsula to the southeastern European Plain across the Balkans). This ‘Villabruna’ ancestry (hereafter, Villabruna cluster or ancestry) showed connections to ancient and present-day Near Eastern populations^4,16^, but the mode and tempo of its expansion into the Italian peninsula remain unexplored. The Villabruna ancestry later appeared in central Europe and it is thought to have largely replaced groups related to the GoyetQ2 ancestry^4^. However, its formation, diffusion and interaction with contemporaneous hunter-gatherers from eastern Europe and their interplay with later expansions of Neolithic farmers from southeastern Europe are not well characterized.

In this study, we analyse 356 ancient hunter-gatherer genomes including new genomic data of 116 individuals dated to 35–5 ka alongside a novel contamination-estimation method based on runs of homozygosity. We provide a systematic description of the genomic transformations that hunter-gatherer groups experienced from the early Upper Palaeolithic onwards across western and central Eurasia and how those are possibly linked to cultural and climatic changes.

## Ancient DNA data generation

**Fig. 1:**
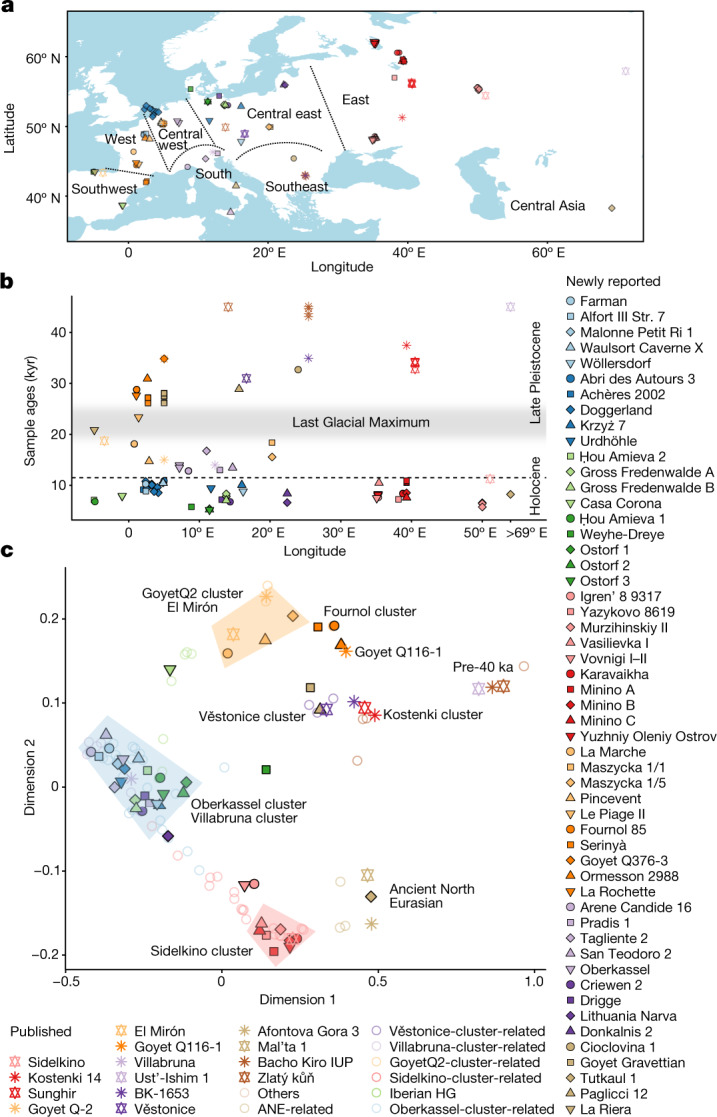
Locations, dates and MDS plot of ancient Eurasian hunter-gatherers. **a**, Geographic locations of newly reported individuals (filled symbols with black outline) and representative previously published individuals (outlined stars). Dotted lines delimit geographic regions described in the text. **b**, Calibrated radiocarbon dates of individuals plotted in **a**. The *y* axis shows the average of calibrated radiocarbon dates in thousands of years (kyr) (Supplementary Data 1.A). The horizontal dashed line marks the boundary between Late Pleistocene and Holocene. **c**, MDS plot of European hunter-gatherers based on 1 − *f*_3_(Mbuti; pop1, pop2). The dimensions are calculated using newly reported and previously published hunter-gatherer groups or individuals with more than 30,000 SNPs. The detailed grouping of individuals shown with empty coloured circles is described in Supplementary Data 1.I.

We generated genome-wide sequencing data for 102 newly reported hunter-gatherers, and increased coverage for 14 previously published individuals^4^. These data cover a time span of around 30,000 years from the Upper Palaeolithic to the Late Neolithic (defined here by the presence of pottery rather than by farming subsistence economy if not indicated), derive from multiple prehistoric cultural contexts, and originate from 54 archaeological sites in 14 countries: 1 Aurignacian-associated individual from Belgium and 1 culturally unassigned individual from Romania (35–33 ka), 15 Gravettian-associated individuals from Spain, France, Belgium, Czechia and Italy (31–26 ka), 2 Solutrean-associated individuals from Spain and France (23–21 ka), 9 Magdalenian-associated individuals from France, Germany, and Poland (18–15 ka), 4 Epigravettian-associated individuals from Italy (17–13 ka), 2 Federmesser-associated individuals from Germany (14 ka), and 81 Mesolithic to Neolithic foragers from across western Eurasia (11–5 ka), together with 1 central Eurasian Neolithic individual from Tajikistan (8 ka) (Fig. 1, Extended Data Table 1, Supplementary Data 1.A, Supplementary Information, section 1 and Supplementary Fig. 1).

We built 1 to 8 single- and double-stranded genetic libraries for each individual and enriched them for human DNA on 1.24 million single nucleotide polymorphisms^6^ (SNPs), which were then sequenced and yielded 0.04- to 7.64-fold coverage on average over the targeted SNPs. Genetic sexing revealed 78 male individuals and 38 female individuals (Supplementary Fig. 12). The levels of contamination from modern human DNA were estimated on the basis of mitochondrial DNA (mtDNA), X chromosome and autosomal DNA, and with a haplotype copying model that is extended here to autosomal data in runs of homozygosity (ROH) (Methods, Supplementary Information, sections 2 and 3, Supplementary Figs. 2–11 and Supplementary Table 1). Substantially contaminated libraries as well as marginally contaminated libraries of individually analysed genomes were filtered to maintain reads showing postmortem DNA damage (Methods and Supplementary Figs. 10 and 11). Pseudo-haploid genotypes were called on the targeted SNPs by randomly sampling a single allele at each position, resulting in individuals with 6,600 to 1.07 million SNPs covered on the 1.24-million-SNP panel (Extended Data Table 1 and Supplementary Data 1.A). The newly generated genotypes were merged with 240 published ancient hunter-gatherer genomes and modern worldwide populations for downstream analyses (Supplementary Data 1.G). Contrary to the proposal in Fu et al.^4^ but in agreement with Petr et al.^17^, we do not observe a substantial decrease of Neanderthal ancestry in most European hunter-gatherers through time (Supplementary Information, section 6 and Supplementary Figs. 15–17). This provides further support for the model with no long-term decline of genome-wide Neanderthal ancestry in modern humans following their introgression^18^.

## Before the LGM

The Gravettian culture was one of the most widely distributed Upper Palaeolithic cultures across western Eurasia before the LGM^19^. It is often considered as a pan-European cultural mosaic with regional variations in material to symbolic productions^20,21^. In this debated framework, Gravettian-associated individuals have been suggested to represent a biologically homogeneous population on the basis of craniometric and genomic data^4,22^. However, published Gravettian-associated genomes originate from central and southern Europe, leaving the genetic profile of Gravettian-associated human groups from western and southwestern Europe undescribed.

**Fig. 2:**
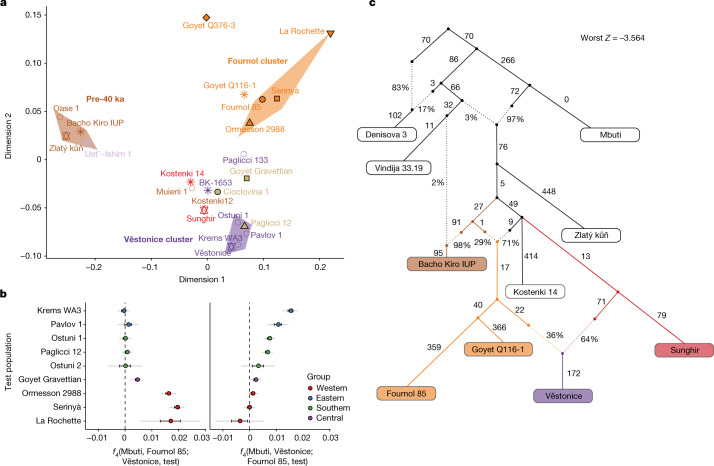
Genetic differences among Gravettian-associated populations. **a**, MDS plot of pre-LGM individuals. The pre-40 ka group and the Fournol and Věstonice clusters are marked as shaded areas in different colours. Individuals and groups are plotted with the same colours and symbols as in Fig. 1 and names are indicated next to the symbols. **b**, Gravettian-associated individuals form two distinct groups, with central-eastern and southern European individuals as part of the Věstonice cluster and western and southwestern European individuals as part of the Fournol cluster. In central-western Europe, Gravettian-associated individuals from Goyet show affinity to both clusters. Error bars show 1× s.e.m. (black) or 3× s.e.m. (grey) of the *f*_4_ values estimated from 5 cM-block jackknife analysis. **c**, Admixture graph modelling of the main pre-LGM European hunter-gatherer lineages created using qpGraph.

**Extended Data Fig. 1:**
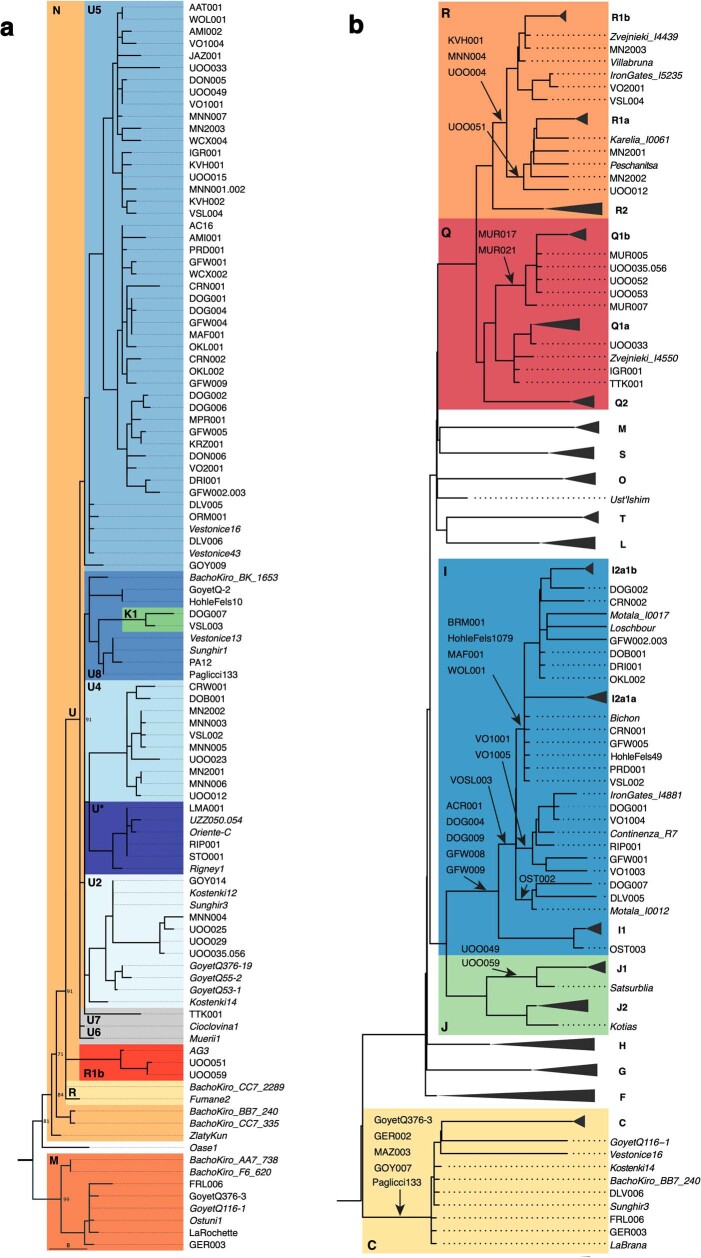
MtDNA and Y-chromosome phylogenies. Bold letters refer to (**a**) mtDNA and (**b**) Ychr haplogroups, whose boxes are coloured according to the legend in Extended Data Fig. 2. The labels in italic denote previously published individuals without new data generated in this study.

**Extended Data Fig. 2:**
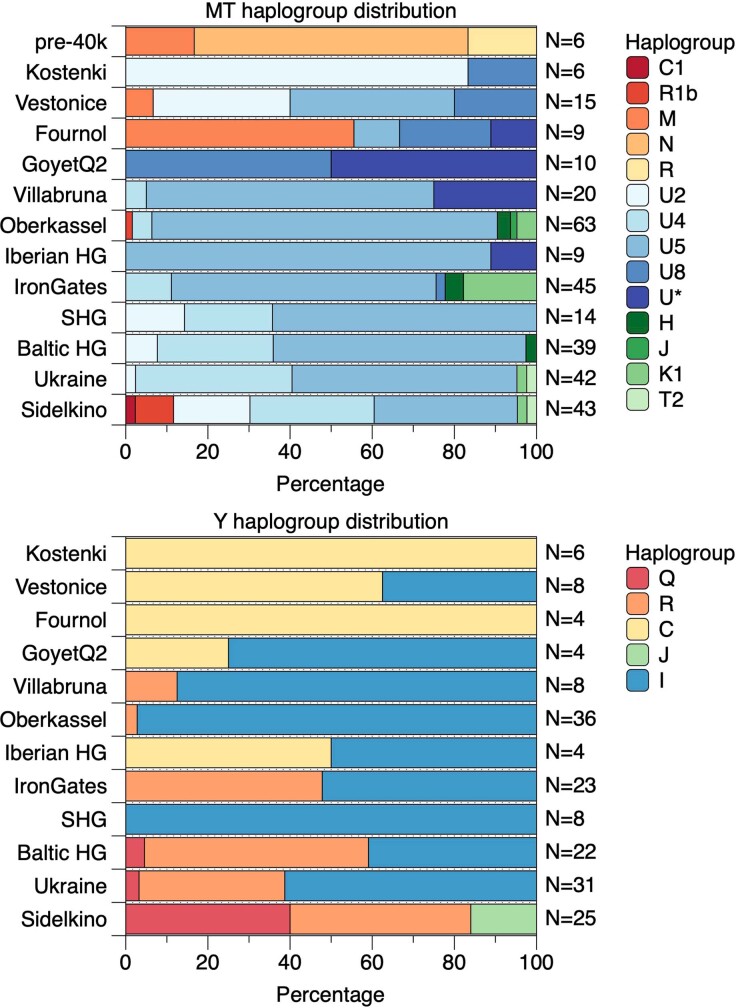
The distribution of mtDNA and Y-chromosome haplogroups among different hunter-gatherer groups. The length of each coloured bar represents the fraction of individuals carrying the corresponding haplogroup (legend on the right of each panel). The number of individuals in each group is written to the right of each bar. We only plotted groups with more than two individuals and, for this reason, individuals from the GoyetQ116-1 cluster are included here into the Fournol cluster.

To gain an overview of the genomic background of European hunter-gatherers before the LGM, we used multidimensional scaling (MDS) to plot a dissimilarity matrix of pairwise outgroup *f*_3_-statistics in the form 1 − *f*_3_(Mbuti; pop1, pop2) (Fig. 2a). This plot reveals the presence of three distinct groupings: (1) a pre-40 ka group with individuals from the Ust’Ishim, Bacho Kiro, Zlatý kůň and Peştera cu Oase sites, (2) a Věstonice cluster including Gravettian-associated individuals from central–eastern and southern European sites (Dolní Věstonice, Pavlov, Krems-Wachtberg, Paglicci and Ostuni), and (3) a Fournol cluster (hereafter, Fournol cluster or ancestry) comprising Gravettian-associated individuals from western and southwestern European sites (Ormesson, La Rochette, Fournol and two Serinyà cave sites (Mollet III and Reclau Viver)). The previously described Věstonice cluster, including a newly reported 29,000-year-old individual from Paglicci cave (Paglicci 12) in southern Italy, is closely related to the previously published genomes from Sunghir and Kostenki 12 in western Russia, which are dated to 34 ka and 32 ka, respectively^4,23^. The newly defined Fournol cluster is closely related to Aurignacian-associated individuals from Belgium dated to 35 ka (Goyet Q116-1 and the newly reported Goyet Q376-3 individual). Notably, and contrary to the report by Fu et al.^4^, another Gravettian-associated population from central–western Europe (Goyet in Belgium, *n* = 6 individuals) is both geographically and genetically intermediate between the Věstonice and Fournol clusters. The similarity between Goyet Q116-1 and Goyet Q376-3 and the Fournol cluster is also observed at the mtDNA level, with both groups including individuals who carried mtDNA haplogroup M, which has not been found in European individuals from after the LGM^24^ (Extended Data Figs. 1 and 2).

**Extended Data Fig. 3:**
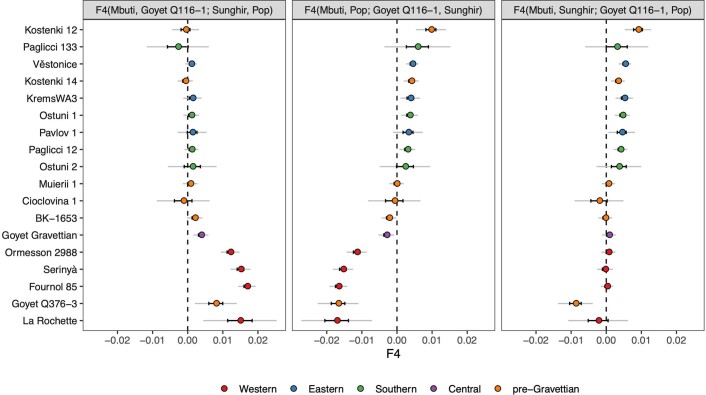
*F*_*4*_-statistics comparing the affinity of pre-LGM European hunter-gatherers to Goyet Q116-1 and Sunghir. The colours correspond to the grouping of tested populations, dots refer to the *f*_4_-values and the dark and light error bars to 1*SE and 3*SE estimated from 5 cM block jackknife, respectively. This figure shows that the Gravettian-associated individuals from western Europe (Fournol cluster) are closely related to Goyet Q116-1 while the Gravettian-associated individuals from central-eastern and southern Europe (Věstonice cluster) are closely related to the Sunghir group, representative of the Kostenki cluster. Details are provided in Data S2.B.

We further validated the genetic distinction between the Věstonice and Fournol clusters observed in the MDS plot with a series of *f*_4_-statistics (Supplementary Data 2.B). All individuals belonging to the Fournol cluster show higher affinity to Goyet Q116-1 than to the Sunghir group (*n* = 4), and the Věstonice-cluster individuals show higher affinity to the Sunghir group than to Goyet Q116-1 (Extended Data Fig. 3). These *f*_4_-statistics also confirm that Goyet Q376-3 carries a similar ancestry to Goyet Q116-1 and Kostenki 12 carries a similar ancestry to the Sunghir group, whereas Bacho Kiro 1653 (35 ka) from Bulgaria, Muierii 1 (34 ka) and Cioclovina 1 (32 ka) from Romania, and Paglicci 133 (33 ka) from southern Italy are equally related to Goyet Q116-1 and Sunghir. We further tested whether individuals included in the Věstonice and Fournol clusters share similar allele frequencies with the main representatives of those two clusters. With the statistics *f*_4_(Mbuti, Fournol 85; Věstonice, test) and *f*_4_(Mbuti, Věstonice; Fournol 85, test), we show that all Věstonice-cluster individuals are significantly closer (|*Z*|>3) to the Věstonice group (*n* = 5) and the Fournol-cluster individuals are closer to Fournol 85, whereas the geographically intermediate Gravettian-associated Goyet group shows extra affinity to both clusters (Fig. 2b).

**Extended Data Fig. 4:**
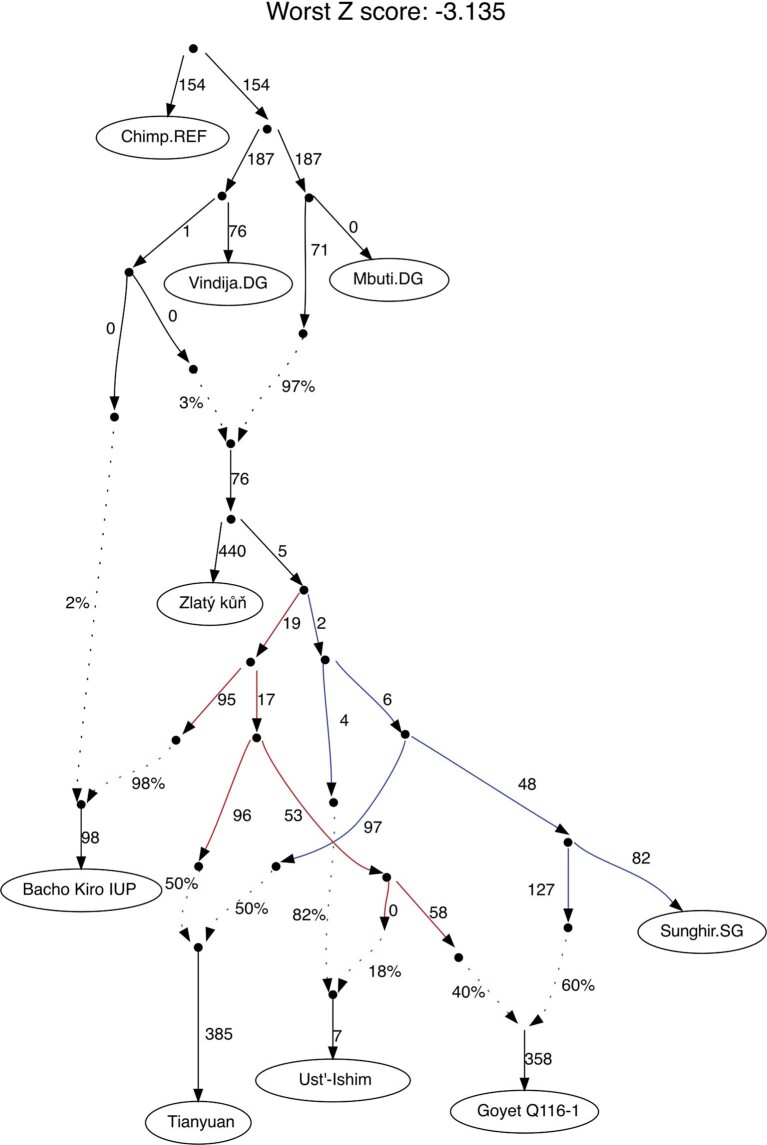
Admixture graph modelling of pre-34 ka hunter-gatherer lineages. In this admixture graph, the lineage related to Zlatý kůň splits more basally than the Bacho Kiro IUP group, who contributes to Tianyuan, Ust’Ishim and Goyet Q116-1 (indicated with red lines), but not to the Sunghir group.

We further modelled the genetic profile of pre-LGM individuals with qpGraph (Supplementary Information, section 10 and Supplementary Figs. 19–25). The admixture graph shows that the Bacho Kiro IUP group (*n* = 3) shares ancestry with multiple early modern human lineages^2^ (Supplementary Information, section 7), and that the more than 45,000-year-old Zlatý kůň genome^1^ is the most deeply divergent non-African lineage sequenced to date (Extended Data Fig. 4). This is also validated by *f*_4_-statistics of the form *f*_4_(Mbuti, Zlatý kůň; test1, test2), which are consistent with zero for all other pre-LGM hunter-gatherers (Supplementary Data 2.C), indicating an equidistant relationship of Zlatý kůň to the tested groups. When Gravettian-associated individuals are included in an admixture graph also featuring Kostenki 14, we find that Fournol 85 fits best as a sister lineage of Goyet Q116-1, whereas the Věstonice group is modelled as a mixture between a lineage related to the Sunghir group and one related to the Goyet Q116-1–Fournol 85 branch (Fig. 2c). This is also supported by *f*_4_-statistics of the form *f*_4_(Mbuti, Fournol 85; Sunghir, test), which are significantly positive for all the individuals included in the Věstonice cluster (Supplementary Data 2.B). Therefore, as previously reported^2^, the Věstonice cluster itself results from admixture between western and eastern lineages, which might contribute to the observed homogeneity in cranial morphology among Gravettian-associated individuals^22^.

These results show that some, but not all, of the genomic ancestries present in Europe between around 40 ka and 30 ka survived in the Gravettian-associated populations studied so far. The Kostenki (and Sunghir group) ancestry contributed to the previously described Věstonice cluster represented by Gravettian-associated individuals from central-eastern and southern Europe^4^. By contrast, the Goyet Q116-1 genetic profile gave rise to the newly described Fournol cluster identified in Gravettian-associated individuals from western and southwestern Europe. Notably, this genetic distinction coincides with dissimilarities in mortuary practice among genetically analysed Gravettian-associated individuals from different parts of Europe. Individuals in western and southwestern Europe related to the Fournol cluster are consistently deposited in cave sites and occasionally exhibit anthropogenic marks whereas individuals related to the Věstonice cluster are buried with grave goods and/or personal ornaments and ochre in open air or cave sites in central-eastern and southern Europe, respectively (Supplementary Figs. 29–32 and Supplementary Table 4). The oldest individual in the Fournol cluster is Ormesson 2988 from northeastern France (31 ka, Early/Middle Gravettian), whereas a Gravettian group from Goyet in Belgium (27 ka, Late Gravettian) is found to be a mixture between the Věstonice and Fournol clusters. This suggests that between the Early/Middle and Late Gravettian there was an east-to-west expansion of the Věstonice-associated ancestry that reached central-western Europe and created a longitudinal admixture cline between those two genetically distinct pre-LGM populations.

## LGM in southwestern and western Europe

The Solutrean culture is temporally intermediate between the Gravettian and the Magdalenian (or the Badegoulian) cultures, and is found in southwestern and western Europe, which are considered to have been climatic refugia for human populations during the LGM^25,26^. However, the extent to which groups associated with the Solutrean culture are in genetic continuity with earlier and later populations from the same region is unknown because no genomic data from Solutrean-associated individuals have been reported previously. Both newly sequenced genomes from Solutrean-associated individuals (Le Piage II (23 ka) from southwestern France and La Riera (level 14, 21 ka) from northern Spain) show a generalized affinity with members of the Fournol and GoyetQ2 clusters in outgroup *f*_3_-statistics (Supplementary Data 2.A). In the MDS plot, the Le Piage II individual falls particularly close to individuals belonging to the Fournol cluster, suggesting a local genetic continuity of this ancestry during the LGM (Supplementary Fig. 13). *F*_4_-statistics further support this view, revealing that Le Piage II is more closely related to the Fournol cluster than the Věstonice cluster (*f*_4_(Mbuti, Le Piage II; Věstonice, Fournol 85) ≫ 0, *Z* = 6.58). We also compared its affinity to El Mirón (northern Spain), the oldest Magdalenian-associated individual sequenced to date (19 ka). *F*-statistics suggest that Le Piage II is genetically intermediate between Fournol 85 and El Mirón (Supplementary Data 2.D). Moreover, previous studies have shown that El Mirón carries a genetic contribution from the Villabruna cluster, which is found in Epigravettian-associated individuals from Italy^4,15^. El Mirón has a significantly higher similarity to the Villabruna cluster than Fournol 85 and Le Piage II, while the affinity to the Villabruna cluster in Le Piage II is not significantly higher than in Fournol 85 (Supplementary Data 2.D). Overall, the Solutrean-associated Le Piage II individual links the preceding Fournol ancestry with the succeeding ancestry found in El Mirón, providing direct evidence for genetic continuity throughout the LGM in southwestern and western Europe. These European regions, therefore, constitute climatic refugia where human populations survived during the LGM.

## Post-LGM in the Italian peninsula

After the LGM, the Epigravettian culture was widespread in southern and southeastern Europe. In spite of growing discussions about its nature^27,28^, the Epigravettian culture has been traditionally assumed to be the result of a transition from the preceding local Gravettian^29^. However, the level of genetic continuity between individuals associated with these cultures and the population structure among Epigravettian-associated individuals have not been fully explored. Here, we report genomic data from 4 individuals, including 3 approximately 13,000-year-old genomes from northeastern Italy (Pradis 1), northwestern Italy (Arene Candide 16) and Sicily (San Teodoro 2), as well as increased genome-wide coverage from Tagliente 2^15^ dated to 17 ka.

In the MDS plot, we find that all of the newly and previously reported Epigravettian-associated individuals fall within the Villabruna cluster^4^ (Fig. 1c). A series of *f*_4_-symmetry statistics confirm that all the Epigravettian-associated individuals are cladal, and do not share excess affinity with any local (Paglicci 12) or non-local preceding ancestries (Goyet Q116-1, Kostenki 14, Mal’ta 1 or Věstonice) (Supplementary Data 2.F). Moreover, none of the Epigravettian-associated individuals have more affinity to southern European than to central-eastern European Gravettian-associated groups, as shown by *f*_4_(Mbuti, Epigravettian-associated individual/group; Věstonice, Paglicci 12) that is consistent with 0 (Supplementary Data 2.G).

**Fig. 3:**
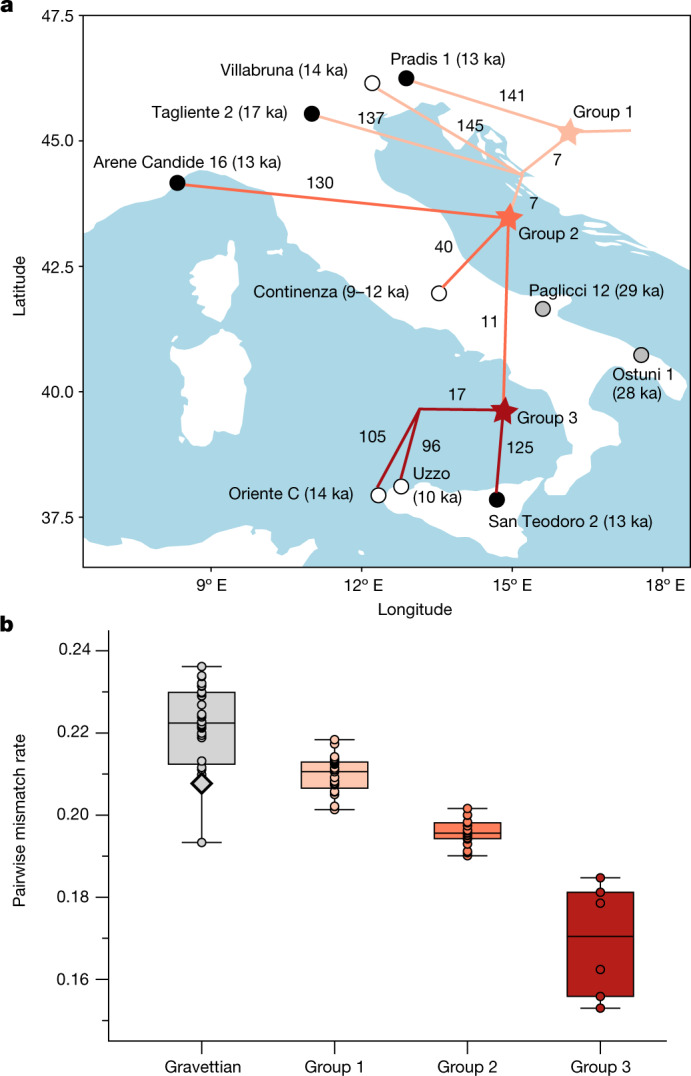
The population substructure and diversity of Epigravettian-associated groups in southern Europe. **a**, Population structure among Epigravettian-associated populations revealed by a neighbour-joining tree based on pairwise *f*_2_ genetic distances. Branch labels show unique drift lengths; black dots refer to individuals with newly generated data and white dots refer to previously published genomes; the position of each node does not imply the location where the split took place. **b**, Population diversity shown by PMR between individuals in different groups. The grouping of Epigravettian-associated populations is shown with the same colour in **a**. The black-outlined diamond marked out in the Gravettian group shows the PMR between the two Gravettian-associated individuals from southern Italy (Paglicci 12 and Ostuni 1). In the box plot the centre line is the median, box bounds delineate the interquartile range and whiskers extend to maximum and minimum values, excluding outliers. The sample size of individual pairs included in each group is reported in Supplementary Data 3.A.

Next, we investigated the genetic relationships between Epigravettian-associated individuals across the Italian peninsula, by reconstructing a phylogeny based on a matrix of pairwise *f*_2_ genetic distances (Fig. 3a and Supplementary Fig. 9) and testing the relative affinity among them using *f*_4_-statistics in the form *f*_4_(Mbuti, Epigravettian A; Epigravettian B, Epigravettian C) (Supplementary Data 2.E). The inferred topology reveals a phylogeographic pattern irrespective of individual ages. In particular, the 13 ka Pradis 1 individual from northeastern Italy represents the most basal lineage compared to all other Epigravettian-associated individuals, including the older Tagliente 2 and Villabruna genomes from northern Italy (group 1). Individuals from northwestern Italy (Arene Candide 16), central Italy (Continenza) and Sicily fall on a phylogenetically more derived branch (group 2), which further diversified into a branch composed of Sicilian hunter-gatherers only (group 3). Within Sicily, the 14 ka Oriente C individual shows higher affinity with the much younger but geographically closer 10 ka Uzzo group^30^ (*n* = 2) than with the almost contemporaneous San Teodoro 2 individual from eastern Sicily.

**Extended Data Fig. 5:**
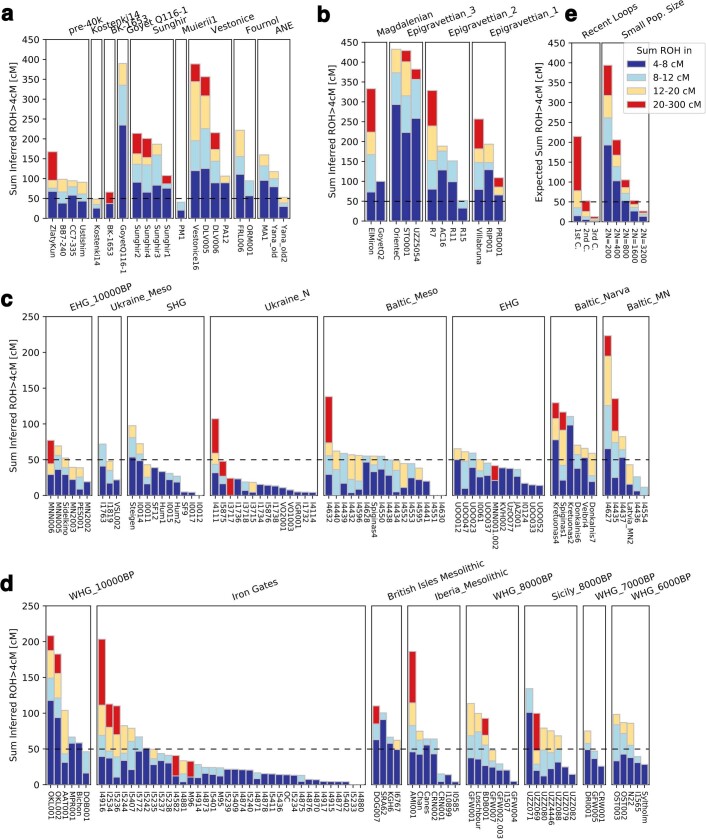
Summary of ROH segments detected in Eurasian hunter-gatherers. We visualize the total amount of ROH longer than 4 cM for (**a**) pre-LGM individuals, (**b**) Epigravettian- and Magdalenian-associated individuals, (**c**) individuals carrying high proportions of Sidelkino-related ancestry, and (**d**) individuals carrying high proportions of Oberkassel-related ancestry. Colour legend is shown in (**e**). Each bar represents one individual with the ROH grouped in four length categories (grouped by colour). The inferred pattern of short ROH (4–8 cM, visualized in blue) being common is in stark contrast to most later farmer populations, where the majority of individuals have no short ROH whatsoever (see^101^), and evidences small effective population sizes across West Eurasian hunter-gatherer groups. A dashed line of 50 cM total ROH is drawn in each panel to help comparison between panels with different y-axis scales. Details of the grouping and ROH segments are provided in Data S3.B.

Finally, we estimated the genetic diversity of Epigravettian-associated individuals in the dataset by calculating both pairwise mismatch rates (PMR) on pseudo-haploid genotypes and individual heterozygosity levels on pseudo-diploid genotypes (Supplementary Data 3.A). Compared with the genetic diversity observed among all analysed Gravettian-associated groups, Epigravettian-associated individuals show significantly lower amounts of genetic diversity (two-tailed *t*-test, *P* < 0.001) (Fig. 3b). Moreover, we reveal a north-to-south decrease in genetic diversity among the Epigravettian-associated groups, with the highest PMR and heterozygosity values found in northern Italian individuals (group 1), intermediate in western and central Italian individuals (group 2) and the lowest in Sicilian individuals (group 3) (Fig. 3b). A similar pattern is observed through the analysis of ROH segments (Extended Data Fig. 5 and Supplementary Information, section 9). We detect the highest amount of ROHs in Epigravettian-associated individuals from Sicily, who carry an extreme amount of more than 200 cM of short ROHs (4–8 cM). This suggests a very small recent effective population size, estimated to be in the order of around 70 individuals (Supplementary Table 2), causing the low genetic diversity in Sicilian Epigravettian hunter-gatherers.

To summarize, our results highlight a genetic turnover in the Italian peninsula of the Gravettian-associated Věstonice cluster by the Epigravettian-associated Villabruna cluster that might correlate with discontinuities observed in the archaeological record^31^. We show that all analysed Epigravettian-associated individuals carry a homogeneous Villabruna ancestry, with the intra-group genetic structure mainly determined by their geographical, and not temporal, distribution. The phylogenetic reconstruction of Epigravettian-associated genomes, with Pradis 1 diverging more deeply than all others, indicates that the turnover took place much earlier than 17 ka—the date of the more derived Tagliente 2 genome. This, together with the evidence of Villabruna ancestry in El Mirón 19 ka, further suggests that this genetic discontinuity could be the result of palaeogeographic and palaeoecological transformations connected to the LGM^32^, rather than to the Bølling–Allerød warming period^4,15^ (14.7–12.9 ka). In addition, our phylogeographic analysis points to northeastern Italy as the possible entry point of the Epigravettian-associated gene pool in the Italian peninsula. This finding, in conjunction with the genetic affinity of the Villabruna cluster to ancient and present-day Near Eastern ancestries^4,15,16^ (Supplementary Information, section 8, Supplementary Fig. 18 and Supplementary Data 2.O), suggests the Balkans as a source of the incoming Epigravettian-associated population. The LGM could thus have created a corridor south of the Alps for east-to-west human movements that genetically connected hunter-gatherer populations from the Balkans to Iberia, possibly also via dispersals along existing lower-sea-level coasts^32^.

## Post-LGM in western and central Europe

The Magdalenian culture was widely distributed in southwestern, western and central Europe after the LGM^33^. Despite this wide geographical range, it is not clear whether different groups associated with this culture originated from a common source population and how those groups were genetically related to each other. Previous studies identified two different genetic compositions in Magdalenian-associated individuals—the GoyetQ2 cluster including central-western European genomes dated to around 15 ka (from France, Belgium and Germany), and the ancestry of the El Mirón individual from Spain^4,14^ from around 19 ka. Both of these ancestries carry a genetic component distantly related to the Goyet Q116-1 individual dated to 35 ka, with the Iberian individual also showing an affinity to the Villabruna cluster^4,14^. By co-analysing previously published data with our newly reported genomes associated with the Magdalenian from La Marche (18 ka) and Pincevent (15 ka) in western and northern France, respectively, and Maszycka (18–16 ka) in southern Poland, we confirm that the Goyet Q116-1 ancestry survived in all studied Magdalenian-associated genomes besides in Gravettian and Solutrean-associated individuals from southwestern and western Europe (Fig. 1). Notably, the Fournol ancestry provides a better proxy than Goyet Q116-1 for the genetic component found in the GoyetQ2 cluster and in El Mirón (Supplementary Data 2.H). However, using *f*_4_-statistics, we show that all Magdalenian-associated individuals, and not only El Mirón, carry Villabruna-related ancestry when compared to the Fournol cluster (Supplementary Data 2.H). This affinity is even stronger towards Epigravettian-associated individuals from western and central Italy and Sicily (group 2 and group 3, respectively) than to those from northern Italy (group 1) (Supplementary Data 2.F).

**Fig. 4:**
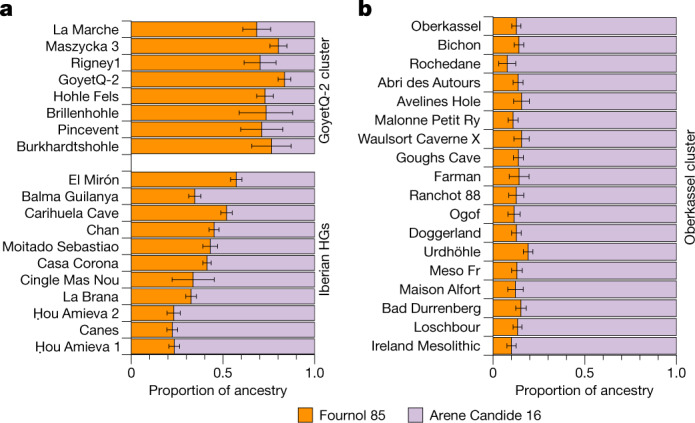
Ancestry modelling of post-19 ka individuals in southwestern, western and central Europe. **a**,**b**, The ancestries of individuals in the GoyetQ-2 cluster and Iberian hunter-gatherers (HGs) (**a**) and individuals in the Oberkassel cluster (**b**) were modelled using qpAdm, with Fournol 85 and Arene Candide 16 representing the Fournol and Villabruna ancestries, respectively. The length of the colour bar shows the proportion of each ancestry. The error bar shows the s.e.m. of estimates from 5-cM-block jackknife analysis. Details of the modelling are provided in Supplementary Data 3.C.

We thus modelled individuals belonging to the GoyetQ2 cluster and El Mirón as a mixture between the Fournol 85 and Arene Candide 16 genomes as proxies to represent the Fournol and Villabruna ancestries, respectively, in Magdalenian-associated groups (Fig. 4a). Besides El Mirón, who has around 43% Villabruna ancestry, all other Magdalenian-associated individuals have a lower proportion of this component (19–29%) and can thus be assigned to the GoyetQ2 cluster (Fig. 4a and Supplementary Data 3.C). This is further validated by *f*_4_-statistics of the form *f*_4_(Mbuti, Arene Candide 16; Goyet Q-2, Magdalenian-associated individuals), which is significantly positive only for El Mirón, whereas all other tested individuals and Goyet Q-2 are symmetrically related with respect to Arene Candide 16 (Supplementary Fig. 26 and Supplementary Data 2.H).

Our analyses demonstrate that the Fournol cluster is a better source for Magdalenian-associated genomes than Goyet Q116-1. Therefore, most of the ancestry found in these post-LGM individuals probably traced back to Gravettian-associated groups from western and southwestern Europe. The genetic affinity to the Villabruna ancestry is present in El Mirón and in Magdalenian-associated individuals from western and central Europe. This suggests that genetic links between southern and southwestern European hunter-gatherers around the time of the LGM extended north of the Pyrenees. The resulting GoyetQ2 cluster includes individuals spanning from western France to Poland in the period between 18 and 15 ka. Therefore, contrary to previous suggestions^34^, this demonstrates that the post-LGM diffusion of the Magdalenian was indeed associated with northward and northeastward population expansions from western Europe^35^.

## Post-14 ka to Neolithic

Previous studies have shown that two main hunter-gatherer ancestries were predominant across most parts of Europe after around 14 ka—that is, the western hunter-gatherer (WHG) ancestry, related to the Villabruna cluster, and the eastern hunter-gatherer (EHG) ancestry, showing affinity to both the Villabruna and the ancient north Eurasian (ANE) ancestry found in Upper Palaeolithic Siberian individuals^4,36^. Hunter-gatherers carrying an admixed WHG/EHG genetic profile have been sequenced from various regions of northern and eastern Europe, raising the question of how these two types of ancestries formed and interacted with each other through time and space^37–40^.

**Extended Data Fig. 6:**
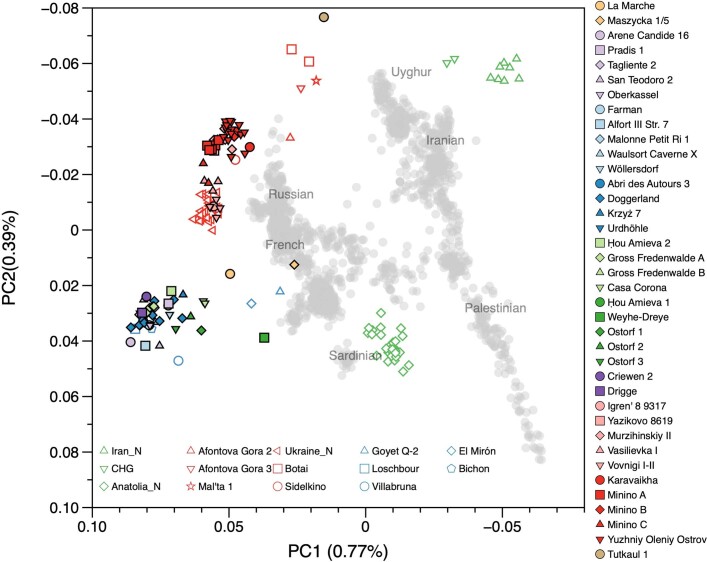
West Eurasian PCA showing the genetic positioning of post-LGM hunter-gatherers. Present-day individuals (gray dots) genotyped on the Human Origins dataset are used to define the PCA variation onto which ancient genomes (coloured symbols) are projected. The newly reported individuals with over 15,000 SNPs on the Human Origins dataset are shown in black-outlined and filled symbols, as illustrated in the legend on the right, while representative ancient genomes are shown in outlined symbols, as illustrated in the legend at the bottom of the PCA.

In the MDS plot (Fig. 1c) and a west Eurasian principal component analysis (PCA) (Extended Data Fig. 6 and Supplementary Fig. 14), most post-14 ka individuals from western and central Europe fall close to the WHG cluster and those from eastern Europe close to the EHG cluster, whereas the Tutkaul 1 individual from central Asia falls close to the ANE-related group. The two 14 ka Oberkassel individuals mark the earliest presence of WHG ancestry north of the Alps, which we therefore rename the Oberkassel cluster (hereafter, Oberkassel cluster or ancestry), using the name of the oldest reported individual to date carrying such ancestry with more than one-fold coverage, for consistency^4^. On the basis of *f*_4_-statistics, we find that individuals assigned to the Oberkassel cluster are closer to the Arene Candide 16 genome than any other Epigravettian-associated group from Italy (Supplementary Data 2.F). Moreover, the Oberkassel cluster carries both Villabruna ancestry and a contribution from GoyetQ2 ancestry (Supplementary Data 2.J). This was confirmed with qpAdm, in which we could model all individuals from the Oberkassel cluster as a broadly constant mixture of approximately 75% Arene Candide 16 and 25% Goyet Q-2 (or 90% Arene Candide 16 and 10% Fournol 85) (Fig. 4b and Supplementary Data 3.C). The observation that post-14 ka individuals from western and central Europe and also from Britain^41^ carry a homogeneous genetic makeup instead of displaying repeated local admixtures with GoyetQ2 ancestry implies that the Oberkassel-ancestry profile was already largely formed before its dispersal. This is in sharp contrast to the genetic history of Iberian hunter-gatherers, where the spread of the Villabruna/Oberkassel ancestry involved multiple local admixture events with groups carrying high proportions of GoyetQ2 ancestry^14^ (Fig. 4 and Supplementary Data 3.C). The long-lasting genetic continuity in Iberia is also reflected in the preservation until the Mesolithic of Y-chromosome haplogroup C, which was predominant in pre-LGM groups but rarely found after the LGM in other parts of Europe (Extended Data Figs. 1 and 2).

Using *f*_4_-statistics and qpAdm, we confirm that EHG populations in eastern Europe are a mixture of Villabruna/Oberkassel and ANE ancestries (Supplementary Information, section 11 and Supplementary Data 2.K). *F*_4_-statistics also show that the approximately 8.2 ka Yuzhniy Oleniy Ostrov group from Karelia in western Russia formed by 19 genomes has comparable or lower affinity to Villabruna ancestry than all the other EHG groups (Supplementary Data 2.K). The oldest individual revealing an indistinguishable genetic profile from the Yuzhniy Oleniy Ostrov group is the 11 ka Sidelkino individual from Samara in western Russia^42^. For consistency with the previously discussed nomenclature, we rename the EHG ancestry as the Sidelkino cluster (hereafter, Sidelkino cluster or ancestry). The genetic distinction between the Oberkassel and Sidelkino clusters is also clearly noticeable in the diversity of uniparentally inherited markers, as the Oberkassel cluster is dominated by mtDNA haplogroup U5 and Y-chromosome haplogroup I, whereas individuals from the Sidelkino cluster show a higher frequency of mtDNA haplogroups U2, U4 and R1b, and carry uniquely Y-chromosome haplogroups Q, R and J (Extended Data Figs. 1 and 2).

**Fig. 5:**
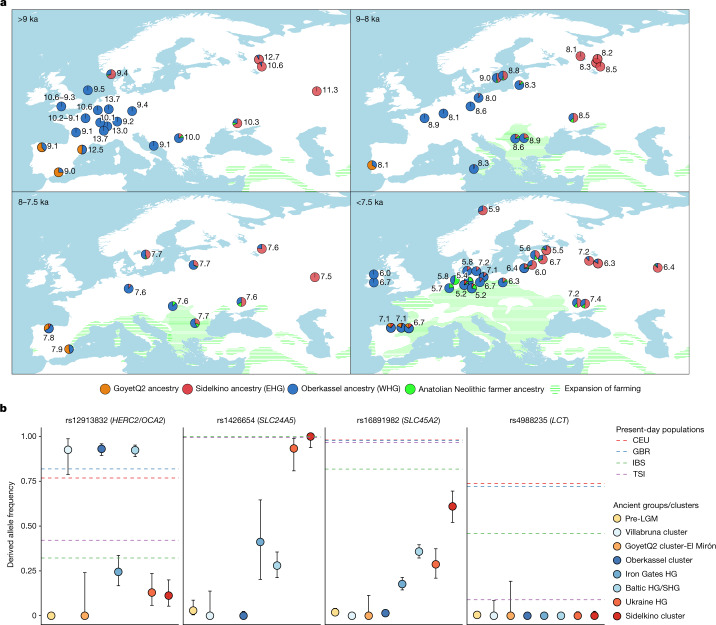
Ancestry modelling of hunter-gatherers from 14–5.2 ka and their allele frequencies on phenotypic SNPs. **a**, The genetic ancestry of hunter-gatherers dated between 14 ka and 5.2 ka modelled using qpAdm, with Oberkassel, Yuzhniy Oleniy Ostrov, Goyet Q-2 and Neolithic farmers from present-day Turkey (Barcın, Menteşe and Boncuklu sites) representing Oberkassel (WHG) (blue), Sidelkino (EHG) (red), GoyetQ2 (orange) and Anatolian Neolithic farmer (green) ancestries, respectively. The average calibrated date is shown, with pie charts indicating the estimated proportion of ancestry for each group or individual. Details of the modelling are provided in Supplementary Data 3.E,F. The expansion of farming by 9, 8, 7.5 and 7 ka is shown as green shades. Adapted from 10.5281/zenodo.5903165 (CC BY 4.0). **b**, Allele frequencies of different hunter-gatherer groups (coloured dots) on four SNPs related to skin colour (*SLC24A5* and *SLC45A2*), eye colour (*HERC2*/*OCA2*) and lactase persistence (*LCT*). Dots are maximum likelihood estimates and error bars show 95% confidence intervals of the derived allele frequencies (*n*, the number of individuals in each group, is provided in Supplementary Data 3.G). Dashed lines show the frequencies estimated for the indicated present-day 1000 Genomes Project populations (CEU, Utah residents of northern and western European ancestry; GBR, British; IBS, Spanish; TSI, Tuscan )^37^. Details on the allele frequency estimates are provided in Supplementary Information, section 12, Supplementary Figs. 27 and 28 and Supplementary Data 3.G.

**Extended Data Fig. 7:**
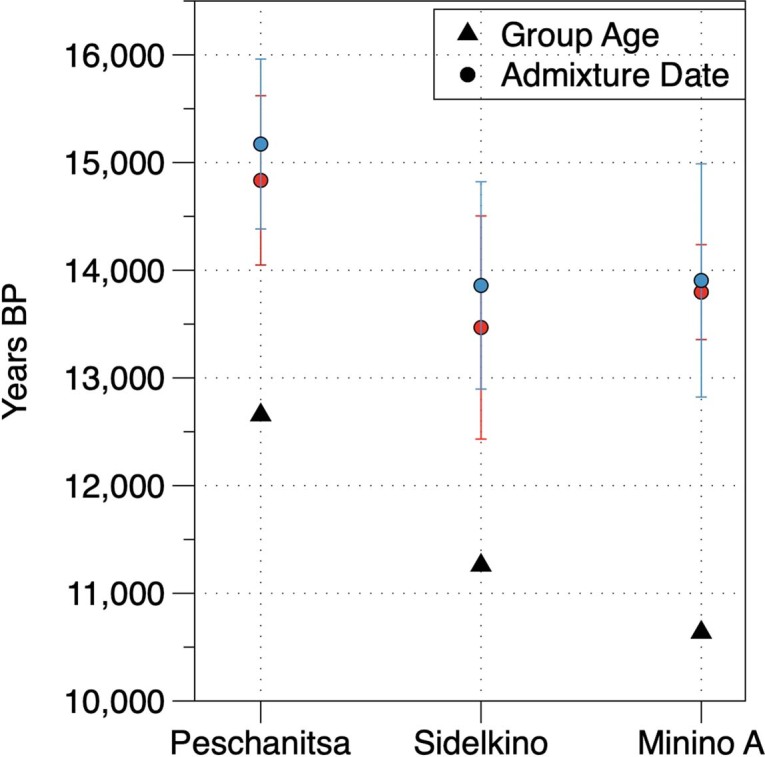
Admixture dates between Oberkassel/Villabruna and ANE ancestries in the oldest individuals from the Sidelkino cluster. The triangles show the average calibrated dates of the tested groups and the dots show the estimated admixture dates with the software DATES^105^ using Oberkassel (red dots) or Villabruna (blue dots) clusters as one source and ANE-related individuals as the other source population. The generation time is set to 29 years and the error bars show the SE of the admixture date estimated from jackknife resampling (*n *= 22 autosomal chromosomes). Additional details are provided in Supplementary Information, section 11 and Supplementary Table 3.

We then attempted to model 250 published and newly reported hunter-gatherers dated to 14–5 ka using qpAdm as a mixture of Oberkassel, Sidelkino, GoyetQ2 ancestries, and an ancestry maximized in Anatolian Neolithic farmers (ANF), as a considerable portion of the sequenced hunter-gatherer genomes date after around 8 ka, when ANF ancestry started spreading across Europe. Our results show that the contact zone and the admixture patterns between the Oberkassel and Sidelkino ancestries changed over time (Fig. 5). Between 14 and 8 ka, all hunter-gatherers in western and central Europe carried only Oberkassel ancestry, with no detectable contribution from the Sidelkino cluster. Further north and east, individuals from the Baltics (Baltic HG), Scandinavia (SHG), the Balkans (Iron Gates HG) and Ukraine (Ukraine HG) already carried an Oberkassel/Sidelkino admixed ancestry^38,40^ before 8 ka. In addition, those groups also carry affinity to ANF suggesting more complex genetic processes behind their demographic history^16^. Moreover, two of the oldest published groups from western Russia belonging to the Sidelkino cluster—Peschanitsa (13 ka)^43^ and the newly reported Minino individuals (11 ka)—showed extra affinity to the Oberkassel cluster, possibly owing to variability in this ancestry proportion during the initial formation phase of the Sidelkino-ancestry profile. Using DATES software, we estimated the admixture between Villabruna/Oberkassel and ANE ancestries in these old Sidelkino-cluster-related individuals to around 15–13 ka (Extended Data Fig. 7 and Supplementary Table 3), which coincides roughly with the first appearance of the Oberkassel ancestry in central Europe. This raises the possibility that the replacement by the Oberkassel cluster and the formation of the Sidelkino cluster might have been the result of population expansions influenced by the abrupt warming during the Bølling–Allerød interstadial^4,24^.

**Extended Data Fig. 8:**
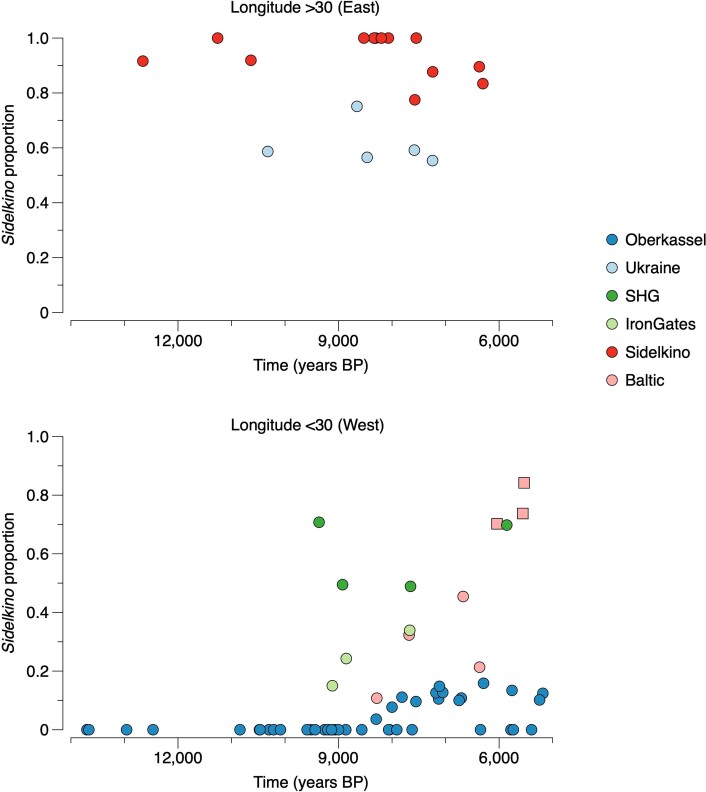
Changes of Sidelkino and Oberkassel ancestry proportions in post-14 ka hunter-gatherers. The bivariate plots show the expansion of Oberkassel and Sidelkino ancestries through time in two European areas (longitude below and above 30 degrees). The x-axis shows the average age of each tested individual/group and the y-axis shows the proportion of Sidelkino ancestry, relative to the total hunter-gatherer ancestry (Oberkassel + Sidelkino) in each group. The three squares highlight Baltic HG groups associated with the Comb Ceramic Culture (CCC) that show a marked increase in Sidelkino-related ancestry compared to older Baltic HG groups.

From around 8 ka, we begin to observe admixture events with Sidelkino ancestry in central Europe. This is first detected in an individual from Gross Fredenwalde in northeastern Germany and reaches around 10% in most European hunter-gatherer individuals thereafter (Extended Data Fig. 8). Soon after 8 ka, Sidelkino ancestry was absent in eastern Spain but it had already reached northern Iberia alongside an increase in Oberkassel ancestry (Fig. 5). Conversely, additional Oberkassel ancestry is identified in eastern Europe by at least 7.5 ka in newly generated genomes from Minino I and Yazykovo from the upper Volga region, whereas a 1,000-years-older individual from Minino I did not have this genetic component. Considering a freshwater reservoir signal in the upper Volga region making radiocarbon dates on human remains appear up to about 500 years older than their true age^44^, there could be an interval of more than 1,000 years between the first evidence of admixture in central European hunter-gatherers with Sidelkino ancestry and eastern European hunter-gatherers with Oberkassel ancestry. However, additional genomes intermediate in time and space are needed to assess whether those two admixture events were independent or part of a common demographic process.

After 7.5 ka, as ANF ancestry had reached regions north of the Alps, individuals carrying a hunter-gatherer genetic profile were primarily restricted to the northern fringes of Europe (Fig. 5). In this period, the Oberkassel-ancestry admixture spread further east, reaching Samara by around 6.5 ka, and an increase in Sidelkino ancestry was detected in hunter-gatherers from the Baltic region, which was previously associated with the transition from the Narva culture to the Comb Ceramic culture^38,39^ (Extended Data Fig. 8). In central Europe, admixture with ANF ancestry became highly common but not ubiquitous, indicating the co-existence of hunter-gatherer and farmer societies without admixing for several hundred years. The youngest individual carrying large portions of hunter-gatherer ancestry in the analysed dataset is from Ostorf in northern Germany, dated to around 5.2 ka (>90% Oberkassel cluster plus Sidelkino-cluster components) (Supplementary Data 3.F). Individuals at this site might mark one of the last occurrences of such high levels of hunter-gatherer-related ancestries, just centuries before the emerging European Bronze Age.

On the basis of PCA and outgroup *f*_3_-statistics, the Neolithic Tutkaul 1 individual from Tajikistan is closely related to Upper Palaeolithic individuals from south-central Siberia (Afontova Gora 3 (AG3) and Mal’ta 1), and roughly contemporaneous West Siberian hunter-gatherers (Tyumen and Sosnoviy), both carrying high proportions of ANE ancestry^45^ (Fig. 1c and Extended Data Fig. 6). We tested the affinity of Tutkaul 1 to worldwide ancient and modern populations relative to AG3. Contrary to West Siberian hunter-gatherers, Tutkaul 1 does not carry an extra eastern Eurasian ancestry, but shows affinity to Iranian Neolithic farmers and some younger populations from Iran and the Turan region (Supplementary Data 2.L). Conversely, individuals in the Sidelkino cluster are genetically closer to AG3 than Tutkaul 1. This suggests that the newly reported Neolithic individual from central Asia carries an ancestry that might be a good proxy for the ANE-related contribution to Iran and the Turan region^45^ from around 5.5 ka but not to roughly contemporaneous hunter-gatherers from eastern Europe.

In sum, we describe the formation and interaction between the Oberkassel and Sidelkino clusters, the two main hunter-gatherer ancestries present in Europe from 14 ka onwards. The genomic similarity of the Oberkassel cluster to Arene Candide 16 in northwestern Italy might imply that Epigravettian-associated ancestry spread from the south to central Europe passing through the western side of the Alpine region. The Sidelkino ancestry also emerged around 14 ka with its first direct evidence in eastern Europe^43^ dated to 13 ka. The increasing level of admixture between distinct hunter-gatherer populations from around 8 ka onwards indicates an intensified mobility of those forager groups. This might have been in part triggered by the concomitant expansion of Neolithic farmers across Europe and/or by environmental factors, such as the climatic event around 8.2 ka, the largest abrupt cooling in the northern hemisphere during the Holocene epoch^46,47^.

## Phenotypically relevant variants

Leveraging the substantially increased sample size, we investigated genetically distinct hunter-gatherer groups for allele frequencies at selected loci that are known to be associated with specific phenotypic traits in present-day Europeans (Fig. 5b and Supplementary Figs. 27 and 28). Consistent with previous findings, none of the analysed groups show the derived allele at SNP rs4988235 on the *LCT* gene, which is responsible for lactase persistence. As previously hinted^37^, we find a large frequency variation in alleles related to skin and eye pigmentation among post-LGM hunter-gatherer groups. For the SNP associated with light eye colour (*HERC2/OCA2* (rs12913832)), individuals from the Villabruna cluster, Oberkassel cluster, Baltic HG and SHG groups show high frequencies of the derived allele (>90%), which is responsible for the green or blue eye phenotype, whereas Sidelkino cluster, Ukraine HG and Iron Gates HG groups show low occurrence of this allele (10–25%). Instead, for the two SNPs associated with skin colour (*SLC24A5* (rs1426654) and *SLC45A2* (rs16891982)), Sidelkino cluster and Ukraine HG groups show a higher frequency (>90% for *SLC24A5* and 29–61% for *SLC45A2*) of the derived alleles related to light skin colour, compared with Oberkassel and Villabruna clusters, where those alleles are almost completely absent (<1%). On the basis of the genetic variation of present-day Europeans, this could imply phenotypic differences between post-14 ka hunter-gatherer populations across Europe, with individuals in the Oberkassel cluster possibly exhibiting darker skin and lighter eyes, and individuals in the Sidelkino cluster possibly lighter skin and darker eye colour.

## Discussion and conclusions

**Extended Data Fig. 9:**
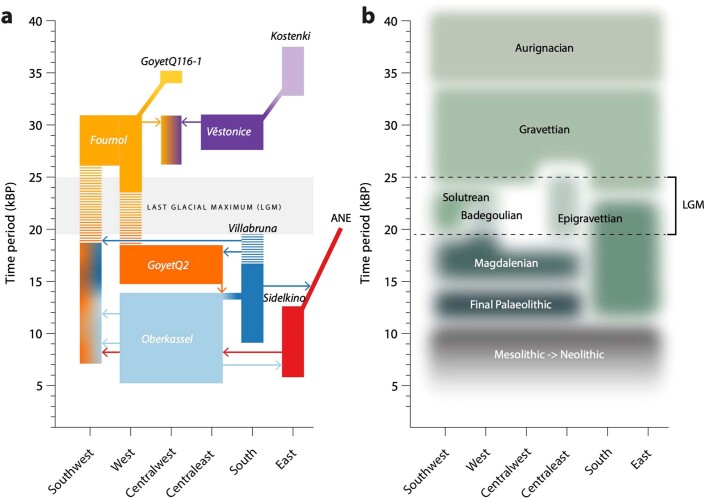
Graphical summary depicting the main genetic transformations in post-40 ka hunter-gatherers from Europe. This figure shows (**a**) the distribution of and interaction between hunter-gatherer genetic ancestries and (**b**) a simplified schematic representation of major chrono-cultural subdivisions of the European Upper Palaeolithic (green blocks) followed by a grouped Mesolithic to Neolithic block (in gray). The x-axes report the geographic regions as divided in Fig. 1a, and the y-axes report time in thousand years before present (kBP). In panel **a**, genetic affinity between different ancestries is indicated by thick lines or shades, while admixture is indicated with arrows. In panel **b**, the colour code does not imply archaeological similarities.

The data generated in this study enabled us to investigate genomic transformations of and interactions between Eurasian hunter-gatherers at high resolution (Extended Data Fig. 9). We provide five novel insights into the genomic history of hunter-gatherer populations over a time span of 30,000 years from the Upper Palaeolithic to the Neolithic.

First, we show that individuals associated with the Gravettian culture across Europe were not a biologically homogeneous population. Culturally, however, we see both widespread general tendencies, such as weaponry and some portable art^48^, and other aspects that have a more regional character, such as mortuary practices (Supplementary Information, section 13), various originalities in lithic and hard organic materials tool kits and adornments^20,21^. The ancestry found in individuals associated with the preceding Aurignacian culture from central Europe (GoyetQ116-1 ancestry) gave rise to Gravettian-associated individuals from western and southwestern Europe. This derived ancestry—the Fournol cluster—survived during the LGM in Solutrean-associated individuals, possibly within the Franco-Cantabrian climatic refugium^25^, leading to later populations associated with the Magdalenian culture (GoyetQ2 cluster and El Mirón). Conversely, the ancestry found in pre-30 ka eastern European individuals (Kostenki cluster and Sunghir group) contributed to Gravettian-associated individuals from central and southern Europe (Věstonice cluster), the latter without descendants retrieved in post-LGM populations from those regions.

Second, the ancestry of individuals associated with the Epigravettian culture (Villabruna cluster), which was found to genetically connect European and Near Eastern hunter-gatherers, reached southern Europe well before the transition between the Early and Late Epigravettian^4,15^ and possibly as early as the Gravettian–Epigravettian transition. A phylogeographic reconstruction of different lineages carrying this ancestry further suggests its entry point into northeastern Italy from the Balkans followed by a north-to-south expansion into the Italian peninsula alongside a population decline through sequential bottlenecks.

Third, Magdalenian-associated individuals not only from Iberia but also from the rest of Europe carry Epigravettian-associated ancestry (Villabruna cluster). Genetic analyses of western European individuals associated with the preceding Badegoulian culture might provide clues on the processes that led to the formation of the GoyetQ2 cluster. As inferred from the archaeological record^35^, the spread of the Magdalenian across Europe is linked to southwestern to northern and northeastern post-LGM population expansions and not to movements from southeastern refugia^34^.

Fourth, we extend the finding of a large-scale genetic turnover as early as 14 ka in central and western European hunter-gatherers associated with multiple techno-complexes—Federmesser, Azilian and other Final Palaeolithic groups^4^—despite considerable technological continuity with the preceding late Magdalenian. This broadly distributed ancestry (the Oberkassel cluster (also known as WHG)) is most closely related to an Epigravettian-associated individual from northwestern Italy, suggesting that its expansion into continental Europe might have started from the west—and not the east—side of the Alps. Moreover, the almost complete genetic replacement of the Magdalenian-associated gene pool raises the hypothesis that parts of Europe were differentially populated during the abrupt climatic variation starting around 14.7 ka with the Bølling–Allerød warming period, creating areas where southern European populations could expand. This might also explain the genetic uniformity of the Oberkassel cluster across large parts of western Eurasia but genomic data from between 15 and 14 ka is needed to understand the exact dynamics of this turnover.

Fifth, the Oberkassel ancestry in western and central Europe and the Sidelkino ancestry in eastern Europe remained largely isolated for almost 6,000 years until genetic interactions were first observed—around 8 ka in northeastern Germany, possibly associated with cultural exchanges along the Baltics^49^ and around 7.5 ka in the upper Volga region, possibly linked to the spread of pottery in the region^50^.

In conclusion, our study reveals that western and southwestern Europe served as climatic refugia for the persistence of human groups during the coldest phase of the last Ice Age whereas populations in the Italian peninsula and the eastern European plain were genetically overturned, challenging the role of these regions as glacial refugia for humans. The incoming Villabruna ancestry later became the most widespread hunter-gatherer ancestry across Europe. Further palaeogenomic studies on Upper Palaeolithic individuals from the Balkans will be essential for understanding whether southeastern Europe represents the source of the Villabruna ancestry and a climatic refugium for human populations during the LGM.

*Note added in proof:* A companion paper^51^ describes genome-wide data of a 23,000-year-old Solutrean-associated individual from southern Iberia that extend the evidence of genetic continuity across the LGM in southwestern Europe.

## Methods

### Archaeological sampling

The ancient human specimens analysed in this work derive from multiple scientific collaborations. All remains were sampled with the approval of the institutions responsible for the analysis of archaeological material. This was achieved through collaboration with local curators and scientists from the countries where the skeletal material is preserved and who are listed among the authors of this study. The responsible co-authors for the material from each archaeological site are listed in Supplementary Information, section 1.

The analysed individuals span from the Upper Palaeolithic to the Neolithic. While terms such as lithic industry, techno-complex, prehistoric tradition, and so on might be more appropriate to refer to the various associated chrono-cultural subdivisions, they concern different levels of discussion and are not applicable to all contexts investigated here. Therefore, the broader terms ‘archaeological culture’ or simply ‘culture’ are used here to refer to archaeologically defined material cultures without implying links to modern anthropological and/or ethnographical concepts of culture.

### Radiocarbon dating

We report 47 new radiocarbon dates performed on skeletal elements of 40 individuals by the Curt-Engelhorn-Zentrum Archaeometrie in Mannheim (MAMS, *n* = 29), Center for Isotope Research, University of Groningen (GrA and GrM, *n* = 5), University of Aarhus (AAR, *n* = 3), Beta Analytics (Beta, *n* = 2), Zürich (ETH, *n* = 3), International Chemical Analysis (ICA, *n* = 2), Natural History Museum in Paris (Echo Lab, *n* = 1) and Vilnius (FTMC, *n* = 2) (Supplementary Data 1.A). The dates were calibrated using OxCal 4.4^52^ with calibration curve IntCal20 at 95.4% probability^53^ and when multiple dates were available for the same individual we used the function R_Combine to combine them^52^. We did not correct the calibrated dates for marine or freshwater reservoir effects but, when available, we report individual stable isotope values (δ^15^N/δ^13^C and C:N ratio) in Supplementary Data 1.A to evaluate the potential impact of such reservoir effects.

### Ancient DNA processing

The human remains were processed in dedicated laboratories at the Max Planck Institute for the Science of Human History in Jena (Germany), University of Tübingen (Germany), University of Florence (Italy), Leiden University Medical Center (the Netherlands) and University of Tartu (Estonia). Human bones and teeth were sampled in clean room facilities to minimize the inclusion of modern human DNA contamination during this procedure. DNA was extracted from the generated bone or tooth powder following established protocols. A subset of samples (GER002 and GER003) were pre-treated with a washing step to reduce surface contamination^54^. A negative and cave bear positive controls were included. For the DNA lysis, a solution of 900 μl EDTA, 75 μl H_2_O and 25 μl proteinase K was added. In a rotator, samples were digested for at least 16 h at 37 °C, and for pre-treated samples this was followed^55^ by an additional hour at 56 °C. The suspension was then centrifuged and transferred into a binding buffer as previously described^56^. To bind DNA, silica columns for high volumes (High Pure Viral Nucleic Acid Large Volume Kit (Roche)) were used. After 2 washing steps using the manufacturer’s wash buffer, DNA was eluted in TET (10 mM Tris, 1 mM EDTA and 0.05% Tween) in two steps for a final volume of 100 μl. After DNA lysis, a subset of samples was extracted using silica-coated magnetic particles on an automated liquid handling system (Agilent Technologies Bravo NGS Workstation)^57^. Double-stranded DNA libraries were built from 25 μl of DNA extract, without the presence of uracil DNA glycosylase (ds_nonUDG) or in the presence of uracil DNA glycosylase (ds_halfUDG), following a double-stranded ‘UDG-half’ library preparation to reduce, but not eliminate, the amount of deamination-induced damage towards the ends of ancient DNA (aDNA) fragments^58^. Negative and positive controls were carried alongside each experiment. Libraries were quantified using the IS7 and IS8 primers^59^ in a quantification assay using a DyNAmo SYBR Green qPCR Kit (Thermo Fisher Scientific) on the LightCycler 480 (Roche). Each aDNA library was double indexed^60^ in 1–4 parallel 100 μl reactions using PfuTurbo DNA Polymerase (Agilent). The indexed products for each library were pooled, purified over MinElute columns (Qiagen), eluted in 50 μl TET and again quantified using the IS5 and IS6 primers^59^ using the quantification method described above. The purified products were amplified in multiple 100 μl reactions using Herculase II Fusion DNA Polymerase (Agilent) following the manufacturer’s specifications with 0.3 μM of the IS5/IS6 primers. After another MinElute purification, the product was quantified using the Agilent 2100 Bioanalyzer DNA 1000 chip. An equimolar pool of all libraries was then prepared for shotgun sequencing on Illumina Hiseq4000 platform using 75bp single-end reads for screening. Single-stranded DNA libraries were built from 30 μl of DNA extract in the absence of uracil DNA glycosylase (ss_nonUDG) followed by double indexing, using an automated version of the protocols described in^61^ on the liquid handling system mentioned before. The single-stranded library of Cuiry Les Chaudardes 1 was produced with partial UDG treatment (ss_halfUDG)^62^ (Supplementary Data 1.B).

### DNA enrichment and sequencing

Both double-stranded and single-stranded libraries were further amplified with IS5/IS6 primers to reach a concentration of 200–400 ng/μl as measured on a NanoDrop spectrophotometer (Thermo Fisher Scientific). The libraries underwent shallow shotgun sequencing on an Illumina HiSeq 4000 instrument with 75 single-end-run cycles using the manufacturer’s protocol, to evaluate the human endogenous DNA content and quality. Samples with a percentage of human DNA in shotgun data around 0.1% or greater were enriched for a set of 1,237,207 targeted SNPs (1240k capture) across the human genome^6^. mtDNA capture^63^ was also performed for those libraries where mtDNA coverage was not high enough to assess mtDNA haplogroup and contamination. Illumina sequencing platforms were also used to sequence the 1240k and mtDNA captured libraries (Supplementary Data 1.B).

The de-multiplexed capture sequencing reads were cleaned and mapped to human reference genome hs37d5 using EAGER pipeline 1.92.55^64^. Within the pipeline, the adapters were removed by AdapterRemoval 2.2.0^65^, reads were mapped with BWA 0.7.12 aln/samse algorithm^66^, duplications were removed by DeDup 0.12.1 (https://github.com/apeltzer/DeDup) and damage patterns of each library were checked with mapDamage 2.0.6 and 2.0.9^67^. The deduplicated bam files were filtered using PMDtools 0.60^68^ with a threshold of 3, to reduce potential modern DNA contamination based on postmortem DNA deamination. For ds_halfUDG libraries, we masked 2 bp from both ends of the reads with trimBam in bamUtil 1.0.13 (https://github.com/statgen/bamUtil) to remove the damaged sites.

The mitochondrial capture sequencing reads were cleaned by AdapterRemoval 2.2.0 to remove the adapters and reads with lengths below 30 bp. Then the cleaned reads together with cleaned reads from 1240k capture sequencing were mapped to human reference mitochondrial sequence NC_012920.1 with BWA 0.7.12 aln/samse algorithm (parameters –n 0.01, –l 16500) and realigned with CircularMapper^64^. The mapped reads from the same individual and library set-up were merged and duplications were removed with DeDup. Reads with a mapping quality below 30 were then filtered with samtools, and the consensus sequences were generated by Schmutzi^69^.

### Ancient DNA authentication and genotyping

The sex of each individual was determined by the ratio of sequencing coverages on sex chromosomes versus autosomes (Supplementary Data 1.C). Individuals with libraries showing signs of contamination were further tested using PMD-filtered bam files. Individuals with at least one library showing Y/Auto ratio > 0.2 were determined as male individuals, and with Y/Auto < 0.2 were determined as female individuals^4^ (Supplementary Fig. 12).

The nuclear DNA contamination was estimated with several methods. We applied ANGSD 0.934^70^ and hapCon^71^ for libraries from male individuals, and applied contamLD^72^ and a newly developed method that analyses contamination in ROH for female and male libraries (see Supplementary Information, section 2 for a detailed description). The mtDNA contamination was estimated by Schmutzi (--notusepredC --uselength)^69^ for all the libraries. Libraries showing a mitochondrial or nuclear contamination rate over 10% were considered substantially contaminated whereas those between 5 and 10% were considered marginally contaminated and were treated differently (details are provided in Supplementary Information, section 3).

The cleaned reads with base quality and mapping quality over 30 were piled up with mpileup in SAMtools 1.3^73^ on the 1240k targeted sites. For contaminated libraries we used the PMD-filtered bam files as the input for genotyping. Then pseudo-haploid genotypes were called using pileupCaller 1.4.0.2 (https://github.com/stschiff/sequenceTools) under random haploid calling mode. For ds_halfUDG libraries, we called genotypes on all targeted sites from 2bp-masked bam files; for ds_nonUDG libraries, we called genotypes on transversion sites only; for ss_nonUDG libraries, we called genotypes with single-strand mode, which ignores forward reads at C/T polymorphisms and reverse reads at G/A polymorphisms.

Then we merged the genotypes from different libraries of the same individual, by randomly picking alleles from available genotype calls, using a custom script. After merging, individuals with less than 6,000 SNPs on 1240k sites were excluded from further analysis because of low coverage. We also genotyped a selection of previously published individuals with the same approach (Supplementary Data 1.G)^2,4,30,74–78^. Then we combined our newly generated genotypes with published genotypes from ancient and modern individuals from AADR v42.4 (Allen Ancient DNA Resource (https://reich.hms.harvard.edu/allen-ancient-dna-resource-aadr-downloadable-genotypes-present-day-and-ancient-dna-data) version 42.4) for downstream analysis^1,4,7,14,16,23,36–40,42,43,45,79–94^.

For individual heterozygosity calculation, we also called pseudo-diploid genotypes from each library, using pileupCaller 1.4.0.3 under random diploid calling mode and the same strategy for different types of libraries as pseudo-haploid genotype calling.

### Uniparental markers

The mitochondrial haplogroups were determined using HaploGrep 2^95^, based on the consensus sequences generated from Schmutzi inspected for each sample at increasing quality filters (from q0 to q20). Inconsistent haplogroup assignments were manually verified as indicated^24^ (Supplementary Data 1.L). For phylogenetic reconstruction (Extended Data Fig. 1) we used MUSCLE (-maxiters 2)^96^ to create a multiple genome alignment of previously published sequences and newly reported mtDNA consensus sequences with q20 according to defined thresholds (minimum average coverage >5-fold, contamination estimate <20%, HaploGrep 2 haplogroup assignment consistent with manual assignment). We built a Maximum Parsimony tree with 103 mtDNA sequences plus an African sequence as the outgroup (not shown) after the removal of individuals younger than 6.5 ka and mtDNAs from the same site with an identical placement. The tree was calculated on 16,528 positions (partial deletion 95%) and with 500 bootstrap iterations using MEGA10^97^.

To determine the Y-chromosome haplogroups of male individuals, we genotyped the Y-chromosome reads using a Y-SNP list (v.15.73) from the International Society of Genetic Genealogy (ISOGG) dataset, ignoring C-to-T and G-to-A transitions on the forward and reverse reads, respectively. This procedure allowed us to manually traverse the ISOGG Y-Haplogroup Tree, checking in a semi-automatic way which positions were covered. This process allowed us to assign an ancestral or derived haplogroup for covered branches, and to make corrections to calls in cases where, for instance, a more derived haplogroup was called because of residual ancient damage (C-to-T or G-to-A mismatches) in terminal read positions at diagnostic SNPs^98^ (Supplementary Data 1.A). For the placement of individuals onto a Y-chromosomal phylogenetic tree (Extended Data Fig. 1), we used pathPhynder^99^ based on the tree from Karmin et al.^100^. We used the default posterior threshold of 0.01, and mapping and sequencing quality cutoffs of 30. We then removed samples with less than 0.04X coverage (calculated on the mappable, non-recombining region of the Y chromosome^98^) to avoid arbitrarily placing low-coverage samples at the root of major haplogroups. This results in a tree with 57 newly reported and previously published ancient individuals while present-day sequences are collapsed in the major Y-chromosome haplogroups (the most basal lineages are not shown). The tentative placements of low-coverage ancient individuals based on their haplogroup assignment (Supplementary Data 1.A) are indicated with arrows on the respective branches.

### Biological relatedness and population diversity

The analysis of biological relatedness was performed by calculating relatedness coefficient (*r*) based on PMR on the autosomal SNPs (Supplementary Data 1.F and Supplementary Information, section 4). The baseline of each population was determined using the average heterozygosity rate of individuals estimated from pseudo-diploid genotypes (Supplementary Data 1.E).

The ROH segments in hunter-gatherer genomes were identified using hapROH^101^. As recommended, we analysed individuals with over 400,000 SNPs called on the 1240k panel^101^ and we called ROH longer than 4 cM (Supplementary Data 3.B). The effective population sizes (*N*_e_) were then estimated using a maximum likelihood method, after filtering individuals with a signal of close-kin inbreeding (individuals with at least 50 cM of their genome in ROH spanning >20 cM) (Supplementary Information, section 9).

### Population genetics analysis

The PCA was carried out by smartpca in EIGENSOFT 6.0.1^102^, with modern individuals used for calculation and all the ancient individuals projected on the calculated PCs. The “lsqproject: YES” parameter was used to minimize the effect of missing data in ancient individuals. The PCA was calculated with 1379 individuals from 87 western Eurasian modern populations on the 1240k_HO dataset, which was intersected between the 1240k and Human Origins datasets (Supplementary Data 1.K).

The MDS analysis showing the genetic affinity among European hunter-gatherers was based on the distance matrix derived from outgroup *f*_3_-statistics, in the form 1 − *f*_3_(Mbuti.DG; pop1, pop2) and performed with classical MDS algorithm (cmdscale) implemented in R 3.5.1. The hunter-gatherers were grouped based on their geographic origins and dates (Supplementary Information, section 5). The *f*_3_-statistics were calculated with qp3Pop 435 in ADMIXTOOLS 5.1 package^103^.

The pairwise *f*_2_ distance matrix of Epigravettian-associated groups was generated with qpfstats 200 in ADMIXTOOLS 7.0.2 package, with parameters “allsnps: YES, scale: NO”, and Mbuti.DG set as the outgroup. The neighbour-joining tree was then reconstructed using the neighbour-joining method implemented in Ape 5.3 package^104^ of R 3.5.1.

The *f*_4_-statistics were calculated by qpDstat 755 with parameter “f4 mode: YES”, with the Mbuti.DG population from Africa used as outgroup in all *f*-statistics analyses. The tool qpAdm 810 in ADMIXTOOLS 5.1 was applied to model the ancestries of admixed populations, with “allsnps” mode and the outgroup set selection described in Supplementary Information, section 11. Admixture graphs were reconstructed using qpGraph 6450, with allsnps mode to correct for low-coverage sample and Mbuti.DG set as the outgroup. Admixture events were dated using the ancestry covariance pattern-based DATES 753 program^105^, with a bin size of 0.1 cM for covariance calculation and the start of exponential fitting at *d* ≥ 0.5cM.

### Phenotypic SNP analysis

As the coverage for most ancient samples was not sufficient for diploid genotype calling, we counted the reads covering selected phenotypic SNPs on reference or alternative alleles and computed the group-based allele frequencies following a maximum likelihood approach described in Mathieson et al.^37^. Details on individuals involved in the analysis, read counts processing and allele frequency computation are provided in the Supplementary Information, section 12 and Supplementary Data 1.J and 3.G.

### Reporting summary

Further information on research design is available in the Nature Portfolio Reporting Summary linked to this article.

## Online content

Any methods, additional references, Nature Portfolio reporting summaries, source data, extended data, supplementary information, acknowledgements, peer review information; details of author contributions and competing interests; and statements of data and code availability are available at 10.1038/s41586-023-05726-0.

### Supplementary information

Supplementary Information Archaeological background, method descriptions and discussions additional to the main text, including sections 1–13, Figs. 1–32, Tables 1–4 and references.

Reporting Summary

Supplementary Data 1 Metadata information of studied individuals.

Supplementary Data 2 Raw data of *f*-statistics analyses.

Supplementary Data 3 Raw data of other types of analyses.

## Figures and Tables

**Extended Data Table 1:**
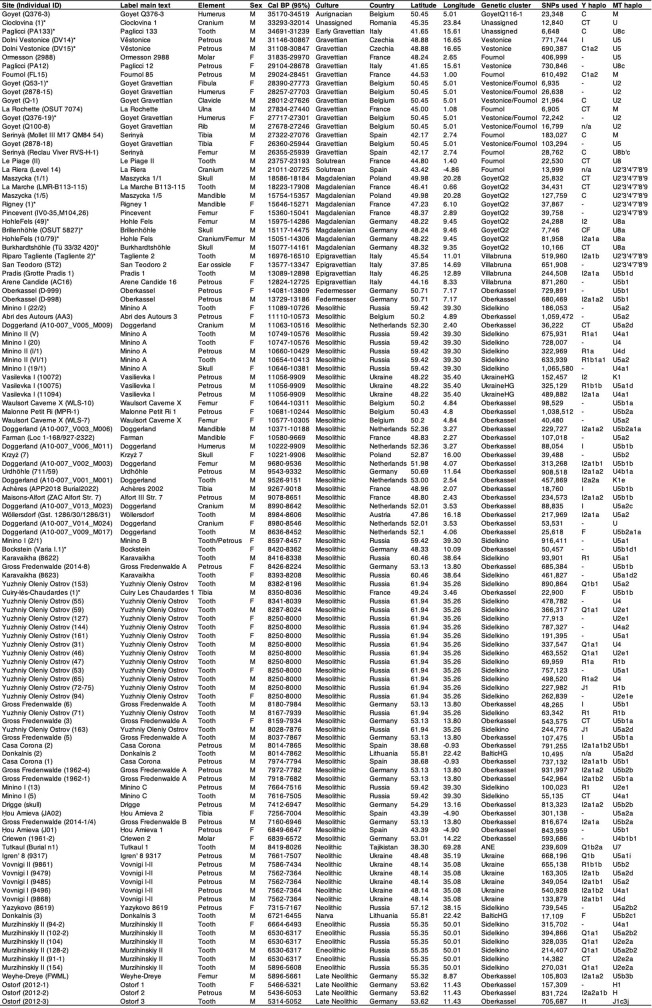
Summary statistics for individuals with newly reported genomic data

Previously published individuals with additional data generated in this study are marked with an asterisk in the first column. The table is sorted based on cultural association maintaining an in-group temporal order from the oldest to the youngest individual.

## Data Availability

The aligned sequences of all individuals with new genomic data reported in this study are available at the European Nucleotide Archive (ENA) under study accession number PRJEB51862. The compiled genotype file used for analyses, including re-genotyped published genomes, has been uploaded at the Edmond Data Repository of the Max Planck Society (https://edmond.mpdl.mpg.de/dataset.xhtml?persistentId=doi:10.17617/3.Y1KJMF).

## Code availability

The code for the newly developed ROH based contamination estimate method is available at https://github.com/hyl317/hapROH. A user manual including an installation guide is available at https://haproh.readthedocs.io/en/latest/hapROH_with_contamination.html. The version used for this work is archived at https://edmond.mpdl.mpg.de/dataset.xhtml?persistentId=doi:10.17617/3.Y1KJMF.
