# Author Correction: Palaeogenomics of Upper Palaeolithic to Neolithic European hunter-gatherers

**DOI:** 10.1038/s41586-023-05942-8

**Published:** 2023-03-22

**Authors:** Cosimo Posth, He Yu, Ayshin Ghalichi, Hélène Rougier, Isabelle Crevecoeur, Yilei Huang, Harald Ringbauer, Adam B. Rohrlach, Kathrin Nägele, Vanessa Villalba-Mouco, Rita Radzeviciute, Tiago Ferraz, Alexander Stoessel, Rezeda Tukhbatova, Dorothée G. Drucker, Martina Lari, Alessandra Modi, Stefania Vai, Tina Saupe, Christiana L. Scheib, Giulio Catalano, Luca Pagani, Sahra Talamo, Helen Fewlass, Laurent Klaric, André Morala, Mathieu Rué, Stéphane Madelaine, Laurent Crépin, Jean-Baptiste Caverne, Emmy Bocaege, Stefano Ricci, Francesco Boschin, Priscilla Bayle, Bruno Maureille, Foni Le Brun-Ricalens, Jean-Guillaume Bordes, Gregorio Oxilia, Eugenio Bortolini, Olivier Bignon-Lau, Grégory Debout, Michel Orliac, Antoine Zazzo, Vitale Sparacello, Elisabetta Starnini, Luca Sineo, Johannes van der Plicht, Laure Pecqueur, Gildas Merceron, Géraldine Garcia, Jean-Michel Leuvrey, Coralie Bay Garcia, Asier Gómez-Olivencia, Marta Połtowicz-Bobak, Dariusz Bobak, Mona Le Luyer, Paul Storm, Claudia Hoffmann, Jacek Kabaciński, Tatiana Filimonova, Svetlana Shnaider, Natalia Berezina, Borja González-Rabanal, Manuel R. González Morales, Ana B. Marín-Arroyo, Belén López, Carmen Alonso-Llamazares, Annamaria Ronchitelli, Caroline Polet, Ivan Jadin, Nicolas Cauwe, Joaquim Soler, Neus Coromina, Isaac Rufí, Richard Cottiaux, Geoffrey Clark, Lawrence G. Straus, Marie-Anne Julien, Silvia Renhart, Dorothea Talaa, Stefano Benazzi, Matteo Romandini, Luc Amkreutz, Hervé Bocherens, Christoph Wißing, Sébastien Villotte, Javier Fernández-López de Pablo, Magdalena Gómez-Puche, Marco Aurelio Esquembre-Bebia, Pierre Bodu, Liesbeth Smits, Bénédicte Souffi, Rimantas Jankauskas, Justina Kozakaitė, Christophe Cupillard, Hartmut Benthien, Kurt Wehrberger, Ralf W. Schmitz, Susanne C. Feine, Tim Schüler, Corinne Thevenet, Dan Grigorescu, Friedrich Lüth, Andreas Kotula, Henny Piezonka, Franz Schopper, Jiří Svoboda, Sandra Sázelová, Andrey Chizhevsky, Aleksandr Khokhlov, Nicholas J. Conard, Frédérique Valentin, Katerina Harvati, Patrick Semal, Bettina Jungklaus, Alexander Suvorov, Rick Schulting, Vyacheslav Moiseyev, Kristiina Mannermaa, Alexandra Buzhilova, Thomas Terberger, David Caramelli, Eveline Altena, Wolfgang Haak, Johannes Krause

**Affiliations:** 1 Archaeo- and Palaeogenetics, Institute for Archaeological Sciences, Department of Geosciences, University of Tübingen, Tübingen, Germany; 2 Senckenberg Centre for Human Evolution and Palaeoenvironment at the University of Tübingen, Tübingen, Germany; 3 Department of Archaeogenetics, Max Planck Institute for Evolutionary Anthropology, Leipzig, Germany; 4 State Key Laboratory of Protein and Plant Gene Research, School of Life Sciences, Peking University, Beijing, China; 5 Department of Anthropology, California State University Northridge, Northridge, CA USA; 6 Université de Bordeaux, CNRS, MC, PACEA UMR 5199, Pessac, France; 7 School of Mathematical Sciences, University of Adelaide, Adelaide, South Australia Australia; 8 Instituto Universitario de Investigación en Ciencias Ambientales de Aragón, IUCA-Aragosaurus, Zaragoza, Spain; 9 Department of Archaeogenetics, Max Planck Institute for the Science of Human History, Jena, Germany; 10 Institute of Zoology and Evolutionary Research, University of Jena, Jena, Germany; 11 Center of Excellence ‘Archaeometry’, Kazan Federal University, Kazan, Russia; 12 Department of Biology, University of Florence, Florence, Italy; 13 Estonian Biocentre, Institute of Genomics, University of Tartu, Tartu, Estonia; 14 St John’s College, University of Cambridge, Cambridge, UK; 15 Department of Biological, Chemical and Pharmaceutical Sciences and Technologies, University of Palermo, Palermo, Italy; 16 Department of Biology, University of Padova, Padova, Italy; 17 Department of Chemistry G. Ciamician, Alma Mater Studiorum, University of Bologna, Bologna, Italy; 18 Department of Human Evolution, Max Planck Institute for Evolutionary Anthropology, Leipzig, Germany; 19 UMR 8068 CNRS, TEMPS—Technologie et Ethnologie des Mondes Préhistoriques, Nanterre Cedex, France; 20 Musée National de Préhistoire, Les Eyzies de Tayac, France; 21 Paléotime, Villard-de-Lans, France; 22 UMR 5140 CNRS, Archéologie des Sociétés Méditerranéennes, Université Paul-Valéry, Montpellier, France; 23 UMR 7194, Histoire Naturelle de l’Homme Préhistorique (HNHP), Département Homme et Environnement, Muséum National d’Histoire Naturelle, CNRS, UPVD, Paris, France; 24 Association APRAGE (Approches pluridisciplinaires de recherche archéologique du Grand-Est), Besançon, France; 25 Inrap GE, Metz, France; 26 Skeletal Biology Research Centre, School of Anthropology and Conservation, University of Kent, Canterbury, UK; 27 Dipartimento di Scienze Fisiche, della Terra e dell’Ambiente, U.R. Preistoria e Antropologia, Università degli Studi di Siena, Siena, Italy; 28 Accademia dei Fisiocritici, Siena, Italy; 29 Centro Studi sul Quaternario ODV, Sansepolcro, Italy; 30 Institut National de Recherches Archéologiques, Bertrange, Luxembourg; 31 Department of Cultural Heritage, University of Bologna, Ravenna, Italy; 32 Human Ecology and Archaeology (HUMANE), Department of Archaeology and Anthropology, Institució Milà i Fontanals de Investigación en Humanidades, Consejo Superior de Investigaciones Científicas (IMF - CSIC), Barcelona, Spain; 33 UMR 7209—Archéozoologie et Archéobotanique-Sociétés, Pratiques et Environnements, Muséum National d’Histoire Naturelle, Paris, France; 34 Dipartimento di Scienze Della Vita e Dell’Ambiente, Sezione di Neuroscienze e Antropologia, Università Degli Studi di Cagliari, Cittadella Monserrato, Cagliari, Italy; 35 Dipartimento di Civiltà e Forme Del Sapere, Università di Pisa, Pisa, Italy; 36 Center for Isotope Research, Groningen University, Groningen, The Netherlands; 37 Inrap CIF, Croissy-Beaubourg, France; 38 UMR 7206 Éco-Anthropologie, Équipe ABBA. CNRS, MNHN, Université de Paris Cité, Musée de l’Homme, Paris, France; 39 PALEVOPRIM Lab UMR 7262 CNRS-INEE, University of Poitiers, Poitiers, France; 40 Centre de Valorisation des Collections Scientifiques, Université de Poitiers, Mignaloux Beauvoir, France; 41 Musées de Poitiers–Ville de Poitiers, Poitiers, France; 42 Departamento de Geología, Facultad de Ciencia y Tecnología, Universidad del País Vasco/Euskal Herriko Unibertsitatea (UPV/EHU), Leioa, Spain; 43 Sociedad de Ciencias Aranzadi, Donostia-San Sebastian, Spain; 44 Centro UCM-ISCIII de Investigación sobre Evolución y Comportamiento Humanos, Madrid, Spain; 45 Institute of Archaeology, University of Rzeszów, Rzeszów, Poland; 46 Foundation for Rzeszów Archaeological Centre, Rzeszów, Poland; 47 Center for Genomic Medicine, Massachusetts General Hospital, Boston, MA USA; 48 Department of Psychiatry, Harvard Medical School, Boston, MA USA; 49 Groninger Instituut voor Archeologie, Groningen University, Groningen, The Netherlands; 50 Stralsund Museum, Stralsund, Germany; 51 Institute of Archaeology and Ethnology, Polish Academy of Science, Poznań, Poland; 52 Institute of History, Archaeology and Ethnography, Dushanbe, Tajikistan; 53 ArchaeoZOOlogy in Siberia and Central Asia—ZooSCAn, CNRS–IAET SB RAS International Research Laboratory, IRL 2013, Institute of Archaeology SB RAS, Novosibirsk, Russia; 54 Research Institute and Museum of Anthropology, Moscow State University, Moscow, Russia; 55 Grupo de I+D+i EVOADAPTA (Evolución Humana y Adaptaciones durante la Prehistoria) Departamento de Ciencias Históricas, Universidad de Cantabria, Santander, Spain; 56 Instituto Internacional de Investigaciones Prehistóricas de Cantabria (IIIPC), Universidad de Cantabria-Gobierno de Cantabria-Banco Santander, Santander, Spain; 57 Departamento de Biología de Organismos y Sistemas, Universidad de Oviedo, Oviedo, Spain; 58 Quaternary Environments and Humans, OD Earth and History of Life, Royal Belgian Institute of Natural Sciences, Brussels, Belgium; 59 Musées Royaux d’Art et d’Histoire, Bruxelles, Belgium; 60 Institute of Historical Research, University of Girona, Catalonia, Spain; 61 INRAP/UMR 8215 Trajectoires 21, Paris, France; 62 School of Human Evolution and Social Change, Arizona State University, Tempe, AZ USA; 63 Department of Anthropology, University of New Mexico, Albuquerque, NM USA; 64 GéoArchPal-GéoArchÉon, Viéville sous-les-Cotes, France; 65 Archäologie & Münzkabinett, Universalmuseum Joanneum, Graz, Austria; 66 Museum ‘Das Dorf des Welan’, Wöllersdorf-Steinabrückl, Austria; 67 Pradis Cave Museum, Clauzetto, Italy; 68 Department of Humanities, University of Ferrara, Ferrara, Italy; 69 National Museum of Antiquities, Leiden, The Netherlands; 70 Faculty of Archaeology, Leiden University, Leiden, The Netherlands; 71 Biogeology, Department of Geosciences, University of Tübingen, Tübingen, Germany; 72 Unité de Recherches Art, Archéologie Patrimoine, Université de Liège, Liège, Belgium; 73 I.U. de Investigación en Arqueología y Patrimonio Histórico, University of Alicante, Sant Vicent del Raspeig, Alicante, Spain; 74 Arpa Patrimonio S. L., Alicante, Spain; 75 Amsterdam Centre of Ancient Studies and Archaeology, University of Amsterdam, Amsterdam, The Netherlands; 76 Department of Anatomy, Histology and Anthropology, Faculty of Medicine, Vilnius University, Vilnius, Lithuania; 77 Service Régional de l’Archéologie de Bourgogne-Franche-Comté, Besançon Cedex, France; 78 Laboratoire de Chrono-Environnement, UMR 6249 du CNRS, UFR des Sciences et Techniques, Besançon Cedex, France; 79 Weyhe, Germany; 80 Ulm, Germany; 81 LVR-LandesMuseum Bonn, Bonn, Germany; 82 Institute of Pre- and Protohistory, University of Tübingen, Tübingen, Germany; 83 Department of Archeological Sciences, Thuringian State Office for Monuments Preservation and Archeology, Weimar, Germany; 84 University of Bucharest, Faculty of Geology and Geophysics, Department of Geology, Bucharest, Romania; 85 Institute for Advanced Studies in Levant Culture and Civilization, Bucharest, Romania; 86 German Archaeological Institute, Berlin, Germany; 87 Brandenburg Authorities for Heritage Management and Archaeological State Museum, Zossen, Germany; 88 Institute for Pre- and Protohistory, Kiel University, Kiel, Germany; 89 Institute of Archeology at Brno, Czech Academy of Sciences, Centre for Palaeolithic and Paleoanthropology, Brno, Czechia; 90 Institute of Archaeology, Academy of Sciences of the Republic of Tatarstan, Kazan, Russia; 91 Samara State University of Social Sciences and Education, Samara, Russia; 92 Early Prehistory and Quaternary Ecology, Department of Geosciences, University of Tübingen, Tübingen, Germany; 93 Paleoanthropology, Institute for Archaeological Sciences, Department of Geosciences, University of Tübingen, Tübingen, Germany; 94 DFG Centre for Advanced Studies ‘Words, Bones, Genes, Tools’, University of Tübingen, Tübingen, Germany; 95 Royal Belgian Institute of Natural Sciences, Brussels, Belgium; 96 Anthropologie-Büro, Berlin, Germany; 97 Institute of Archaeology Russian, Academy of Sciences, Moscow, Russia; 98 School of Archaeology, University of Oxford, Oxford, UK; 99 Peter the Great Museum of Anthropology and Ethnography (Kunstkamera), Russian Academy of Sciences, Saint Petersburg, Russia; 100 Department of Cultures, University of Helsinki, Helsinki, Finland; 101 Seminar for Pre- and Protohistory, Göttingen University, Göttingen, Germany; 102 Lower Saxony State Service for Cultural Heritage, Hannover, Germany; 103 Department of Human Genetics, Leiden University Medical Center, Leiden, The Netherlands

**Keywords:** Archaeology, Population genetics, Biological anthropology, Evolutionary genetics

Correction to: *Nature* 10.1038/s41586-023-05726-0 Published online 1 March 2023

In the version of this article initially published, the affiliation listed for David Caramelli was incorrect (Kazan Federal University, Kazan, Russia). The affiliation has been corrected to the Department of Biology, University of Florence, Florence, Italy in the HTML and PDF versions of the article.

