# Supplementary material for: Palaeogenomics of Upper Palaeolithic to Neolithic European hunter-gatherers
Source: Nature. 2023 Mar 1;615(7950):117–26. doi: 10.1038/s41586-023-05726-0 (PMC9977688; doi:10.1038/s41586-023-05726-0)
Supplement: Supplementary file 1 — Archaeological background, method descriptions and discussions additional to the main text, including sections 1–13, Figs. 1–32, Tables 1–4 and references. [file 41586_2023_5726_MOESM1_ESM.pdf]

nature portfolio

https://doi.org/10.1038/s41586-023-05726-0

Supplementary information

Palaeogenomics of Upper Palaeolithic to Neolithic European hunter-gatherers

In the format provided by the authors and unedited

Supplementary Information

Section 1: Archaeological context information 2

Section 2: ROH contamination estimation 58

Section 3: Ancient DNA authenticity 71

Section 4: Uniparental markers and biological relatedness 76

Section 5: Genetic clustering of European hunter-gatherers 79

Section 6: Neanderthal ancestry in European hunter-gatherers 82

Section 7: East Asian affinity in Goyet Q116-1-related populations 86

Section 8: Near Eastern affinity in European hunter-gatherers 87

Section 9: Run of homozygosity (ROH) in European hunter-gatherers 89

Section 10: Reconstructing the admixture graph of major pre-LGM genetic ancestries 91

Section 11: Genetic ancestry modelling of post-LGM hunter-gatherers 100

Section 12: Phenotypic analysis of European hunter-gatherers 104

Section 13: Mortuary practices of genetically analyzed Upper Paleolithic humans 107

Supplementary References 113

Section 1: Archaeological context information

In this section we describe the archeological sites from where the genetically analyzed human remains originate. Details about uncalibrated dates, dating lab IDs and publications are provided in Data S1.A. Next to each analyzed individual are reported radiocarbon dates calibrated in years before present (BP) using OxCal 4.4 and the R_Combine command was used when multiple dates associated with the same individual are available (Bronk Ramsey, 2009) with the calibration curve IntCal20 (Reimer et al., 2020) at 95.4% probability. Non-direct dates are indicated as "(layer)". No correction for marine or freshwater reservoir effects were applied to calibrated dates with the exception of the non-directly dated individuals from Yuzhniy Oleniy Ostrov (Schulting et al., 2022). Moreover, when available, we report individual stable isotope values ($\delta^{15}N$, $\delta^{13}C$ and C:N ratio) to evaluate the potential impact of such reservoir effects (Data S1.A).

References:

Bronk Ramsey, C. Bayesian Analysis of Radiocarbon Dates. *Radiocarbon* **51**, 337–360 (2009).
Reimer, P. J. et al., The IntCal20 Northern Hemisphere radiocarbon age calibration curve (0–55 cal kBP). *Radiocarbon* **62**, 725–757 (2020).
Schulting, R. J. et al., Radiocarbon dating from Yuzhniy Oleniy Ostrov cemetery reveals complex human responses to socio-ecological stress during the 8.2 ka cooling event. *Nat. Ecol. Evol.* **6**, 155–162 (2022).

Abri des Autours, Belgium

Patrick Semal, Caroline Polet, Nicolas Cauwe

Analyzed individual:

- AAT001 (AA3): 11110-10573 calBP

The caves in the Meuse Valley and its tributaries have been used by humans as settlements and/or burial sites since the Middle Paleolithic. Numerous excavations were carried out from the 19th century and many caves were found to contain Neanderthal and modern human remains. The vast majority of the modern human fossils relate to the Middle and Late Neolithic. It was not until 1984 that the first Mesolithic burial was attested, although older finds have sometimes been reported as Mesolithic but without scientific evidence (Toussaint, 2013). Today, there are about ten sites that all relate to the Early Mesolithic (Polet et al., 2020) from recent excavations or from older excavations supported by direct dating of human remains.
An attribution to the Late Mesolithic has been suggested for the site of La Martina (Dewez et al., 1995) but it seems that the results of classical radiocarbon dating led to a wrong interpretation and that different dates carried out afterwards on the material have invalidated this interpretation (Toussaint & Ramon, 1997).
The site of Abri des Autours is located in the Freyr Rocks on the right bank of the Meuse near the town of Dinant, Namur province, Belgium. The site was excavated by the Royal Museums of Art and History in 1992 and 1993 (Cauwe et al., 1994). Three burial structures were identified: 1) the first structure (AA-1) is a collective burial dated to the Middle Neolithic (Cauwe, 1995). It contains 446 human remains for an MNI of 3 adults and 6 juveniles (Polet and Cauwe, 2007); 2) The second structure (AA-2) is a collective burial associated with Mesolithic archaeological material (Cauwe et al., 1994) and dated to the Early Mesolithic: 9090 ± 140 BP (OxA-5838; Cauwe, 1995). It contains

1196 human remains for an MNI of 5 adults, one cremated adult and a minimum of 6 immature individuals (Polet and Cauwe, 2007); 3) The third structure is an individual burial without associated archaeological material. It is stratigraphically earlier than the AA-1 and AA-2 assemblages (Cauwe et al., 1994). It has been directly radiocarbon dated to 9500 ± 75 BP (Cauwe, 1994). The anthropological study shows that it is a female individual aged over 50 years (Polet and Cauwe, 2007). The stable isotopes of the AA3 individual suggest that the main dietary protein must have been provided mainly by terrestrial mammals (Bocherens et al., 2007). The material belongs to the collections of the Royal Museums of Art and History. The human remains of structures AA-1 and AA-2 are deposited in the Archaeological Museum of the Upper Meuse in Godinne. The human remains of structure AA3 are kept at the Royal Belgian Institute of Natural Sciences.

References:
Bocherens, H., Polet, C. & Toussaint, M. Palaeodiet of Mesolithic and Neolithic populations of Meuse Basin (Belgium): Evidence from stable isotopes. *Journal of Archaeological Science* **34**, 10–27 (2007).
Cauwe, N. *et al.,* De l'individuel au collectif : les sépultures de l'abri des Autours à Dinant (Namur). *Notae Praehistoricae* **13–1993**, 101–107 (1994).
Cauwe, N. Il y a près de 11.000 ans, l'histoire d'une Mésolithique. *Notae Praehistoricae* **14–1994**, 91–93 (1994).
Cauwe, N. Chronologie des sépultures de l'abri des Autours à Anseremme-Dinant. *Notae Praehistoricae* **15–1995**, 51–60 (1995).
Dewez, M., Cordy, J.-M. & Groessens-Van Dyck, M.-C. La Grotte de La Martina (Dinant, Belgique) et sa sépulture mésolithique. *C.R. Acad. Sc. Paris* **321**, série IIA, 639–641 (1995).
Polet, C. & Cauwe, N. Étude anthropologique des sépultures préhistoriques de l'abri des Autours (Province de Namur, Belgique). *Anthropologie et Préhistoire* **118**, 87–126 (2007).
Polet, C., Drucker, D. G., Glas, C., Sabaux, C., Goffette, Q., Samsel, M., Jadin, I., Warmenbol, E. & Villotte, S. Waulsort Caverne X: A new cave site with Early Mesolithic human remains in Belgium. *Mesolithic Miscellany* **28(2)**, 25–43 (2020).
Toussaint, M. in *Transitions, ruptures et continuité en Préhistoire, Vol 2 Paléolithique et Mésolithique* (eds. Jaubert, J., Fourment, N. & Depaepe, P.) 183–200 (Société Préhistorique Française, 2013).
Toussaint, M. & Ramon, F. Les ossements humains présumés mésolithiques de la grotte de La Martina, à Dinant, ne seraient-ils pas plutôt néolithiques ? *Notae Praehistoricae* **17-1997**, 157–167 (1997).

Achères, France

Frédérique Valentin, Dorothée Drucker, Bénédicte Souffi, Laure Pecqueur

Analyzed individual:
- ACR001 (APP 2018, Burial 2002): 9267-9018 calBP

The genetically analysed burial (2002) from the Achères "Parc Paysager" site, Yvelines, France, excavated by the *Institut National de Recherches Archéologiques Préventives*, is located about 100 m north of a small hunting halt dated to the early Mesolithic (late 9th millennium BC), and about 50 m north of another burial excavated in 2013 and dated to the late 8th millennium BC (Jaulneau, 2013; Debout et al., 2014). The bones were discovered at the top of a sandy dome between the Seine and a

paleochannel, at a depth of about 1.30 m and under several pebbles and stone fragments, some of which were burnt. They represent a secondary addition, due to the absence of traces of heating on the surrounding sediment or on the bones. The pit was probably circular in shape and had small dimensions, adapted to the size of the body. It contained a single individual of over 30 years of age whose sex could not be estimated by osteological techniques because of the poor preservation of the pelvic bones. The body was placed in a squatting position, with the pelvis facing north-northeast. The pit was immediately filled with sediment after deposition, although the skeleton was compacted under the action of gravity. No grave goods, either worn or deposited, were found with the skeleton.

References:
Debout G., Blin A., Le Jeune Y. (2014) – Du Tardiglaciaire et du Mésolithique à Achères (Yvelines). Une sépulture secondaire de la fin du Boréal, *in* B. Valentin, S. Griselin, et L. Mevel (dir.), *Paléolithique final et Mésolithique dans le Bassin parisien et ses marges. Habitats, sociétés et environnements : rapport de projet collectif de recherche, programmes P7, P8 et P10.* Nanterre, UMR 7041 - Ethnologie préhistorique, p. 197-209.
Jaulneau C. (2013) – *Achères (Yvelines), « Parc paysager, phase 1 » : rapport de diagnostic*, Montigny-le-Bretonneux, SADY, 181 p.

Arene Candide, Italy

David Caramelli, Alessandra Modi, Martina Lari, Vitale Sparacello, Elisabetta Starnini

Analyzed individual:
- AC16 (Arene Candide 16): 12824-12725 calBP

Arene Candide is a large cave system facing the sea, opening 90 m above current sea level on the slopes of Monte Caprazoppa, Finale Ligure, northwestern Italy. Its name (White Sands) derives from a large white sand dune that rose from the sea up to the entrance of the cave, now quarried away. The deposit in eastern portion of the cave yielded one of the most complete stratigraphic sequences in the western Mediterranean, spanning from the Mid-Upper Paleolithic (Gravettian) to the Byzantine era (Bietti and Molari, 1994; Maggi et al., 1997; Arobba et al., 2017). The investigation of the Upper Pleistocene layers began in the 1940s (Cardini, 1941; 1942; 1946; 1980), and led to the discovery of a large funerary area, apparently marked by the deposition of a complete set of moose antlers: the "Epigravettian necropolis". The necropolis, chronologically spanning the duration of the Younger Dryas, ~12,800-11,800 cal BP, consists of a minimum of 22 individuals in primary and secondary deposition which were buried following a complex multi-stage funerary program (Formicola et al., 2005; Sparacello et al., 2018; 2021). Individual AC16, a largely complete skeleton of an adolescent (c. 12-14 years old based on epiphyseal fusion), was found in 1970 against the southern wall of the cave (Zone A), in a primary supine distended deposition (Formicola and Toscani, 2014). In contrast with later depositions of the necropolis, no grave goods were directly associated with the burial, although a fragment of another moose antler was found nearby. Based on anatomical traits, AC16 was assigned a male sex (Formicola and Toscani, 2014), whereas the genetic analysis revealed a female individual. Directly dated to ~12,800 calBP, AC16 is insofar the earliest deposit of the Epigravettian necropolis.

References:

Arobba, D., Panelli, C., Caramiello, R., Gabriele, M., Maggi, R. 2017. Cereal remains, plant impressions and 14C direct dating from the Neolithic pottery of Arene Candide Cave (Finale Ligure, NW Italy). J. Archaeol. Sci. Rep. 12, 395-404.

Bietti, A., Molari, C. 1994. The Upper Pleistocene deposit of the Arene Candide cave (Savona, Italy): general introduction and stratigraphy. Quaternaria Nova 4, 9-27.

Cardini, L. 1941. Ricerche paletnologiche nella Caverna delle Arene Candide. Arch. Antropol. Etnol. 70, 110-119.

Cardini, L. 1942. Nuovi documenti sull'antichità dell'uomo in Italia: reperto umano del Paleolitico superiore della Grotta delle Arene Candide. Raz. Civil. 3, 5-25.

Cardini, L. 1946. Gli strati mesolitici e paleolitici della caverna delle Arene Candide. Riv. St. Lig. 12, 29-37.

Cardini, L. 1980. La necropoli mesolitica delle Arene Candide. Mem. Ist. It. Paleont. Um. 3, 9-31.

Formicola, V., Pettitt, P.B., Maggi, R., Hedges, R. 2005. Tempo and mode of formation of the Late Epigravettian necropolis of Arene Candide cave (Italy): direct radiocarbon evidence. J. Archaeol. Sci. 32, 1598-1602.

Formicola, V., Toscani, F. 2014. A Late Epigravettian skeleton of an adolescent from Arene Candide Cave (Excavations 1970-1971). Bull. Mus. Anthropol. Préhist. Monaco 54, 131-138

Maggi, R., 1997. The radiocarbon chronology. In: Maggi, R. (Ed.), Arene Candide: a Functional and Environmental Assessment of the Holocene Sequence (Excavations Bernabò Brea - Cardini 1940-50). Mem. Ist. It. Paleont. Um. 5, 31–52.

Sparacello, V.S., Rossi, S., Pettitt, P., Roberts, C.A., Riel-Salvatore, J., Formicola, V. 2018. New insights on Final Epigravettian funerary behavior at Arene Candide Cave (Western Liguria, Italy). J. Anthropol. Sci. 96, 161-184.

Sparacello, V.S., Dori, I., Rossi, S., Varalli, A., Riga, A., Riel-Salvatore, J., Miguel-Gravel, C., Seghi, F., Goude, G., Palstra, S.W.L., Starnini, E., Formicola, V., Moggi-Cecchi J. 2021. New human remains from the Late Epigravettian "necropolis" of Arene Candide (Liguria, northwestern Italy): direct radiocarbon evidence and inferences on the funerary use of the cave during the Younger Dryas. Quat. Sci. Rev. 268, 107131.

Bockstein, Germany

Kurt Wehrberger

Analyzed individual:

- Bockstein (Varia I.1): 8420-8362 calBP

Archeological description previously published in Posth et al., 2016 and Fu et al., 2016.

Brillenhöhle, Germany

Katerina Harvati

Analyzed individual:

- Brillenhöhle (OSUT 5827): 15117-14475 calBP

Archeological description previously published in Posth et al., 2016 and Fu et al., 2016.

Burkhardtshöhle, Germany

Katerina Harvati

Analyzed individual:

- Burkhardtshöhle (Tü 33/32 420): 15077-14161 calBP

Archeological description previously published in Posth et al., 2016 and Fu et al., 2016.

Casa Corona, Spain

Javier Fernández-López de Pablo, Magdalena Gómez-Puche, Marco Aurelio Esquembre Bebia

Analyzed individuals:

- CRN001 (UE 183, individual 1): 7974-7794 calBP
- CRN002 (UE 182, individual 2): 8014-7865 calBP

Casa Corona is an open-air site located in the municipality of Villena (Alicante, Spain), in the upper course of the Vinalopó River. This area occupies a wide natural corridor with an average altitude of 500 m a.s.l. dominated by quaternary deposits of aeolian sands, endorheic lagoons, and ponds. The site was settled on the top of a Pleistocene dune, at the northern margin of an endorheic basin. The presence of surface archaeological materials around the location of Casa Corona was first reported in 2006 as a part of a program of archaeological surveys conducted by the Museum of Villena (Pérez-Amorós and Hernández-Alcaraz, 2006). The archaeological excavations, however, were conducted in 2008 by the company Arpa Patrimonio S.L. in the context of a rescue program due to the construction of a new high-speed rail network between Alicante and Madrid. Subsequent fieldwork campaigns were undertaken in 2013, 2014 and 2017 under the direction of JFLdP and MGP, over the eastern adjacent parcel of the 2008 rescue excavations.

Casa Corona is a multicomponent site with a long archaeological sequence. Along the different fieldwork campaigns, the total excavated area covers approximately 1000 m^2, yielding archeological features spanning different occupation phases: from the Early Mesolithic to the Bell Baker Chalcolithic (Fernández-López de Pablo et al., 2011, 2013 and 2014). The specimens genetically analysed in this study come from two individual burials directly radiocarbon dated to the Late Mesolithic, and extensively described in a previous study (Fernández-López de Pablo et al., 2013). For both individuals the bone collagen quality indicators (van Klinken 1999) were good enough to conduct reliable AMS radiocarbon dating and stable isotope palaeodietary analyses.

Sample CRN001 comes from the previously published burial 1, which was described as a primary individual interment of an adult woman aged 35-40 years, deposited into a burial pit in supine position, with the legs flexed and the face towards the left. The previously published female attribution must be revised according to the current aDNA evidence reported in the present study, with the individual assigned to the male genetic sex. Palaeodietary evidence, based on $\delta^{13}C$ and $\delta^{15}N$

stable isotope analysis, indicates a fully terrestrial diet without any clear signal of either marine or freshwater protein input.
Sample CRN002 corresponds to the burial 2, a primary individual interment of a child aged 1-1.5 years deposited in supine position into a burial pit. Stable isotope $\delta^{13}C$ and $\delta^{15}N$ palaeodietary evidence indicates higher stable isotope $d^{15}N$ values than burial 1, which can be explained by breastfeeding of the individual in burial 2.
The radiocarbon dating results from burial 1 (Beta-7070±40 BP) and burial 2 (OxA-V-2392-92, 7070±40 BP) are statistically similar (X^2=0.8, df=1, p <0.05) both corresponding to ~7,900 calBP and suggesting a short-term funerary activity in this site. For the central sector of the Iberian Mediterranean coast (covering the current provinces of Alicante and Valencia), the burials of Casa Corona represent the most recently dated human remains of Mesolithic populations, occupying a terminal position into the Late Mesolithic of trapezes, just two centuries before the appearance of the first farming communities.

References:
Fernández-López de Pablo, J., Gómez-Puche, M., Martínez-Ortí, A. (2011) Systematic consumption of non-marine gastropods at open-air Mesolithic sites in the Iberian Mediterranean region. Quat. Int. 244, 45–53. https://doi.org/https://doi.org/10.1016/j.quaint.2011.05.031
Fernández-López de Pablo, J., Salazar-García, D.C., Subirà-Galdacano, M.E., Roca de Togores, C., Gómez-Puche, M., Richards, M.P., Esquembre-Bebiá, M.A. (2013) Late Mesolithic burials at Casa Corona (Villena, Spain): direct radiocarbon and palaeodietary evidence of the last forager populations in Eastern Iberia. J. Archaeol. Sci. 40, 671–680. https://doi.org/https://doi.org/10.1016/j.jas.2012.09.005
Fernández-López de Pablo, J., Gómez-Puche, M., Esquembre-Bebiá, M.A. (2014) Casa Corona (Villena, Alicante, Spain). In: Sala Ramos, R. (Ed.), Los Cazadores Recolectores Del Pleistoceno y Del Holoceno En Iberia y El Estrecho de Gibraltar. Estado Actual Del Conocimiento Del Registro Arqueológico. Universidad de Burgos – Fundación Atapuerca, Burgos, pp. 331–337.
Pérez-Amorós, L., Hernández-Alcaraz, L. (2006) Noticia sobre las prospecciones arqueológicas realizadas en la partida de El Campo (Villena, Alicante). Recerques del Museu d'Alcoi 15, 93–102.
van Klinken, G.J. (1999) Bone Collagen Quality Indicators for Palaeodietary and Radiocarbon Measurements. J. Archaeol. Sci 26, 687–695.

Cioclovina, Romania

Dan Grigorescu

Analyzed individual:
- Cioclovina (Cioclovina 1): 33293-32014 calBP

Archeological description previously published in Posth et al., 2016 and Fu et al., 2016.

Criewen, Germany

Bettina Jungklaus, Thomas Terberger

Analyzed individual:
- CRW001 (Criewen 1961- Individual 2): 6839-6572 calBP

The site Criewen 4 is located in the lower Oder area on the western edge of the river. Two burials were partially destroyed before archaeologists started excavation of the site (Geisler / Wetzel, 1999). Of burial 1, only the lower part of the pit with the right arm, the pelvis and the legs was preserved. This adult individual was buried in supine position in reddish sands. A total of 36 perforated shells (*Theodoxus fluviatilis*) were found in the area of the upper legs and probably served as decoration. The most interesting discovery is a perforated mace head made of marble that was found in the disturbed part. It is highly likely that the object is part of the grave goods. The mace head finds parallels in the Middle Neolithic Rössen culture (Geisler / Wetzel, 1999; Cziesla, 2008). Burial 2 is an inhumation burial in supine position lying also in reddish sands with the lower part disturbed. This adult individual was found with about 3,000 perforated shells of local origin (*Theodoxus fluviatilis*; 1x *Valvata piscinalis*). Most of the shells were found near the skull and the chest and might have decorated clothes. Burial 2 was radiocarbon dated to c. 6,700 cal BP (KIA-4347: 5,890 ± 40 BP). The elevated 15N value suggests that some reservoir effect can be expected for the date and the true age of the internment is probably about 100 years younger. The first aDNA analysis provided U4 and U5 mtDNA haplogroups for the two individuals (Terberger et al., 2015), which are typical for the Mesolithic population of Central Europe (Bramanti et al., 2009; Posth et al., 2016). The Criewen individuals belong to the few Late Mesolithic burials of the 7th millennium calBP in the inland. The mace head found with burial 1 testifies to contacts with farming communities at that time (Geisler / Wetzel, 1999; Cziesla, 2008).

References:
Bramanti, B. / M. G. Thomas / W. Haak / M. Unterlaender / P. Jores / K. Tambets / I. Antanaitis-Jacobs / M. Haidle / R. Jankauskas / C.-J. Kind / F. Lueth / T. Terberger / J. Hiller / S. Matsumura / P. Forster / J. Burger, Genetic discontinuity between local hunter-gatherers and Central Europe's first farmers. Science 326, 2009, 137–140.
Cziesla, E., Zur bandkeramischen Kultur zwischen Elbe und Oder. Germania 86, 2008, 405–464.
Geisler, H. / G. Wetzel, Mittelsteinzeitliche und mittelalterliche Bestattungen vom "Rollmannsberg" bei Criewen, Lkr. Uckermark. In: E. Cziesla / T. Kersting / S. Pratsch.(eds.), Den Bogen spannen... Festschrift für Bernhard Gramsch zum 65. Geburtstag. Beiträge zur Ur- und Frühgeschichte Mitteleuropas 20 (Weissbach 1999) 259–280.
Posth, C. / G. Renaud / A. Mittnik / D.G. Drucker / H. Rougier / Ch. Cupillard / F. Valentin / C. Thevenet / A. Furtwängler / Ch. Wißing / M. Francken / M. Malina / M. Bolus / M. Lari / E. Gigli / G. Capecchi / I. Crevecoeur / C. Beauval / D. Flas / M. Germonpré / J. van der Plicht / R. Cottiaux / B. Gélly / A. Ronchitelli / K. Wehrberger / D. Grigourescu / J. Svoboda / P. Semal / D. Caramelli / H. Bocherens / K. Harvati / N. J. Conard / W. Haak / A. Powell / J. Krause, Pleistocene Mitochondrial Genomes Suggest a Single Major Dispersal of Non-Africans and a Late Glacial Population Turnover in Europe. Current Biology 26, 2016, 1–7.
Terberger, T. / A. Kotula / S. Lorenz / M. Schult / J. Burger / B. Jungklaus, Standing upright to all eternity – the Mesolithic burial site at Groß Fredenwalde, Brandenburg (NE Germany). Quartär 62, 2015, 133–153.

Cuiry-lès-Chaudardes (Les Fontinettes), France

Corinne Thevenet

Analyzed individual:
- Chaudardes 1 (CuiryLesChaudardes1): 8350-8036 calBP

Archeological description previously published in Posth et al., 2016 and Fu et al., 2016.

Doggerland, The Netherlands

Eveline Altena, Luc Amkreutz, Paul Storm, Liesbeth Smits, Johannes van der Plicht

Analyzed individuals:

- DOG001 (A10-007_V002_M003): 9680-9536 calBP
- DOG002 (U 2014/12.4; A10-007_V003_M006): 10371-10188 calBP
- DOG003 (9507-04; A10-007_V005_M009): 11063-10516 calBP
- DOG004 (U 2014/12.17; A10-007_V006_M011): 10222-9909 calBP
- DOG006 (A10-007_V009_M017): 8636-8452 calBP
- DOG007 (U 2014/12.3; A10-007_V001_M001): 9526-9151 calBP
- DOG009 (A10-007_V013_M023): 8990-8642 calBP
- DOG010 (A10-007_V014_M024): 8980-8546 calBP

Between 20,000 and 8,000 years ago the North Sea was dry land, inhabited by late Paleolithic and Mesolithic hunter-gatherers. This stretch of land is known as 'Doggerland' (Fig. S1). Large-scale fishing activities and modern sand extraction for construction of new land and beach replenishment have brought up a unique archaeological and paleoenvironmental record consisting of vast quantities of well-preserved remains of humans, artefacts, animals and other environmental remains. Despite the lack of detailed contextual information several pilot studies demonstrate that these finds bear significant scientific value to the archaeological understanding of prehistoric humans living in a highly dynamic landscape (Amkreutz et al., 2017, 2019; Amkreutz and Van der Vaart-Verschoof 2022; Dekker et al., 2021; Glimmerveen et al., 2004; Storm et al., 2014, 2021; van der Plicht et al., 2016). The eight samples from Doggerland that are reported here are isolated finds of human skeletal fragments that have been found by chance at different locations and at different times. Most can be traced back to their original find location or zone with some degree of accuracy. Some pieces are owned by private collectors and made available for scientific research, others were donated to archaeological institutions. Carbon and nitrogen isotope analysis on a collection of prehistoric human remains from the North Sea indicated that freshwater fish and marine food sources formed a substantial component in the diet of these people, causing issues with calibration of radiocarbon dates due to reservoir effects (van der Plicht et al., 2016). This should also be kept in mind for samples for which no carbon and nitrogen isotope analysis was performed.

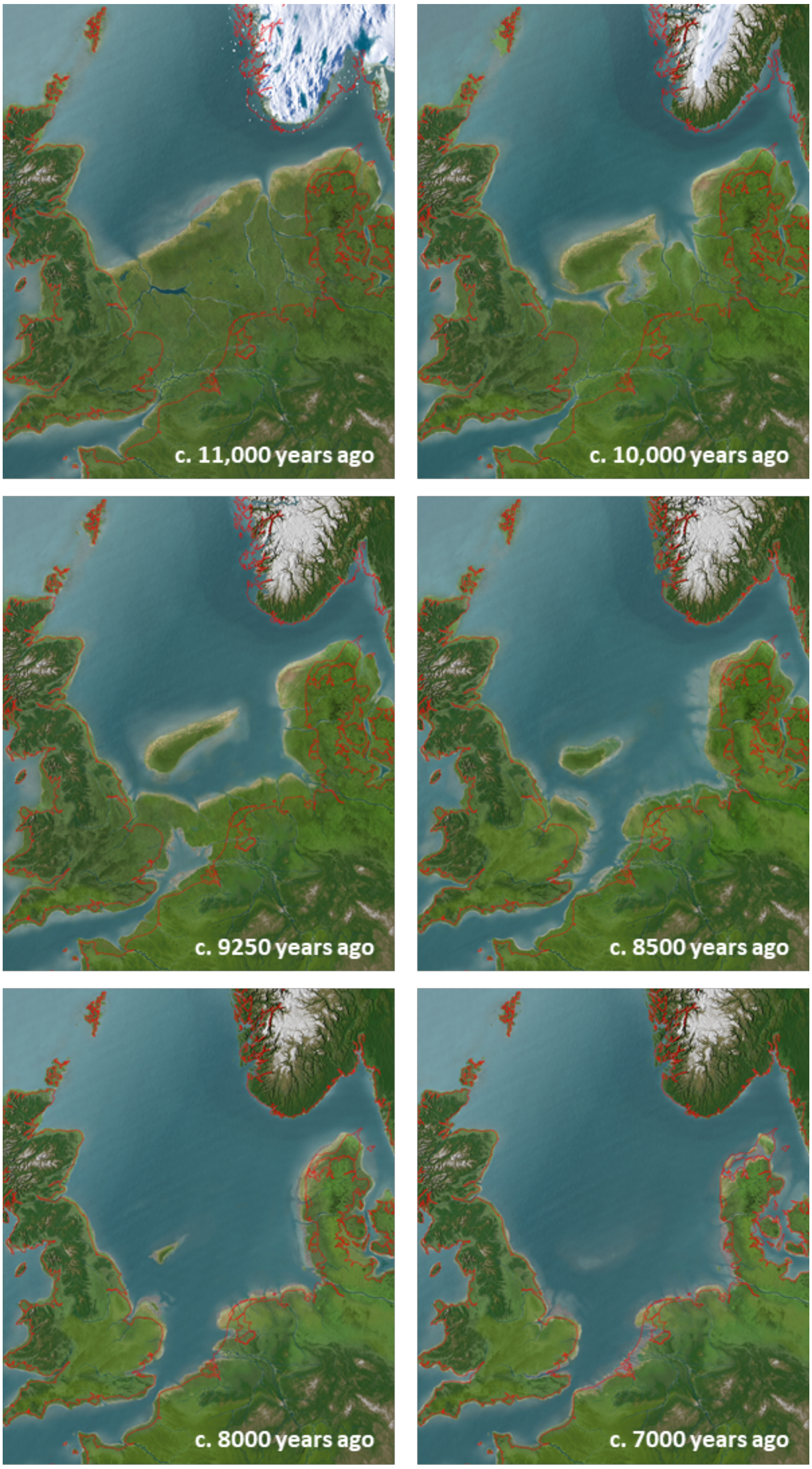

Figure S1. Representation of the Doggerland changing landscape across the early Holocene. Reconstruction by Kim Cohen (Utrecht University), Marc Hijma (Deltares) and Luc Amkreutz (Rijksmuseum van Oudheden). Present-day coast lines marked in red (©Rijksmuseum van Oudheden/Olav Odé).

Individual U 2014/12.3 (A10-007_V001, sample M001, Jan Glimmerveen; Luc Amkreutz, National Museum of Antiquities) is represented by the left half of a mandible with all premolars and molars. The first premolar was selected for DNA analysis. The genetic sex is male. A morphological analysis also indicated that the sex was most likely male and that he died in his adult years, probably aged somewhere between 25 and 45 years (Storm et al., 2014). The mandible was described as being overall robust, including a retromolar gap (Storm et al., 2010, 2014). The diet of this person was dominated by both freshwater fish and marine food sources ($\delta^{13}C$ −15.4 ‰, $\delta^{15}N$ 15.7 ‰) (van der Plicht et al., 2016). It dates to the early Mesolithic period (7546-7318 calBC, GrA-11642, 8370 ± 50 BP; Glimmerveen 2004; Storm 2010), but the freshwater fish and marine components of his diet can have caused a substantial reservoir effect. The hemi-mandible was found on October 29, 1993 by Piet van Es on board of a fishing ship, but the precise location is unknown.

Individual A10-007_V002 (sample M003, Klaas Post, North Sea Fossils) is a left femur diaphysis with a partial proximal extremity. A piece of bone was sampled from the diaphysis for DNA analysis. The genetic sex is male, but a morphological sex estimation was not performed. Based on what is preserved of the bone, the epiphysis of the greater trochanter seems fully fused, which leads to an age estimation of ≥16 years based on Scheuer and Black (2000). It dates to the early Mesolithic period (7728-7586 calBC, MAMS-48201, 8627 ± 35 BP; this publication). It was recovered from the Eurogeul on September 28, 2011 by Albert Hoekman on board of a fishing ship.

Individual U2014/12.4 (A10-007_V003, sample M006, Jan Glimmerveen; Luc Amkreutz, National Museum of Antiquities) corresponds to the right half of a mandible with part of the *mentum osseum* and the third molar, but without the ramus. A piece of bone was taken from the mandible for DNA analysis. The genetic sex is male, a morphological sex estimation could not be performed. Based on the dentition this individual most likely died in his adult years. It was dated to the early Mesolithic period (8422-8241 calBC, MAMS-34582; 9091 ± 37 BP; this publication). It was recovered west of the Brown Bank on October 17, 2011 by Albert Hoekman on board of a fishing ship.

Individual 9507-04 (A10-007_V005, sample M009, Klaas Post, North Sea Fossils; Rob van Eerden, Archaeological provincial depot of Noord-Holland) is a frontal bone preserving part of the right orbit, from which bone powder was directly drilled for DNA analysis. The genetic sex is male, but an osteological sex and age estimation was not performed. This individual had a terrestrial diet with a substantial component of freshwater fish ($\delta^{13}C$ −24.4 ‰, $\delta^{15}N$ 12.2 ‰) (van der Plicht et al., 2016). This fragment is dated to the late Paleolithic/early Mesolithic (9110-8610 calBC, GrA-57506; 9440 ± 45 BP; van der Plicht et al., 2016), but the freshwater fish in this individual's diet can have caused a substantial reservoir effect. It was recovered near the Noordhinder trenches on board of a fishing ship on January 16, 2013.

Individual U 2014/12.17 (A10-007_V006, sample M011, Klaas Post, North Sea Fossils; Luc Amkreutz, National Museum of Antiquities) is represented by a humeral diaphysis. A piece of bone was removed for DNA analysis. The genetic sex is male. Morphological age and sex estimations were not possible but based on the dimensions of the bone, this individual was most likely at least a teenager. This individual had a substantial component of marine food sources in his diet ($\delta^{13}C$ −13.4 ‰, $\delta^{15}N$ 14.1 ‰) (van der Plicht et al., 2016). It was dated in the early Mesolithic (8271-7966 calBC, GrA-62225, 8945 ± 45 BP; van der Plicht et al., 2016), but the marine foods in this individual's diet can have caused a reservoir effect. The fragment was recovered near the Brown Bank on board of a fishing ship on September 16, 2014 by Albert Hoekman.

Individual A10-007_V009 (sample M017, Dick Duineveld) is a right maxilla with two premolars and the first molar. The second premolar was used for DNA analysis. The genetic sex is male, a morphological sex estimation was not possible. Based on the dentition this individual most likely died in his adult years. This fragment is dated to the middle Mesolithic (6678-6552 calBC, MAMS-34584, 7781 ± 35 BP; this publication). It was found at an artificial sandbank (Sand Motor) on the coast of Loosduinen in September 2017 by Dick Duineveld. The sand for the Sand Motor was retrieved from the North Sea, approximately 10 km north-west of the Sand Motor.

Individual A10-007_V013 (sample M023, Mirte Medendorp) is a left temporal bone, without the *pars petrosa*, from which bone powder was directly drilled for DNA analysis. The genetic sex is male, which is supported by the sex estimation based on the *processus mastoideus* and the supramastoid crest, according to Arbeitsgruppe Europäischer Anthropologen (1979). There are no clear age markers present, but this fragment most likely belonged to an adult. Carbon and nitrogen isotope analysis was performed according to van der Plicht et al. (2016). His diet was predominantly terrestrial but with substantial freshwater and marine components ($\delta^{13}C$ = -17.61 ‰, $\delta^{15}N$ = +16.17 ‰). It was dated to the middle Mesolithic period (7036-6696 calBC, GrA-68069, 7950 ± 45 BP; this publication), but the marine foods in this person's diet can have caused a considerable reservoir effect. It was found on the beach at the Maasvlakte 2 by Mirte Medendorp in the summer of 2016. Maasvlakte 2 is part of the harbour of Rotterdam and was constructed with sand from the North Sea, which was retrieved approximately 10 km north-west from the Maasvlakte 2.

Individual A10-007_V014 (sample M024, Sander and Marcel Dikstra) is a frontal bone, including the upper parts of the orbits, from which bone powder was directly drilled for DNA analysis. The genetic sex is female and confirms the morphological sex estimation according to Arbeitsgruppe Europäischer Anthropologen (1979). Possibilities for age estimation were limited but it is estimated, based on the dimensions of the fragment that it belonged to someone at least 15 years of age. Carbon and nitrogen isotope analysis was performed according to van der Plicht et al., (2016). This analysis indicated that this person had a predominantly terrestrial diet but with a substantial component of freshwater fish ($\delta^{13}C$ = -23.75 ‰, $\delta^{15}N$ = +12,9 ‰) (van der Plicht et al., 2016). He was dated to the middle Mesolithic period (7023-6602 calBC, GrA-63799, 7870 ± 45 BP; van der Plicht et al., 2016), although the freshwater fish in this person's diet can have caused a considerable reservoir effect. The fragment was found on the beach at the Maasvlakte 2 by Theo Dikstra in 2014.

References:

Amkreutz, L., Niekus M., Smit B., Schiltmans D., 2017. Meer dan bijvangst! De prehistorische archeologie van de Noordzee, Cranium 2017, 34-47.

Amkreutz, L., Spithoven, M., 2019. Hunting beneath the waves. Bone and antler points from the North sea Doggerland off the Dutch coast, in: D. Gross, H. Lübke, H. Meadows, D. Jantzen (eds), Working at the sharp end: from bone and antler to Early Mesolithic life in Northern Europe. Untersuchungen und Materialien zur Steinzeit in Schleswig-Holstein und im Ostseeraum 10: Kiel/Hamburg.

Amkreutz, L., van der Vaart-Verschoof, S., 2022. Doggerland. Lost world under the North Sea. Leiden, Sidestone Press.

Dekker, J., Sinet-Mathiot, V., Spithoven, M., Smit, B., Wilcke, A., Welker, F., Verpoorte, A., Soressi, M., 2021. Human and cervid osseous materials used for barbed point manufacture in Mesolithic Doggerland, Journal of Archaeological Science: Reports 35, 102678.

Glimmerveen, J., Mol, D., Post, K., Reumer, J. W. F., van der Plicht, H., de Vos, J., van Geel, B., van Reenen, G., Pals, J. P., 2004. The North Sea project: the first palaeontological, palynological, and

archaeological results, in: N. C. Flemming (ed), Submarine prehistoric archaeology of the North Sea. Research priorities and collaboration with industry. CBA Research Report 141, English Heritage/Council for British Archaeology, 43-52.
Scheuer, L., Black, S., 2000. Developmental Juvenile Osteology. London, Academic press.
Storm, P., 2010. Start onderzoek *Homo sapiens* resten Noordzee: micro-evolutie in de Lage Landen, Cranium nov, 63-66.
Storm, P., Altena, E., van der Colk, T., Kootker, L., Mol, D., Post, K., 2014. Mesolithische man uit de Noordzee. Analyse van een opgevist prehistorisch stuk onderkaak, Grondboor & Hamer 68(4/5), 131-137.
Storm, P., Altena, E., Amkreutz, L., Smit, B., Peeters, H., 2021. De opmerkelijke robuuste morfologie van de prehistorische onderkaak van Burgh-Haamstede, Cranium 38-1, 42-54.
van der Plicht, J., Amkreutz, L. W. S. W., Niekus, M. J. L. Th., Peeters, J. H. M., Smit, B. I., 2016. Surf'n turf in Doggerland: dating, stable isotopes and diet of Mesolithic human remains from the southern North Sea, Journal of Archaeological Science Reports 10, 110-118.

Donkalnis, Lithuania

Rimantas Jankauskas, Justina Kozakaitė

Analyzed individuals:

- DON005 (Donkalnis 2): 8413-8339 calBP
- DON006 (Donkalnis 3): 6733-6505 calBP

The Donkalnis site is located in the central north-western part of Lithuania, and is situated on a small moraine hill (elevation up to 156 m a.s.l.) towering above the swamp at the northern part of the lake Biržulis where the rivulet Druja flows into it. In some stages of the Stone Age the hill may have been an island (Butrimas et al., 1985, 2012; Kunskas et al., 1985). It is a set of settlements dating to the Mesolithic, Late Neolithic and Early to Middle Bronze Age and burials dated to a Mesolithic and Neolithic.
After the first melioration of the lake in the 1930s the site became accessible on foot and local residents started exploiting it as a gravel quarry. The site received archaeologists' attention only in 1979, and was excavated in 1981-1983 by archaeologist A. Butrimas. The result was the discovery of seven undisturbed inhumation graves and remains of at least six individuals, whose graves were disturbed due to quarry activities. The site contained also numerous pottery fragments, flint artefacts and pits. Stable isotope data on dietary patterns of the Donkalnis individuals indicate a mixed diet of freshwater fish and hunted forest animals, although plant based foods was also likely (Antanaitis-Jacobs et al., 2009; Piličiauskas et al., 2017).
Donkalnis 2 and Donkalnis 3 were located in shallow pits in close vicinity, thus the archaeologists initially considered them being a double grave. Donkalnis 2 (morphologically identified as a young male and also confirmed genetically) was decorated by the symmetrical placement of 57 animal tooth-pendants i.e., a necklace of 27 teeth was found next to the individual, and teeth of different animals were placed on top of the frontal bone, in both eye-sockets, nostrils, etc. Archaeologist A. Butrimas interpreted this as a symbolic meaning, most likely as an indication of the special social status of the buried person (Butrimas et al., 1985). To the right side of Donkalnis 2, Donkalnis 3 (morphologically identified as a young female but genetically identified here as male) was buried with strongly flexed legs, without grave goods, and much less ochre in comparison with other graves in the Donkalnis site. The skeletons of these two individuals were then placed in display at the National Museum of

Lithuania. After a first radiocarbon dating, doubts arose that the individuals were indeed buried simultaneously in a double grave. Therefore, additional dating was performed in 2021. The new dates confirmed the previous indication of a significant time span between the two burials: Donkalnis 2 was dated to 7593±33 BP (FTMC-IL27-2, ~8400 calBP), and Donkalnis 3 was dated to 5843±30 BP (FTMC-IL27-1, ~6,600 calBP), attributing them to the Mesolithic and Subneolithic (Narva) periods, respectively. Recent $^{87}Sr/^{86}Sr$ analysis (Piličiauskas et al., 2022) indicates that the most plausible areas of origin for Donkalnis 2 are the northeastern Baltic, southern Finland or Karelia. Such a distant origin (~500–700 km) for a relatively high number of analysed Lithuanian hunter-gatherers (6/36) is not unexpected in the light of aDNA and archaeological data (Mittnik et al., 2018). In contrast, Donkalnis 3 was most probably born closer to the site, such as on the Baltic coast (~85 km).

References:

Antanaitis-Jacobs I., Richards M., Daugnora L., Jankauskas R., Ogrinc R., 2009. Diet in early Lithuanian prehistory and the new stable isotope evidence. Archaeologia Baltica 12, p. 12-30.
Butrimas A., 2012. *Donkalnio ir Spigino Mezolito – Neolito kapinynai. Seniausi laidojimo paminklai Lietuvoje*. Vilnius: Vilniaus dailės akademijos leidykla.
Butrimas A., Kazakevičius V., Česnys G., Balčiūnienė I., Jankauskas R., 1985. Ankstyvieji Virvelinės keramikos kultūros kapai Lietuvoje. Lietuvos archeologija 4, p. 14-24.
Kunskas R., Butrimas A., Česnys G., Balčiūnienė I., Jankauskas R., 1985. Duonkalnis: vėlyvojo neolito gyvenvietė, alkas ir kapinynas. Lietuvos archeologija 4, p. 25-66.
Mittnik A., Wang C.C., Pfrengle S., Daubaras M., Zariņa G., Hallgren F., Allmäe R., Khartanovich V., Moiseyev V., Tõrv M., Furtwängler A., 2018. The genetic prehistory of the Baltic Sea region. Nature communications 9(1), pp. 1-11.
Piličiauskas G., Jankauskas R., Piličiauskienė G., Craig O.E., Charlton S., Dupras T., 2017. The transition from foraging to farming (7000–500 cal BC) in the SE Baltic: A re-evaluation of chronological and palaeodietary evidence from human remains. Journal of Archaeological Science: Reports 14, p. 530-542.
Piličiauskas G., Simčenka E., Lidén K., Kozakaitė J., Miliauskienė Ž., Piličiauskienė G., Kooijman E., Šinkūnas P., Robson H.K., 2022. Strontium isotope analysis reveals prehistoric mobility patterns in the southeastern Baltic area. Archaeological and Anthropological Sciences 14, pp. 1-21. https://doi.org/10.1007/s12520-022-01539-w

Dolní Věstonice, Czechia

Jirí Svoboda, Sandra Sázelová

Analyzed individuals:

- DLV005 (DV14): 31146-30867 calBP
- DLV006 (DV15): 31108-30847 calBP

Archeological description previously published in Posth et al., 2016 and Fu et al., 2016.

Drigge, Germany

Thomas Terberger, Claudia Hoffmann, Bettina Jungklaus

Analyzed individual:

- DRI001 (Drigge skull): 7412-6947 calBP

The Drigge site is located at the southern coast of Rügen island in northern Germany. It was detected in 1934 during sand dredging. Some large finds were collected which originated from a depth of about 2-5 m below sea level (Terberger, 1999). According to sediment remains on the finds they were originally lying in a humic layer. We can assume that the finds originate from a coastal site. A coastal location of the site is supported by one seal bone (*Halichoerus grypus*) and by one bone of a smaller whale (*Delphinapterus leucas*) in the find assemblage. Altogether, 55 finds of bone and antler were collected. Most of them can be determined as red deer. There are three T-shaped antler axes and several pieces of distinctive production waste of such antler axes, which are typical for the Ertebølle Culture (c. 7,400–6,200 calBP). An AMS-date (UtC-6938: 6,070±60 BP) on one of these specimens corresponds to the typological evidence and assigns the finds to the aceramic phase of the Ertebølle Culture. A largely preserved human skull also belongs to the assemblage. This is supported by the same type of bone preservation and a radiocarbon date of ~7,200 calBP (Uz-4093: 6,250±80 BP). This older date might be explained by some reservoir effect and probably the skull belongs to the same time period as the rest of the material. The skull is assigned to an adult male individual and shows cutmarks, which are interpreted as the result of scalping activities. First a long cut along the sagittal suture was performed followed by smaller cut marks suggesting subsequent removal of the skin from both sides of the skull. The Drigge skull provides rare evidence of Mesolithic systematic scalping (Terberger, 1999).

References:
Terberger, T., Die endmesolithischen Funde von Drigge, Kr. Rügen. Kannbialen auf Rügen? Bodendenkmalpflege in Mecklenburg-Vorpommern 46, Jahrbuch 1998 (1999), 7-44.

Farman, France

Frédérique Valentin, Bénédicte Souffi

Analyzed individual:

- FRM001 (Loc 1-168/927-2322): 10580-9669 calBP (layer)

The human remains discovered at the site "62 rue Henry-Farman" in Paris, during excavations carried out by the *Institut National de Recherches Archéologiques Préventives* (Souffi et al., 2013), were found among other remains, including numerous animal bones and other remains, within a concentration attributed to the early 8th millennium BC (locus 1). They consist of two fragments of the same mandible (genetically analysed here) and a portion of a femur shaft. The bones are gracile and of adult size but it is difficult to assess from macroscopic examination whether they represent the same individual or not. The fragmentation, which occurred when the bones were already dry, is of natural or accidental origin. They display no animal marks, scratches or gnawings, nor any cut marks or traces of human teeth that might suggest cannibalism. Even if it is not possible to completely rule out the hypothesis of a disturbed burial, it can be assumed, given the current state of research, that these are human remains intentionally discarded in a domestic area because they were found near animal remains still in articulation.

References:
Souffi B., Marti P., Chaussé C., Griselin S., Bridault A., David E., Drucker D., Gosselin R., Granai S., Hamon C., Leduc C., Vanhaeren M., Valentin F. 2013. Occupations mésolithiques en bord de Seine : le site de Paris 15ème arrondissement « 62 rue Henry-Farman ». Organisation et fonctionnement. *In*: Valentin B., Souffi B., Ducrocq T., Fagnart J.-P., Seara F., Verjux C. (éds.), *Palethnographie du Mésolithique : recherches sur les habitats de plein-air entre Loire et Neckar / Mesolithic palethnography : Research on open-air campsites from the river Loire to the Neckar,* pp. 13-35, Séances de la Société Préhistorique Française 2, Paris.

Fournol, France

Sébastien Villotte, André Morala, Mathieu Rué, Stéphane Madelaine, Laurent Crepin, Jean Baptiste Caverne, Emmy Bocaege, Mona Le Luyer

Analyzed individual:
- FRL006 (FL15 n° 85): 29024-28451 calBP

The Fournol site is located in Soturac, Lot (France). It is a relatively small rock shelter (ca. 10 m long and 3 m depth), open to the south and developed into Coniacian limestone 162 m above sea level. Excavations at Fournol started at the very beginning of the 20th century. The archeological deposit was greatly disturbed by these works and, despite connections between the excavator (S. Gaillard) and prehistorians of the time (E. Cartailhac, H. Breuil and A. Delugin), the site was largely forgotten. After World War II, the site was occasionally excavated again, but the nature and location of the recovered material are unknown (Morala 1979, 1984, 2017). In 1972, an archaeological survey revealed its existence and its industrial content, which was attributed to the Lower Solutrean and Middle Gravettian (Morala 1979, 1984), and more recently also to the Late Aurignacian (Morala 2017). Following a new major clandestine degradation that occurred on the site in the early 2000s, annual excavations were undertaken from 2015 to 2018 (Morala et al., 2017). Three main geoarcheological units have been identified so far within and in front of the rock shelter, with a 15° to 5° slope to the outside. Unit 1 is formed by a dark brown, carbonated sandy loam matrix with limestone clasts (gravels to blocks). This unit is several decimeters thick (possibly close to one meter in some areas), and was highly disturbed, both by bioturbation and by the previous clandestine excavations. Faunal and human remains (more than 200), as well as lithic material characteristic of the Middle Gravettian (“Gravettes”, “microgravettes”, back flakes and some Noailles burins) were found in this unit (Morala 2017; Villotte et al., 2019). The artifact assemblage seemed homogenous, apart from a few modern artifacts (some glass shards and small metallic fragments) likely related to the previous excavations, and no evidence of intermixture between archeological layers from different cultures was found. One of the human remains from Unit 1 was directly dated to 24820 + 220 BP - 28319-27331 cal. BP (Morala 2015; Villotte et al., 2019). The fragment of temporal bone (N° 85) analysed here was discovered in 2015 in the square O22 (see Fig 1 in Villotte et al., 2019), in an area that was disturbed by 21st century clandestine excavations.

References:
Morala, A., 1979. Soturac (Lot) - Les Ardailloux et Couvert. Gallia Préhistoire, 22. pp. 648–649.
Morala, A., 1984. Périgordien et Aurignacien en Haut-Agenais : Etude d'ensembles lithiques. In: Archives d'Ecologie Préhistorique, Ecole des Hautes Etudes en Sciences Sociales, Tome 7, Toulouse.
Morala, A., 2015. Abri de Fournol, Soturac, Lot. La Revue des Musées de France, 2. p. 56.

Morala, A., 2017. Soturac. Fournol (Lot). In: Bilan scientifique régional d'Occitanie 2015 - Direction Régionale des Affaires Culturelles Midi-Pyrénées, Service Régional de l'Archéologie et de la Connaissance du Patrimoine. pp. 137–138.
Villotte, S., Crépin, L., Rué, M., Bocaege, E., Le Luyer, M., Madelaine, S., Caverne, J.-B., Morala, A., 2019. Evidence for previously unknown mortuary practices in the Southwest of France (Fournol, Lot) during the Gravettian. Journal of Archaeological Science: Reports, 27. p. 101959.

Troisième caverne of Goyet, Belgium

Hélène Rougier, Isabelle Crevecoeur, Patrick Semal, Hervé Bocherens, Helen Fewlass, Asier Gómez-Olivencia, Sahra Talamo, Christoph Wißing

Analyzed individuals:

- GOY001 (Goyet Q100-8): 27678-27246 calBP
- GOY007 (Goyet Q-1): 28012-27626 calBP
- GOY009 (Goyet 2878-18): 26360-25944 calBP
- GOY014 (Goyet 2878-15): 28257-27703 calBP
- GoyetQ376-3 (Goyet Q376-3): 35170-34519 calBP
- GoyetQ116-1 (Goyet Q116-1): 34809-34340 cal BP
- GoyetQ376-19 (Goyet Q376-19): 27717-27301 calBP
- GoyetQ53-1 (Goyet Q53-1): 28390-27773 calBP

The Troisième caverne of Goyet in Belgium was excavated in the latter half of the 19th and beginning of the 20th century, and again at the end of the 1990s. The main excavations were performed in 1868 by Edouard Dupont who identified Paleolithic human occupations (Dupont, 1872) that were later attributed to the Middle and Upper Paleolithic (including Mousterian, Lincombian-Ranisian-Jerzmanowician, Aurignacian, Gravettian, and Magdalenian material) as well as to the Neolithic and historic period (Flas, 2006). Starting in 2008, the reassessment of both the human and faunal collections from the site yielded new human remains. Due to the lack of detailed documentation of the excavated material, their association to a specific occupation was impossible and a multidisciplinary study of the human remains and their context was undertaken. Morphometric and taphonomic features, completed by the direct radiocarbon dating of the remains, were used to assign them to different periods. In combination with isotopic and genetic analyses (Rougier et al., 2016b; Wißing et al., 2016, 2019) the results allowed for the specimens to be assigned to either late Neandertals or *Homo sapiens*. We identified modern human specimens from different periods of the Upper Paleolithic, namely the Aurignacian, Gravettian, and Magdalenian. They include fragmentary elements from the cranial and infracranial skeleton that represent at least 10 individuals (Rougier et al., 2016a). In the present study, we report the aDNA of four bone specimens (Goyet Q100-8, a rib fragment; Goyet Q-1, a clavicle diaphysis; Goyet 2878-18, a tibia diaphysis; and Goyet 2878-15, a humerus diaphysis fragment), which are directly dated to the Gravettian (this study). In addition, we analyzed the nuclear DNA of Goyet Q376-3, a humerus diaphysis fragment directly dated to the Aurignacian and whose mtDNA was previously produced (Posth et al., 2016). Finally, two additional samples were re-analyzed in order to produce a higher nuclear DNA coverage than reported in Fu et al. (2016): Goyet Q376-19 (a Gravettian humerus diaphysis fragment), and Goyet Q53-1 (a Gravettian fibula diaphysis fragment). Each specimen represents a different individual with the great majority of them representing adults/adolescents and only Goyet Q-1 belonging to a juvenile.

Two new direct ^{14}C dates were performed on two human bones, Goyet Q116-1 and Goyet Q376-3. The collagen was extracted at the Department of Human Evolution, Max Planck Institute for Evolutionary Anthropology (MPI-EVA) in Leipzig (Germany) following the procedures presented by Fewlass et al. (2019). To supervise possible contamination introduced during the pre-treatment stage, a pretreated ^{14}C-free bone sample was used (Korlević et al., 2018). Prior to sending the sample to the AMS facility for AMS dating, the collagen yield, C:N ratios, together with isotopic values were evaluated and all passed the evaluation criteria (Goyet Q116-1 percentage of Collagen = 16.3 % and C:N = 3.2; Goyet Q376-3 percentage of Collagen = 15.4 % and C:N = 3.2) for good quality collagen (van Klinken, 1999; Talamo et al., 2021).

The collagen obtained from the two individuals was radiocarbon dated twice with an Accelerator Mass Spectrometer at two different radiocarbon laboratories (MAMS and ETH) in order to obtain very precise ^{14}C dates for calibration with the recently updated IntCal20 calibration curve (Bard et al., 2020; Reimer et al., 2020). The combined ^{14}C age of Goyet Q116-1 is 30,208 ± 78 BP and that of Goyet Q376-3 is 30,403 ± 82 BP (obtained using the R_Combine command in OxCal 4.4.2; Ramsey, 2009), which correspond to 95.4 % confidence intervals of 34809-34340 calBP and 35170-34519 calBP, respectively, for their calibrated ages. These new results confirm the attribution of Goyet Q116-1 and Q376-3 to the Aurignacian period (Posth et al., 2016).

References:

Bard, E., Heaton, T. J., Talamo, S., Kromer, B., Reimer, R. W. & Reimer, P. J., 2020. Extended dilation of the radiocarbon time scale between 40,000 and 48,000 y BP and the overlap between Neanderthals and *Homo sapiens*. *Proc. Natl. Acad. Sci. USA* 117: 21005-21007. doi: 10.1073/pnas.2012307117.

Dupont, E., 1872. L'Homme pendant les âges de la pierre dans les environs de Dinant-sur-Meuse, 2ème édition. C. Muquardt Ed., Bruxelles.

Fewlass, H., Tuna, T., Fagault, Y., Hublin, J.-J., Kromer, B., Bard, E. & Talamo, S., 2019. Pretreatment and gaseous radiocarbon dating of 40–100 mg archaeological bone. *Sci. Rep.* 9(1): 5342.

Flas, D., 2006. La transition du Paléolithique moyen au supérieur dans la plaine septentrionale de l'Europe. Les problématiques du Lincombien-Ranisien-Jerzmanowicien. Thèse, Université de Liège (Belgique).

Fu, Q., Posth, C., Hajdinjak, M., Petr, M., Mallick, S., Fernandes, D., Furtwängler, A., Haak, W., Meyer, M., Mittnik, A., Nickel, B., Peltzer, A., Rohland, N., Slon, V., Talamo, S., Lazaridis, I., Lipson, M., Mathieson, I., Schiffels, S., Skoglund, P., Derevianko, A. P., Drozdov, N., Slavinsky, V., Tsybanov, A., Grifoni Cremonesi, R., Mallegni, F., Gély, B., Vacca, E., Gonzáles Morales, M. R., Straus, L. G., Neugebauer-Maresch, C., Teschler-Nicola, M., Constantin, S., Moldovan, O. T., Benazzi, S., Peresani, M., Coppola, D., Lari, M., Ricci, S., Ronchitelli, A., Valentin, F., Thevenet, C., Wehrberger, K., Grigorescu, D., Rougier, H., Crevecoeur, I., Flas, D., Semal, P., Mannino, M. A., Cupillard, C., Bocherens, H., Conard, N. J., Harvati, K., Moiseyev, V., Drucker, D. G., Svoboda, J., Richards, M. P., Caramelli, D., Pinhasi, R., Kelso, J., Patterson, N., Krause, J., Pääbo, S. & Reich, D., 2016. The genetic history of Ice Age Europe. *Nature* 534: 200-205.

Korlević, P., Talamo, S. & Meyer, M., 2018. A combined method for DNA analysis and radiocarbon dating from a single sample. *Sci. Rep.* 8(1): 1-10.

Posth, C., Renaud, G., Mittnik, A., Drucker, D. G., Rougier, H., Cupillard, C., Valentin, F., Thevenet, C., Furtwängler, A., Wißing, C., Francken, M., Malina, M., Bolus, M., Lari, M., Gigli, E., Capecchi, G., Crevecoeur, I., Beauval, C., Flas, D., Germonpré, M., van der Plicht, J., Cottiaux, R., Gély, B., Ronchitelli, A., Wehrberger, K., Grigorescu, D., Svoboda, J., Semal, P., Caramelli, D., Bocherens, H., Harvati, K., Conard, N. J., Haak, W., Powell, A. & Krause, J., 2016. Pleistocene Mitochondrial

Genomes Suggest a Single Major Dispersal of Non-Africans and a Late Glacial Population Turnover in Europe. *Curr. Biol.* 26: 827-833.
Ramsey, C. B., 2009. Dealing with outliers and offsets in radiocarbon dating. *Radiocarbon* 51(3): 1023-1045.
Reimer, P. J., Austin, W. E. N., Bard, E., Bayliss, A., Blackwell, P. G., Bronk Ramsey, C., Butzin, M., Cheng, H., Edwards, R. L., Friedrich, M., Grootes, P. M., Guilderson, T. P., Hajdas, I., Heaton, T. J., Hogg, A. G., Hughen, K. A., Kromer, B., Manning, S. W., Muscheler, R., Palmer, J. G., Pearson, C., van der Plicht, J., Reimer, R. W., Richards, D. A., Scott, E. M., Southon, J. R., Turney, C. S. M., Wacker, L., Adolphi, F., Büntgen, U., Capano, M., Fahrni, S. M., Fogtmann-Schulz, A., Friedrich, R., Köhler, P., Kudsk, S., Miyake, F., Olsen, J., Reinig, F., Sakamoto, M., Sookdeo, A. & Talamo, S., 2020. The INTCAL20 northern hemisphere radiocarbon age calibration curve (0–55 cal kBP). *Radiocarbon* 62: 1-33. doi: 10.1017/RDC.2020.41.
Rougier, H., Crevecoeur, I., Beauval, C., Flas, D., Posth, C., Wißing, C., Furtwängler, A., Germonpré, M., Gómez-Olivencia, A., Semal, P., van der Plicht, J., Bocherens, H. & Krause, J., 2016a. The Troisième caverne of Goyet (Belgium): An exceptional site with both Neandertal and Upper Paleolithic human remains. *PESHE* 5: 211.
Rougier, H., Crevecoeur, I., Beauval, C., Posth, C., Flas, D., Wißing, C., Furtwängler, A., Germonpré, M., Gómez-Olivencia, A., Semal, P., van der Plicht, J., Bocherens, H. & Krause, J., 2016b. Neandertal cannibalism and Neandertal bones used as tools in Northern Europe. *Sci. Rep.* 6: 29005.
Talamo, S., Fewlass, H., Maria, R. & Jaouen, K., 2021. "Here we go again": the inspection of collagen extraction protocols for ^{14}C dating and palaeodietary analysis. *STAR: Sci. Technol. Archaeol. Res.* 7(1): 62-77.
van Klinken, G. J., 1999. Bone Collagen Quality Indicators for Palaeodietary and Radiocarbon Measurements. *J. Archaeol. Sci.* 26: 687-695.
Wißing, C., Rougier, H., Baumann, C., Comeyne, A., Crevecoeur, I., Drucker, D. G., Gaudzinski-Windheuser, S., Germonpré, M., Gómez-Olivencia, A., Krause, J., Matthies, T., Naito, Y. I., Posth, C., Semal, P., Street, M. & Bocherens, H., 2019. Stable isotopes reveal patterns of diet and mobility in the last Neandertals and first modern humans in Europe. *Sci. Rep.* 9: 4433.
Wißing, C., Rougier, H., Crevecoeur, I., Germonpré, M., Naito, Y. I., Semal, P. & Bocherens, H., 2016. Isotopic evidence for dietary ecology of late Neandertals in North-Western Europe. *Quat. Int.* 411: 327-345.

Groß Fredenwalde, Germany

Thomas Terberger, Andreas Kotula, Henny Piezonka, Bettina Jungklaus, Franz Schopper

Analyzed individuals:

- GFW001 (GF-1962-Individual1): 7918-7682 calBP
- GFW002.3 (GF-1962-Individual 4): 7972-7782 calBP
- GFW004 (GF-2014-feature 8): 8426-8224 calBP
- GFW005 (GF-2014-feature 1/4): 7160-6946 calBP
- GFW007 (Individual 3): 8159-7934 cal BP
- GFW008 (Individual 5): 8037-7867 cal BP
- GFW009 (Individual 6): 8180-7984 cal BP

The Mesolithic burial site Groß Fredenwalde is located in Uckermark, northeastern Germany, and it was accidently detected on top of a prominent hill in 1962. Since then the site is known for the

“multiple burial” (Gramsch & Schoknecht 2003). Analysis of the human remains identified three adult individuals and three children (Jungklaus et al., 2016). Recent studies of archive documents allowed the identification of at least two separate burials found in 1962 (Kotula et al., 2020). In 2012 new fieldwork started and further burials on the site could be excavated (Terberger et al., 2015). Recently, additional burials were identified for a total of twelve Late Mesolithic individuals originating from at least eight interments in a restricted area. Most of the burials date to the early Atlantic period (~8,400 to 7,800 calBP). ^{15}N-values of c. -11.3 to -12.3 ‰ for the individuals suggest some level of reservoir effect. Two AMS-dates on deer tooth pendants from the old findings provide more reliable dates of ~8,000 calBP. The burial of a young man (feature 1/4), however, is about 1,000 years younger (~6,900 calBP). The individual was probably put into the burial pit in an upright position and after decomposition the skeleton collapsed. The individual dates to the time when early LBK farmers had already established in Uckermark since ~7,200 calBP (Cziesla, 2008; Terberger et al., 2015; Ismail-Weber, 2017). The closest LBK settlements were located just ~12 km east of Groß Fredenwalde. Archaeological fieldwork has not been completed yet and further burials might be present at the site. Currently, feature 1/4 remains an isolated evidence for the more recent Mesolithic burial phase. An additional Neolithic burial dated to the later 6th millennium calBP was found a few metres from the Mesolithic interments. This new evidence shows that the Groß Fredenwalde hill, with its prominent position in the landscape, was a burial site for multiple Stone Age phases.
Kinship relationships were detected among the Groß Fredenwalde individuals. The female Individual 3 (GFW007) is first-degree related with the male Individual 1 (GFW001) and second-degree related with the male child Individual 4 (GFW002.3), which was found lying on the abdomen of Individual 3.

References:

Cziesla, E., Zur bandkeramischen Kultur zwischen Elbe und Oder. Germania 86, 2008, 405-464.
Gramsch, B. / U. Schoknecht, Gross Fredenwalde, Lkr.Uckermark – eine mittelsteinzeitliche Mehrfachbestattung in Norddeutschland. *Veröffentlichungen des Brandenburgischen Landesmuseum für Ur- und Fruhgeschichte* 34, 2000 (2003), 9-38.
Ismail-Weber, M., …100 km from the next settlement… Mobility of Linear Pottery Groups in Brandenburg, North-Eastern Germany. In: S. Scharl / B. Gehlen (eds.), Mobility in Prehistoric Sedentary Societies. Papers of the CRC 806 Workshop in Cologne 26-27 June 2015. Kölner Studien zur prähistorischen Archäologie 8 (Köln 2017) 75-117.
Jungklaus, B. / A. Kotula / T. Terberger, New investigations on the Mesolithic burial of Groß Fredenwalde, Brandenburg – first results. In: J. Grünberg / B. Gramsch / J. Orschiedt (eds.), Mesolithic burials – Rites, symbols and social organisation of early postglacial communities (Halle 2016) 419-433.
Kotula, A. / H. Piezonka / T. Terberger, The Mesolithic Cemetery of Groß Fredenwalde (NE Germany) and its Cultural Affiliations. Lietuvos Archeologija 46, 2020, 65-84.
Terberger, T. / A. Kotula / S. Lorenz / M. Schult / J. Burger / B. Jungklaus, Standing upright to all eternity – the Mesolithic burial site at Groß Fredenwalde, Brandenburg (NE Germany). Quartär 62, 2015, 133-153.

Hohle Fels, Germany

Nicholas Conard

Analyzed individuals:

- HohleFels10_79 (HF 79 IIb 876,HF 10 Ic 405): 15975-14286 calBP calBP

- HohleFels49 (HF 49 Ib1 66): 15878-15311 calBP

Archeological description previously published in Posth et al., 2016 and Fu et al., 2016.

Ḥou Amieva, Spain

Borja González-Rabanal, Manuel R. González Morales, Ana B. Marín-Arroyo, Belén López, Carmen Alonso Llamazares

Analyzed individual:

- AMI001 (JA02): 7256-7004 calBP
- AMI002 (JA01): 6849-6647 cal BP

Ḥou Amieva cave is located close to Llanes in the eastern part of Asturias province (northern Spain). The cave is a sinkhole draining an adjacent karstic depression that opens in Upper Carboniferous compact limestones. In 2010, during the exploration of the cave by the Sociedad Espeleológica y Barranquista ESCAR, several human and faunal remains were discovered in a short fossil gallery over the surface of a debris cone, located below an ancient entrance. A rescue excavation, directed by the Department of Anthropology from the University of Oviedo, allowed the identification of different human bones, including a skull, a coxal bone and a few more of a neonate individual.
Specimen JA02 consists of a left human tibia from a neonate individual. The length of this bone is 55 mm, which would imply an age of 32-34 gestation weeks (González-Rabanal et al., 2019). The death might have taken place close to birth, but it is difficult to say if the individual was born and later died or if the death occurred intrauterine. The tibia was dated to 6230 ± 40 (ICA-20B/10118) in the Late Mesolithic (~7,100 cal BP). This individual is four hundred years older than the petrous bone of another individual found at the site (JA01). Stable isotopes analysis of $\delta^{13}C$ and $\delta^{15}N$ shows lower carbon and nitrogen values than JA01, probably derived from the food consumed by the mother during pregnancy and not by milk consumption, confirming that the individual died before or soon after birth (González-Rabanal et al., 2019).
Specimen JA01 corresponds to a highly fragmented human skull found lying on its right side. Morphological features such as the nuchal crest, the supraorbital torus and the size of the mastoid process were used to assign the individual to a female, which is also genetically confirmed. Age determination was done through the evaluation of the cranial suture closure that provides an age range of 39-65 years. Palaeopathological analysis was undertaken to study four blunt trauma found in the left parietal and the occipital bones. They are oval depressions with evidence of healing and bone remodelling, therefore these lesions did not cause the death of the individual, but it is possible that the woman had some impediments during her life. A fragment of the skull was directly dated to 5910 ± 40 BP (ICA-19B/0173) corresponding to the Late Mesolithic (~ 6,700 calBP). Stable isotope analysis of $\delta^{13}C$ and $\delta^{15}N$ showed a mixed diet with terrestrial and marine proteins based on carbon and nitrogen enrichment.

Reference:

González-Rabanal, B., Alonso-Llamazares, C., González Morales, M. R., López, B., Marín-Arroyo, A. B. (2019). Estudio preliminar de los restos humanos mesolíticos de la cueva Ḥou Amieva (Llanes, Asturias). In: XXI Congreso de la Sociedad Española de Antropología Física, Universidad de Granada, 24-26 de Junio de 2019.

Igren' 8, Ukraine

Alexandra Buzhilova

Analysed individual:

- IGR001 (9317): 7661-7507 calBP

The multi-layered Igren' 8 site was located on a bank of the Samara river, a tributary of the Dnepr River. It was discovered in 1945 by an expedition led by the Institute of Archeology of the Academy of Sciences in Ukraine (Dobrovolsky, 1949). The site is dated archaeologically to the Neolithic – Eneolithic. The early Eneolithic in the area is associated with the local Sredniy Stog II archaeological culture (Telegin, 1973). Preserved bone remains date both to the Neolithic and later periods. The human remains are stored in the Museum of Anthropology of Moscow State University. According to physical anthropologists, skulls from Igren' 8 are dolichocranial with large cerebral parts and average height and width of the face (Konduktorova, 1973). According to Potekhina (1999), two morphological features are characteristic of Ukrainian Eneolithic remains: 1) a massive hypermorphic feature and 2) a gracile mesomorphic feature. Both features are present in the craniological series of Igren' 8. It was therefore suggested that the gracile craniological complex in the population associated with this culture might be the result of admixture with representatives of the earlier Trypolia culture (Potekhina, 1999).

References:

Dobrovolsky A. V. 1949. Eighth Igrenian Neolithic site. Archaeology of the Dnepr. No 2. P. 243–252. (in Ukrainian).

Konduktorova T. S. 1973. Anthropology of the Ukrainian population of the Mesolithic, Neolithic and Bronze Ages. M.: Nauka. 128 p. (in Russian).

Potekhina I. D. 1999. Population of Ukraine in the Neolithic and Early Eneolithic according to anthropological data. Kiev: Institute of Archeology of the National Academy of Sciences of Ukraine. (in Russian).

Telegin D. Ya. 1973. Middle Stog culture of the Copper Age. Kiev. 168 p. (in Ukrainian).

Karavaikha, Russia

Alexandra Buzhilova

Analysed individuals:

- KVH001 (8622, burial 6): 8416-8338 calBP
- KVH002 (8623, burial 7): 8393-8208 calBP

The site of Karavaikha was discovered on the right bank of the Eloma River, which flows from the west into Lake Vozhe (Kirillovsky District, Vologda Region). Since 1938, Bryusov excavated this site over seven field seasons (Bryusov, 1961). A part of the human remains is housed in the Museum of Anthropology of Moscow State University. Remains of only adult individuals were found at the site, most of them buried on their backs with the head oriented to the south. In two cases, the remains were in a sitting position (one of them is sample KVH002, burial 7), and one individual was buried on the right side. All burials were single, except for grave No. 2, in which there were remains of two individuals. Part of the skeletons was covered by ochre. In the initial years of the excavation, Bryusov

established the chronological sequence in the burial complex of Karavaikha, dating it from the late Mesolithic to the Eneolithic (Bryusov, 1961). This was partially confirmed by radiocarbon dating attempts (burial 1 - 8200 ± 50 GIN-7173, burial 6 - 4420 ± 50 GIN-7172 and burial 7 - 6880 ± 90 GIN-7176) (Utkin, Kostyleva, 2001). Bio-archeological studies have shown morphological heterogeneity of the human remains, which could be associated with the use of the burial complex for an extended time. Moreover, a mixture of craniological features was observed (Akimova, 1953; Gerasimov, 1955).

References:

Akimova M. S. 1953. New paleoanthropological finds of the Neolithic era in the forest zone of the European part of the USSR. Brief Communications of the Institute of Ethnography of the USSR. Vol. 18. P. 55-65. (In Russian).
Bryusov A. Ya. 1961. The Karavaikha site. In: The archeology of the Vologda region. Ed. Bryusov A. Ya. Vologda. P. 72-95. (In Russian).
Gerasimov M. M. 1955. Facial restoration through the skull: a modern and fossilised person. Proceedings of the Institute of Ethnography (new series). Vol. 28. 558 p. (In Russian).
Utkin A. V., Kostyleva E. L. 2001. Burials in the site of Karavaikha. Russian archaeology. No. 3. P. 55-66. (In Russian).

Krzyż, Poland

Jacek Kabacinski, Thomas Terberger, Bettina Jungklaus

Analyzed individual:

- KRZ001 (KRZYZ 7): 10221-9906 calBP (layer)

The Krzyż site is located on the lower terrace of the northern edge of the Noteć River in southern Pomerania in the district of Czarnków-Trzcianka (voivodeship Wielkopolska, Poland). In 2003, the discovery of Mesolithic antler and bone tools led to the beginning of archeological excavations at the site, which are still ongoing. Fieldwork uncovered parts of the settlement and a dump layer in a paleochannel of the Noteć River. Moreover, remains of multiple settlements were found including thousands of flint artefacts and bone and antler remains such as tools, composite tools, wooden, plant and bark objects (Kabaciński et al., 2008, 2011). The two oldest radiocarbon dates on faunal remains place the initial occupation of the site around the mid of the Preboreal period (between ~10,800 and 10,500 calBP; Poz-34354: 9,430 ± 50 BP; Poz-34352: 9,320 ± 50 BP), while the date on charcoal extracted from the profile dates the uppermost find layer around the end of the Boreal period (~9,200 calBP; Poz-27418: 8,210 ± 40 BP) (Kabaciński et al., in press). In 2018, a human skull was found 1.56-1.78 m below the surface (26.58-26.80 m a.s.l.; Kabaciński et al., in prep.). The skull was deposited in a fine detritus gyttja overlying bottom sands and gravels of the Noteć river paleochannel. It was lying directly on a ~40 cm long split of pine wood and was associated with characteristic Mesolithic artefacts, including a red deer antler axe with a fragment of a wooden shaft. A direct AMS-date on the skull resulted in ~10,900 calBP (MAMS-40653: 9,548 ± 26 BP). A second date was measured on the fragment of pine split directly underlying the skull. The date of 10,100 calBP (Poz-110982: 8,940 ± 50 BP) is considerably younger than the date of the skull. The age difference of ~800 years might, at least partially, be explained by a considerable reservoir effect in the human specimen. The ^{15}N-value of 12.4 ‰ for the skull (^{13}C: -23.9 ‰) is only moderately elevated. Dates on fish from the Noteć River, however, point to a modern reservoir effect of ~500-700 years. Moreover, the same

age difference was observed during the dating of fish bones and hazelnut shells discovered in the Boreal find layer. Estimating a reservoir effect of ~300 years, the skull might be dated to ~10,600 calBP. The isolated finding of the skull in the dump layer and its rather weathered surface might be due to a burial rite where the dead body was deposited somewhere else before arriving at the find location. Only the back of the skull is preserved including the *Ossa parietalia* and the *Os occipitale*. The skull is gracile and the ossified *sutura sagittalis* as well as the *sutura lambdoidea* suggest an age at death of 40-60 years. No pathological features were identified.

References:
Kabaciński, J., B. Jungklaus, M. Mannino, B. Fuller, M. Litynska-Zajac, I. Okuniewska-Nowaczyk, C. Posth, J. Mugaj, T. Terberger, The Early Holocene human skull from Krzyż Wielkopolski, Western Poland (in prep.).
Kabaciński, J., A. Henry, E. David, M. Rageot, C. Cheval, M. Winiarska-Kabacińska, M. Regert, A. Mazuy, F. Orange, Expedient and efficient: an Early Mesolithic composite implement from Krzyż Wielkopolski. *Antiquity*, in press.
Kabaciński J., S. Hartz, T. Terberger, Elks in the early Stone Age art of the northern Lowlands. *Prähistorische Zeitschrift*, 86 (2), 2011, 151-164.
Kabaciński J., E. David, D. Makowiecki, R. Schild, I. Sobkowiak-Tabaka, M. Winiarska-Kabacińska, Stanowisko mezolityczne z okresu borealnego w Krzyżu Wielkopolskim. *Archeologia Polski*, LIII (2), 2008, 243-288.

La Marche, France

Gildas Merceron, Géraldine Garcia, Jean-Michel Leuvrey, Coralie Bay-Garcia

Analyzed individual:
- LMA001 (LMR-B113-115): 18223-17908 calBP

First reported in 1914 but excavated only from 1937 onwards (Péricard and Lwoff, 1940), the cave of La Marche located along the Vienne River (Lussac-les-Châteaux, department of Vienne, Western France) is known for its exceptional collection of engraved blocks (more than 4,000) with human and animal representations attributed to the Middle Magdalenian culture (e.g., Airvaux and Pradel, 1984; Chisena and Delage, 2018; Fuentes, 2010; Gaudron, 1942; Lwoff, 1941; Mélard, 2008; Pales and de Saint Péreuse, 1969). Since then, excavators removed a layer containing dental and bone remains as well as a large number of lithic artifacts. The backdirt from this excavation remained in the cave until a second team collected it in the 1990s. This material makes up the so-called "Bastière" collection stored at the Museums of Poitiers (France) under the single number « inv. 999.3.1 ». From the excavations conducted in the 1990s, a parietal, four mandibular fragments as well as numerous isolated teeth (n = 35) from humans of different ages were found (Henry-Gambier, 2010; Le Luyer et al., 2019; 2021). From the 500-kg of material from the "Bastière" collection, Garcia et al. (2018) have sorted off more than a thousand of identifiable bone and dental remains mostly of mammals. Pradel (1959) had identified at the cave of La Marche eight taxa of mammals including carnivores (*Ursus*, *Canis*, *Meles*), ruminants (Large bovine, *Saiga*, *Rangifer*, *Cervus*) and horses (*Equus*). Garcia et al., (2018) completed the faunal list with at least seven new taxa including Phocidae (cf. *Halichoerus*), *Vulpes*, *Crocuta*, *Panthera* and *Felis* among carnivores, *Capra*, *Sus* and *Castor*. Several shell fragments of European pond turtles were also found. Garcia et al. (2018) have also identified 16 human dental remains including deciduous molars and permanent incisors, premolars and molars with

different wear rates indicating a minimum number of three individuals. The tooth from the cave of La Marche analyzed in the present study belongs to the "Bastière" « inv. 999.3.1 » collection. This is a deciduous upper right second molar (LMR-B113-115). In addition, Garcia et al. (2018) have identified more than 30 human bone remains including phalanges, carpals and metacarpals, tarsals and metatarsals, a zygomatic bone, fragments of femur, maxilla, mandibles and hyoid bones (Garcia et al., 2018). Due to the origin of this material with no stratigraphic context, Merceron and Garcia (2020) ran radiocarbon dating (Beta Analytic Lab) on 15 mammal teeth including specimens potentially out-of-context, notably those assigned to fossorial species (badgers, foxes) that may have disrupted the stratigraphic context as well as a series of bovid teeth that may be assigned to livestock from historical periods. Eleven teeth had enough collagen preserved to provide a radiocarbon date. Two incisors assigned to a large caninae, one incisor assigned to a vulpin, and a lower third molar of a horse provided ^{14}C ages ranging from 15,450 to 14,850 BP and thus calendar ages ranging from 17,800 to 18,900 calBP. It is worth noting that a second lower third molar of horse provided a ^{14}C age of ~30,000 BP; such earlier occurrences are not unexpected as the caves along the Vienne River have known human occupations through the whole Late Pleistocene. With the exception of a single Solutrean flint (Leuvrey, 2015), there are no other lithic remains found that could be assigned to human populations older than the Magdalenian ones. One tooth of a large bovinae and two teeth of small caprinae confirm the use of the cave for livestock by local populations from at least the late Middle Age until the late 19th century. Three incisors of badgers and foxes confirm that fossorial species have disrupted the stratigraphy of the cave deposits at least since the end of the Pleistocene onwards. The radiocarbon dating produced by Merceron and Garcia (2020) reveals an early Middle Magdalenian period for the main assemblage, which confirms earlier dating attempts on four horse teeth (^{14}C ages from 14,870 to 14,560 BP; Brou et al., 2013) and on a human tooth (^{14}C age 14,685 ± 75 BP; OxA-30980; Barshay-Szmidt et al., 2016).

References:

Airvaux, J., Pradel, L., 1984. Gravure d'une tête humaine de face dans le Magdalénien III de La Marche, commune de Lussac-les-Châteaux (Vienne). Bull. Soc. Préh. Fr. 81, 212–215.

Barshay-Szmidt, C., Costamagno, S., Henry-Gambier, D., Laroulandie, V., Pétillon, J.-M., Boudadi-Maligne, M., Kuntz, D., Langlais, M., Mallye, J.-B., 2016. New extensive focused AMS 14C dating of the Middle and Upper Magdalenian of the western Aquitaine/Pyrenean region of France (ca. 19–14 ka cal BP): Proposing a new model for its chronological phases and for the timing of occupation. Quat. Int. 414, 62–91.

Brou, L., Primault, J., Airvaux, J., 2013. Chronologie absolue du Magdalénien en Poitou-Charentes (Projet Collectif de Recherche, SRA Poitou-Charentes).

Chisena, S., Delage, C., 2018. On the Attribution of Palaeolithic Artworks: The Case of La Marche (Lussac-les-Châteaux, Vienne). Open Archaeol. 4, 239–261.

Fuentes, O., 2010. Les représentations humaines au Magdalénien en Poitou-Charentes, in: Préhistoire entre Vienne et Charente : Hommes et Sociétés du Paléolithique. Chauvigny, Association des publications chauvinoises, 383–396.

Garcia, G., Fournel, M., Daver, G., Guy, F., Leuvrey, J.-M., Magniez, C., Fleury, J., Valentin, X., Merceron, G., 2018. New paleontological data from the La Marche Cave (upper Pleistocene, Lussac-Les-Châteaux, Vienne), in: Colloque Annuel de La Société d'Anthropologie de Paris. Poitiers, France.

Gaudron, G., 1942. Éléments d'une méthode destinée à reproduire par photographie directe des plaquettes gravées, comme celles de la grotte de la Marche, à Lussac-les-Châteaux. Bull. Soc. Préh. Fr. 39, 99–101.

Henry-Gambier, D., 2010. Les fossiles humains du Paléolithique supérieur de Poitou-Charentes, in: Préhistoire entre Vienne et Charente : Hommes et Sociétés du Paléolithique. Chauvigny, Association des publications chauvinoises, 25–43.
Leuvrey, J.-M., 2015. Inventaire de la collection Bastière, La Marche (Lussac les Chateaux, Vienne, France). Musées de Poitiers (unpublished document, in French).
Le Luyer, M., Airvaux, J., Henry-Gambier, D., 2019. New human remains associated with Magdalenian rock art from La Marche cave (Lussac-Les-Châteaux, France). Presented at the European Society of Human Evolution, p. 106.
Le Luyer, M., Airvaux, J., Henry-Gambier, D., 2021. Les dents humaines magdaléniennes de la grotte de La Marche (Lussac-Les-Châteaux, Vienne, France). PALEO 31, 158-186.
Lwoff, S., 1941. Gravures à représentations d'humains du Magdalénien III. Fouilles de La Marche, commune de Lussac-les-Châteaux (Vienne). Bull. Soc. Préh. Fr. 38, 145–161.
Mélard, N., 2008. Pierres gravées de la Marche à Lussac-les-Châteaux (Vienne): techniques, technologie et interprétations. Gallia Préhistoire 50, 143–268.
Merceron, G., Garcia, G., 2020. Rapports de Fouilles « La Marche : Datations pour des données fauniques et anthropologiques inédites». Service Régional d'Archéologie, Direction Régionale des Affaires Culturelles de Nouvelle Aquitaine, Poitiers (unpublished annual report, in French).
Pales, L., de Saint Péreuse, M. T., 1969. Les gravures de la Marche. Ophrys.
Péricard, L., Lwoff, S., 1940. LA MARCHE: Commune de Lussac-les-Châteaux (Vienne) Premier atelier de Magdalénien III à dalles gravées mobiles: Campagnes de fouilles 1937-1938. Bull. Soc. Préh. Fr. 37, 155–180.
Pradel, L., 1959. La grotte magdalénienne de la Marche. Commune de Lussac-les-Chateaux (Vienne). Mém. Soc. Préh. Fr. 5, 170–191.

La Riera, Spain

Lawrence Guy Straus, Geoffrey Clark

Analyzed individual:

- RIE002 (Level 14): 21011-20725 calBP

Located at c. 30 m above present sea level on the narrow coastal plain near the foot of a dramatic, ±1000 m-high mountain range, midway between Santander and Oviedo, in Posada de Llanes, and 1.75 km from the present shore of the Bay of Biscay, La Riera is a small cave that is part of a major cluster of Paleolithic and Mesolithic sites in eastern Asturias that was discovered and excavated by local archeologist Count Vega del Sella in the early 20th century. It was at this site in 1917-18 that he correctly defined the chronological position of regional shell middens, which he assigned to the Asturian culture above a long sequence of Upper Paleolithic layers (Vega del Sella 1930). Repeatedly looted in subsequent years, part of the remaining intact archeological deposit in La Riera was the object of an intensive, international, interdisciplinary project directed by Clark and Straus in 1976-79, resulting in numerous articles, a monograph (Straus and Clark, 1986), and many restudies of specific materials by both Spanish and foreign archeologists and natural scientists. The project was, among other things, a pioneer in the Spanish context in the major application of radiocarbon dating for the establishment of absolute chronology in the Upper Paleolithic and Mesolithic, albeit with several problems of stratigraphic coherence and assay reliability. The sequence that was revealed includes Early Upper Paleolithic, Solutrean, Lower and Upper Magdalenian, Azilian and Asturian levels. Together with the adjacent site of Cueto de la Mina, La Riera contains one of the longest, most

important Solutrean sequences in Cantabrian Spain, corresponding to the Last Glacial Maximum, a time when northwestern Europe was at least mainly abandoned and the human range had retreated to refugia in southern France and Iberia (Straus 1991a,b; 2000; 2015). Distinctive Solutrean points (laurel and willow leaves, unifacial, shouldered and concave base points) were found in Levels 2,3,4,5,6,7,8,9,10,14,15 and 17. However these temporally/culturally diagnostic artifacts are far more abundant in the layers below Level 8 than in Levels 8-17, perhaps because of activity/functional differences and/or the vicissitudes of archeological sampling (i.e., where the excavation happened by accident to fall relative to spatial distributions of different artifact types in successive occupations of the cave space). Level 14 was originally dated by the conventional radiocarbon method on bone - likely with errors - to 15,690±310 uncal BP and later by A. Craighead (1999) on mollusk shell to 16,410±105 uncal BP (with a marine reservoir effect estimated at -400 years, which is far greater than the c. -100 years of Soares et al. [2016]). However, underlying Level 12 was also conventionally dated on charcoal to 17,210±350 uncal BP and overlying Levels 15, 16 and 17 were respectively dated to 17,225±350, 18,220±610, and (with two dates) 17,070±230 and 16,090±200 (with another likely erroneous date of 15,600±570 uncal BP for Level 15). With its earliest Solutrean-point-bearing levels dating to 21-20 uncal ky BP (as confirmed by a series of new bone and shell ^{14}C dates done with ultrafiltration (Soares et al., 2016), La Riera has one of the longest and most complete Solutrean sequences in Spain. Level 14, along with several other Solutrean-point-bearing sites in the Vasco-Cantabrian region (e.g, Las Caldas, Chufín, Cova Rosa, Altamira, El Ruso, Arlanpe), was thought to date to about 17-17.5 uncal ky BP—the end of this culture-historical period or techno-complex, as attested by the beginning of the Initial Magdalenian in El Mirón cave (Cantabria) shortly thereafter (Hopkins et al., 2021). The new AMS date on the Level 14 human cranial bone fragment whose DNA is presented in this article is 17,300±50 uncal BP (21,002-20,766 cal BP at 95% probability—MAMS-51979), confirms this and is fully consonant with expectations for the late Solutrean in Cantabrian Spain. Level 14, like other Solutrean levels in La Riera, was characterized by massively specialized hunting of red deer, with smaller amounts of ibex, supplemented by limpets and trout, at a time when the shore was about 10 km distant.

References:

Craighead, A. 1999. Climate change and patterns in the exploitation of economic resources (marine Mollusca and ungulate fauna) in Cantabrian Spain at the end of the Pleistocene, ca.21-6.5 kyr BP. In J. Driver, ed., Zooarchaeology of the Pleistocene/Holocene Boundary. British Archaeological Reports S-800, Oxford, pp. 9-20.
Hopkins, R.J.A., Straus, L.G., González Morales, M.R. 2021. Assessing the chronostratigraphy of El Mirón Cave, Cantabrian Spain. Radiocarbon 63, 821-852. doi.1017/RDC.2020.121.
Monge Soares, A., Gutiérrez-Zugasti, I., González-Morales, M., Matos Martins, J., Cuenca-Solana, D., Bailey, G. 2016. Marine radiocarbon reservoir effect in Late Pleistocene and Early Holocene coastal waters off northern Iberia. Radiocarbon 58, 869-883. doi.10.1017/RDC.2016.71.
Straus, L.G. 1991a. SW Europe at the Last Glacial Maximum. Current Anthropology 32,189-199.
Straus, L.G. 1991b. Human Geography of the Late Upper Paleolithic in Western Europe: Present State of the Question. Journal of Anthropological Research 47, 259-278.
Straus, L.G. 2000. A quarter-century of research on the Solutrean of Vasco-Cantabria*,* Iberia *&* beyond*.* Journal of Anthropological Research 56, 39-58.
Straus, L.G. 2015. The human occupation of southwestern Europe during the Last Glacial Maximum: Solutrean cultural adaptations in France and Iberia. Journal of Anthropological Research 71, 465-492.
Straus, L.G., Clark, G.A. 1986. La Riera Cave. Anthropological Research Papers 36, Tempe, Arizona.

La Rochette, France

Katerina Harvati

Analyzed individual:

- LRO001 (OSUT 7074): 27834-27440 calBP

Archeological description previously published in Posth et al., 2016.

Le Piage, France

Hélène Rougier, Priscilla Bayle, Mona Le Luyer, Bruno Maureille, Foni Le Brun-Ricalens, Jean-Guillaume Bordes

Analyzed individual:

- LPI002 (PG08 J-1D α(2) #27): 23757-23193 calBP

Le Piage (Fajoles, France) is located at the foot of a Coniacian limestone cliff containing numerous karstic cavities. The very sandy sedimentation is conditioned by both an important spring still active and the limestone disintegration. Excavated between 1958 and 1968, and since 2004, the deposit has yielded Mousterian, Châtelperronian, Protoaurignacian, Early and Late Aurignacian, Solutrean, and Badegoulian technocomplexes (Champagne and Espitalié, 1981; Bordes et al., 2008). The latter two are intimately mixed by solifluction. The level that yielded the individual analyzed here produced very rich lithic and bone industries, as well as adornment. The fauna is ultra-dominated by reindeers. The tooth that was sampled for aDNA is an upper permanent incisor (PG08 J-1D α(2) #27). Along with several other teeth and cranial specimens, it represents a young adult or late adolescent (Le Piage II) directly dated to the Last Glacial Maximum (Rougier et al., in prep.).

References:

Bordes, J.-G., Le Brun-Ricalens, F., Castel, J.-C., Ducasse, S., Faivre, J.-P., Feruglio, V., Henry-Gambier, D., Lacrampe-Cuyaubère, F., Laroulandie, V., Lenoble, A., Martin, H., Maureille, B., Morala, A., Morin, E., Renard, C., Rendu, W., Rigaud, S., Rougier, H., Szmidt, C., Tartar, E., Texier, J.-P., Teyssandier, N., 2008. Les débuts du Paléolithique supérieur dans le Sud-Ouest de la France : nouvelles fouilles au Piage (2004-2006). Problématique et premiers résultats. *In*: Jaubert, J., Bordes, J.-G., Ortega, I. (Dir.), *Les Sociétés du Paléolithique dans un Grand Sud-Ouest de la France : nouveaux gisements, nouveaux résultats, nouvelles méthodes*. Journées SPF, Université Bordeaux 1, Talence - 24-25 nov. 2006. Mémoires de la Société préhistorique française 47, pp. 261-288.
Champagne, F., Espitalié, R., 1981. *Le Piage, site préhistorique du Lot*. Mémoires de la Société préhistorique française 15, 205 p.
Rougier, H., Bayle, P., Le Luyer, M., Maureille, B., Barshay-Szmidt, C., Castel, J.-C., Ducasse, S., Morin, E., Renard, C., Texier, J.-P., Le Brun-Ricalens, F., Bordes, J.-G., in prep. The first directly dated Solutrean human fossil. Journal of Human Evolution.

Maisons-Alfort, France

Frédérique Valentin, Richard Cottiaux

Analyzed individual:

- MAF001 (ZAC Alfort Str 7): 9078-8651 calBP

The burial site (ALF III - Structure 7) at Maisons-Alfort in the Val-de-Marne is an isolated burial site. It was discovered during a rescue excavation carried out in 1999 by the *Institut National de Recherches Archéologiques Préventives* (France) at "Zac d'Alfort", at the confluence of the Marne and the Seine rivers (Cottiaux et al., 2002). The skeleton, probably belonging to an adult individual, is poorly preserved and morphological sex could not be assigned. The individual was buried in a pit dug in brown sandy silt with poorly visible boundaries and the pit's bottom was lined with stones collected in the surrounding environment. The individual was placed in a left lateral decubitus position, with a strongly constrained position (hyperflexed) where the feet and pelvis were brought into contact (Valentin et al., 2008). The original position of the upper body could not be reconstructed, but it appears that the burial had an east/west orientation, with the head towards the west. Two laminar flakes and a fragment of a small blade were found near the skeleton. The burial was directly radiocarbon dated to ~8,900 calBP (LY-9817: 8030 ± 50 BP) and assigned to the Middle-Late Mesolithic period of Western Europe.

References:

Cottiaux R., Casadei D., Chaussee C., Delattre V., Hachem L., Martial E. (2002) *Maisons-alfort ZAC d'Alfort (Val-de-Marne), sauvetages urgents 1998-2001*, Document Final de Synthèse, Saint-Denis, 2 vol., 133 p., 165 fig.

Valentin F., Cottiaux R., Buquet-Marcon C. Confalonieri J., Delattre V., Lang L., Le Goff I., Lawrence-Dubovac P., Verjux C. (2008) Découvertes récentes d'inhumations et d'incinération datées du Mésolithique en Ile de France. *Revue Archéologique d'Ile-de-France*, 1 : 21-42.

Malonne Petit Ri, Belgium

Patrick Semal, Ivan Jadin

Analyzed individual:

- MPR001 (MPR-1): 10681-10244 calBP

The site of Petit Ri, located in Malonne near Namur (Province of Namur) was discovered in 1962 by Michel Charpentier. These are the remains of a cave partially destroyed by quarrying (Jadin & Carpentier, 1994). The remains of several individuals were found (Twiesselmann & Orban, 1994) as well as several lithics (Eloy & Jadin, 1994) and fauna remains (Cordy, 1994). Anthropological analysis shows that based on fibula skeletal elements the minimum number of individuals is four (Twiesselmann & Orban, 1994). However, the majority of the bones discovered may correspond to a single individual. The association between the infracranial bones and the skull is possible but cannot be established with certainty due to the discovery conditions. The skull has a robust morphology and displays archaic features while the infracranial skeleton is gracile (Twiesselmann & Orban, 1994). The radiocarbon dating (OxA-5042) of the right femur provided an age of 9270 ± 90 BP, which places it within the dates obtained for Early Mesolithic burials in the region (Jadin & Carpentier, 1994). Direct dating of the skull was not carried out as the skull was varnished and this may result in an artificially younger radiocarbon date. Stable isotopes suggest that terrestrial mammals were the

main source of dietary proteins (Bocherens et al., 2007). The material belongs to the collections of the Royal Belgian Institute of Natural Sciences in Brussels.

References:
Bocherens, H., Polet, C. & Toussaint, M. Palaeodiet of Mesolithic and Neolithic populations of Meuse Basin (Belgium): Evidence from stable isotopes. *Journal of Archaeological Science***34**, 10–27 (2007).
Cordy, J.-M. Étude de la faune de la Grotte du Petit Ri à Malonne. *Anthropologie et Préhistoire* **105**, 87–91 (1994).
Éloy, L. & Jadin, I. L'industrie lithique de Petit Ri à Malonne (Namur, Belgique). *Anthropologie et Préhistoire* **105**, 83–86 (1994).
Jadin, I. & Carpentier, M. La sépulture mésolithique du Petit Ri à Malonne (Namur, Belgique) - Contexte archéologique et position chronologique. *Anthropologie et Préhistoire* **105**, 65–82 (1994).
Twiesselmann, F. & Orban, R. Ossements humains découverts dans le massif rocheux du Petit Ri à Malonne (province de Namur, Belgique). *Anthropologie et Préhistoire* **105**, 93–125 (1994).

Maszycka, Poland

Marta Poltowicz-Bobak, Dariusz Bobak, Bettina Jungklaus, Thomas Terberger

Analyzed individuals:

- MAZ001 (1/1): 18586-18184 calBP
- MAZ003 (1/5): 15754-15357 calBP

The Maszycka cave is located on the left slope of the Pradnik valley in the Krakow district of southwestern Poland. Fieldwork at this site started in 1883 when the cave and a smaller part of the terrace were investigated (~60 m^2). In the 1960s, fieldwork outside of the cave by St. K. Kozłowski identified a colluvial loess layer, which contained Upper Paleolithic find material. The find layer was clearly separated from the top most layer with Neolithic and Bronze Age remains (Kozłowski et al., 1995; 2012). Around 290 stone artefacts and ~360 taxonomically identified faunal remains can be reported for the Upper Paleolithic assemblage. The faunal remains are dominated by horse, followed by reindeer, red deer, bovines, saiga and bear. Some bones of rhinoceros might indicate hunting of this large herbivore, while mammoth is only represented by some ivory. The processing of bone, antler and ivory on the site is demonstrated by some half-finished products and tools. Points of different types and sizes are present. Many of the pieces are decorated by geometric ornamentation and grooved sagaies that resemble examples from the Magdalenian in western Europe. This is supported by eight so-called navettes and their fragments most typical for the *Magdalénien à navettes* from France. A polished perforated reindeer antler with phallus-like sculptured ends and additional decoration is another characteristic feature of that Magdalenian phase. An additional important find is a ~40 cm long rib with slots along both edges probably used for hafting (backed) bladelets. The object can be interpreted as a prestigious dagger. Since the 19th century, the Maszycka cultural remains have been connected to the Magdalenian and in the 1980s the assemblage was more precisely compared with the French "Magdalénien à navettes" facies (Allain et al., 1985, Kozłowski et al., 1995). Recently, two radiocarbon dates on faunal remains and two on human remains assigned the site to ~18,300 calBP (oldest date KIA 39228: 15,155 ±60 BP, youngest date KIA 39225: 14,855 ±60 BP; Kozłowski et al., 2012). These results fit properly with the dates of this Magdalenian phase in western Europe. Archeological remains show also apparent connections to eastern assemblages of that time

(Kozłowski et al., 1995) and, accordingly, a Late Glacial re-colonization of central-eastern Europe from eastern refugia has been suggested (Maier 2015). A total of 50 human skeletal fragments were detected in the entrance area and in front of the cave during the early excavation phase (36 pieces) and fieldwork in the 1960s (14 pieces) (Orschiedt et al., 2017). The material is believed to originate from the Upper Paleolithic layer, but only the remains found during the last excavation period can be ascribed more reliably to this layer. The fragmentary material allowed the reconstruction of a minimum number of nine individuals (four adults and five children; Orschiedt et al., 2017). Most fragments (43 pieces) derive from skulls and some elements carry traces of manipulation fitting in the general pattern observed for Magdalenian human remains.

References:
Allain, J. / R. Desbrosse / J.K. Kozłowski / A. Rigaud, Le Magdalénien à navettes. Gallia Préhistoire 28, 1985, 37-124.
Kozłowski, S.K. / E. Sachse- Kozłowska / A. Marshack / T. Madeyska / H. Kierdorf / A. Lasota-Moskalewska / G. Jakubowski / M. Winiarska-Kabacinska / Z. Kapica / A. Wiercinski, Maszycka Cave, a Magdalenian site in Southern Poland. Jahrbuch des Römisch-Germanischen Zentralmuseums Mainz 40, 1993, 115-252.
Kozłowski, S.K. / M. Połtowicz-Bobak / D. Bobak / T. Terberger, New information from Maszycka Cave and the Late Glacial recolonisation of Central Europe. Quaternary International 272-273, 2012, 288-296.
Maier, A., The Central European Magdalenian: Regional Diversity and Internal Variability (Dordrecht, 2015).
Orschiedt, J. / T. Schüler / M. Poltowicz-Bobak / D. Bobak / S.K. Kozlowski / T. Terberger, Human remains from Maszycka Cave, Poland: The treatment of human bodies in the Magdalenian. Archäologisches Korrespondenzblatt 47, 2017, 423-439.

Minino I and Minino II, Russia

Alexandra Buzhilova, Alexander Suvorov

Analysed individuals:

- MNN001.002 (2/1 ind): 8597-8457 calBP
- MNN003 (5): 7616-7505 calBP
- MNN004 (13): 7664-7516 calBP
- MNN005 (19/1 ind): 10646-10381 calBP
- MNN006 (20): 10747-10576 calBP
- MNN007 (22/2 ind): 11089-10726 calBP
- MN2001 (I/ 1 ind): 10660-10429 calBP
- MN2002 (V): 10749-10576 calBP
- MN2003 (VI/ 1 ind): 10654-10413 calBP

The archaeological sites Minino I (MNN) and Minino II (MN2) are located 0.5–0.8 km northeast of the Minino village in the Vologda region (Russia). They were found on the left bank of the Dmitrovka River, 0.4–0.7 km from its confluence with Lake Kubenskoye, which is part of the Sukhona - Severnaya Dvina - White Sea basin. The sites are located on a floodplain terrace close to the lake. The cultural layer has a thickness of 0.3 to 1.0 m and dates from the Middle Mesolithic to the 13th century AD. Prehistoric burials at the Minino I site were investigated in the eastern part of the site in an

exploration pit in 1993 and in excavation site 1 between 1996 and 2000. Prehistoric burials at the Minino II site were investigated in excavation site 3 between 2000 and 2005. Most of the graves are located in a shallow pit corresponding to the size of the buried body, dug in the underlying cultural layer or slightly deeper. Single burials are most common, but there are also multiple burials with two and three individuals as well as partial burials. The retrieved prehistoric graves do not belong to a single burial period. The majority of objects date to a wide chronological span from the second part of the Mesolithic to the Early Neolithic, and radiocarbon dating confirmed this time interval (dates from AAR, GIN, OxA and UBA dating labs) (Wood et al., 2013). Some burial goods parallel those associated with the Veret'e and Butovo archaeological cultures (Suvorov & Vasilieva, 2003). An accompanying inventory is often missing but a few randomly distributed flint and bone tools were found in some of the graves. Only burial 19 (170 artefacts) and burial 20 (271 artefacts) are distinguished by a much higher number of artefacts. The associated pendants were made of animal teeth and perforated squares of animal and fish bones, and appear to have been sewn onto the costume, allowing the reconstruction of headdresses, clothes and possibly even a bag (e.g. in grave 20 of Minino I) (Buzhilova, Suvorov, Krylovich, 2008). The human remains are housed at the Institute of Archaeology, Russian Academy of Sciences in Moscow. A total of 39 individuals were morphologically examined, focusing mainly the remains of adult individuals. The reconstruction of the physical activity shows an overall pattern of occupational stress similar to what observed in other hunter-gatherers. Neither pathological markers nor indicators of dental diseases were found, with the exception of enamel hypoplasia. Craniological analyses show analogies between the male individuals from Minino I and II and synchronous populations from northern-eastern Europe such as a group of individuals from Yuzhniy Oleniy Ostrov (burials 139, 27, 131, 128 and 76), Popovo (burial 3) and Peschanitsa. The oldest individuals from Minino I and II with analysed crania (burials 19/1 and V) are morphologically closest to Peschanitsa and older Paleolithic hunter-gatherers (Buzhilova, 2016).

References:
Buzhilova A.P., Suvorov A.V., Krylovich O.A. On the issue of reconstruction of the way of life of the population of the late Stone Age (based on the materials of the Minino archaeological complex on the Kubenskoye Lake) // *KSIA*. 2008. No. 222, P. 1–18. (in Russian).
Buzhilova A.P. A reconstruction of the lifestyle of early humans by natural-science methods // *Herald of the Russian Academy of Sciences*. 2016. Vol. 86, no. 4. P. 297–304.
Suvorov A.V., Vasilieva N.B. Two insert tools from the burial of the III monument of Minino II on the Kubenskoye lake // St. Petersburg trasological school and the study of ancient cultures of Eurasia: In honour of the anniversary of G.F. Korobkova. - SPb. 2003. P. 287–292. (in Russian).
Wood R.E., Higham T.F., Buzhilova A., Suvorov A., Heinemeier J., Olsen J. Freshwater radiocarbon reservoir effects at the burial ground of Minino, Northwest Russia. *Radiocarbon*. 2013. Vol. 55, no. 1. P. 163–177.

Mollet III and Reclau Viver (Serinyà caves), Spain

Joaquim Soler Subils, Neus Coromina, Isaac Rufí, Dorothée Drucker

Analyzed individuals:
- GER002 (RVS T4 330-350 (RVS-H-1)) : 26355-25939 calBP
- GER003 (MIII M17 QM84 54): 27322-27076 calBP

The sites of Reclau Viver and Mollet III are located in the vicinity of Serinyà (Girona, Catalonia), in a place known as Paratge del Reclau. This is a 200 meters long travertine cliff that comprises several neighbouring prehistoric sites (Reclau Viver, Pau, Mollet I, Mollet III, Arbreda, Cau del Roure, among other minor sites), which were discovered and partially excavated between 1940 and 1973 by the medical doctor Josep Maria Corominas and since then by the University of Girona. Together they provide a continuous record of the cultural and environmental evolution in this area of the western Mediterranean from the late Middle Pleistocene until the Bronze Age.

Reclau Viver was totally excavated by Corominas between 1943 and 1948. The site provided the first and one of the major sequences of the Upper Paleolithic in Catalonia. It comprises Early Aurignacian (levels A and B), Iberian Middle Gravettian (C), Final Gravettian (D), Protosolutrean (E) and Solutrean (F). A stretch from the Neolithic and Bronze Age, mixed with the blocks of the collapsed roof, seals the site (Soler, 1986). In 1948, Corominas discovered several human remains while excavating the Final Gravettian level (D) with scarce evidence on the funerary practices, although the same level provided a large amount of personal ornaments (Soler and Soler, 2013). This Final Gravettian level also revealed a rich ensemble of bone remains, stone and bone artefacts, which denotes different uses of this cave during the same period. The human remains discovered by Corominas were forgotten during the following decades until recent research has allowed identifying them among the collection of the Archaeological Museum of Banyoles. Several ^{14}C AMS dates frame the chronology of the whole level D between 21,900 and 19,700 uncal BP, although the funerary practices developed during a narrower time span determined by direct ^{14}C AMS dates on the human remains between 21,900 and 20,600 uncal BP (Soler and Soler, 2016; Drucker et al., 2021).

Mollet III is located between Reclau Viver and Arbreda cave in the same cliff and it was also excavated by Josep Maria Corominas. In 1972, he recovered an isolated human neurocranium from the Upper Paleolithic between layers 4 and 5. Unfortunately, its position was ambiguous, due to the fact that in that sector, layers 4 and 5 are at the interface between the Neolithic-Bronze Age and the Upper Paleolithic. After a short excavation conducted by academic archaeologists in 1973, it was concluded that the neurocranium belonged to the Bronze Age and the interest on this human remain vanished. However, the study of archaeological finds related to this fossil indicated a Gravettian chronology, which was later confirmed by a direct ^{14}C AMS date of 22,300 uncal BP (GrA-43783; Soler et al., 2013), placing the neurocranium in the Final Gravettian. This result motivated the reprise of the excavations between 2013 and 2020, which have provided a series of human remains from the same period, whose Final Gravettian chronology has also been confirmed by a direct ^{14}C AMS result (22,800 uncal BP, ETH-66710; Drucker et al., 2021).

References:

Drucker, D.G., Naito, Y.I., Coromina, N., Rufí, I., Soler, N. and Soler, J., 2021. Stable isotope evidence of human diet in Mediterranean context during the Last Glacial Maximum. Journal of Human Evolution, 154, 102967.

Soler, N., 1986. Les indústries del Paleolític Superior en el Nord de Catalunya. Ph.D. Dissertation. Universitat de Barcelona.

Soler, N. and Soler, J., 2013. The Solutrean of the Eastern Pyrenees/Le Solutréen dans les Pyrénées Orientales. Supplément à la Revue archéologique du centre de la France, 47(1), 65-74.

Soler, N. and Soler, J., 2016. The first *Homo sapiens* in Catalonia, hunters and gatherers from the old Upper Palaeolithic. Catalan Hist. Rev., 9, 9-23.

Soler, J., Soler, N., Agustí, B. and Bolus, M., 2013. The Gravettian calvaria from Mollet III cave (Serinyà, Northeastern Iberian Peninsula). Journal of Human Evolution, 65(32), 322-329.

Murzihinskij II, Russia

Andrey Chizhevsky, Aleksandr Khokhlov, Rezeda Tukhbatova

Analyzed individuals:

- MUR001 (burial 91, skeleton 1): 6530-6317 calBP (layer)
- MUR002 (burial 94, skeleton 2): 6664-6493 calBP
- MUR005 (burial 102, skeleton 2): 6530-6317 calBP (layer)
- MUR007 (burial 104): 6491-6320 calBP (layer)
- MUR017 (burial 128, skeleton 2): 6530-6317 calBP (layer)
- MUR021 (burial 154): 5896-5608 calBP

Murzihinskij II site is situated on the island Milicejskij in the Republic of Tatarstan (Alekseevskij district, Russia). Most of the burials from Murzihinskij II were dated by archaeologists as Late Bronze and Early Iron Ages. In 1995, Eneolithic burials were found on the southern side of the central part of the archeological site, 3.5-4.0 km from the modern coastal strip. The burials were distributed over three northeast-southwest oriented rows with 3-6 graves each but also between the rows in an unorganized manner. Twelve graves with the outline of the grave pit were found either with a square shape or with a round shape. The depth of burials varies between 4 and 30 cm, with an average of 11 cm. Two-thirds of the burials are collective ones and contain from two to four skeletons. Most individuals were inhumed but there were also some burned burials. In many graves ochre was found ranging from some pieces to a full grave covered with it. All skeletons were sitting or lying on the back with bent legs. Judging from the most complete skeletons, individuals were originally buried in sitting position, with half-bent legs, hands with forearms placed on their knees and the head hanging over the chest. The orientation of the lying individuals varies but the eastern direction is most common. The skeletons in collective burials were placed in two ways: 1) two to four skeletons located in one row, shoulder to shoulder and heads towards one side; 2) three skeletons with heads oriented in different directions and feet towards the centre of the grave pit. All skeletons are poorly preserved. According to the paleoanthropological analysis of A. Khokhlov, a subset of the skulls from Murzihinskij II shows a morphology similar to inhabitants of the Urals and the Middle Volga forests during the neo-Eneolithic period. The other subset is instead more similar to the European morphologies suggesting that this population might be the result of recent genetic admixture. Most of the burials contain grave goods, and some animal bones such as martens, foxes, marmot and beaver. Moreover, in one of the burials a fragment of a cattle blade was found. In two burials, bones of fossil animals were found: in burial 90 a fragment of a woolly rhinoceros tooth (*Coelodonta antiquitatis*) and in burial 118 a tooth of an aurochs (*Bos primigenius*). In addition, several wooden tools were found, arrowheads from flint and bones, two daggers, a dart tip and two axes made of soft limestone. In burial 118 between the legs of skeleton 1, a sculpted model of a phallus was found. This remain is 26.7 cm long, made from silicic limestone and similar models made of wood, horns and bones are characteristic of the Volosovo culture. The Volosovo culture is a Neolithic culture that existed from the end of the fifth millennium BP until the first half of the fourth millennium BP in the Oka River basin below Riazan' and in the lower extent of the Kliaz'ma River. The culture was described by V. A. Gorodtsov and named after a site discovered at the village of Volosovo, near Murom. The Volosovo culture is characterized by large sites with extensive mud-hut dwellings, distinctive types of flint tools, figurines made of bone and stone, and vessels made with clay mixed with shells and decorated with stamped impressions and small holes. During the fourth millennium BP, the Volosovo culture spread far to the north, for example up to the Nikolo-Perevoz site on Dubna. Two small cup-shaped vessels with a rounded bottom were found in two burials. In the clay there are plant mixtures.

These cups are decorated with comb ornament, forming zigzag lines in various combinations. Similarities with these ceramics are found in ceramic complexes of the Bor culture. In addition, the same ornamental motifs are present on the ceramics of the Yurtikovskaya culture of the Kama region. The Gari Bor culture is found on the Kama in the second half of the 7th-millennium calBP. It is thought that the Novoilyinskoye and Gari-Bor traditions were formed based on the Kama Neolithic culture. The territory presenting the Bor culture during the Eneolithic is Perm and the Volga-Kama region. In the 1950s, they were considered an integral part of the Turbino culture in the late stage of its development. Now Bor monuments are grouped in a single culture, which is characterized by archaic Neolithic ceramic and flint complexes. The time period of its existence is determined in different ways between the second half of the sixth millennium BP or the first half - middle of the sixth millennium BP. Hunting and fishing were the main sources of subsistence. Finally, most of the burial inventory finds analogies with the monuments of the Volga-Ural during the Eneolithic. Moreover, the presence of a copper ornament, cattle bones and European skull morphologies suggest a significant southern influence at the site.
In two Eneolithic individuals from the Murzihinskij II site genomic data from *S. enterica* were previously retrieved and analyzed (Key et al., 2020).

References:
Key, F.M., Posth, C., Esquivel-Gomez, L.R., Hübler, R., Spyrou, M.A., Neumann, G.U., Furtwängler, A., Sabin, S., Burri, M., Wissgott, A. and Lankapalli, A.K., 2020. Emergence of human-adapted *Salmonella enterica* is linked to the Neolithization process. *Nature ecology & evolution*, 4(3), pp. 324-333.

Oberkassel, Germany

Ralf W. Schmitz, Susanne C. Feine

Analyzed individuals:

- OKL001 (Oberkassel 2 D-999): 14081-13809 calBP
- OKL002 (Oberkassel 1 D-998): 13729-13186 calBP

In February 1914 the Late Glacial double burial of Oberkassel was discovered by quarry workers. Five years later, a comprehensive work on the find material and its context was published by Max Verworn, Robert Bonnet and Gustav Steinmann. This work is entitled "*Der diluviale Menschenfund von Obercassel bei Bonn*." According to the description of the quarry workers, the skeletons of the 35 to 45 year old male and of the 25 year old female laid at the bottom of a sand layer, with less than a meter separating the skeletons. Both human remains, the skeleton of a dog and additional grave goods were in a clearly defined area that was approximately 20 to 30 cm thick and approximately 3 meters in diameter. A few days after the discovery of the site, scientists from the University of Bonn and the institutional forerunner of the LVR-Landesmuseum Bonn carried out a test excavation on the red colored area. They were able to excavate some foot bones of the male and some bones of the dog in situ to determine the exact stratigraphic position. All finds were coated with an intense red coloration, which was created by covering the dead bodies with the mineral hematite. According to the workers, the skeletons were covered with large flat basalt blocks. Here it is especially worth noticing that the skeletons and above all the skulls remained so remarkably well preserved although they were covered by 6 meters of sediment. The basalt plates possibly reduced the direct pressure on the bodies. In additional to the well preserved human skeletons important grave goods have been discovered

including the skeleton of a young dog (Thalmann et al., 2013), an 8-cm long figurine of an elk cow made of elk antler, a 20-cm long polished bone pin adorned with an animal head, a modified penis bone of a bear and a modified red deer incisor (Giemsch et al., 2015). Since 2008 the skeletal remains and the grave goods have been the subject of a multi-disciplinary project (Giemsch & Schmitz, 2015). In 2012, a field season brought new information about the late Pleistocene sediment layers and yielded an arrowhead, the first typologically relevant stone tool from the site (Feine et al., 2015). The finds from 1914 were preserved with glue shortly after their discovery and, therefore, direct radiocarbon dating is challenging. The dating results are divided between an older group (six dates from the female and the dog with a weighted mean of 12,191±25 uncal BP) and a younger group (three dates from the male, the dog and the bear baculum with a weighted mean of 11,649±45 uncal BP). The data of the older group are considered more reliable, dating the site to a calibrated age of ~14,200 years BP, and thus during the transitional period between the Final Magdalenian and the Curved-back Point Industries (Higham et al., 2015; Street & Jöris 2015).

References:
Feine, S. C. / Giemsch, L. / Schmitz, R. W. 2015: The excavations of 1994 and 2012.- In: Giemsch, L. / Schmitz, R. W. (eds.), The Late Glacial Burial from Oberkassel Revisited.- Rheinische Ausgrabungen 72: 43-55; Darmstadt.
Giemsch, L. / Schmitz, R. W. (eds.) 2015: The Late Glacial Burial from Oberkassel Revisited.- Rheinische Ausgrabungen 72; XII + 300 p.; 230 fig.; Darmstadt.
Giemsch, L. / Tinnes, J. / Schmitz, R. W. 2015: Comparative studies of the art objects and other grave goods from Bonn-Oberkassel.- In: Giemsch, L. / Schmitz, R. W. (eds.), The Late Glacial Burial from Oberkassel Revisited.- Rheinische Ausgrabungen 72: 231-251; Darmstadt.
Higham, T. / Schmitz, R. W. / Giemsch, L. / Feine, S. C. / Street, M. 2015: Radiocarbon dating of the Oberkassel specimens.- In: Giemsch, L. / Schmitz, R. W. (eds.), The Late Glacial Burial from Oberkassel Revisited.- Rheinische Ausgrabungen 72: 63-65; Darmstadt.
Nehlich, O. / Richards, M. P. 2015: Dietary reconstruction of the two skeletons from Oberkassel by stable carbon and nitrogen isotope analysis from bone collagen.- In: Giemsch, L. / Schmitz, R. W. (eds.), The Late Glacial Burial from Oberkassel Revisited.- Rheinische Ausgrabungen 72: 219-222; Darmstadt.
Street, M. / Jöris, O. 2015: The age of the Oberkassel burial in the context of climate, environment and the late glacial settlement history of the Rhineland.- In: Giemsch, L. / Schmitz, R. W. (eds.), The Late Glacial Burial from Oberkassel Revisited.- Rheinische Ausgrabungen 72: 25-42; Darmstadt.
Thalmann, O. / Shapiro, B. / Cui, P. / Schuenemann, V. J. / Sawyer, S. K. / Greenfield, D. L. / Germonpré, M. B. / Sablin, M. V. / López-Giráldez, F. / Napierala, H. / Uerpmann, H.-P. / Loponte, D. M. / Acosta, A. A. / Giemsch, L. / Schmitz, R. W. / Worthington, B. / Buikstra, J. E. / Druzhkova, A. / Graphodatsky, A. S. / Ovodov, N. D. / Wahlberg, N. / Freedman, A. H. / Schweizer, R. M. / Koepfli, K.-P. / Leonard, J. A. / Meyer, M. / Krause, J. / Pääbo, S. / Green, R. E. / Wayne, R. K. 2013: Complete mitochondrial genomes of ancient canids suggest a European origin of domestic dogs. *Science* 342: 871-874 + Supplementary Information Online.

Ormesson, France

Pierre Bodu, Marie-Anne Julien

Analyzed individual:

- ORM001 (2988): 31835-29970 calBP (layer)

The Bossats site at Ormesson (Seine-et-Marne, France), located 5 km west of the Loing Valley, has been excavated since 2009 by a CNRS team under the direction of Pierre Bodu (Bodu et al., 2010, 2019a). This open-air site presents different levels of human occupation from the Middle and Upper Paleolithic (Levallois, Discoid, Châtelperronian, Solutrean), and includes a level attributed to the Gravettian (Bodu et al., 2013; 2014; 2017; 2019b; Leroyer, 2021). The persistence of human occupations over nearly 100,000 years is probably linked to the topography and to the fact that the valley narrows considerably at the site, constituting a strategic point in the past for game hunting. Moreover, sandstone blocks surrounding the site likely provided a natural protection. The embedding loess sediment allowed a good preservation of osseous and lithic remains. Nine radiocarbon dates derive from the Gravettian layer providing an age of ca. 26,500 uncal BP (Bodu et al., 2019a). This occupation is attributed to an early phase of the Gravettian, characterised by rectilinear blades cut with soft stones and by the presence of numerous burins and Gravette points. This level yielded a single dwelling unit, which extends over nearly 100 m^2 and is particularly rich in archeological remains with nearly 17,000 fragments of flint artefacts and bones from a minimum of eight bison individuals, one reindeer and one horse (Lacarrière et al., 2015; Bodu et al., 2019a; Lacarrière, 2021). These large herbivores were probably hunted in the valley below the site. Bison is rare in faunal assemblages of the Paleolithic sites of the Paris Basin, and Ormesson's Gravettian level is the largest bison archaeological assemblage north of the Loire River. Therefore, Bison preferential hunting at Ormesson is particularly remarkable (Bodu et al., 2019a). One hundred sixty-seven pieces of shell ornaments imported from at least 80 km from Ormesson also testifies of the widespread connectivity of the site (Peschaux, 2021). The occupation is organised around two fire areas, including a hearth at the centre. Cut flints, pieces of animal carcasses and broken bones for extracting marrow were found around the hearth. A human deciduous tooth of a young individual (Ormesson 2016, level 1, locus 1, C29.2988) was discovered at the edge of this hearth, perfectly integrated within the Gravettian archaeological level. This human deciduous tooth was lost during the life of the individual at an estimated age of 10-12 years old. It indicates that other members of the group were presents with the Gravettian hunters when they came to the site ca. 30,000 years ago (Bodu et al., 2019a).

References:

Bodu P., Bignon O., Dumarçay G., 2010. Le gisement des Bossats à Ormesson, région de Nemours (Seine-et-Marne) : un site gravettien à faune dans le Bassin parisien. Mémoire LII, Société préhistorique française, p. 259-272.

Bodu P., Salomon H., Leroyer M., Naton H.-G., Lacarrière J., Dessoles M., 2013. An open-air site from the recent middle Palaeolithic in the Paris Basin (France): Les Bossats at Ormesson (Seine-et-Marne). Quaternary International, 331, p. 39-59.

Bodu P., Dumarçay G., Naton H.-G., 2014. Un nouveau gisement solutréen en Île-de-France, le site des Bossats à Ormesson (Seine-et-Marne). Bulletin de la société préhistorique française, 111, 2, p. 225-254.

Bodu P., Salomon H., Lacarrière J., Baillet M., Ballinger M., Naton H.-G., Théry-Parisot I., 2017. Un gisement châtelperronien de plein air dans le Bassin parisien : Les Bossats à Ormesson (Seine-et-Marne). Gallia Préhistoire, 57, p. 3-64.

Bodu P., Baillet M., Ballinger M., Dumarçay G., Goutas N., Jamon M., Julien M.-A., Lacarrière J., Legrand-Pineau A., Lejay M., Leroyer M., Lucas C., Moine O., Naton H.-G., Peschaux C., Salomon H., Stoetzel E., Suire J., Théry-Parisot I., Touzé O., 2019a. Le gisement paléolithique multistratifié des Bossats à Ormesson (Seine-et-Marne) : Palethnologie ou pâle ethnologie ? Actes du Congrès préhistorique de France, Amiens - 30 mai-4 juin 2016. Mémoire de la Société préhistorique française, 2, p. 231-262.

Bodu P., Bouché F., Ballinger M., Dumarçay G., Goutas N., Lacarrière J., Legrand-Pineau A., Lucas C., Naton H.-G., Théry-Parisot I., 2019b. The site of les Bossats in Ormesson (Seine-et-Marne, France). A vast solutrean campsite in the Paris Basin. *In:* Cascalheira J., Schmidt I. (Dir.), *Human adaptation to the Last Glacial Maximum: The Solutrean and its neighbors*, Cambridge Scholars Publishing. p. 65-88.
Lacarrière J., 2021. Hit the North! Review of recent archaeozoological discoveries from Gravettian sites in the North of France. Examples from Renancourt 1 and Les Bossats and regional perspective integrating Central Belgium. *In:* Touzé O., Goutas N., Salomon H., Noiret P. (Dir.), *Les sociétés gravettiennes du Nord-Ouest européen : nouveaux sites, nouvelles données, nouvelles lectures*, Actes du colloque international « Le Nord-Ouest européen au Gravettien : apports des travaux récents à la compréhension des sociétés et de leurs environnements », Université de Liège - 12-13 avril 2018. Liège: Presses universitaires de Liège (ERAUL, 150), Bruxelles: Société royale belge d'Anthropologie et de Préhistoire (Anthropologica et Præhistorica, 130), p. 53-73.
Lacarrière J., Bodu P., Julien M.-A., Dumarçay G., Goutas N., Lejay M., Peschaux C., Naton H.-G., Théry-Parisot I., Vasiliu L., 2015. Les Bossats (Ormesson, Paris basin, France): A new early Gravettian bison processing camp. Quaternary International, 359-360, p. 520-534.
Leroyer M., 2021. Les derniers quarante-mille ans du Paléolithique moyen en Ile-de-France (80-40 ka) : Lacunes et nouvelles perspectives au voisinage des affleurements de sables stampiens. De l'Ile-de-France à l'Europe du Nord-Ouest. Les peuplements humains avant le dernier maximum glaciaire. Bilan, objectifs et perspectives de la recherche. Revue archéologique de Picardie, n° spécial 36, p. 213-239.
Peschaux C., 2021. Objets de parure et pièces assimilées des sites gravettiens du nord-ouest de l'Europe. Nouvelles données fournies par l'étude des collections de Maisières « Canal » (Belgique), Les Bossats à Ormesson et Amiens-Renancourt 1 (France). *In:* Touzé O., Goutas N., Salomon H., Noiret P. (Dir.), *Les sociétés gravettiennes du Nord-Ouest européen : nouveaux sites, nouvelles données, nouvelles lectures*, Actes du colloque international « Le Nord-Ouest européen au Gravettien : apports des travaux récents à la compréhension des sociétés et de leurs environnements », Université de Liège - 12-13 avril 2018. Liège: Presses universitaires de Liège (ERAUL, 150), Bruxelles: Société royale belge d'Anthropologie et de Préhistoire (Anthropologica et Præhistorica, 130), p. 75-92.

Ostorf-Tannenwerder, Germany

Friedrich Lüth, Bettina Jungklaus, Thomas Terberger

Analyzed individuals:

- OST001 (Ostorf 2012-ind. 1): 5466-5321 calBP
- OST002 (Ostorf 2012-ind. 2): 5436-5053 cal BP
- OST003 (Ostorf 2012-ind. 3, mandible no. 212): 5314-5052 calBP

The small island Tannenwerder is located in Lake Ostorf in the city of Schwerin, northern Germany. The first inhumation burials were detected on the island already in the 19th century and various excavations during the 20th century revealed a larger cemetery with a total of c. 74 individuals across 33 burials (Lübke et al., 2009; Patolla & Henke, 2009). Fieldwork directed by F. Lüth in 2012 revealed a new burial and additional interments might be present at the site. While typological evidence suggested a Neolithic date, the location of the cemetery on an island is uncommon for this time. Furthermore, the frequent presence of grave goods such as transverse arrowheads, animal tooth pendants sometimes in large numbers, and fish hooks led to the interpretation of a surviving final

Mesolithic population (Schuldt, 1961). New systematic studies including a series of AMS-dates assign the site to ~5,200 to 4,900 calBP (Funnel Beaker to early Single Grave Culture) (Lübke et al., 2009; Olsen et al., 2010; Fernandes et al., 2015). The suggested Mesolithic lifestyle of the population is supported by elevated ^{15}N-values, which indicate regular consumption of freshwater resources (Fernandes et al., 2015; Terberger et al., 2018). Mesolithic roots for some of the individuals could also be revealed by the first paleogenetic data showing the presence of individuals with U5 mtDNA haplogroups (Bramanti et al., 2009). The Ostorf population was living within a spatially and temporally Funnel Beaker context and adopted some Neolithic cultural elements, but followed a subsistence strategy normally associated with a hunter-gatherer diet (Fernandes et al., 2015).

References:
Bramanti, B., Thomas, M. G., Haak, W., Unterlaender, M., Jores, P., Tambets, K., Antanaitis-Jacobs, I., Haidle, M., Jankauskas, R., Kind, C.-J., Lüth, F., Terberger, T., Hiller, J., Matsumura, S., Forster, P. & Burger, J. Genetic discontinuity between local hunter-gatherers and Central Europe's first farmers. Science 326, 2009, 137-140.
Fernandes, R., Grootes, P., Nadeau, M.-J. & Nehlich, O. Quantitative diet reconstruction of a neolithic population using a bayesian mixing model (FRUITS): the case study of Ostorf (Germany). Am. J. Phys. Anthropol. 158, 2015, 325-340. doi:10.1002/ajpa.22788.
Lübke, H., Lüth, F. & Terberger, T. Fishers or farmers? The Archaeology of the Ostorf Cemetery and Related Neolithic Finds in the Light of new information. Berichte der Römisch-Germanischen Kommission 88, 2007, 307-338.
Olsen, J., Heinemeier, J., Lüth, F., Lübke, H. & Terberger, T. Dietary habits and freshwater reservoir effects in bones from a Neolithic NE German cemetery. Radiocarbon 52, 2010, 635-644.
Patolla, M. & Henke, W. The skeletal population from Ostorf (Mecklenburg) – new evidence for its relationships and life-style. Berichte der Römisch-Germanischen Kommission 88, 2007, 353-383.
Schuldt, E., Abschließende Ausgrabungen auf dem jungsteinzeitlichen Flachgräberfeld von Ostorf 1961. Jahrbuch Bodendenkmalpflege in Mecklenburg, 1961, 131-178.
Terberger, T., Burger, J., Lüth, F., Müller, J. & Piezonka, H. Step by step - The Neolithisation of Northern Central Europe in the light of stable isotope analyses. Journal of Archaeological Science 99, 2018, 66-86.

Paglicci, Italy

Francesco Boschin, David Caramelli, Alessandra Modi, Martina Lari, Annamaria Ronchitelli, Stefano Ricci, Sahra Talamo

Analyzed individuals:
- Paglicci12 (PA12): 29104-28678 calBP
- Paglicci 133 (PA133): 34691-31239 calBP (layer)

Grotta Paglicci is located on the Gargano Promontory (Apulia, Foggia, southern Italy) at about 143 m above sea level. Excavations were directed by F. Zorzi (Natural History Museum of Verona) from 1961 to 1963, and by the University of Siena from 1971 onwards (by Prof. A. Palma di Cesnola first and later by Prof. A. Ronchitelli). Beside part of the deposit that yielded ancient Middle Paleolithic and Acheulean lithic industries, the main part of the 12 m thick stratigraphy embraces several phases of the Upper Paleolithic, spanning from the Protoaurignacian to the Final Epigravettian (Berto et al., 2017). Several mobiliary art objects were found in the Gravettian and particularly in the Epigravettian

layers. In addition, in an inner room there are the only Paleolithic wall paintings discovered in Italy so far depicting horses and hands, probably dated to the ancient Epigravettian (Palma di Cesnola, 2001). The human remains analysed in this work are from different stratigraphic units. Dates reported below are calibrated with the IntCal20 curve in the OxCal 4.4 software (Ramsey, 2009; Reimer et al., 2020).
PA133 is an isolated lower third molar from layer 23C2, referred to the beginning of the Early Gravettian at the site. This layer yielded a tephra with strong affinities to the Codola eruption, dated to ~33,000 years ago (Giaccio et al., 2008). This is in line with ^{14}C dates performed on charcoal from levels above and below layer 23C2 i.e., layer 23A is dated to 33,364-31,238 calBP (UTC-1414), whilst layer 24A1 to 34,690-32,195 cal BP (UTC-1789) (Palma di Cesnola, 2004).
PA12 is a young individual (aged 12-13 years) found at the top of layer 22. The body laid on the back without any pit being dug into the cave's floor. Genetic analysis carried out in this work indicates that the sex is female. The individual was buried with a funeral kit composed by a bone piercer, eleven lithic implements, one manganese block, two gastropod shells (*Luria lurida*) and several deer craches, most of them close to the skull, one to the right ankle, and one on the left wrist. The left humerus was missing (Ronchitelli et al., 2015; Mezzena and Palma di Cesnola, 1972).
A new direct ^{14}C date was performed on one rib. The collagen was extracted at the Department of Human Evolution, Max Planck Institute for Evolutionary Anthropology (MPI-EVA) in Leipzig (Germany) following the procedures in Talamo and Richards (2011) and Talamo et al., (2021) (MPI-Code: R-EVA). The outer surface of the bone sample was first cleaned by a shot blaster and then 500 mg of the whole bone was taken. The sample was then decalcified in 0.5M HCl at room temperature until no CO_2 effervescence is observed. 0.1M NaOH is added for 30 minutes to remove humics. The NaOH step was followed by a final 0.5M HCl step for 15 minutes. The resulting solid was gelatinized following Longin (1971) at pH 3 in a heater block at 75°C for 20h. The gelatin was then filtered in an Eeze-Filter™ (Elkay Laboratory Products (UK) Ltd.) to remove small (>80 μm) particles. The gelatin was then ultrafiltered 60 with Sartorius "VivaspinTurbo" ultrafilters (30 kDa MWCO) (Brown et al., 1988). Prior to use, the filter was cleaned to remove carbon-containing humectants (Brock et al., 2007). The sample was then lyophilized for 48 hours. To supervise possible contamination introduced during the pre-treatment stage, a pretreated ^{14}C-free bone sample was used (Korlevic et al., 2018). Prior to sending the sample to the Mannheim facility for AMS dating (laboratory code MAMS; Kromer et al., 2013) the collagen yield, C:N ratios, together with isotopic values were evaluated and all passed the evaluation criteria (bones with >1% weight collagen and C:N ratios in the range 2.9–3.6; Van Klinken, 1999) for good quality collagen.

References:

Berto, C., Boscato, P., Boschin, F., Luzi, E. & Ronchitelli, A. Paleoenvironmental and paleoclimatic context during the Upper Palaeolithic (late Upper Pleistocene) in the Italian Peninsula. The small mammal record from Grotta Paglicci (Rignano Garganico, Foggia, Southern Italy). *Quaternary Science Reviews* **168**, 30-41 (2017).
Brock, F., Bronk Ramsey, C. & Higham, T. Quality assurance of ultrafiltered bone dating. *Radiocarbon* **49(2)**, 187-192 (2007).
Brown, T. A., Nelson, D. E., Vogel, J. S. & Southon, J. R. Improved Collagen Extraction by modified Longin method. *Radiocarbon* **30(2)**, 171-177 (1988).
Giaccio, B., et al., The Campanian Ignimbrite and Codola tephra layers: Two temporal/stratigraphic markers for the Early Upper Palaeolithic in southern Italy and eastern Europe. *Journal of Volcanology and Geothermal Research* **177**, 208-226 (2008).
Korlević, P., Talamo, S. & Meyer, M. A combined method for DNA analysis and radiocarbon dating from a single sample. *Scientific Reports* **8(1)**, 4127 (2018).

Kromer, B., Lindauer, S., Synal, H.-A. & Wacker, L. MAMS – A new AMS facility at the Curt-Engelhorn-Centre for Achaeometry, Mannheim, Germany. *Nuclear Instruments and Methods in Physics Research Section B: Beam Interactions with Materials and Atoms* **294**, 11-13 (2013).
Longin, R. New method of collagen extraction for radiocarbon dating. *Nature* **230(5291)**, 241-242 (1971).
Mezzena F. & Palma di Cesnola, A. Scoperta di una sepoltura gravettiana nella Grotta Paglicci (Rignano Garganico). *Rivista di Scienze Preistoriche* **27**, 27-50 (1972).
Palma di Cesnola, A. Le Paléolithique supérieur en Italie. Série "Préhistoire d'Europe" 9 (Jérôme Millon, Grenoble, 2001).
Palma di Cesnola, A. Paglicci. L'Aurignaziano e il Gravettiano antico (Claudio Grenzi Ed., 2004).
Ramsey, C. B. Dealing with outliers and offsets in radiocarbon dating. *Radiocarbon* **51(3)**, 1023-1045 (2009).
Reimer, P. J., et al., The IntCal20 Northern hemisphere radiocarbon age calibration curve (0–55 cal kbp). *Radiocarbon* **62 (4)**, 1-33 (2020).
Ronchitelli, A., et al., When technology joins symbolic behaviour: The Gravettian burials at Grotta Paglicci (Rignano Garganico e Foggia e Southern Italy). *Quaternary International* **359-360**, 423-441 (2015).
Talamo, S. & Richards, M. A comparison of bone pretreatment methods for AMS dating of samples >30, 000 BP. *Radiocarbon* **53(3)**, 443-449 (2011).
van Klinken, G. J. Bone Collagen Quality Indicators for Palaeodietary and Radiocarbon Measurements. *Journal of Archaeological Science* **26**, 687-695 (1999).

Pincevent, France

Olivier Bignon-Lau, Frédérique Valentin, Pierre Bodu, Grégory Debout, Michel Orliac, Antoine Zazzo

Analyzed individual:

- PIN004 (IV0-35,M104,26): 15360-15041 calBP (layer)

The site of Pincevent (La Grande Paroisse; Seine-et-Marne, France) is a very well-preserved open-air site of the Paris basin, with 23 Magdalenian occupation floors. The site was discovered in 1964 and has been excavated since (Leroi-Gourhan, Brézillon, 1966). At 5 km downstream the Yonne-Seine confluence, several smooth overflows of silt or sandy silt from the Seine deposits produced an exceptional stratigraphic sequence that has recorded frequent visits of Magdalenian-associated groups during approximately one century. Among the successive occupation floors recorded, two large residential camps have been investigated. The largest one spanning 4,500 m² corresponds to level IV20, which has been interpreted as a mass killing site of reindeers (at least 76 individuals) during their autumn migration (Leroi-Gourhan, Brézillon, 1972; Julien, Karlin, 2014). Level IV0 with its four occupation units is less excavated (1,400 m²) and only one has been published so far (Bodu et al., 2006). Contrasting with level IV20, the Magdalenian-associated remains of level IV0 have yielded larger units, evidence of hunting of both reindeer and horse during a year-round occupation, and the presence of eight human bones associated with the smallest unit named 35-M103. Such a unit seems to carry a set of features rendering it different from the others, but refittings of burned stones and knapped flints confirm a strict contemporaneity of the whole camp. Particularities of unit 35-M103 consist both in the limited amount of artefacts and particularly in their composition such as unburned large stones and a strongly disproportionate number of reindeer scapulas and horse maxillary teeth.

Several skeletal human elements have been identified in the middle of this unit i.e., upper second premolar, humerus, radius, femur and fibula. The newly reported radiocarbon date (MUSE-21121, 12750±45 uncal BP) was performed on the human premolar from the same archeological unit but it is not possible to assess if it belongs to the same individual as the femur from which we obtained genetic data.

References:
Leroi-Gourhan, A., & Brézillon, M. (1966) - L'habitation magdalénienne n°1 de Pincevent, près de Montereau (Seine-et-Marne). *Gallia Préhistoire*, 9, p. 263-385.
Leroi-Gourhan, A., & Brézillon, M. (1972) - *Fouilles de Pincevent: Essai d'analyse ethnographique d'un habitat magdalénien (la section 36)*. Paris, CNRS Editions, Gallia Préhistoire, VIIe supplément.
Bodu, P., Julien, M., Valentin, B., & Debout, G. (2006) - Un dernier hiver à Pincevent: Les magdaléniens du niveau IV0 (Pincevent, La Grande-Paroisse, Seine-et-Marne). *Gallia Préhistoire*, 48, p. 1-180.
Julien, M., & Karlin, C. (2014) - *Un automne à Pincevent - Le campement magdalénien du niveau IV20*. Paris, Société préhistorique française, Mémoire n° 57, 639 p.

Pradis, Italy

Stefano Benazzi, Matteo Romandini, Christiana L. Scheib, Tina Saupe, Helen, Fewlass, Sahra Talamo

Analyzed individual:
- PRD001 (Grotte Pradis 1): 13089-12898 calBP

The Pradis caves on the eastern side of the Carnic Prealps in the Friuli Venezia Giulia region (Italy) are located at 560 m a.s.l. in the Pradis Plateau. Bordered by mountains reaching 2,000-2,300 m, the Plateau covers an area of over 6 km^2 at an elevation of 530-590 m, is wedged to the north between Pala Mount (1,231 m), Rossa Mount (1,369 m) and Ciaurlec Mount (1,148m), while to the south by the gorge of the Cosa stream that flows towards the upper Friulian plain. Between the plain and the Prealps, this plateau was of strategic importance for access to the mountain. Over the millennia, the hydrographic network, developed tight and deep ravines (Torrente Cosa and Rio Secco), with numerous natural shelters and caves excavated in carbonate formations, some of which were filled with Pleistocene sediments. Among these are the Pradis caves (or Grotte "Verdi" di Pradis), used during the Mousterian and the Late Glacial Epigravettian (Bartolomei et al., 1979; Corai, 1980; Gurioli et al., 2011); Grotta del Rio Secco with a Mousterian and Gravettian levels (Peresani et al., 2014; Romandini et al., 2014; 2018; Talamo et al., 2014) and Grotta del Clusantin (Peresani et al., 2009; Romandini et al., 2012). Despite the abundance of paleontological and archaeological materials found, the three shelters of the Pradis caves were radically transformed into a tourist place of worship and refreshment, with the consequent loss of most of the information sealed in the sediments. The deposits spared were investigated in 1970 and 1971 under the direction of a paleontologist, G. Bartolomei (University of Ferrara) (Bartolomei et al., 1979; Corai, 1980; Bartolomei and Tonon, 1997). Of the three shelters, in the Riparo I, Pleistocene deposits were still present in two meters thick, but limited to a 3 m^2 backed to the wall at the bottom. The stratigraphic succession is composed of a set of units mainly formed by the accumulation of cryoclastic gravel with variable content of silts and guano levels. At the base, a thick layer of gravel with clayey matrix (levels 13 to 7) contained almost exclusively cave bear bones and some rare Mousterian lithic artifacts. Above these, a level of

guano (6) is covered with low silt gravel (5) and gravel with larger amount of silt (layers 4 to 2). Finally, layers 2 and 1 (the latter being split into 1a and 1b) yielded abundant bones of mammals, especially marmots (Nannini et al., 2022) and the deciduous human tooth analyzed in this study (Lugli et al., 2022). The lithic artifacts from levels 1 and 2 are made of allochthonous flint, probably from the basin of the Venetian Prealps (Corai, 1980), and refer to the Late Epigravettian. Along with lithic artifacts, hard animal material artifacts have also been preserved: two marmot clavicles with incised notches and two bone points (layer 1a) (Gurioli et al., 2011). The faunal association records large cervids (moose, red deer) and caprids (ibex and chamois) with cut marks and percussion marks (Nannini, 2018; Nannini et al., 2022). However, the most targeted game was the alpine marmot (*Marmota marmota*), as pointed out from the 99% of the bones (over 11,000 NISP). These remains show an evident standardized butchering scheme that allows the reconstruction of various processing phases.

Recently, new results were added to this aspect with a multi-disciplinary investigation on a deciduous human tooth (Pradis 1) recovered from the Epigravettian layers (Lugli et al., 2022). Pradis 1 is an exfoliated deciduous molar (Rdm_2) lost during life by an 11-12-year-old child. A direct radiocarbon date provided an age of 13,088-12,897 cal BP (95% probability, IntCal20). In Lugli et al. 2022, the Amelogenin peptides extracted from tooth enamel indicate that Pradis 1 likely belonged to a male individual. In the same study, the Sr isotope ratio of the tooth enamel differs from the local baseline value, suggesting that the child spent his first year of life far from the Pradis caves. Sr isotopes are also suggestive of a cyclical/seasonal mobility pattern exploited by the Epigravettian hunters.

Close to the end of the Upper Palaeolithic, the general scenario emerging from these studies tells how in the Pradis Plateau the shelters of the Pradis caves alongside the neighbouring Clusantin cave were used by seasonal nomad human groups, who consumed marmot in a particular geographic area specialized in the hunting and exploitation of this rodent.

A more detailed description of the archaeological context of the Pradis caves is published in Gurioli et al., 2011 and Nannini et al., 2022.

References:

Bartolomei, G., Broglio, A., Palma di Cesnola, A., 1979. Chronostratigraphie et écologie de l'Epigravettien en Italie. In: De Sonneville-Bordes, D. (Ed.), La fin des temps glaciaires en Europe. Colloques Internationaux du C.N.R.S, vol. 271, pp. 297-324.

Bartolomei, G., Tonon, M., 1997. Un esempio di paleoecologia del Tardiglaciale e dell'Olocene antico in Insediamenti preistorici del Friuli occidentale (ed. Diego Gaspardo). Bollettino Società Naturalisti "Silvia Zenari", Pordenone, pp. 93-102.

Corai, P., 1980. Le più antiche culture preistoriche della Ladinia (Paleolitico e Mesolitico). Ladinia IV, 183-218.

Gurioli, F., Bartolomei, G., Nannini, N., Peresani, M., Romandini, M., 2011. Deux clavicules de marmotte épigravettiennes incisées, provenant des Grottes Verdi de Pradis (Alpes italiennes). Paleo 22, 311-318.

Lugli, F., Nava, A., Sorrentino, R., Vazzana, A., Bortolini, E., Oxilia, G., Silvestrini, S., Nannini, N., Bondioli, L., Fewlass, H., Talamo, S., Bard, E., Mancini, L., Müller, W., Romandini, M., Benazzi, S., 2022. Tracing the mobility of a Late Epigravettian (~ 13 ka) male infant from Grotte di Pradis (Northeastern Italian Prealps) at high-temporal resolution. Sci. Rep. 12, 8104.

Nannini, N., 2018. Tra archeozoologia, paleobalistica e antropologia. Lettura degli impatti delle armi da getto epigravettiane su resti faunistici nel Tardoglaciale dell'Italia nord-orientale. Phd thesis, University of Ferrara, 233 p.

Nannini, N., Duches, R., Fontana, A., Romandini, M., Boschin, F., Crezzini, J., Peresani, M., 2022. Marmot hunting during the Upper Palaeolithic: The specialized exploitation at Grotte di Pradis (Italian pre-Alps). Quaternary Science Reviews 277, 107364.
Peresani, M., Duches, R., Miolo, R., Romandini, M., Ziggiotti, S., 2011. Small specialized hunting sites and their role in Epigravettian subsistence strategies. A case study in Northern Italy. In: Bon, Fr., Costamagno, S., Valdeyron, N. (Eds.), Hunting Camps in Prehistory. Current Archaeological Approaches, Proceedings of the International Symposium, May 13-15 2009, University Toulouse II - Le Mirail, P@lethnology, 3, 251-266
Peresani, M., Romandini, M., Duches, R., Jéquier, C., Nannini, N., Pastors, A., Picin, A., Schmidt, I., Vaquero, M., Weniger, G. C., 2014. New evidence for the Neanderthal demise and earliest Gravettian occurrences at Rio Secco Cave, Italy. J. Field Archaeol. 39, 401-416.
Romandini, M., Peresani, M., Gurioli, F., Sala, B., 2012. *Marmota marmota*, the most common prey species at Grotta del Clusantin: Insights from an unusual casestudy in the Italian Alps. ICAZ International Conference of Archaeozoology, Session 4-3, Hominin Subsistence in the Old World during the Pleistocene and Early Holocene. Quaternary International 252, 184-194.
Romandini, M., Peresani, M., Laroulandie, V., Metz, L., Pastoors, A., Vaquero, M., Slimak, L., 2014. Convergent evidence of eagle talons used by Late Neanderthals in Europe: a further assessment on symbolism. PLoS ONE 9(7), e101278.
Romandini, M., Terlato, G., Nannini, N., Tagliacozzo, A., Benazzi, S., Peresani, M., 2018. Humans and Bears a Neanderthal tale. Reconstructing uncommon behaviors from zooarchaeological evidence in southern Europe. J. Archaeol. Sci. 90, 71-91.
Talamo, S., Peresani, M., Romandini, M., Duches, R., Jéquier, C., Nannini, N., Pastors, A., Picin, A., Vaquero, M., Weninger, G. C., Hublin, J. J., 2014. Detecting human presence at the border of the northeastern Italian Pre-Alps. 14C dating at Rio Secco Cave as expression of the first Gravettian and the late Mousterian in the northern Adriatic region. PLoS ONE 9(4), e95376.

Rigney, France

Christophe Cupillard, Frédérique Valentin

Analyzed individual:
- Rigney (Rigney1): 15646-15271 calBP

Archeological description previously published in Posth et al., 2016 and Fu et al., 2016.

Riparo Tagliente, Italy

Stefano Benazzi, Gregorio Oxilia, Eugenio Bortolini, Luca Pagani, Christiana L. Scheib, Tina Saupe, Sahra Talamo

Analyzed individual:
- RIP001 (Tagliente 2): 16976-16510 calBP

The site of Riparo Tagliente is located in one of the main valley bottoms of the pre-alpine massif of Monti Lessini (Valpantena Valley). Discovered in 1958 by Francesco Tagliente, the site has been investigated since 1962 by the *Museo Civico di Storia Naturale* of Verona and, some years later, by the University of Ferrara (Italy). The stratigraphic series is formed by two main deposits separated by

a river escarpment: a lower one referred to MIS 4-3 occupation with Mousterian and Aurignacian assemblages, and an upper one dated to the Late Glacial attesting a Late Epigravettian occupation. The latter is one of the most complete in Northern Italy spanning between the first part of the Lateglacial and the beginning of the Bølling–Allerød interstadial according to radiocarbon dates which range from 17,219-16,687 calBP (layer 13a alpha) to 14,572–13,430 calBP (layers 10–8) (Fontana et al., 2009; 2018).

Riparo Tagliente therefore offers the earliest evidence of human re-occupation of the southern Alpine slope (Fontana et al., 2009; 2018), at the beginning of glacier withdrawal at 17,700–17,300 calBP (Ravazzi et al., 2014; Monegato et al., 2017). The favourable position of the site facilitated intensive occupation by Late Epigravettian groups during the spring and the end of the summer. Its archaeological record consists of structures, fireplaces, rich lithic assemblages, faunal and ochre remains, bone tools and marine-shell beads as well as beads obtained from and red deer canines (Bietti et al., 2004; Peretto et al., 2004; Cavallo et al., 2017a; 2017b). Diversity and abundance of findings suggest an efficient exploitation of local resources (Bertola et al., 2007; Fontana et al., 2009), although the presence of tools and artefacts obtained from Northern Adriatic Apennine cherts (Umbria-Marche Basin), as much as the presence of hundreds of marine shell beads, corroborate the hypothesis of persistent exchanges with this area (Fontana et al., 2009; Bertola et al., 2018). A burial (Tagliente 1) was uncovered in the southern sector of the site in 1973 (Bartolomei et al., 1974; Corrain 1977) and dated to 16,150–15,530 calBP (Gazzoni et al., 2013). Unfortunately, a Medieval pit had removed the upper part of the skeleton. Only a few ribs and vertebrae, the distal fragments of the radius and ulna and some phalanges were discovered on the trampling floor, while the lower part of the skeleton from the pelvis to the feet was well preserved in the grave (Broglio & Villabruna 1991). At the same site, an incomplete left hemi-mandible (named Tagliente 2) was found within disturbed sediments located immediately outside the shelter (deposit reworked in historical times). As previously documented (Corrain, 1966; Oxilia et al., 2021), the mandible is preserved with part of the body and the ramus preserved. More in detail, the condyle process is missing as well as the portion of the mandibular body in front of the mental foramen and the alveolus of the left first premolar. The preservation of dental tissues provided several pieces of information on dietary habits and pathological condition showing an emphasised contribution of animal proteins in the diet of this individual (Oxilia et al., 2021), as well as the earliest evidence of focal cemento-osseous dysplasia (FCOD) in Europe (Bortolini et al., 2021). Radiocarbon dating obtained from the root of the first molar (16,980–16,510 calBP; Bortolini et al., 2021) confirms Riparo Tagliente as the earliest direct evidence of Epigravettian recolonization of the southern Alpine slopes. Previous genomic analysis carried out on the hemimandible (Bortolini et al., 2021) has already shown affinities between this individual and the *Villabruna* cluster (Fu et al., 2016), supporting the hypothesis of a much earlier than previously thought diffusion of *Villabruna* genetic components linked to Balkans/Anatolian refugia into Southern Europe (at least ~17,000 calBP).

The anthropological materials found at Ripato Tagliente are currently stored at the Natural History Museum of Verona. Permission to access the Tagliente 2 specimen was granted by the SABAP of Verona, Rovigo and Vicenza (Veneto Archaeological Superintendency, Ministry of Cultural Heritage and Activities of Italy) which is legally responsible for the conservation and scientific study of the skeletal remains found at the site.

References:

Bartolomei, G. et al., Una sepoltura epigravettiana nel deposito pleistocenico del Riparo Tagliente in Valpantena (Verona). *Rivista di Scienze Preistoriche* **29**, 101–152 (1974).

Bertola, S., Broglio, A. & Cassoli, P. F. in *L'Italia tra 15.000 e 10.000 anni fa. Cosmopolitismo e regionalità nel Tardoglaciale* 39–94 (Museo Fiorentino di Preistoria "Paolo Graziosi", 2007).

Bertola, S., Fontana, F. & Visentin, D. in *Palaeolithic Italy. Advanced Studies on Early Human Adaptations in the Apennine Peninsula* 219–246 (Sidestone Press, 2018).
Bietti, A. et al., Inorganic Raw Materials Economy and Provenance of Chipped Industry in Some Stone Age Sites of Northern and Central Italy. *Coll. Antropol.* **28**, 41–54 (2004).
Bortolini, E. et al., Early Alpine occupation backdates westward human migration in Late Glacial Europe. *Curr. Biol.* **31**, 2484–2493.e7. doi: 10.1016/j.cub.2021.03.078.
Broglio, A. & Villabruna, A. Vita e morte di un cacciatore di 12000 anni fa. Risultati preliminari degli scavi nei ripari Villabruna (Valle del Cismon. Val Rosna, Sovramonte, Belluno). *Odeo Olimpico* 1–19 (1991).
Cavallo, G. et al., Sourcing and processing of ochre during the late upper Palaeolithic at Tagliente rockshelter (NE Italy) based on conventional X-ray powder diffraction analysis. *Archaeol. Anthropol. Sci.* **9**, 763–775 (2017a).
Cavallo, G. et al., Textural, microstructural, and compositional characteristics of Fe-based geomaterials and Upper Paleolithic ocher in the Lessini Mountains, Northeast Italy: Implications for provenance studies. *Geoarchaeology* **32**, 437–455 (2017b).
Corrain, C. Un frammento di mandibola umana, rinvenuto al RiparoTagliente in Valpantena (Verona). *Atti dell'Istituto Veneto di Scienze, Lettere ed Arti* **1965-66** (1966).
Corrain, C. I resti scheletrici umani della sepoltura epigravettiana del Riparo Tagliente in Valpantena (Verona). *Bollettino del Museo Civico di Storia Naturale di Verona* **4**, 35–79 (1977).
Fontana, F., Cilli, C., Cremona, M. G., Giacobini, G. & Gurioli, F. Recent data on the Late Epigravettian occupation at Riparo Tagliente, Monti Lessini (Grezzana, Verona): a multidisciplinary perspective. *Preistoria Alp* **44**, 51–59 (2009).
Fontana, F. et al., in *Palaeolithic Italy. Advanced Studies on Early Human Adaptation in the Apennine Peninsula* (eds. Borgia, V. & Cristiani, E.) 287–310 (Sidestone, 2018).
Fu, Q. et al., The genetic history of Ice Age Europe. *Nature* **534**, 200–205 (2016).
Gazzoni, V., Goude, G., Herrscher, E. Guerreschi, A., Antonioli, F. & Fontana, F. Late Upper Palaeolithic human diet: first stable isotope evidence from Riparo Tagliente (Verona, Italy). *Bull. Mém. Soc. Anthropol. Paris* **25**, 103–117 (2013).
Monegato, G., Scardia, G., Hajdas, I., Rizzini, F. & Piccin, A. The Alpine LGM in the boreal ice-sheets game. *Sci. Rep.* **7**, 2078 (2017).
Oxilia, G. et al., Exploring late Paleolithic and Mesolithic diet in the Eastern Alpine region of Italy through multiple proxies. *Am. J. Phys. Anthropol.* **174**, 232–253 (2021).
Peretto, C., Biagi, P., Boschian, G. & Broglio, A. Living-Floors and Structures From the Lower Paleolithic to the Bronze Age in Italy. *Coll. Antropol.* **28**, 63–88 (2004).
Ravazzi, C. et al., The latest LGM culmination of the Garda Glacier (Italian Alps) and the onset of glacial termination. Age of glacial collapse and vegetation chronosequence. *Quat. Sci. Rev.* **105**, 26–47 (2014).

San Teodoro, Italy

David Caramelli, Alessandra Modi, Giulio Catalano, Luca Sineo

Analyzed individual:

- STO001 (ST2): 13577-13347 calBP

The San Teodoro cave is located around 2 km from the sea shore and 135 m above sea-level in the province of Messina in Sicily (Italy). The site is a limestone cave discovered by F. Anca who

described it in 1860 (Galland et al., 2019). Afterwards Gemellaro and De Gregorio excavated the cave followed by Vaufrey in 1925. Between 1937 and 1940 the skeletons of San Teodoro 1, 2, 3, 6 and 7 were discovered and in 1942 the skeletons of San Teodoro 4 and 5 were found by C. Maviglia, P. Graziosi and L. Bernabò Brea, who described a stratigraphic sequence of six layers (from F at the bottom to A at the top). All human skeletal remains derive from layer D, despite being retrieved excavating through layer E that did not contain lithic industries but animal remains including *Elephas mnaidriensis*, *Equus hydruntinus, Cervus elaphus* and *Crocuta crocuta spelaea* (Bonfiglio et al., 2001; Catalano et al., 2020; D'Amore et al., 2009; Graziosi, 1943; 1947; Graziosi & Maviglia, 1946; Vigliardi, 1968). The San Teodoro 2 individual was buried in a shallow grave along the cave axis with arms along the body and legs extended towards the south. The individual was covered with red ochre and accompanied by deer horns, animal bones and fluited pebbles as grave goods (Graziosi, 1947; Graziosi & Maviglia, 1946; Vigliardi, 1968). Archeologically the human occupation of San Teodoro cave is associated with a late Upper Paleolithic lithic industry such as the Late (or Final) Epigravettian culture containing from flint of microlithic type to large quartz flakes (Graziosi, 1947; Graziosi & Maviglia, 1946; Vigliardi, 1968). The individual San Teodoro 1 was directly radiocarbon dated to 12,580 ± 130 years BP (ETH-34451), which supports the Late Epigravettian age for this individual (Mannino et al., 2011). The genetic analysis performed in this study showed that San Teodoro 2, previously attributed to a male on the basis of the skull morphology (Graziosi, 1947; Mallegni, 2005), is instead a female individual.

References:
Bonfiglio L., Mangano G., Marra A.C. & Masini F. 2001. A new late Pleistocene vertebrate faunal complex from Sicily (S.Teodoro cave, North-Eastern Sicily, Italy). *Boll. Soc. Paleontol. Ital.*, 40: 149-158.
Catalano G., Modi A., Mangano G., Sineo L., Lari M. & Bonfiglio L. 2020. A mitogenome sequence of an Equus hydruntinus specimen from Late Quaternary site of San Teodoro Cave (Sicily, Italy). *Quaternary Science Reviews*, 236: 106280.
D'Amore G., Di Marco S., Tartarelli G., Bigazzi R. & Sineo L. 2009. Late Pleistocene human evolution in Sicily: comparative morphometric analysis of Grotta di San Teodoro craniofacial remains. *Journal of Human Evolution*, 56: 537-550.
Galland M., D'Amore G., Friess M., Miccichè R., Pinhasi R., Sparacello V.S. & Sineo L. 2019. Morphological variability of Upper Paleolithic and Mesolithic skulls from Sicily. *Journal of Anthropological Sciences*, 97: 151-172.
Graziosi P. 1943. Gli scavi dell'Istituto Italiano di Paleontologia Umana nella Grotta di S. Teodoro (Messina). *Memorie della Società Toscana di Scienza Naturali*, 52: 82-99.
Graziosi P. 1947. Gli uomini paleolitici della Grotta di S. Teodoro (Messina). *Rivista di Scienze Preistoriche*, 2: 123-233.
Graziosi P. & Maviglia C. 1946. La Grotta di S. Teodoro (Messina). *Rivista di Scienze Preistoriche*, 1: 277-283.
Mallegni F. 2005. San Teodoro. In: G. Alciati, V. Pesce Delfino & E.Vacca (eds.): *Catalogue of Italian human remains from the Palaeolithic to the Mesolithic*. Supplement to the Volume 83 of *J. Anthropol. Sci.*, pp. 133-136.
Mannino M.A., Di Salvo R., Schimmenti V., Di Patti C., Incarbona A., Sineo L. & Richards M.P. 2011. Upper Palaeolithic hunter-gatherer subsistence in Mediterranean coastal environments: an isotopic study of the diets of the earliest directly-dated humans from Sicily. *Journal of Archaeological Science*, 38: 3094-3100.
Vigliardi A. 1968. L'industria litica della grotta di S. Teodoro in provincia di Messina. *Rivista di Scienze Preistoriche*, 23: 33-144.

Tutkaul, Tajikistan

Svetlana Shnaider, Tatiana Filimonova, Natalia Berezina

Analyzed individual:

- TTK001 (Burial n1): 8419-8026 calBP (layer 2)

The Tutkaul site is located in Tajikistan, 70 km southeast of Dushanbe in the Dashti-Mazar region on the bank of Vakhsh River. In 1956, A.P. Okladnikov discovered the site during an archeological survey of the Nurek reservoir flooded areas (Okladnikov, 1959). The Tajik Archeological Expedition led the rescue archeological program leaded by V.A. Ranov during six field seasons (1963, 1965-1969) in which the site was excavated (Ranov & Korobkova, 1971). The uppermost level at the site has a medieval fortified settlement and Bronze age layer under which level 1 and 2 is associated with the Hissar Neolithic culture. Below this level there are levels 2a and 3 that represent the lowest stratigraphic units linked to the Early and Late Mesolithic (Shnaider et al., 2020). Three human burials were discovered at the base of level 2. Those contain a total of four individuals: two juvenile individuals (burial 3), an adult (burial 1), and a young adult (burial 2). All skeletons were positioned on the left side and with southeast-northwest orientation. In burial 1 a complete skeleton was found laying in a crouched position (Kiyatkina, 1976). The upper and lower jaw teeth were abraded. The skull combined morphological features characteristic of both males and females. It was finally attributed to a female individual (Kiyatkina, 1976) but the genetic sex is found to be male. Investigation of the postcranial skeleton was not performed or not published. Due to the severe abrasion of the teeth, this feature was not used in determining the age. The age of the individual was instead determined to 30-40 years based on the degree of obliteration of the skull sutures. The skull as well as postcranial bones from burial 1 are now lost. The young adult individual (burial 2) was represented by a skull vault and the upper part of the postcranial skeleton, bones from the face were absent and no teeth were mentioned in the first publication. According to the preservation and degree of wearing, the genetically analyzed tooth most likely belongs to burial 1 but direct radiocarbon dating on it was unsuccessful. The Golden Valley laboratory obtained a dating of 8,425-8,025 calBP (GV-02104, 7450±106 BP) from an unidentified bone fragment from burial 3 (Kocher et al., 2021), which possibly dated to a similar period of burial 1 since they derive from the same stratigraphic level.

References:

Kiyatkina, T.P., Materialy k paleoantropologii Tadzhikistana. Donish Publishing House, Dushanbe, 1976. P. 186.

Kocher, A., Papac, L., Barquera, R., Key, F.M., Spyrou, M.A., Hübler, R., Rohrlach, A.B., Aron, F., Stahl, R., Wissgott, A., van Bömmel, F. Ten millennia of hepatitis B virus evolution. *Science* **374**(6564), 182–188 (2021).

Okladnikov, A.P. [The investigations of Stone Age sites in Tajikistan. Preliminary results of work 1948, 1952–1954]. *Mat. Investig. USSR Archaeol.* **66**, 12–71 (1958).

Ranov, V.A., Korobkova, G.F. Tutkaul–mnogosloinoe poselenie gissarskoi kultury v yuzhnom Tadjikistane. *Soviet Archaeology* **2**, 133–147 (1971).

Shnaider, S.V., Kolobova, K.A., Filimonova, T.G., Taylor, W., Krivoshapkin, A.I. New insights into the Epipaleolithic of western Central Asia: The Tutkaulian complex. *Quat. Int.* **535**, 139–154 (2020).

Urd cave, Germany

Tim Schüler

Analysed individual:

- DOB001 (711/59): 9543-9332 calBP

The site Urd cave site is part of the region around the village of Döbritz in the southeastern part of Germany. Other better-known caves are also located in the direct vicinity, such as Wüste Scheuer and Kniegrotte. All these caves and rock shelters were formed by erosion in the dolomite of a Zechstein reef. The research of the Zechstein region is closely connected with the name of Martin Richter from Pößneck, who started excavating the Kniegrotte in 1930 (Richter 1955). After the first explorations of Urd cave in 1937, M. Richter examined the largest part of the cave between 1946 and 1955. In 1968 Rudolf Feustel reopened the western profile on the slope in front of the cave to further study the site stratigraphy (Feustel 1971). The excavated part of the Urd cave with a length of around 40 m can be subdivided into different sections. The old entrance of the cave, towards the so-called *Einbruchskessel*, is located in the southern part of the complex and provides access to the partially collapsed first hall, the so-called *Südhalle*. Around 2.5 m above the floor of the first room near the entrance starts a second cavity with a length of around 7 m and a width of around 3.5 m. The height decreases rapidly from 2.5 m in the passage around the *Südhalle* to a final height of 1 m. This hall was named *Oberhalle* by the excavator and is the location where the genetically analysed human skull was found. From here, after ~15 m towards the northeast begins the third cave complex at the end of the *Südhalle*. M. Richter named the larger parts *Hochkammer* and *Kultkammer*, which are in total ~18 m long ending into an additional entrance (Terberger et al. 2003).

Unfortunately, it is not possible to match up the stratigraphic relations of individual parts of the cave. It seems that only a Late Palaeolithic lower part and a mixed (Upper Palaeolithic to Bronze Age and younger) upper part exist. The reasons for the lack of homogeneity were previously discussed and are possibly the result of differential processes like transportation by water, bioturbation and solifluction from the plateau (Feustel 1971, Ullrich 1975, Terberger et al. 2003).

The skull of an adult male individual (Grimm and Ullrich 1965) was found 1.2 m above the floor of the *Oberhalle* in a dolomite sand layer, the so-called *homo* layer. In this layer, six postcranial remains from a minimum number of two individuals were also found (Bach 1974, Orschiedt 1999). Older literature proposed an Upper Palaeolithic age of the human remains, based on the type of two artefacts found close to the human bones (Grimm and Ullrich 1965). The lithic assemblage with a total of 133 pieces suggests a Magdalenian to Late Palaeolithic association without differentiation across parts of the cave. Most of the other finds are faunal remains, which are dominated by cave bear. A direct AMS-date on the skull resulted in a Mesolithic age (8,470±50 BP, OxA-10827) (Terberger et al. 2003). A second date was obtained from a fragment of a human humerus deriving from the same layer where the skull was found (8,400±50 BP, OxA-10828). Therefore, the recent interpretation of the human remains in the *Oberhalle* is that they are the result of burial activities in the Mesolithic, even though it is still unclear how they could have accessed the *Oberhalle* around this time (Terberger et al. 2003).

References:

Bach, H. 1974: Menschliche Skelettreste aus Kniegrotte und Urdhöhle. - In: R. Feustel, Die Kniegrotte. Eine Magdalénien-Station in Thüringen. - Veröffentl. des Museums für Ur- und Frühgeschichte Thüringens 5, 202-206. Weimar.

Feustel, R., Kerkmann, K., Schmid, E., Musil, R., Mania, D, von Knorre, D., Jacob, H. 1971: Die Urdhöhle bei Döbritz. - Alt-Thüringen 11, 131-226. Weimar.
Grimm, H., Ullrich, H. 1965: Ein jungpaläolithischer Schädel und Skelettreste aus Döbritz, Kr. Pößneck. - Alt-Thüringen 7, 50-89. Weimar.
Orschiedt, J. 1999: Manipulationen an menschlichen Skelettresten. Taphonomische Prozesse, Sekundärbestattungen oder Kannibalismus? - Urgeschichtliche Materialhefte 13. Tübingen.
Richter, M. 1955: Die jüngere Altsteinzeit im Ostthüringer Orlagau. - Alt-Thüringen 1, 11-42. Weimar.
Terberger, T., Küßner, M., Schüler, T., Street, M. 2003: Mesolithische Menschenreste aus der Urdhöhle bei Döbritz, Saale-Orla-Kreis. - Alt-Thüringen 36, 4-20. Weimar.
Ullrich, H. 1975: Bemerkungen zu den Fundumständen und zur Deutung der menschlichen Skelettreste aus der Urdhöhle bei Döbritz. - Zschr. für Archäol. 9, 307-318. Berlin.

Vasilievka I, Ukraine

Alexandra Buzhilova

Analysed individuals:

- VSL002 (11094): 11056-9909 calBP (layer)
- VSL003 (10072): 11056-9909 calBP (layer)
- VSL004 (10075): 11056-9909 calBP (layer)

Vasilievka I site was discovered in 1953 by A.V. Bodyansky on the left bank of the Dnepr River near the village of Vasilievka in the area of the modern town Dnepr in Ukraine. The excavation of the Vasilievka site was performed by A.D. Stolyar (1957). The retrieved human burials were usually single with head orientation primarily towards the east. Some skeletons were partly covered by ochre. Based on the grave artefacts, as well as parts of other flint tools from the same cultural layer, Stolyar dated the site to the Mesolithic. The skeletal remains are stored in the Museum of Anthropology of Moscow State University. A study of the skulls showed that the assemblage presents at least two anthropological variants. The first craniological variant was found also in the anthropological collection of the Voloshsky burial ground − a synchronous site that is located just 16 km from Vasilievka 1. According to Konduktorova, the presence of several craniological variants in the Mesolithic shows that the population of this territory had a different geographical origin, and that the most common craniological complex could reflect the Late Paleolithic anthropological background (Konduktorova, 1957; 1973).

References:
Konduktorova T.S. 1957. Paleoanthropological materials from the Mesolithic cemetery Vasilievka I. Soviet anthropology. No. 2. P. 189-210. (In Russian).
Konduktorova T.S. 1973. Anthropology of the Ukrainian population of the Mesolithic, Neolithic and Bronze Ages. M.: Nauka. 128 p. (In Russian).
Stolyar A.D. 1957. The Mesolithic burial ground of Vasilievka on the Dnieper. Soviet anthropology. No. 2. P. 179-189. (In Russian).

Vovnigi I, Ukraine

Alexandra Buzhilova

Analysed individuals:

- VO1001 (9479): 7562-7364 calBP
- VO1003 (9496): 7562-7364 calBP (layer)
- VO1004 (9485): 7562-7364 calBP (layer)
- VO1005 (9868): 7562-7364 calBP (layer)

The burial ground of Vovnigi I is located on the left bank of the Dnepr River in front of the village of Vovnigi in the Solonyansky district, and was discovered by Rudinsky in 1949. The numerous burials were organised in a row, densely positioned one next to another, and often with one above the other. In total, 31 burials were excavated and their skeletal remains are stored in the Museum of Anthropology of Moscow State University. The orientation of the buried individuals was along the north-south line, and head orientation was to the southeast. The burials located in the central part were richly covered by ochre while no ochre was found in peripheral burials. Studying the skeleton positions, it was suggested that the dead individuals were pulled strongly together (Rudinsky, 1955). According to Rudinsky, such a skeleton cluster can be considered as a kind of ossuary and the same author proposed that the site was formed in the Neolithic.

Reference:

Rudinsky M.Ya. 1955. Late Neolithic burial grounds of Vovnigi (on the issue of the design of Mariupol type burial grounds). Brief Communications of the Institute of Archeology of the Ukrainian SSR. Vol. 4. P. 147-151. (In Russian).

Vovnigi II, Ukraine

Alexandra Buzhilova

Analysed individual:

- VO2001 (9861): 7586-7434 calBP

The burial ground of Vovnigi II is located on the right bank of the Dnepr River in the centre of the village of Vovnigi in the Solonyansky district. In 1952, Rudyansky excavated 130 burials and, of those, the skeletal remains from 63 burials are stored in Museum of Anthropology of Moscow State University, while another part is in the Museum of Anthropology and Ethnography, RAS (Kunstkamera) in St. Petersburg. The burials located in the central part of the burial site were covered by ochre and, in a similar way as in the Vovnigi I, no ochre was found in the peripheral burials (Rudinsky, 1955). In addition, the skeleton orientation was the same as in Vovnigi I. Rudinsky noted that there are no significant archaeological finds to give a reasonably narrow chronological interval for both archaeological sites. In his opinion, both sites dated to the Neolithic. A portion of the Vovnigi II human material housed in St. Petersburg has been dated by Jacobs to ~7,100 calBP (OxA-5938, OxA-5939, and OxA-5940) (Lillie & Budd, 2011). According to craniological investigations, the Vovnigi I and Vovnigi II individuals did not present major differences, and both carry features present in Late Paleolithic European skulls (Konduktorova, 1956; Gokhman, 1966).

References:
Gokhman I.I. 1966. The population of Ukraine during the Mesolithic and Neolithic (anthropological essay). Moscow: Nauka. 195 p. (In Russian).
Konduktorova T.S. 1956. Skulls from the Vovnigi Late Neolithic burial grounds. Brief Communications of the Institute of Archeology of the Academy of Sciences of the Ukrainian SSR. Vol. 6. P. 68-71. (In Russian).
Lillie M., Budd C. 2011. The Mesolithic-Neolithic Transition in Eastern Europe: Integrating Stable Isotope Studies of Diet with Palaeopathology to Identify Subsistence Strategies and Economy Human. In: Bioarchaeology of the Transition to Agriculture, Eds. R. Pinhasi and J. T. Stock. John Wiley & Sons, Ltd. P. 43-62.
Rudinsky M.Ya. 1955. Late Neolithic burial grounds of Vovnigi (on the issue of the design of Mariupol type burial grounds). Brief Communications of the Institute of Archeology of the Ukrainian SSR. Vol. 4. P. 147-151. (In Russian).

Waulsort Caverne X, Belgium

Patrick Semal, Caroline Polet, Clemence Glas, Sébastien Villotte

Analyzed individuals:

- WCX002 (WLS-10): 10644-10311 calBP
- WCX004 (WLS-7): 10577-10305 calBP

Waulsort Caverne X is one of a series of ten caves (A, B, O, R, Q, V, W, X, Y) in the Waulsort area, Hastière, Province of Namur, Belgium. These caves were excavated from 1877 onwards at the request of Édouard Dupont (Rahir, 1925). No precise location has been preserved in the archives of the time and the various subsequent efforts to identify these caves have remained largely unsuccessful. According to Rahir (1925), all these sepulchral caves are to be related to the Neolithic. This attribution has been confirmed by direct dating on human bones (Cauwe et al., 2002) with the exception of Caverne X which gave a radiocarbon age of 10820±80 BP (OxA-6856; Cauwe et al., 2002). A following study of the anthropological material from Caverne X was carried out where several human bones were radiocarbon dated (Polet et al., 2020). The anthropological material consists of 544 human remains belonging to at least nine individuals (Polet et al., 2020). The new radiocarbon dates were performed on four left femur bones and all gave an age assigned to the Early Mesolithic (9285 ± 30 BP, ETH-74725; 9310 ± 30 BP, ETH-74726; 9340 ± 30 BP, ETH-74727; 9300 ± 30 BP, ETH-74728) (Polet et al., 2020).

References:
Cauwe, N., Orban, R. & Polet, C. Belgium. Prehistoric cave burials. In: C. Bronk Ramsey, T.F.G. Higham, D.C. Owen, A.W.G. Pike & R.E.M. Hedges (eds), Radiocarbon dates from the Oxford AMS System: Archaeometry Datelist 31. ‚*Archaeometry*, **44(3)**, supplement 1, 8-10 (2002).
Polet, C., Drucker, D.G., Glas, C., Sabaux, C., Goffette, Q., Samsel, M., Jadin, I., Warmenbol, E. & Villotte, S. Waulsort Caverne X: A new cave site with Early Mesolithic human remains in Belgium. Mesolithic Miscellany, **28(2)**, 25-43 (2020).
Rahir, E. Les habitats et les sépultures préhistoriques de la Belgique. Bulletin de la Société d'Anthropologie de Bruxelles, **XL**, 13-14 (1925).

Weyhe-Dreye, Germany

Hartmut Benthien

Analyzed individual:

- BRM001 (FWKL): 5896-5661 calBP

The analyzed femur was found in the "Blauer Werder" gravel pit, owned by the Bultmann company, in Weyhe-Dreye on the Weser River in the immediate vicinity of Bremen (Germany). A suction excavator was used to mine the gravel, whose mining was terminated around 1994. The studied right femur was found in either 1993 or 1994 after being discarded as waste and disposed of on a mound during mining. Since the site is under water now, the exact depth from where the specimen was initially retrieved is not available. The superficial morphological structure of the river marshes is made of perimarine clays and silts. Southwest of the Bremer Dune (a dune ridge that extends from the Verdener Heide over Achim to Burg-Grambke parallel to the Weser), an up to 12 m thick alluvial clay was deposited. The Pleistocene layers begin in the Bremer Marsch with the up to 30 m wide Weser sands of the Aller-Weser glacial valley (Baales et al., 2013). Numerous remains of Pleistocene mammals and human remains as well as a number of artifacts from the Upper Paleolithic were discovered probably at a depth of 12 to 14 m (Diepholz, personal communication).

Reference:

Baales, M., Pollmann, H.-O. & Stapel, B., Westfalen in der Alt- und Mittelsteinzeit, LWL, Landschaftsverband Westfalen-Lippe, Darmstadt 2013, 252 p.

Wöllersdorf, Austria

Silvia Renhart, Dorothea Talaa

Analyzed individual:

- WOL001 (Gst. 1286/30/1286/31): 8984-8606 calBP

The skull of the analysed individual was discovered during an excavation campaign at the large prehistoric site of Wöllersdorf in Lower Austria in 2011. The site is situated on the western rim of the southern part of the Vienna Basin. The isolated skull of a ~32-year-old robust male individual had been deposited in a small posthole-like pit 30 cm deep (object no. 2000). The mandible was missing but a left femur was found. The bones were well preserved due to the coarse-grained limestone gravel soil. Under the skull there was a piece of quartzite slab with barely visible traces of a red chalk mineral. This was placed directly on the floor of the pit and it is interpreted as a grave good. The skull shows traces of malnutrition, medical treatment such as a trepanation in the area of the left parietal bone and violence suffered by the individual while still alive. One of the visible injuries on the skull is a rectangular imprint indicating that the male individual was struck on the head with a blunt object, probably a wooden club-like weapon. This likely lethal attack was carried out with extreme violence suggesting that this individual was killed during an armed conflict. According to the radiocarbon dating carried out on a skull fragment in 2015 (Beta-444159), the individual is dated to the 9th millennium BP and associated with the Mesolithic. Skull burials in settlements or campsites like that at Wöllersdorf were part of secondary mortuary rites particularly common during the Mesolithic of southeastern Europe. The type of deposition suggests that the body was first buried somewhere else

and after decay, the calvarium and the femur were removed and buried apart from the postcranial skeleton (Renhart, 2020/2021, Talaa & Herrmann, 2020/2021).

References:
Renhart, S., Die mittelsteinzeitliche Schädelbestattung aus Wöllersdorf (NÖ) – Eine 9000 Jahre alte Geschichte interpersoneller Gewalt. Schild von Steier 29/2020/2021.
Talaa, D. & Ingomar, H., Die mesolithische Schädelbestattung von Wöllersdorf, Niederösterreich. Mesolithic skull burial of Wöllersdorf, Lower Austria. Schild von Steier 29/2020/2021.

Yazykovo, Russia

Alexandra Buzhilova

Analysed individual:

- JAZ001 (8619): 7315-7167 calBP

The Yazykovo 1 peat site near the village of Yazykovo in the Kashinsky district (Tver province) was discovered by B.S. Zhukov, A.E. Alikhova and M.V. Voevodsky in 1928. The researchers excavated ceramics, whose stratigraphic position indicated its greater antiquity compared to the Pit-Comb Ware culture (Zhukov, 1929). Under the main bedding layer in the peat of the Yazykovo 2 complex, the presence of the pottery complex of Yazykovo 1 was revealed. Therefore, Yazykovo 1 and 2 were among the main sites to study the oldest Neolithic periods in the Upper Volga region. These sites, as well as the later discovered Yazykovo 3 site, are attributed to the Central variant of the Upper-Volga archaeological culture (Krainov, 1978). In the framework of the expedition of the Institute of Anthropology of Moscow State University at the Yazykovo site, Zhukov in 1930 and his student Otto Bader in 1935, discovered the bones of two individuals, which were deposited in the Museum of Anthropology of Moscow State University. There is no additional information about these remains, since the documents of the expeditions have not been preserved in the archives.
At present, a detailed periodization has been developed for the Volga-Oka Neolithic. Accordingly, it is common to divide the Neolithic into early and developed periods. Sometimes the term “developed Neolithic” is understood by researchers or used as synonym of “Middle Neolithic” and “Late Neolithic”. A marker of the beginning of the Neolithic is the appearance of pottery at the sites. In fact, the presence of ceramics is so far the only evidence of the Neolithic advance in the region. The Early Neolithic period covers the period from 7,100/7,000 to 6,100/6,000 uncal BP (Engovatova et al., 1998). Instead, the developed Neolithic period spans from 6,100/6,000 to 3,800/3,700 uncal BP (Tsetlin, 2008). The transition from the Mesolithic to the Neolithic of the Upper Volga region is a complicated issue. It has been proposed that the Neolithic population from the Upper Volga region derives from groups associated with the Butovo archaeological culture (the culture of the Volga-Oka interfluves during the 10th-8th millennium BP) with contributions from southern populations (Zhilin, 1994; Krainov, 1996; Koltsov & Zhilin, 1999; Kostyleva, 2003; Tsvetkova, 2012). As Tsvetkova (2019) notes, according to the study of the stone tools, the transition from the Mesolithic to the Neolithic in the Upper Volga region should be associated with one-time contact between the autochthonous population and the groups making pottery with sparse butt-pricked ornamentation. It is likely that the first ceramics came into the region after being produced elsewhere. The absence of differences between the stone tools of the final Mesolithic and the early Neolithic in the Upper Volga region suggests that there was no massive influx of people into the region connected with this cultural transition. Moreover, once in the local environment, the tradition of making early pricked ceramics

did not have a long continuation and was interrupted by the influx of populations producing ceramics with comb ornamentation. The appearance of ceramics with sparse bonded-pricked ornamentation ~7,100/7,000–6,800 uncal BP and its subsequent distribution ~6,800–6,400 uncal BP (Zaretskaya & Kostyleva, 2008; 2011), which were not accompanied by significant changes in stone and bone tool production, can thus be considered as a transitional time between the Mesolithic and Neolithic, reflecting the process of Neolithization.

References:
Engovatova A. V., Zhilin M. G., Spiridonova E. A. 1998. Chronology of the Upper Volga Early Neolithic culture (based on the materials of the multilayer sites of the Volga-Oka interfluve). Russian archeology. 2. P. 11–21. (in Russian)
Koltsov L. V., Zhilin M. G. 1999. Mesolithic of the Volga-Oka interfluve (monuments of the Butovo culture). M.: Science. (in Russian)
Kostyleva E. L. 2003. The main issues of the Neolithization of the Center of the Russian Plain (features of the Neolithization of the forest zone). In: Timofeev V. I. (ed.). Neolithic - Eneolithic of the south and Neolithic of the north of Eastern Europe (new materials, research, problems of the Neolithization of regions). SPb.: Akademprint. P. 213–218. (in Russian)
Krainov D. A. 1978. Chronological framework of the Neolithic of the Upper Volga region. Brief reports of the Institute of Archeology. Neolithic monuments. Issue 153. P. 57–62. (in Russian)
Krainov D. A. 1996. Upper Volga culture. In: Neolithic of Northern Eurasia. M.: Science. P. 166–172. (in Russian)
Tsetlin Yu. B. 2008. Neolithic Centre of the Russian Plain. Ornamentation of ceramics and the method of periodization of cultures. Tula: Grif and K. (in Russian)
Tsvetkova N. A. 2012. Early Neolithic of the Upper Volga basin (based on the results of studying the stone industry). Brief reports of the Institute of Archeology. Issue 227. P. 271–280. (in Russian)
Tsvetkova N. A. 2019. The beginning of the Neolithic era on the Upper Volga. Bulletin of St. Petersburg University. History. T. 64. 2. P. 683–717. doi: 10.21638/11701/spbu02.2019.215 (in Russian)
Zaretskaya N. Ye., Kostyleva E. L. 2008. Radiocarbon Chronology of the Initial Stage of the Upper Volga Early Neolithic Culture. Russian archeology. 1. P. 5–14. (in Russian)
Zaretskaya N. E., Kostyleva E. L. 2011. New data on the absolute chronology of the Liyalovo culture. In: Tver archaeological collection. Issue 8: Sat. Art. Tver. Tver Regional Museum of Local Lore. P. 175–183. (in Russian)
Zhilin M. G. 1994. Some questions of the transition from the Mesolithic to the Neolithic on the Upper Volga. In: Utkin A. V. (ed.). Problems of studying the era of primitiveness and the early Middle Ages of the forest zone of Eastern Europe. Ivanovo: GU KPK Mintopenergo. P. 19–31. (in Russian)
Zhukov B. S. 1929. The theory of chronological and territorial modifications of some Neolithic cultures of Eastern Europe according to the study of ceramics. Ethnography. 1. P. 54–77. (in Russian)

Yuzhniy Oleniy Ostrov, Russia

Vyacheslav Moiseyev, Kristiina Mannermaa, Rick Schulting

Analyzed individuals:
- UOO004 (5773-26_Grave71): 8167-7939 calBP
- UOO012 (5773-17_Grave65): 8250-8000 calBP (layer)
- UOO015 (5773-126_Grave161): 8250-8000 calBP (layer)

- UOO023 (5773-87_Grave144): 8250-8000 calBP (layer)
- UOO025 (5773-41_Grave94): 8250-8000 calBP (layer)
- UOO029 (5773-92_Grave127): 8250-8000 calBP (layer)
- UOO033 (5773-114_Grave153): 8382-8196 calBP
- UOO035.056 (5773-5_Grave59): 8287-8024 calBP
- UOO037 (5773-4_Grave53): 8250-8000 calBP (layer)
- UOO047 (5773-6_Grave55): 8341-8039 calBP
- UOO049 (5773-107_Grave163): 8028-7876 calBP
- UOO051 (5773-148_Grave47): 8250-8000 calBP (layer)
- UOO052 (5773-147_Grave46): 8250-8000 calBP (layer)
- UOO053 (5773-136_Grave31): 8250-8000 calBP (layer)
- UOO059 (Grave72or75): 8250-8000 calBP (layer)

The site Yuzhniy Oleniy Ostrov on the Onega Lake, Karelia (Russia) was discovered in the 1920s. A total of 177 burials were discovered through excavations and archeological examinations during the 1930s and 1950s led by Soviet archaeologists (Gurina, 1956). This Mesolithic site is of particular importance in northwestern Russia for the various mortuary features and associated grave goods. The burials at Yuzhniy Oleniy Ostrov have been radiocarbon dated to a narrow period overlapping the 8,200 calBP cooling climatic event (Schulting et al., 2022). The Kunstkamera Museum in St Petersburg, Russia currently hosts most of the skeletal remains and artifacts from Yuzhniy Oleniy Ostrov. Three studies have reported nuclear genomic data from this site (Haak et al., 2015, Fu et al., 2016, Mittnik et al., 2018). The following individuals have been genetically analyzed in this study, with the anthropological description given according to Gurina (1956) and specified with later observations by K. Mannermaa.

Grave 31: The skeleton of a mature male lay stretched on its back with his head oriented to the east. There is a small amount of ochre. Few artifacts found in the burial include two perforated bear canines, a fragmented bone point, and a flat pebble all found on the left side of the chest.

Graves 46-47: The grave contains a double burial of an adult male and an adolescent individual. Both skeletons lay parallel to each other stretched on their back with the heads oriented to the east slightly deviating to the south. There is a significant amount of ochre, especially at the bottom of the grave pit. The grave pit was covered by several stones. The inventory from the area of both skeletons includes bear canines and plates made of beaver teeth. Additionally, skeleton 46 was accompanied by two bone arrow points. The inventory of skeleton 47 (10-12 years, male according to DNA-analysis) includes a big slate knife, 11 flint arrow points, 21 pendants made of elk teeth, and several pendants made of hyoid bones.

Grave 53: The skeleton of an adult female lay stretched on its back with its head to the east with slight deviation to the north. A small amount of ochre is present in the burial. The only inventoried artifact found in the grave is an elk incisor.

Grave 55: This skeleton belonging to an adult female was found in a triple simultaneous burial. All three skeletons lay parallel to one another and stretched with their head oriented to the north-east, but their positions were different. While the central skeleton of a mature male (individual 56) lay on its back, the other two individuals morphologically defined as females lay on their right (individual 55) and left (individual 57) sides. The grave pit was covered by stones. Female 55 had the poorest inventory kit of all three burials, which included 18 plates made of beaver incisors, an elk incisor, bone point, a fragment of bone craft artifact, and 13 tubular bird bones.

Grave 59: This mature male was buried stretched on his back with his head oriented to the east. The skeleton was densely covered with ochre. One hundred thirteen pendants made of elk incisors were

found along the whole skeleton with the highest concentration in the neck and pelvis areas. The inventory kit also included a slate knife, a dog canine, and a plate made of a beaver tooth.
Grave 72-75: The sample was found among the animal bone collection by K. Mannermaa and confirmed as human by A. Zubova. The exact number of individuals is unknown. Morphological definition of sex or age estimation is not possible.
Grave 144: This poorly preserved skeleton of a mature female covered by ochre lay stretched on its back with the head to the east with slight deviation to the north. The inventory consists of three perforated pebbles, bone craft and bone pendant, four elk and two wolf teeth, a plate made of a beaver tooth and a bird bone.
Grave 127: The skeleton of an adult female lay stretched on its back with the head to the east with slight deviation to the south. The badly preserved bones were densely covered by ochre. A total of 90 elk incisors were concentrated in the pelvis area and near the knees and were likely part of a garment decoration. The inventory kit also included plates made of beaver incisors, one beaver tooth, animal bones and a fragment of craft artifact.
Grave 153: Two skeletons of mature individuals 152 and 153 were found in a double burial made in a single pit grave. Both skeletons lay stretched on their back with their heads to the north-east. Individual 152 is morphologically female; individual 153 is morphologically and genetically defined as male. While the female's inventory kit consists only of a bone piercer, the male skeleton is accompanied by more numerous inventory objects, namely a large antler craft with an elk head at the end, elk incisors and molars, and bone points.
Grave 163: This is a poorly preserved skeleton of an adult male individual, which lays stretched on its back with the head oriented to the east with slight deviation to the south. The bones were covered with ochre. No artifacts were found in this grave.

References:
Fu, Q., Posth, C., Hajdinjak, M., Petr, M., Mallick, S., Fernandes, D., Furtwängler, A., Haak, W., Meyer, M., Mittnik, A. and Nickel, B., 2016. The genetic history of ice age Europe. *Nature*, 534(7606), pp. 200-205.
Gurina, N.N., 1956. Oleniostrovskiy mogil'nik. Materialy i issledovaniya po arkheologii SSR. V. 47. Moskva-Leningrad: Academiya Nauk SSR.
Haak, W., Lazaridis, I., Patterson, N., Rohland, N., Mallick, S., Llamas, B., Brandt, G., Nordenfelt, S., Harney, E., Stewardson, K. and Fu, Q., 2015. Massive migration from the steppe was a source for Indo-European languages in Europe. *Nature*, 522(7555), pp. 207-211.
Mittnik, A., Wang, C.C., Pfrengle, S., Daubaras, M., Zariņa, G., Hallgren, F., Allmäe, R., Khartanovich, V., Moiseyev, V., Tõrv, M. and Furtwängler, A., 2018. The genetic prehistory of the Baltic Sea region. *Nature communications*, 9(1), pp. 1-11.
Schulting, R.J., Mannermaa, K., Tarasov, P.E., Higham, T., Ramsey, C.B., Khartanovich, V., Moiseyev, V., Gerasimov, D., O'Shea, J. and Weber, A., 2022. Radiocarbon dating from Yuzhniy Oleniy Ostrov cemetery reveals complex human responses to socio-ecological stress during the 8.2 ka cooling event. *Nature Ecology & Evolution*, 6(2), pp. 155-162.

Section 2: ROH contamination estimation

Several methods estimate contamination in aDNA by screening the haploid male X chromosome for heterozygosity introduced by contamination [1–3]. In principle, runs of homozygosity (ROH) on the autosomes are effectively haploid and thus they could be used for estimating aDNA contamination similar to the male X chromosome, but for individuals of both sexes. Generally, ROH long enough for detection are rare. They occur in greater numbers only in offspring of close relatives or populations with small effective population sizes (such as hunter-gatherers). In principle, such individuals frequently have enough detectable ROH to estimate autosomal contamination. However, contamination interferes with detecting ROH. False positive ROH would create upward bias of contamination estimates. Therefore, a careful co-evaluation of ROH detection and estimating contamination is needed.

Model to estimate contamination from ROH

Here, we combine two recently developed methods, hapROH [4] and hapCon [3], to infer long ROH and estimate contamination rate for aDNA samples with sufficient ROH. The software hapROH identifies ROH by using the Li&Stephens haplotype copying model [5] with a reference panel. Using the same haplotype copying approach as hapROH, hapCon is designed for estimating contamination from male X chromosomes. Summarizing briefly, hapCon compares read counts to imputed genotypes, and analyzes reads discordant from the imputed allele to infer contamination (incorporating a similar mismatch model as ANGSD, [1]). Here, we extend the hapCon algorithm to run on ROH blocks detected by hapROH. The prerequisite of using this new method is that samples should have at least one long ROH block. Due to small effective population sizes, many hunter-gatherers have long ROHs [4]; therefore, the hunter-gatherer data newly generated for this study is well suited for estimating contamination from ROH.

Formally, given a list of ROH regions $\mathcal{R} = \{r_1, \cdots, r_n\}$ we run the hapCon HMM as described in [3] on each $r_i \in \mathcal{R}$. Let $\mathcal{L}_c(r_i)$ denote the HMM likelihood of contamination c on ROH r_i. Assuming each ROH block contributes independent information,
the overall likelihood becomes: $\mathcal{L}_c(\mathcal{R}) = \prod_{r_i \in \mathcal{R}} \mathcal{L}_c(r_i)$

To obtain the maximize likelihood estimate $\hat{c}$ for the contamination rate c, we numerically maximize this composite likelihood $\mathcal{L}_c(\mathcal{R})$ with respect to c using the L-BFGS-B [6] subroutine provided in SciPy [7].

$$\hat{c} = \mathrm{argmax}_c \mathcal{L}_c(\mathcal{R})$$

We approximate the standard error for $\hat{c}$ by numerically calculating the Hessian of $\mathcal{L}_c(\mathcal{R})$ at $\hat{c}$ using the Python package numdifftools. Confidence intervals for $\hat{c}$ are then obtained by $\hat{c} \pm 1.96 \times$ standard errors, as described in [3].

Validation on simulated contamination

As a proof of concept that inferring contamination from ROH is feasible, we simulated contaminated BAM files using I4432 (a Latvia hunter-gatherer, 6072-5920 calBCE, whole genome sequenced to average depth 21.23x) as the endogenous source and I5319 (Alaska, USA, 1050- 1400 calCE, whole genome sequenced to average depth 21.34x) as the contamination source. Data for both samples are obtained from Allen Ancient Genome Diversity Project (https: //reich.hms.harvard.edu). We down-sampled both BAM files and mixed reads to create a simulated individual with desired genome-wide coverage and contamination rate. We first called ROH blocks on I4432 using hapROH with the 1240k reference panel on the original high coverage BAM file. This analysis obtained five ROH blocks, with a total length of 53.03cM. To avoid false positive ROH and inaccurate ROH boundary driving up contamination rate estimate, we only used ROH blocks longer than 5cM and additionally trimmed both ends by 0.5cM. After this pruning, we obtained four ROH blocks with a total length of 48.99cM for contamination estimation. This set of ROH inferred from high coverage data then served as the basis for downsampling experiments - testing the principal coverage limits of ROH based contamination estimates. We then ran hapCon on these ROH regions using the 1240k reference panel. Our results show that, when about 50cM ROH locations of ROH blocks are known, this approach can robustly estimate contamination rate with little bias down to ca. 0.1x genome-wide average coverage when using 1240k data (Fig. S2).

Interference of contamination with calling ROH

Despite this encouraging performance of estimating contamination on known true ROH, there are several caveats in practice. Most importantly, unambiguous ground truth ROH blocks are usually not available because contamination interferes with ROH detection. State of the art methods for ROH detection require at least 0.3x coverage to detect ROH in 1240k capture data and some uncertainty on inferred ROH and their exact genomic positions remains for such low coverage [4].

To evaluate how contamination interferes with identifying ROH in the low coverage regime, we performed simulation to estimate false positive rate and power for detecting ROH with hapROH. We simulated haplotype data with five blocks of ROH, each with length 8cM, as described in the supplementary material in Ringbauer et al., [4], using CHB (Han Chinese in Beijing, China) reference haplotypes from 1000Genome dataset [8]. We then draw read counts from a Poisson distribution with mean equal to the target coverage multiplied by a factor λ_i specific for each SNP *i*. This weighing factor models that in 1240k capture data some sites have a higher chance to be covered than others. We estimated λ_i by comparing site coverage to genome-wide average coverages in all male ancient samples in Olalde et al., [9]. Contaminant reads are drawn according to the CEU (Northern Europeans from Utah) allele frequency in the 1000 Genome dataset. To simulate read genotype error, we flipped the genotype of every read to the other allele with probability 0.01. As for the reference panel for inference, we used all 1000 Genome haplotypes excluding CHB. To identify ROH, we updated the emission model to the one used in hapCon which explicitly models contamination rate (described in detail in

Huang and Ringbauer [3]).

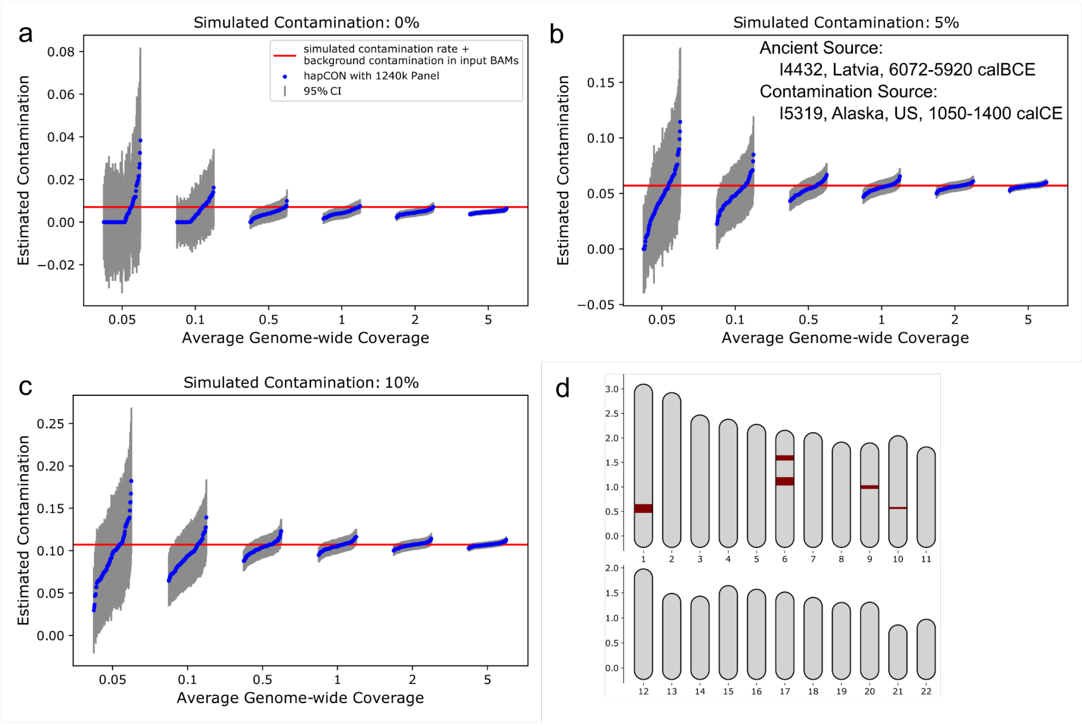

Figure S2. Estimating contamination rate from ROH (using position of ROH inferred from high-coverage data). We simulated three contamination rates (0%, 5%, and 10%) for a range of genome-wide coverages (0.05-5x average depth), and then estimated contamination rate within known ROH blocks using the 1240k reference panel. We added the baseline contamination of I4432 (0.709%, 95% CI:0.644%- 0.774%, estimated by ANGSD [1]) to the simulated contamination rate to establish the expected contamination (red lines). We visualize contamination estimates from each replicate (blue points), with estimates ordered from low to high within each group of 100 independent replicates. The grey vertical bar shows the 95% confidence intervals computed by 1.96*standard error obtained from bootstrapping for ANGSD and obtained from second derivative at minima for hapCon. **(a)** No added contamination. **(b)** 5% simulated contamination. **(c)** 10% simulated contamination. **(d)** Genomic distribution of ground truth ROH blocks of I4432, with genetic distances measured in Morgan. The ROH on Chromosome 10 is shorter than 5 cM and therefore not used when estimating contamination.

We used two metrics, positive predictive value (PPV) and sensitivity, to evaluate ROH detection with simulated contamination. First, PPV is the fraction of all inferred ROH blocks that overlap with any of the simulated true ROH blocks. Second, sensitivity is the fraction of all simulated ground truth ROH blocks that are covered by inferred ROH blocks. We found that in the presence of substantial contamination, assuming no contamination during ROH detection leads to substantially reduced sensitivity to detect ROH blocks, especially in the low-coverage regime (Fig. S3) and for substantial contamination (ą“5%). In contrast, setting

the contamination parameter in the emission model to 5% seemingly provides a good balance between PPV and sensitivity across our simulated contamination ranges. When the sample is not or only moderately contaminated (~5%), the PPV stays high with good sensitivity. When the sample is substantially contaminated (up to 15%), the sensitivity drops, but there remains some power to identify ROH blocks with high PPV. Setting the contamination parameter greater than 5% can substantially boost power for highly contaminated samples; however, this setting comes with a substantial drop in PPV, which is undesirable for estimating contamination as false positive ROH drives up contamination estimate.

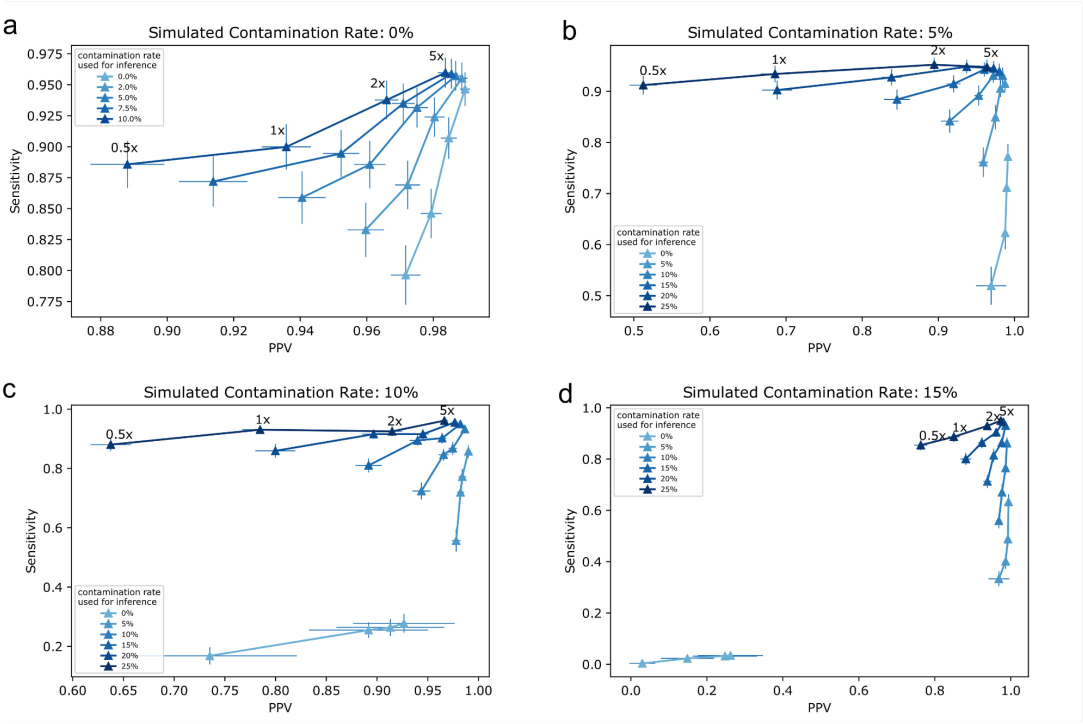

Figure S3. PPV and sensitivity trade-off for ROH detection with contamination. We simulated read count data on 1240k target sites for a range of contamination (0%,5%,10%,15%) and of coverage (0.5x,1x,2x,5x). We then identified ROH using the emission model that explicitly models contamination. For a range of specified contamination rates, we recorded the PPV and sensitivity of ROH detection. Note that the range of specified contamination rate used for inferring ROH without simulated contamination is different from the rest. For each simulated scenario, 100 independent replicates were generated. Each triangle represents the mean (taken over 100 replicates) of PPV and sensitivity for each combination of coverage and specified contamination rate. Horizontal and vertical error bars represent 95% CI for PPV and sensitivity, respectively.

Jointly calling ROH and estimating contamination

Based on the above observations, we designed an "Iterative ROH" approach to jointly infer ROH and estimate contamination, which applies hapROH and hapCon iteratively. In the first step, we infer ROH blocks with hapROH using an emission model set to 5% contamination.

In the next step, we estimate contamination from these inferred ROHs. If the inferred contamination rate exceeds 5%, we repeat this iterative procedure using the inferred contamination rate until either two consecutive contamination estimates do not differ by more than 0.5% or a maximum number of five iterations is reached. We only used segments longer than 5cM and trimmed both ends by 0.5cM.
For comparison, we also implemented a direct approach as a baseline. In this “Direct ROH" approach, we infer ROH blocks using hapROH assuming no contamination, and we then estimate contamination on these inferred ROH blocks using hapCon. Again, we only used segments longer than 5cM and trimmed both ends by 0.5cM.
Our results show that, compared with the direct ROH approach, the iterative ROH approach has substantially higher power for estimating contamination in highly contaminated samples (Table S1). The direct ROH approach tends to slightly underestimate contamination (Fig. S4) while the iterative ROH approach yields largely unbiased estimates. We hypothesize that this is because the direct ROH approach detects ROH regions by assuming no contamination, it tends to be overly conservative and therefore biases towards ROH blocks (or parts of them) with less than average contamination. Based on our simulations, we consider our iterative approach useful for estimating contamination rate for samples with up to 15% contaminated with sufficient ROHs.
For samples with more than 15% contamination, applying hapROH by setting the contamination parameter to be 5% in the first step is still insufficient for identifying ROHs, if there are any. However, setting the contamination rate higher than 5% risks producing false positive ROHs for minimally contaminated samples, which would falsely drive up contamination estimates. Overall, we believe 5% provides a good trade-off between power and false positive rates for most aDNA samples.
Notably, we found that for higher contamination levels (e.g, 15%) the power of our method decreases for samples with high coverage (Table S1). This counter-intuitive behavior is plausibly caused by the simple binomial readcount model being a poor approximation to high coverage aDNA data - reducing the power to infer ROH. We therefore recommend downsampling data to ~1x coverage for samples with > 1x coverage in practice. We have implemented this procedure within our code to perform in-place downsampling.

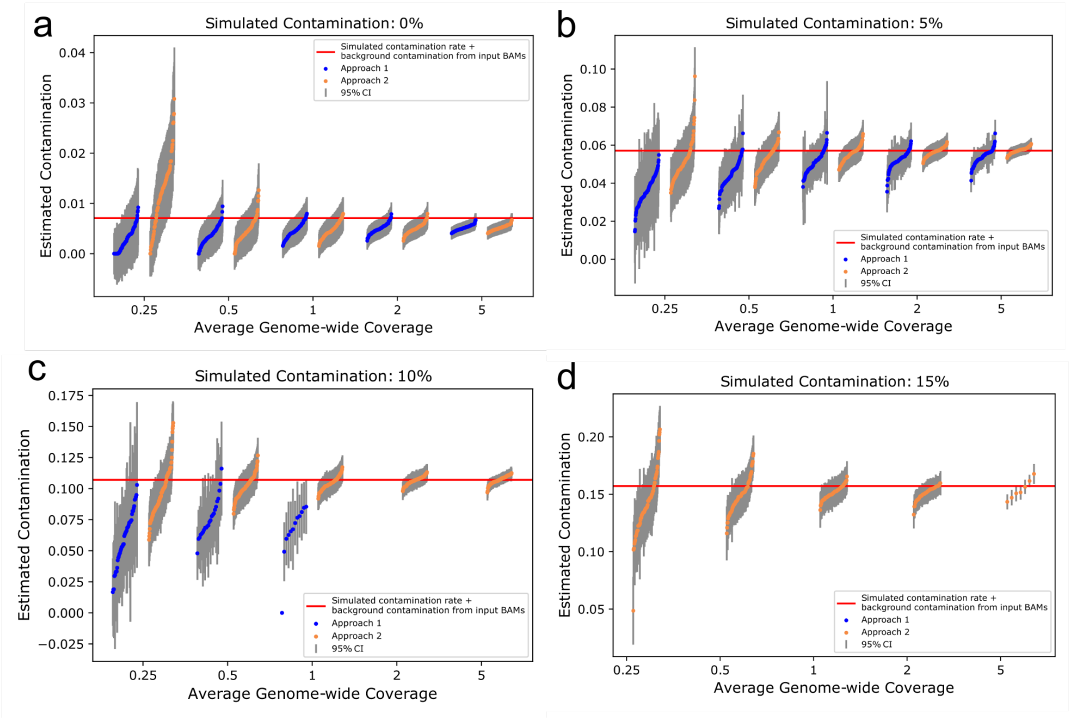

Figure S4. Comparing two approaches for estimating autosomal contamination on simulated contaminated BAM files. We simulated 100 independent replicates for each combination of a range of contamination levels (0%,5%,10%,15%) and of genome-wide coverages (0.25x,0.5x,1x,2x,5x), created as described previously. We visualized contamination estimates from approach 1 (blue) and approach 2 (red) side by side. For high levels of contamination, sometimes no ROH are identified. Our approach cannot provide estimates for these replicates, and no results were shown then. Therefore, for some combinations of contamination and coverage, the numbers of estimates (dots) are less than 100, or none at all. The numbers of replicates in each simulated scenario for which we can obtain contamination estimates are summarized in Table S1. The grey vertical bar shows the 95% confidence intervals computed by 1.96*standard error obtained from second derivative at minima.

Effects of Post-mortem Damage on Contamination Estimates

In this section, we investigate the effects of aDNA characteristic post-mortem damage on our ROH-based contamination estimates. Similar to the simulations in Huang and Ringbauer [10] (Supplementary Section 2.3.4), we estimated empirical damage rates along empirical aDNA sequences. For this we applied the software mapDamage2.0 [11] on La Braña (~7800 calBP, double-stranded library) [12], Zlatý kůň (~45,000 calBP or older, sample age estimated from Neanderthal introgression segment length, single-stranded library) [13] and Bacho Kiro CC7-335 (46-43 cal BP, single-stranded library) [14]. All three samples are non-UDG treated, i.e. no damage removed by enzymatic treatments. The inferred terminal C->T rates range from 15% to over 35%, representing damage profiles of highly damaged aDNA samples of various

preservation status and age. To simulate different contamination and post-mortem damage levels, we used S Papuan-3 and B French-3 as the endogenous and contamination source, respectively. The Papuan individuals harbor moderate amount of ROH blocks (on average 50cM total ROH blocks) [4] comparable to hunter-gatherer groups; therefore, they are a well-suited test case for our method. Both samples originate from the Simons Genome Diversity Project [15]. As in Huang and Ringbauer [10], we used the software Gargammel [16] to add aDNA characteristic damage to sequences from S Papuan-3 according to the estimates from the three empirical ancient samples described above. We then realigned the damaged sequences using BWA (version: 0.7.17-r1198g) [17] with parameters commonly used for aDNA data (-n 0.01, -o 2, and -l 16500). We downsampled the resulting BAM files and mixed them together to the desired contamination levels and coverage. For each combination of coverage and damage profile, we created 25 independent replicates and ran our iterative approach.

Our estimated contamination rates (Fig. S5) indicate that our contamination estimates are generally robust to the presence of aDNA damage. We observe that higher levels of post-mortem damage lead to only a slight upward bias of 0-2% extra contamination. Overall, the upward bias remains tolerable in the application range and does not constitute a major obstacle for applications to empirical aDNA data, where the question is whether a sample is substantially contaminated or not. This bias is probably due to the intertwined nature of detecting ROH and estimating contamination from ROH that makes the two components more susceptible to modeling mis-specifications than their standalone counterpart. However, we believe that non-allele frequency dependent aDNA damage is not expected to substantially affect contamination estimates based on the correlation with allele frequency.

a

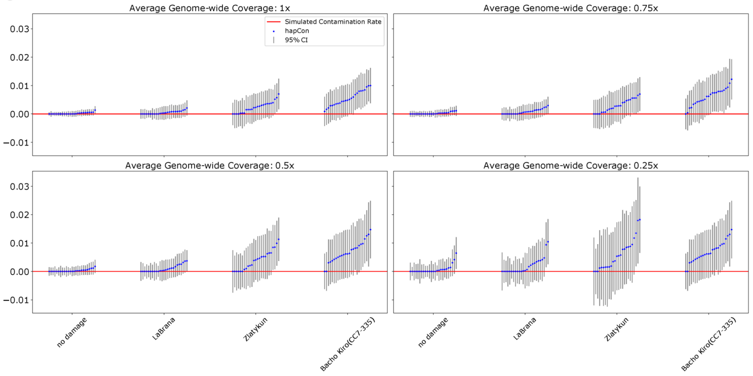

Average Genome-wide Coverage: 1x
Average Genome-wide Coverage: 0.75x
Average Genome-wide Coverage: 0.5x
Average Genome-wide Coverage: 0.25x
Simulated Contamination Rate
hapCon
95% CI
no damage
LaBrana
Zlatykun
Bacho Kiro(CC7-335)

b

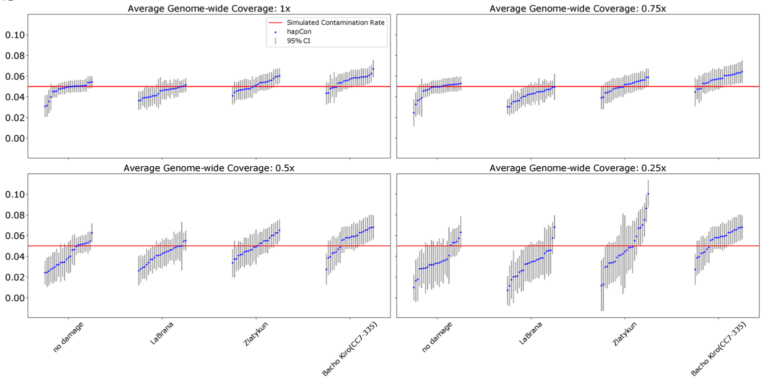

Average Genome-wide Coverage: 1x
Average Genome-wide Coverage: 0.75x
Average Genome-wide Coverage: 0.5x
Average Genome-wide Coverage: 0.25x
Simulated Contamination Rate
hapCon
95% CI
no damage
LaBrana
Zlatykun
Bacho Kiro(CC7-335)

c

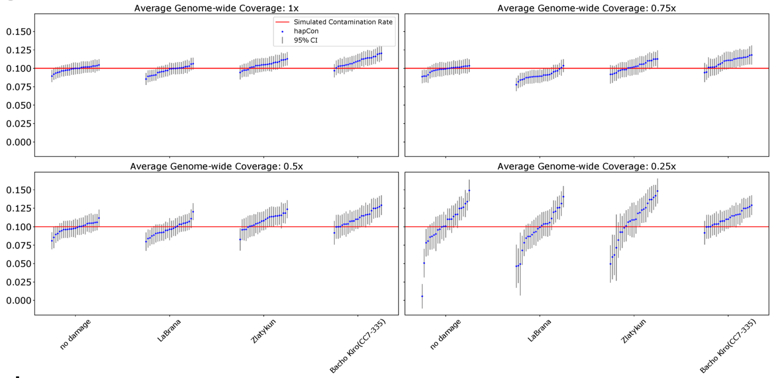

Average Genome-wide Coverage: 1x
Average Genome-wide Coverage: 0.75x
Average Genome-wide Coverage: 0.5x
Average Genome-wide Coverage: 0.25x
Simulated Contamination Rate
hapCon
95% CI
no damage
LaBrana
Zlatykun
Bacho Kiro(CC7-335)

d

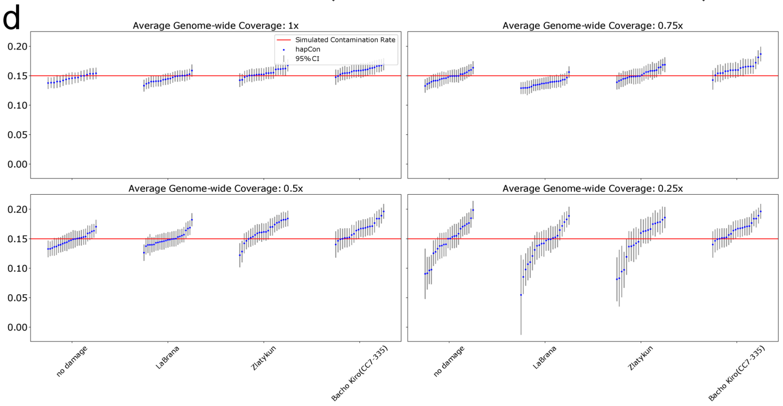

Average Genome-wide Coverage: 1x
Average Genome-wide Coverage: 0.75x
Average Genome-wide Coverage: 0.5x
Average Genome-wide Coverage: 0.25x
Simulated Contamination Rate
hapCon
95% CI
no damage
LaBrana
Zlatykun
Bacho Kiro(CC7-335)

Figure S5. Effects of Post-mortem Damage on Contamination Estimation for Simulated Data with Various Levels of Contamination. We simulated damaged sequences using empirical damage rates estimated from three aDNA samples (details described above) and visualized results of our iterative ROH approach. Compared to simulated contaminated samples without aDNA damage, post-mortem damage causes a slight upward bias of estimated contamination rates. Each simulated scenario had 25 independent replicates. The blue dots depict the MLE point estimate and the vertical grey bar represents 95% confidence intervals computed by 1.96*standard error estimated from second derivative at minima. **a** 0% contamination **b** 5% contamination **c** 10% contamination **d** 15% contamination.

Empirical comparison to ANGSD, hapCon and contamLD

Finally, we compared our ROH contamination estimate to three other methods, ANGSD [1], hapCon [3] and contamLD [18] on the new hunter-gatherer data reported in this study.

For ROH based inference, we analyzed samples with at least 300k SNPs covered as otherwise the ROH calling is subject to false positives. For ANGSD, we analyzed male samples. For both ANGSD and ROH based analysis, we used CEU allele frequency as the proxy for contamination source. A total of 19 male samples have at least one ROH and at least 300k SNPs covered and we could compare ANGSD and ROH based contamination estimates (Fig. S6). The R^2 value is relatively low (0.538), potentially for the following two reasons. First, the ratio of contamination on the X chromosome and on the autosomes are expected to vary depending on the sex the contamination originates from. If a male sample is contaminated by a female sample, then its X chromosome contamination is expected to be up to twice as high as its autosomal contamination. Therefore, the ratio of the X chromosome contamination rate and the autosomal contamination rate is expected to be fall anywhere between 1 and 2, depending on the genetic sex makeup of the contamination source. The correlation *R2* is calculated by finding a single best-fitting line, so it cannot take into account this complicated dosage effect when summarizing the general concordance between ROH and X-chromosome based estimates. Second, ANGSD has relatively large variance with low coverage samples, which is the case for many of the samples here. Given these two caveats, our results indicate that the ROH-based autosomal contamination estimates correlate generally well with ANGSD's X-chromosome based contamination estimate.

Since hapCon performs better than ANGSD in the low coverage regime [3], we also compared hapCon to our ROH contamination estimates on the same set of male samples as we did with ANGSD. We obtain a much higher correlation between the estimates of these two methods (R^2=0.863, Fig. S7), indicating that ROH regions can provide reliable contamination estimates for samples with sufficient ROH blocks (e.g., ą 25cM).

For contamLD, we used its damage correction mode and CEU as the reference panel. A total of 38 samples have at least one ROH and at least 300k SNPs covered and we could compare their contamination estimates of contamLD and hapCon (Fig. S8). Our results indicate that the ROH contamination estimates generally correlate well with contamLD. Despite a low value of R^2(0.384), possibly caused by large variation of contamLD estimates (with often very large CIs), we note that for 35 of the 38 samples both contamLD and iterative ROH approach

estimate them to be less than 5% contaminated, with the remaining three all estimated to be between 5%-10% contaminated by both methods (Fig. S8).

Table S1. Comparison between the direct and iterative ROH approaches to infer contamination. We show the number of replicates (out of a total of 100 independent simulated replicates) for which the two methods, direct ROH and iterative ROH, can provide contamination estimates, respectively. We tested the sensitivity of these two methods at a range of coverages (0.25x-5x) and of contamination levels (5% -15%).

Contamination	5%		10%		15%	
Coverage	Direct ROH	Iterative ROH	Direct ROH	Iterative ROH	Direct ROH	Iterative ROH
0.25x	100	100	45	99	0	90
0.5x	96	100	23	100	0	82
1x	99	100	12	100	0	75
2x	97	100	0	100	0	52
5x	99	100	0	100	0	7

Estimating contamination in ROH on all novel ancient samples

In this study, a total of 108 (71 males and 37 females) new hunter-gatherers' genetic data were reported. Among them, 70 samples (46 males and 24 females) have coverage at least 0.25x. We were able to obtain ROH contamination estimates for 53 samples (32 males and 21 females) with coverage at least 0.25x (see Data S1.D for detailed information about our estimates). We found consistent contamination estimates obtained from the direct and iterative ROH approaches. We only found one female sample (Goyet 2878-18, GOY009) to be contaminated with a value above our upper 10% cut-off (11.3%, 95% confidence interval: 10.1%- 12.5%). Notably, despite significant contamination, we believe it contains a large amount of ROH (119.68cM) and therefore it still provides useful information about the demographic history of Gravettian culture it is associated with. Nevertheless, we have restricted downstream analyses (other than ROH) to PMD-filtered data.

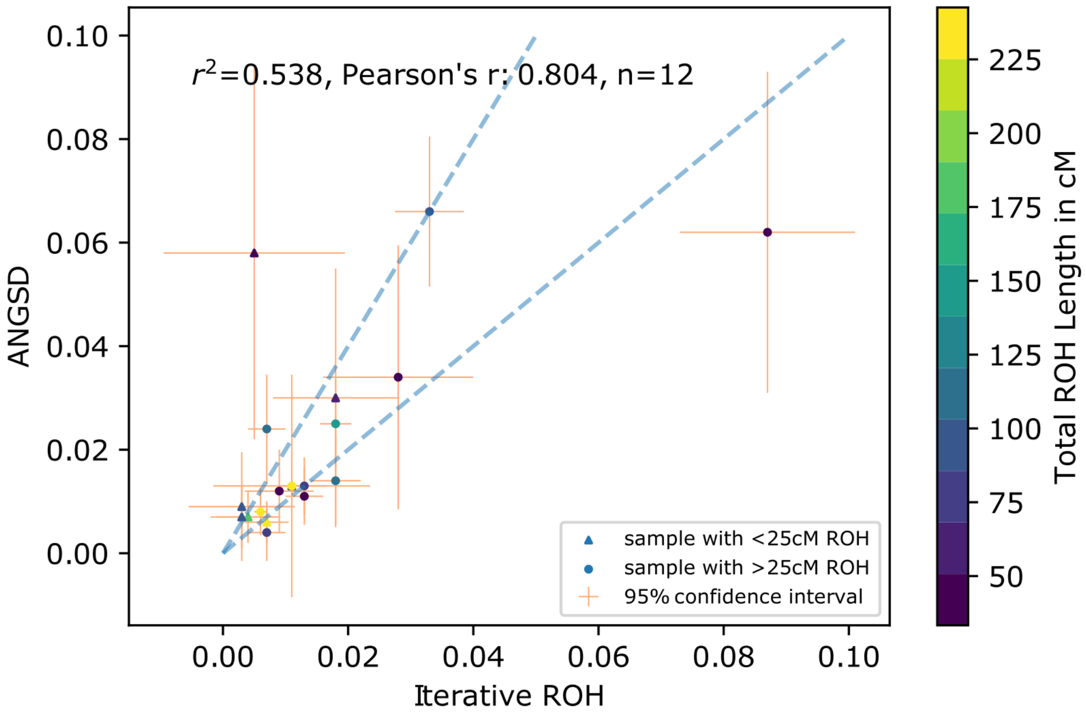

Figure S6. Comparing ANGSD and ROH contamination estimates on male hunter-gatherer samples. We compared ANGSD and ROH contamination estimates on 19 male hunter-gatherer samples with at least 300k SNPs covered. Samples with ą 25*cM* of ROH are denoted by circles and samples with ă 25*cM* by triangles. 95% confidence intervals (±1.96*standard error) are visualized for both ANGSD and ROH contamination estimates. The two dashed blue lines represent the ratios 1 : 1 and 1 : 2. The *r2* (coefficient of determination) and Pearson's correlation values are calculated only on 12 samples with at least 25*cM* of ROH.

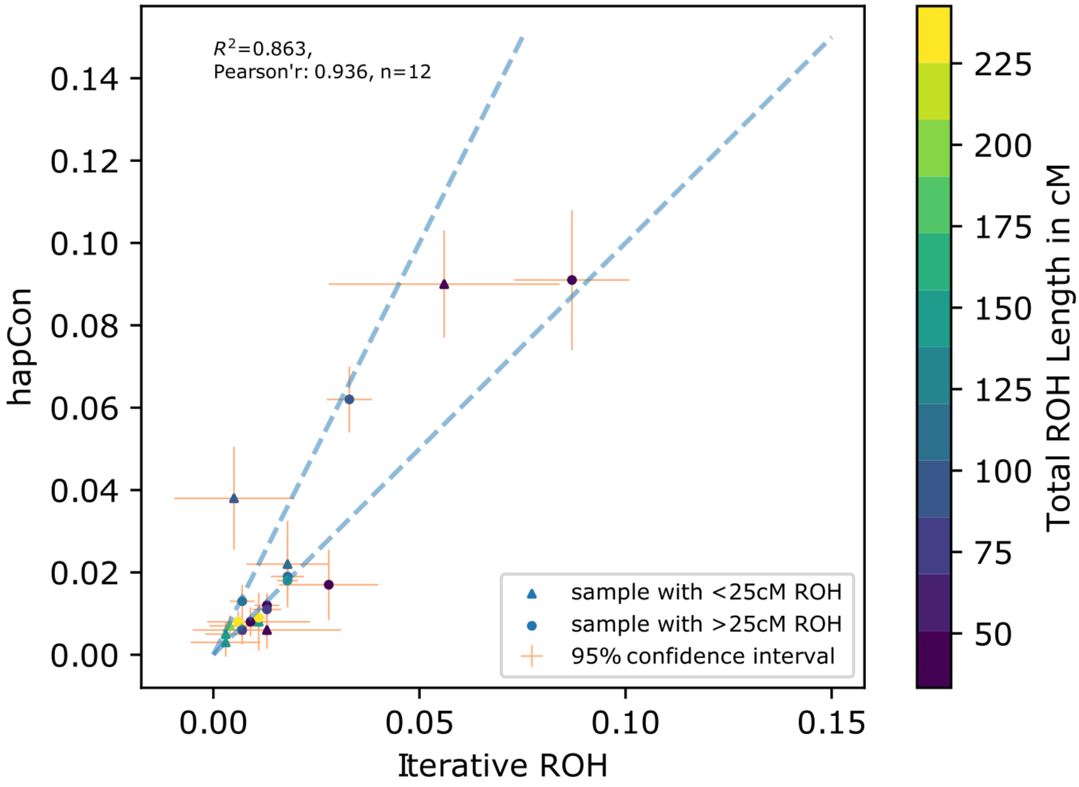

Figure S7. Comparing hapCon and ROH contamination estimates on male hunter-gatherer samples. We compared hapCon and ROH contamination estimates on the same set of male samples as in Fig. S6. All details are the same as Fig. S6 except that the y-axis shows the estimates of hapCon. 95% confidence intervals (±1.96*standard error) were shown as horizontal and vertical bars.

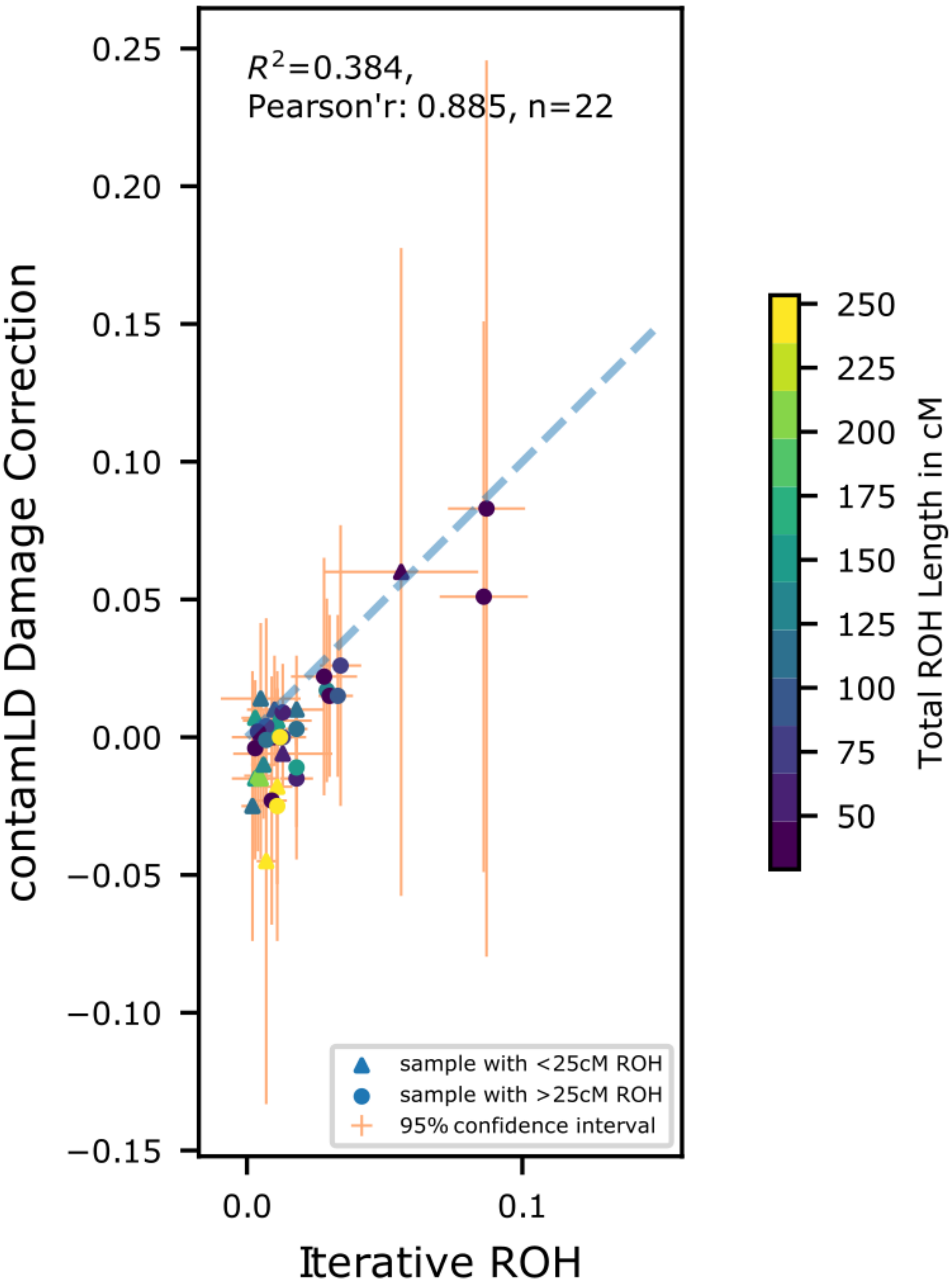

Figure S8. Comparing contamLD and ROH contamination estimates on hunter-gatherer samples. We compared contamLD (damage correction mode) and ROH contamination estimates for 38 male and female hunter-gatherer samples with at least 300k SNPs covered. Samples with ą 25cM of ROH are denoted by circles, while samples with ă 25cM are denoted by triangles. 95% confidence intervals are visualized for both contamLD and ROH contamination estimates. r2 and Pearson's correlation values are calculated only on 22 samples with at least 25cM of ROH. 95% confidence intervals (±1.96*standard error) were shown as horizontal and vertical bars.

Section 3: Ancient DNA authenticity

Damage pattern

All libraries present the damage pattern typical of ancient DNA towards the end of the sequenced molecules. Libraries produced with the single stranded library protocol show an increase in CtoT substitutions but at 3'- and 5'-ends whereas libraries produced with the double stranded library protocol show an increase in CtoT substitutions but at 5'-ends and an increase in GtoA substitutions at 3'-ends (Data S1.B).

Contamination estimations

We apply a series of methods to detect and minimize modern DNA contamination in our newly generated dataset.

First, we estimate nuclear contamination for male individuals using ANGSD 0.934 [19] and hapCon [3]. For individuals with more than one library prepared, the bam files from different libraries were merged before running the programs. For ANGSD, we filter reads for mapping quality >=30 and base quality >=30, and calculate the heterozygosity using polymorphic sites and allele frequencies in 1000 Genome CEU population, with the default HapMap file provided in the software. We select the estimation based on "Method1, new_llh" as the contamination estimation, and only individuals with >50 SNPs covered at least twice are taken into account. We use default parameters for hapCon, and the number of sites covered at least once are reported along the estimation.

Next, we estimate the nuclear contamination for all individuals, using contamLD [18] and a newly developed tool based on ROH on autosomal regions, which is introduced in the supplementary section 3. For contamLD, we filter reads in the same way as ANGSD, and use its damage correction mode (PMD score cutoff = 3) with CEU as the reference panel.

We also estimate the mtDNA contamination using Schmutzi [20], based on off-target reads from the 1240K capture and/or mtDNA capture data. The sequencing reads are first mapped to the human mitochondrial reference genome (rCRS, NC_012920.1) using BWA aln/samse algorithm and realigned with CircularMapper [21]. Then duplications are removed using DeDup [21] and reads with quality scores over 30 are used for contamination estimation in Schmutzi.

Finally, we combined the estimations from different methods to evaluate the contamination levels of the newly reported individuals (Data S1.D). Male individuals with contamination above 10% in either ANGSD or hapCon methods, and female individuals with autosomal hapCon estimates, contamLD (>0.1X coverage) and mtDNA contamination above 10% are considered substantially contaminated. Individuals with contamination below 5% in any of the abovementioned methods are considered non-contaminated. Finally, individuals with contamination levels between 5 and 10% are considered marginally contaminated and treated separately as illustrated below.

In subsequent analyses we use non-PMD-filtered genotypes for the non-contaminated individuals, and PMD-filtered genotypes for the substantially contaminated individuals. Instead, we evaluate the ten marginally contaminated individuals following a case-by-case approach. For samples that are included in groups with multiple individuals from the same site, namely DOG004 (in Doggerland, n=8 individuals), MUR002, MUR005, MUR017 (in Murzihinskiy II, n= 6 individuals) and VO1003 (in Vovnigi I-II, n=5), we carry out downstream analysis on non-PMD-filtered genotypes since marginal levels of individual contamination would be diluted within the group. GER003 is an individual from which multiple differently treated libraries were generated. Its contaminated libraries are PMD-filtered and the other libraries are not (contamination levels of different libraries are reported separately in Data S1.D). For the four remaining individuals that are analyzed ungrouped,

namely BRM001, PRD001, RIP001 and WOL001, both PMD-filtered and non-PMD-filtered genotypes are considered in downstream analyses. When the outcomes are consistent between the two genotypes (BRM001, PRD001, RIP001), in the figures we then plot the results from the non-PMD-filtered genotypes and keep both outputs in the supplementary tables (Data S2 and S3). When we find discordance between both genotypes (WOL001), we only consider the results from the PMD-filtered genotypes. Moreover, for the two marginally contaminated genomes from northern Italy (PRD001 and RIP001) we created a neighbor-joining tree based on pairwise f_2 genetic distances among all Epigravettian-associated individuals (see Methods) with both PMD- and non-PMD-filtered genotypes resulting in qualitatively comparable phylogenetic results (Fig. S9).

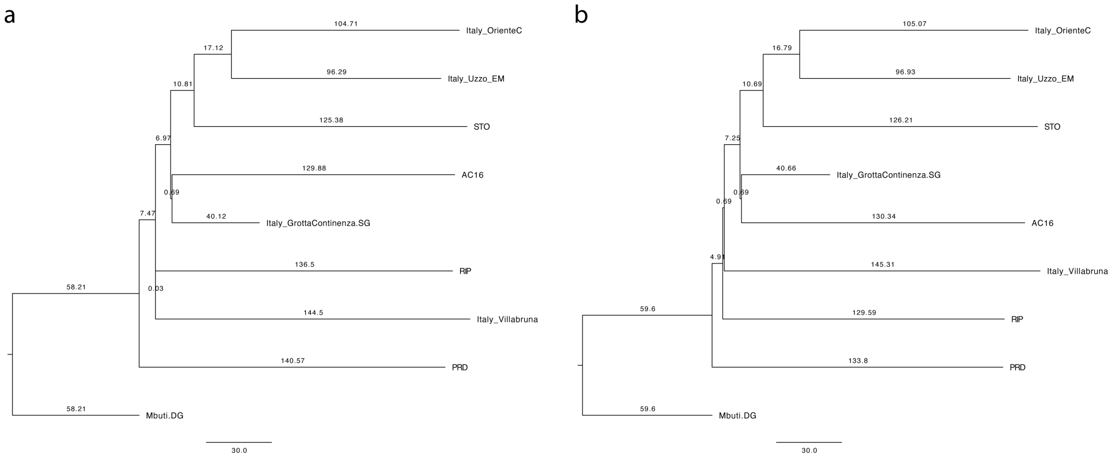

Figure S9. Neighbor-joining tree based on f_2-statistics among Epigravettian-related groups using **a)** non-PMD-filtered genotypes of PRD001 and RIP001 (related to Fig. 3B) and **b)** PMD-filtered genotypes of PRD001 and RIP001.

Finally, individuals with less than 6,000 available SNPs overlapping the 1240k panel, either PMD- or non-PMD-filtered, are removed from downstream analyses (Data S1.A). The only exception is sample Goyet Q373-3 where the hapCon contamination is not reliable due to low coverage and mtDNA contamination is estimated to 12 ± 1% (Data S1.D). In this case, we do not analyze PMD filtered data because the number of resulting SNPs is below our cut-off. However, non-PMD filtered data of Goyet Q373-3 shows high affinity in outgroup f_3-statistics to the contemporaneous Goyet Q116-1 individual (Data S2.A) justifying its use in following comparative analyses.

Assessing the potential effect of genetic attraction between contaminated samples

Considering that this study includes multiple marginally contaminated samples (5-10% contamination), we systematically test the potential attraction between samples that are contaminated with similar contamination backgrounds. Specifically, we simulate contaminated individuals by artificially introducing modern-day DNA contamination into ancient samples.

We select three non-contaminated ancient samples with high whole-genome or SNP coverage, namely Loschbour carrying *Oberkassel* ancestry, Arene Candide 16 (AC16) carrying *Villabruna* ancestry and Paglicci 12 (PA12) carrying *Věstonice* ancestry, and contaminate them with one present-day French (B_French-3) or Han (B_Han-3) genome from the SGDP dataset (Mallick et al., 2016). The Loschbour genome is contaminated at the bam file level, by mixing the endogenous and contaminant bam files in various proportions to generate a ~1X coverage genome. We then generate the pseudohaploid genotypes on 1240k SNP panel with samtools mpileup and pileupcaller as described in the Methods section. The other two individuals, which are 1240k captured, are contaminated with the same present-day individuals at the genotype level, using an in-house script

(https://github.com/TCLamnidis/ContaminateGenotypes) that randomly introduces contaminant alleles into the original genotype. In both approaches, we simulate contamination levels of 1%, 2.5%, 5%, 7.5% and 10% for each source/contaminant combination, and 10 replicates for each contamination level. The resulting 300 simulated genotypes are used for downstream analysis.
We first calculate outgroup-f_3 statistics in the form of f_3(original and artificially contaminated individual, European_HGs; Mbuti.DG), where European_HGs are the hunter-gatherer individuals included in this study. We find that compared to the tested original samples (Loschbour and AC16), the contaminated version shows lower values in f_3-statistics with most of the European_HGs, and the decrease tends to be more apparent for artificial samples with higher contamination levels (Fig. S10). In contrast, f_3-statistics are roughly constant between the original and the artificially contaminated samples when calculated for the four pre-40ka individuals/groups (Ust'Ishim, Zlaty kun, Bacho Kiro IUP and Oase1), in accordance with the fact that these pre-40k groups are equidistant to the original and contaminant individuals.
This analysis highlights the distinct genetic profile between European_HGs and present-day European and Asian genomes, where present-day contamination would lead to an underestimation of the genetic affinity shared by two hunter-gatherer individuals/populations.

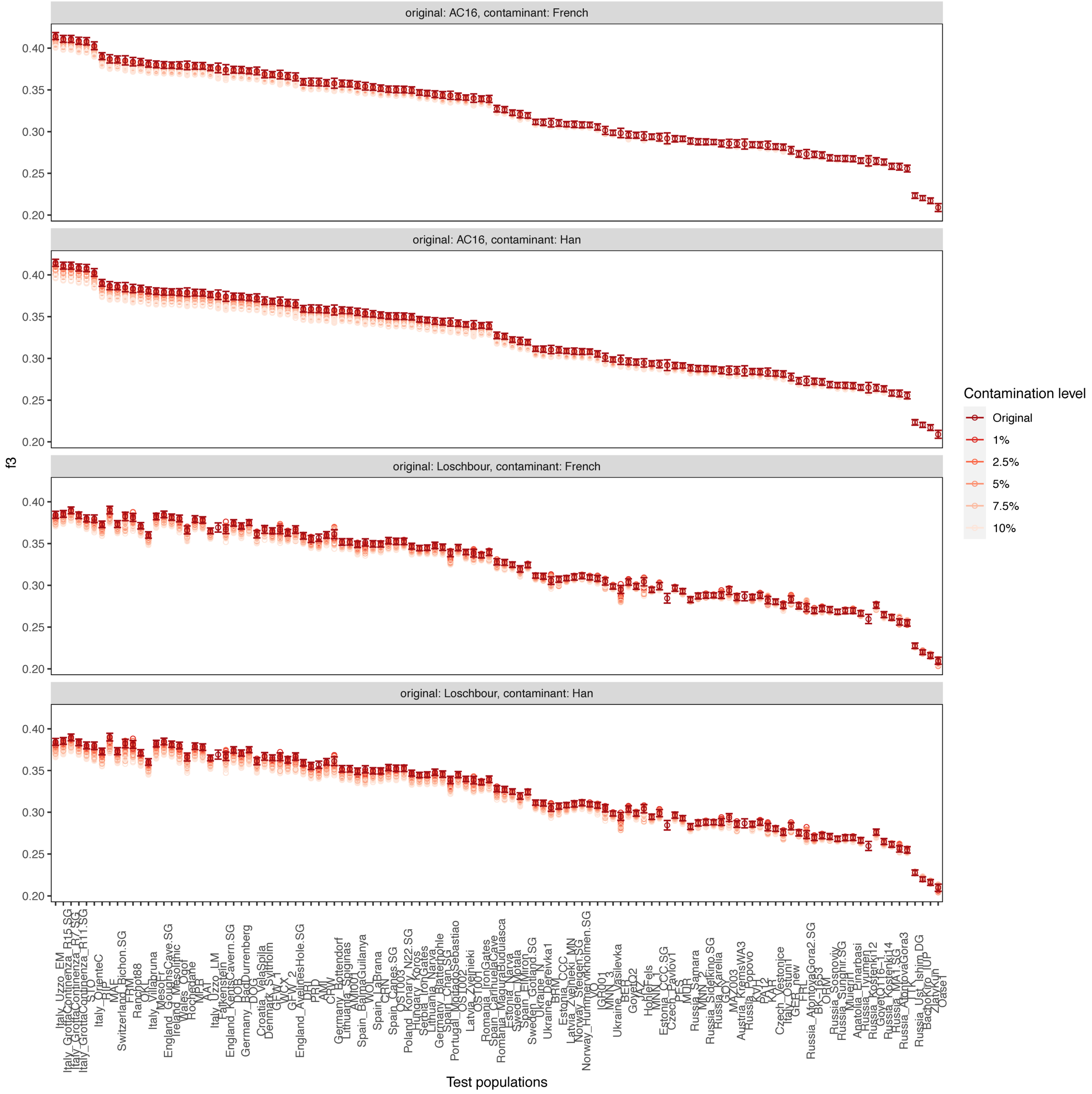

Figure S10. Outgroup-f_3 statistics showing shared genetic affinity between original and artificially contaminated samples with other European hunter-gatherers. This figure summarizes the outgroup-f_3 statistics in the form of f_3(original and artificially contaminated individual, European_HGs; Mbuti.DG) for different original/contaminant combinations. The dark red dot with error bar shows the f_3-values and 1*SE estimated using 5cM weighted block jackknife computed with original Loschbour or AC16 genotype, while the other colors show contaminated samples at different contamination levels. The x-axis reports each tested European hunter-gatherer individual/group.

We further test if two ancient individuals artificially contaminated by the same present-day individual, would be attracted to each other. With f_4-statistics in the form of f_4 (Mbuti, PA12_cont1; AC16, AC16_cont2), we test all combinations of the artificial samples contaminated at various levels with the same present-day French genome. Notably, we find that the f_4-statistics are mainly negative (Fig. S11), indicating that the contaminated PA12 individual (PA12_cont) shows higher affinity to the non-contaminated AC16 individual than to the artificially contaminated versions of AC16 (AC16_cont). The contamination levels in PA12 and AC16 also have different impacts on the resulting f_4-statistics. Higher contamination level in AC16_cont would decrease its affinity to PA12 (Fig. S11a), while

higher contamination in PA12 would decrease its affinity to AC16, resulting in elevated relative affinity to AC16_cont (Fig. S11b). However, even in the most extreme case of 10% contamination in PA12 and 1-10% contaminations in AC16_cont, the f_4-statistics are still not significantly positive, with Z-score ranging between -2.5 and 2.5 (Fig. S11b).

Similarly to the previous analysis based on f_3-statistics, also f_4-statistics suggests that, due to the large genetic distance between the ancestry carried by potential present-day contaminants and our studied hunter-gatherers, there is no an artificial over-estimation of shared genetic affinity between two contaminated samples, even when the contaminant source is exactly the same individual. To the contrary, we find that contaminated individuals would tend to show a lower affinity to other hunter-gatherers, at least for the contamination levels tested here.

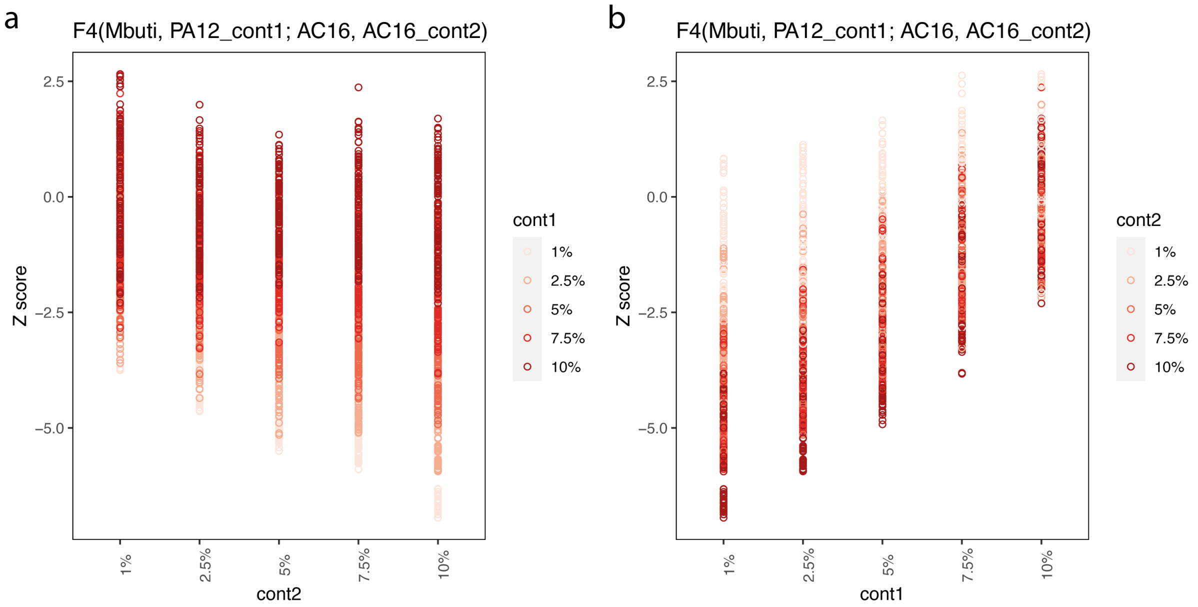

Figure S11. F_4-statistics investigating the potential attraction between contaminated samples. The two panels summarize all combinations of f_4(Mbuti, PA12_cont1; AC16, AC16_cont2) in two different ways. In the **a)** x-axis shows the contamination rate in the artificially contaminated AC16, and the dot colors show the contamination rate in the simulated PA12; **b)** x-axis shows the contamination rate in the artificially contaminated PA12, and the dot colors show the contamination rate in the simulated AC16.

Section 4: Uniparental markers and biological relatedness

Sex determination

We determine the genetic sex for newly reported individuals based on the relative coverage on sex chromosomes and autosomes. The ratio of coverage on X/Auto and Y/Auto was calculated for each library (Data S1.C) and plotted against one another (Fig. S12). We find two clusters corresponding to libraries from male individuals (in blue) and female individuals (in red). Libraries falling in between the two clusters are considered of undetermined sex, indicating potential contamination with DNA from one or multiple individuals of the opposite sex than the endogenous DNA. For libraries of undetermined sex and other contaminated ones (Section 3), we further calculate the coverage on sex chromosomes or autosomes with the PMD-filtered data in order to reduce the effects of contamination (Fig. S12b). Four of the libraries of undetermined sex are then assigned as deriving from male individuals and the other four from female individuals. We also notice that two highly contaminated libraries originally falling in the male cluster (DOG010) and four libraries originally falling in the female cluster (Paglicci 133, two RIE002, DON005) after PMD-filtering are assigned to the female or male cluster, respectively (Data S1.C).

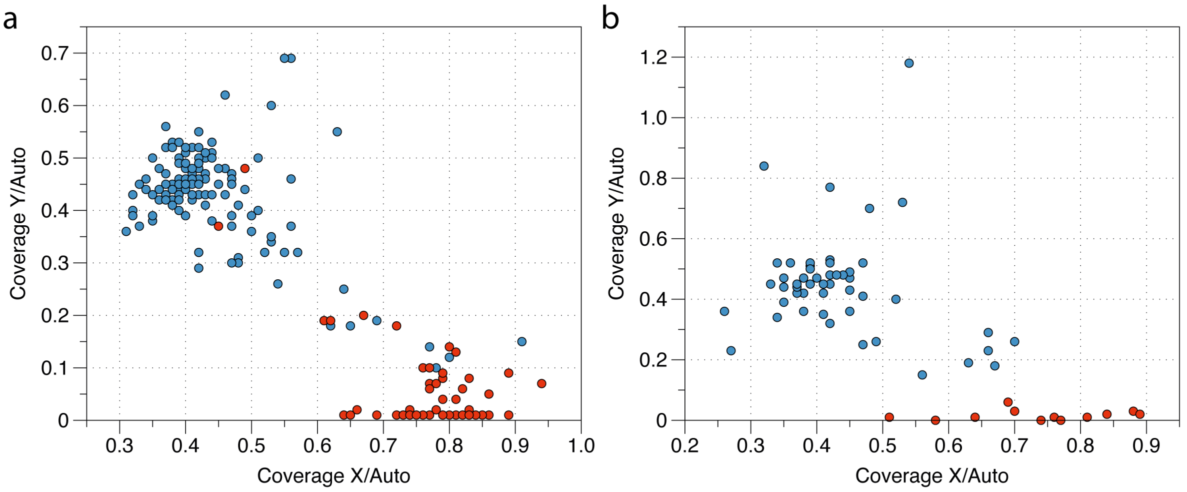

Figure S12. Relative coverages on sex chromosomes and autosomes. a coverage of non-PMD-filtered libraries from all individuals, **b** coverage of PMD-filtered libraries from contaminated individuals. The blue dots show libraries from male individuals and red dots show libraries from female individuals. The sex determination for contaminated libraries is assigned after PMD-filtering (Data S1.C).

Mitochondrial DNA haplogroups

Before 40ka, the analyzed hunter-gatherers carry basal lineages of mtDNA haplogroups M, N and R that seem to not leave descendance in later European individuals. In the *Kostenki* cluster the typical Upper Paleolithic and Mesolithic U-related haplogroups appear, which presence is predominant also in the *Věstonice* cluster. Instead, both individuals from the *GoyetQ116-1* cluster carry haplogroup M [22], which is also found at a high frequency in individuals belonging to the *Fournol* cluster. This haplogroup seemingly disappears from Europe after the LGM, with the *GoyetQ2* cluster represented by sub-haplogroup U8a and basal lineages within the U branch. The latter is also observed in the *Villabruna* cluster that, however, is dominated by haplogroup U5. This haplogroup becomes dominant in the *Oberkassel* cluster as well as in Iberian hunter-gatherers. Moving eastwards, the frequency of U5 decreases as observed in the Iron Gates HG, SHG, Baltics HG and Ukraine HG groups that show

increasingly higher frequencies of haplogroups U2 and U4. In the *Sidelkino* cluster individuals, the U5 haplogroup reaches the lowest representation among post-14ka groups, with the appearance of Asian-associated haplogroups like C1 and R1b, beside haplogroups U2 and U4 as observed for central-eastern and northeastern European populations. In Iron Gates HGs we also find a higher proportion of farmer-related mtDNA haplogroups like H and K1 (Extended Data Fig. 1 and 2, Data S1.H).

Y-chromosome haplogroups

In pre-LGM Europe, the main Y-chromosome haplogroup found is C. It is uniquely present in individuals from the *Kostenki* and *Fournol* clusters and in high proportion in *Věstonice* cluster individuals, alongside haplogroup I. The frequency of haplogroup I increases in the *GoyetQ2* and *Villabruna* clusters to become almost omnipresent in the *Oberkassel* cluster. However, in Iberian hunter-gatherers haplogroup C survives, suggesting some level of paternal continuity through the LGM in Iberia. As observed for the mtDNA haplogroups, the proportion of *Oberkassel*-associated haplogroup I decreases moving eastwards in post-14ka hunter-gatherer groups. Beside SHG, which retain uniquely haplogroup I, other northeastern as well as central-eastern populations (IronGates HG, Baltic HG and Ukraine HG) carry more individuals with haplogroup R. Interestingly, the *Sidelkino* cluster does not exhibit any haplogroup I, being represented by haplogroup R in addition to Asian-related haplogroups J and Q. Haplogroup Q is also found in low frequencies in Baltic and Ukraine HGs (Extended Data Fig. 1 and 2, Data S1.H).

Biological Kinship

We estimate the kinship relationship among individuals from the same archeological site, based on the pairwise mismatch rate (PMR) on autosomal SNPs. As the population diversity of hunter-gatherers can vary considerably in different time periods and regions, we individually calculated the PMR baseline for unrelated individuals based on their individual heterozygosity (Data S1.E). The heterozygosity of each library was first calculated as twice as much as the heterozygosity calculated from pseudodiploid genotypes. Then the heterozygosity of each individual was calculated as the weighted average of all libraries from this individual:

Het_ind = sum(nHet_SNPs_lib)/sum(nSNPs_lib)

The baseline (*b*) for unrelated individuals in the same archeological site was calculated as the average of individual heterozygosities.

We then estimate the kinship among individuals by calculating the relatedness coefficient (*r*) between individuals using PMR and the baseline of each population (Data S1.F) [23].

$$r = 1 - (2*(PMR-(b/2))/b)$$

We identify two pairs of samples deriving from the same individual: MNN001 (tooth) and MNN002 (petrous bone) from Minino I in western Russia, and Hohle Fels 10 (femur) and Hohle Fels 79 (cranium) from Hohle Fels cave in Germany, with *r* = 0.99 and 1.08, respectively. We therefore merged the data from both libraries and renamed these individuals as MNN001.002 and Hohle Fels 1079. We also identify two 1st-degree relatedness. The first pair is MUR001 and MUR007, both male individuals with the same mitochondrial haplogroup, while the coverage of MUR001 is not enough for chrY haplogroup assignment. The second pair is GFW001 and GFW007, one male and one female individual sharing the same mtDNA haplogroup. The individuals MUR001 and GFW007 are therefore removed from group-based, *f*-statistic analyses like outgroup-f_3, f_4 and qpAdm. Instead, the two pairs of individuals related to 2nd degree (GFW007-GFW002.3 and MUR017-MUR021) are not removed from group-based analyses. Finally, we show that the male individuals in the triple burial from Dolní Věstonice in Czechia (Dolní Věstonice 13, 14 and 15) are not related by close kinship (up

to 2nd degree relatedness). Generally, we observe a limited number of closely related individuals buried at the same site, but this could be influenced by the fact that most genetically analyzed individuals originate from different sites and time periods.

Section 5: Genetic clustering of European hunter-gatherers

Grouping of newly reported individuals

We group our newly reported individuals based on their sampling locations and radiocarbon dates. Individuals from different sampling locations were regarded as different genetic groups. In several cases, individuals from the same sampling site are divided into different groups based on their distinction in radiocarbon dates. For the Goyet site, individuals dated to the Gravettian period (28-26k cal BP) are grouped together as GOY (Goyet Gravettian), while Goyet Q376-3, which is dated to ~35k cal BP, is not grouped with them. This also applied to MAZ001 and MAZ003, which are ~3,000 years apart in their ages. Other sites with individuals divided into more than one group included:

- Donkalnis: two individuals dated to ~8,400 calBP and ~6,600 calBP. The younger individual is grouped with previously published Narva-associated individuals from the same site in downstream analysis.
- Ḥou Amieva: two individuals dated to ~7,100 calBP and ~6,700 calBP.
- Minino I-II: three groups dated to 11-10k calBP, ~8,500 calBP, ~7,500 calBP.
- Gross Fredenwalde: two groups dated to ~8,000 calBP and ~7,000 calBP.
- Ostorf site: three individuals dated from ~5,400 calBP to ~5,200 calBP, showing genetic distinction on their PCA positioning (see below).

Genetic clustering based on MDS plots

Considering the relatively old date of most analyzed hunter-gatherers, their genetic diversity may not be properly captured with the one of modern-day populations. We therefore calculated a MDS plot based on pairwise outgroup f_3-statistics (1-f_3(Mbuti.DG; Pop1, Pop2)) to provide an overview of the genetic variation among European hunter-gatherers.

We generate the MDS plot using 123 published and newly reported hunter-gatherer individuals or groups with over 30k SNPs covered on 1240K panel, considering the uncertainly of f_3-statistics for low coverage samples. The result is shown in Fig. 1C and the statistics are listed in Data S2.A. However, some key individuals reported in this study were of lower coverage. To compare these low coverage individuals with the described genetic clusters, we also generate an MDS plot including all 139 European hunter-gatherer groups without filtering for the number of available SNPs (Fig. S13). We find that the Aurignacian-associated Goyet Q376-3 was displaced from Goyet Q116-1, despite their sharing the highest affinity based on outgroup f_3-statistics, possibly affected by its low coverage and/or contamination. For this reason we have not grouped this individual together with Goyet Q116-1. The Gravettian-associated La Rochette individual from France falls in the MDS plot into the *Fournol* cluster together with other Gravettian-associated individuals (Fournol 85 and Serinyà group), as well as the Solutrean-associated individual Le Piage II from France. Individuals from La Marche and Pincevent also fall close to the *GoyetQ2* cluster including other Magdalenian-associated individuals. The Solutrean-associated La Riera (level 14) individual from Spain and the Magdalenian-associated Maszycka 1/1 individual from Poland do not show a consistent MDS clustering pattern as other Solutrean- or Magdalenian-associated individuals. This is probably due to both residual contamination and low coverage. These two individuals are therefore excluded from downstream analyses.

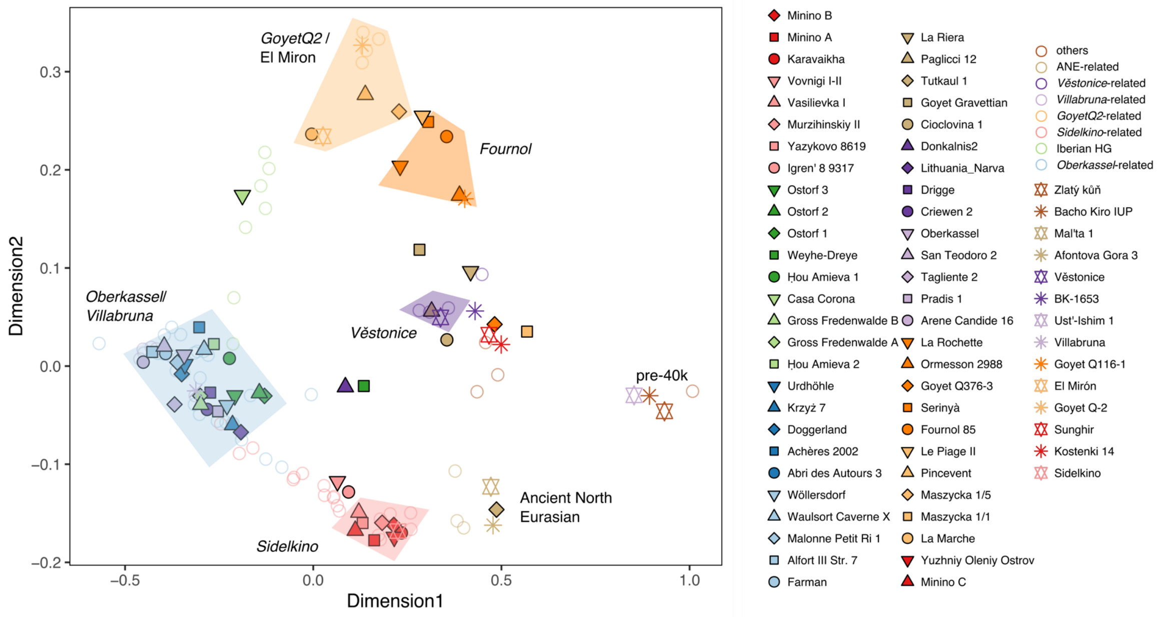

Figure S13. MDS plot of all European hunter-gatherer individuals/populations.

The clustering pattern of the MDS plot with all hunter-gatherers shown above is likely driven by post-LGM groups, which represent the majority of the analyzed dataset. Therefore, we also run MDS with only 23 pre-LGM groups to zoom in the genetic variations among those older populations (Fig. 2A). We identify three distinct genetic clusters in this pre-LGM plot, one including all the groups older than 40,000 BP (pre-40k cluster), and the other two comprising the Gravettian-associated individuals from south-western/western Europe and central-eastern/southern Europe corresponding to the *Fournol* and *Věstonice* clusters, respectively. The Gravettian-associated individuals from central-western Europe (Goyet) fall in between these two clusters. Older individuals dated to 40-32 ka fall between these three clusters, with Goyet Q116-1 being closest to the *Fournol* cluster, and Sunghir and Kostenki 12 being closest to the *Věstonice* cluster. Such different affinities of *Fournol* and *Věstonice* genetic ancestries to the preceding populations are also observed in f_4-statistics (Fig. 2, Data S2.B). The distinction between the pre-40k cluster and the younger populations in the pre-LGM MDS plot indicates a genetic discontinuity between them. However, this does not imply that the pre-40k cluster is formed by genetically homogenous groups. On the contrary, qpGraph modelling indicates that the pre-40k groups belong to different lineages (with the exception of Oase 1, whose data quality is too limited for this type of analysis) (Fig. 2). Moreover, among the identified pre-40k genetic lineages, we confirm that except for a partial genetic contribution from Bacho Kiro IUP to Goyet Q116-1, neither Zlatý kůň nor Ust'Ishim contributes to younger lineages (Fig. S21). Interestingly, previous studies have described a series of climate changes during the 42-40k BP period, which could have influenced the extinction of Neanderthals [24,25]. These climate changes could have also potentially impacted the survival of early modern human populations in Europe.

PCA on post-LGM hunter-gatherers

For the post-LGM hunter-gatherers, we also investigate their genetic ancestries on a West Eurasian PCA, which has been well characterized in previous studies (e.g.[12,26,27]). The PCs are calculated from 89 modern-day western Eurasian populations, and the ancient individuals are projected on the calculated PCs. As previous works have described, most of our newly reported individuals after 14 ka also fall into two distinct *Oberkassel* (WHG) and *Sidelkino* (EHG) clusters, while the newly produced Mesolithic and Neolithic genomes from Ukraine fall on top of the published Neolithic Ukrainian

individuals, and on the cline between the *Oberkassel* and *Sidelkino* clusters (Extended Data Fig. 6, Fig. S14) [26,28]. We also observe a shift in PC space towards Anatolian farmer ancestry in central European individuals younger than 6,000 calBP (individuals from Ostorf and Weyhe-Dreye), suggesting a substantial early Neolithic farmer-related ancestry being admixed in these recent genomes. The Tutkaul 1 individual from Mesolithic Tajikistan falls in proximity of ANE-related individuals like Afontova Gora 3, Mal'ta 1 and Botai. Epigravettian-associated individuals all fall close to the *Oberkassel* cluster, and Magdalenian-associated individuals to the *GoyetQ2* cluster individuals and El Mirón, towards the center of the PC space.

The La Riera and Maszycka 1/1 individuals are displaced from other Magdalenian- or Solutrean-associated individuals towards the direction of Anatolian farmers (Fig. S14). This could indicate either a residual contamination in these two individuals, and/or uncertainty in the positioning due to the low coverage. As mentioned before in the MDS plot section, both individuals are excluded from downstream analyses.

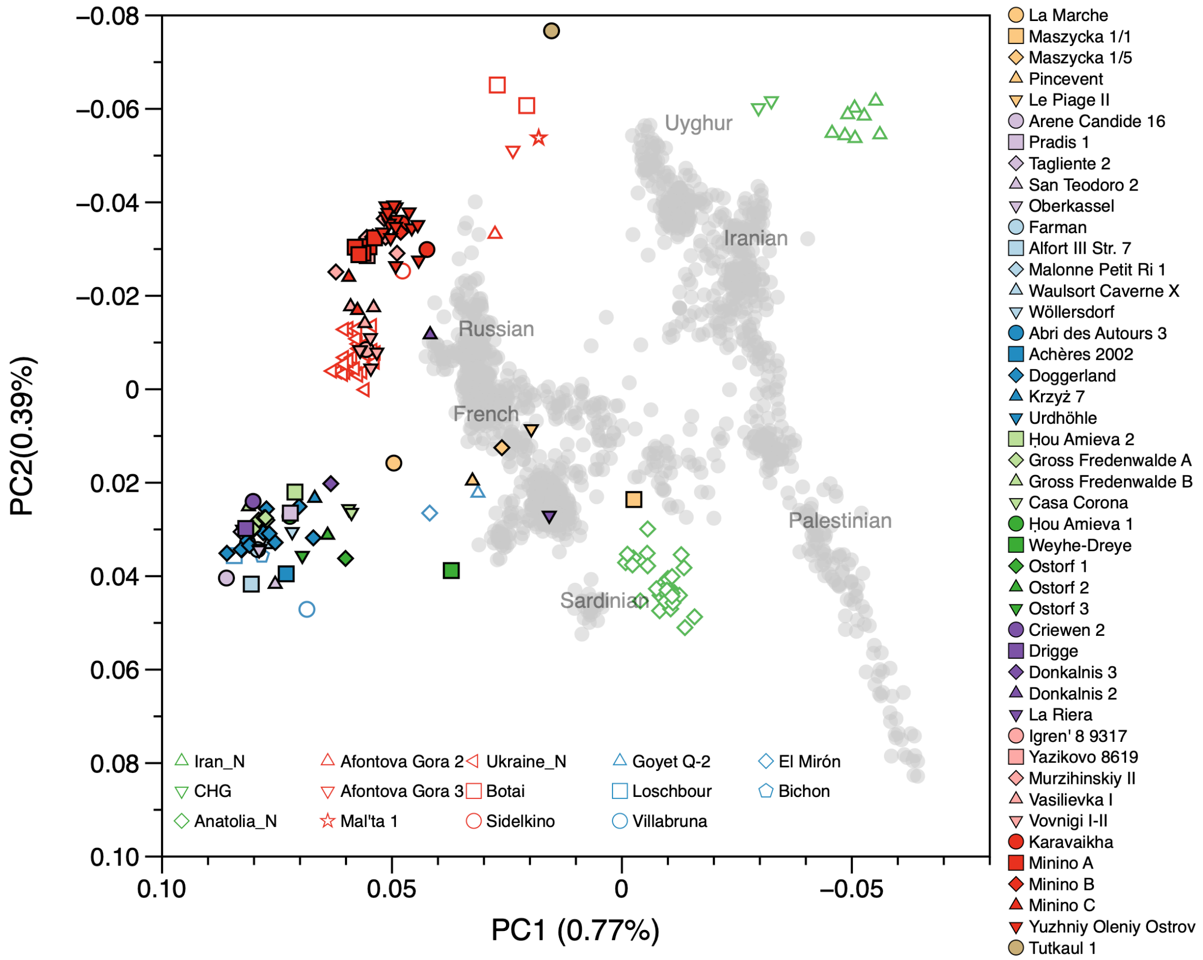

Figure S14. West Eurasian PCA of LGM and post-LGM hunter-gatherers. Present-day individuals (gray dots) genotyped on the *Human Origins* dataset are used to define the PCA variation onto which ancient genomes (colored symbols) are projected. All of the newly reported individuals are shown in outlined, filled symbols, as illustrated in the right legend, including the individuals with <15,000 SNPs on *Human Origins* dataset: La Riera (7,177), Pincevent (8,714), Achères 2002 (9,568), Donkalnis 2 (5,416), Donkalnis 3 (8,944), Maszycka 1/1 (13,506), Doggerland Sand Motor (12,554) and Murzihinskiy II burial 1 (7,507). Representative ancient genomes are shown in outlined symbols, as illustrated in the legend below the PCA.

Section 6: Neanderthal ancestry in European hunter-gatherers

Genetic introgression from Neanderthals has been detected in present-day non-African human populations, with an estimated contribution to around 2-3% [29]. The Fu et al., 2016 study reported a decrease of Neanderthal ancestry in modern humans during the last 45,000 years, supporting the hypothesis that the Neanderthal alleles underwent gradual negative selection [30,31]. However, this observation was challenged by a refined statistic, which found no significant correlation between Neanderthal ancestry proportion and modern human sample date [32].

In this study, we investigate the Neanderthal ancestry proportion in the newly reported and previously published hunter-gatherer populations, using f_4-statistics and f_4-ratio tests implemented in ADMIXTOOLS [33]. With f_4-statistics in the form of f_4(Chimp, Vindija Neanderthal; Zlatý kůň, Testpop), we measure the Neanderthal affinity in the tested European hunter-gatherers compared to the >45k-year-old Zlatý kůň individual, which carries a similar amount of Neanderthal ancestry as most ancient and present-day non-African individuals/groups [13]. We find that with the exception of Bacho Kiro IUP and Oase 1, which show stronger affinity to the Vindija Neanderthal, all the other hunter-gatherer individuals/groups are consistent with Zlatý kůň in their Neanderthal affinity (Data S2.M). This confirms the findings of previous publications reporting a constant level of Neanderthal ancestry in European modern humans during the past 40k years [32], while increased Neanderthal ancestry was found recently admixed in pre-40k-year-old individuals from the Balkans, Oase 1 and the Bacho Kiro IUP population [14,34].

We further estimate the Neanderthal ancestry proportion (α) in the European hunter-gatherer populations (with over 100,000 SNPs available on 1240K panel), using a "direct" f_4-ratio test following the strategy in Petr et al., 2019 [32]:

$$\alpha = \frac{f_4(Altai, Chimp; Test, Mbuti)}{f_4(Altai, Chimp; Vindija, Mbuti)}$$

We find that the estimation is highly variable for low-coverage genomes (Fig. S15), even accounting for the standard error estimated using jackknife. The two samples that were reported to carry additional Neanderthal ancestry, Oase 1 and Bacho Kiro IUP [14,34], still show significantly higher Neanderthal ancestry proportions than the other groups, with α estimated to be 7.6% and 3.5%, respectively (Data S2.N).

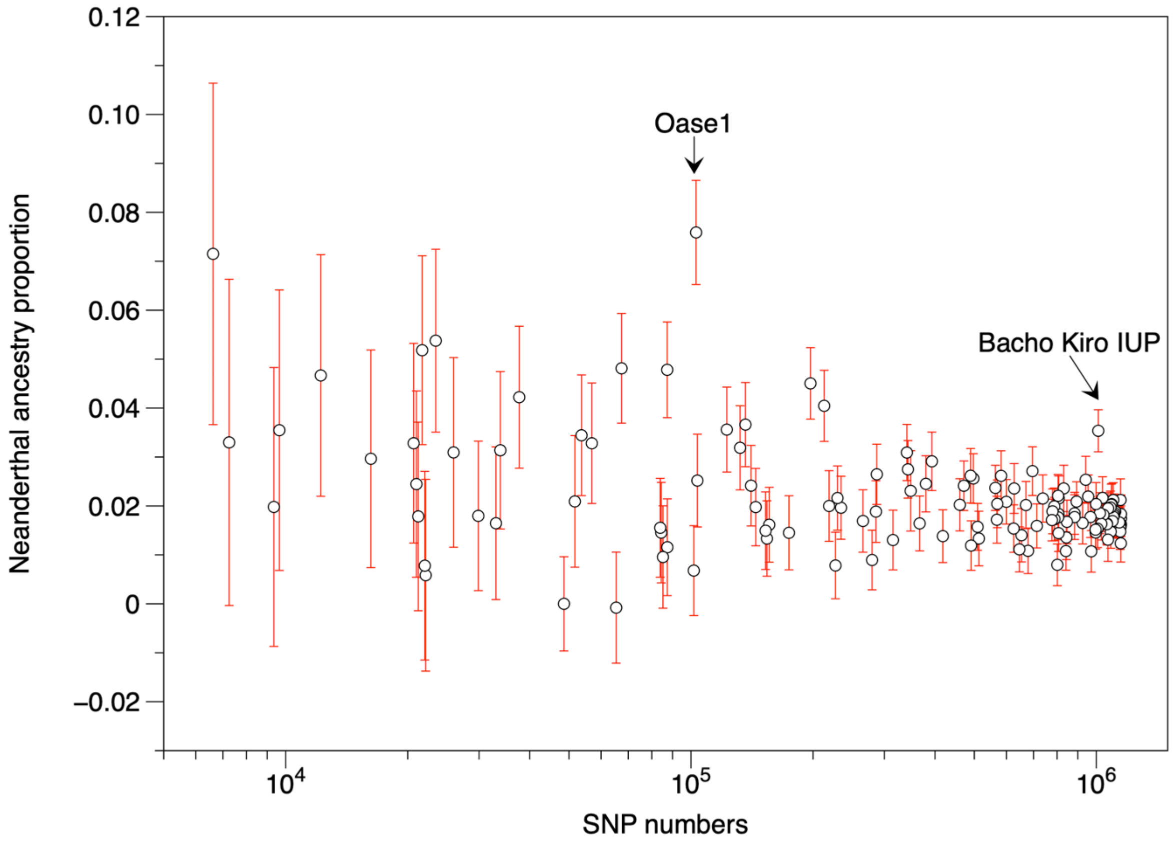

Figure S15. The Neanderthal ancestry estimation is highly affected by data quality. The x-axis shows the SNP number of tested individuals/groups and the y-axis (center of dots) shows the Neanderthal ancestry proportion (α). The dots and error bars show the point estimation and SD based on jackknife resampling on 5cM block SNPs. Oase 1 and Bacho Kiro IUP are pointed out as showing significantly higher Neanderthal ancestry compared to the other tested populations.

In order to investigate how this estimation is affected by data quantity, we downsampled three samples with relatively high SNP coverage, namely Kostenki 14, Fournol 85 and Abri des Autours 3 (AAT), and compared the f_4-ratio estimations to the results obtained with the original genotypes. For each individual, we downsample the original genotype to 50k, 100k, 200k, 300k, 500k and 1M SNPs by randomly introducing missing SNPs across the genome, and for 100 replicates at each coverage, using an in-house script. Since the SNPs available in the original genotype of Fournol are ~610k, we start the downsampling for this individual from 500k SNPs. We then estimate the Neanderthal ancestry proportion in each downsampled genotype and summarize the f_4-ratio estimations in Fig. S16. As expected, we directly show that the uncertainty in the estimation of the Neanderthal ancestry proportion increases by decreasing the SNP coverage. At around 50k SNPs, the value can deviate from the original estimation up to 3%. Moreover, until 200k SNPs the deviation of some downsampled samples fall outside the full uncertainty range obtained with the highest SNP number (1M or 500k). Therefore, we choose 300k SNPs as a cut-off for a robust estimation, when the variation is around 1% in Neanderthal ancestry, roughly twice as much as the standard deviation obtained with the jackknife resampling integrated in the f_4-ratio software.

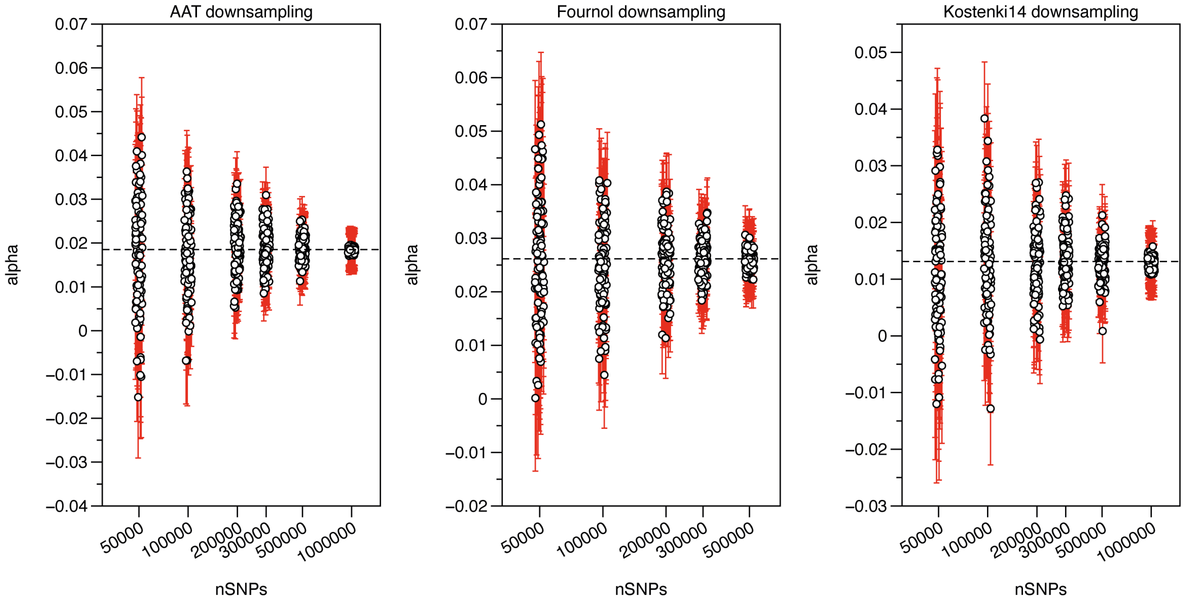

Figure S16. Relationship between variation in f_4-ratio estimation and sample coverage. The dashline shows the point estimation obtained with the original genotype, and the white dots with red error bars shows the point estimation of f_4-ratio with 1*SE estimated using 5cM block jackknife resampling, from downsampled genotypes.

When we only consider the populations with over 300k SNPs available, the estimation is around 1-3% for most European hunter-gatherers. After removing from the tested populations the ones with known additional Neanderthal ancestry (Oase 1 and Bacho Kiro IUP), or carrying significant farmer ancestry (Weyhe-Dreye, Ostorf, Hungary_Koros, Poland_Konary, Germany_Blatterhohle, Estonia_CCC, see Supplementary Information section 11 for the detection of farmer ancestry), we fit the correlation between sample age and Neanderthal ancestry using a linear function. We do not find significant correlation between the sample dates and Neanderthal ancestry proportion (coefficient of determination $R^2 = 0.028621$), which confirms the conclusion of [32] that the Neanderthal ancestry in modern human genomes did not decrease significantly in the past 45,000 years (Fig. S17).

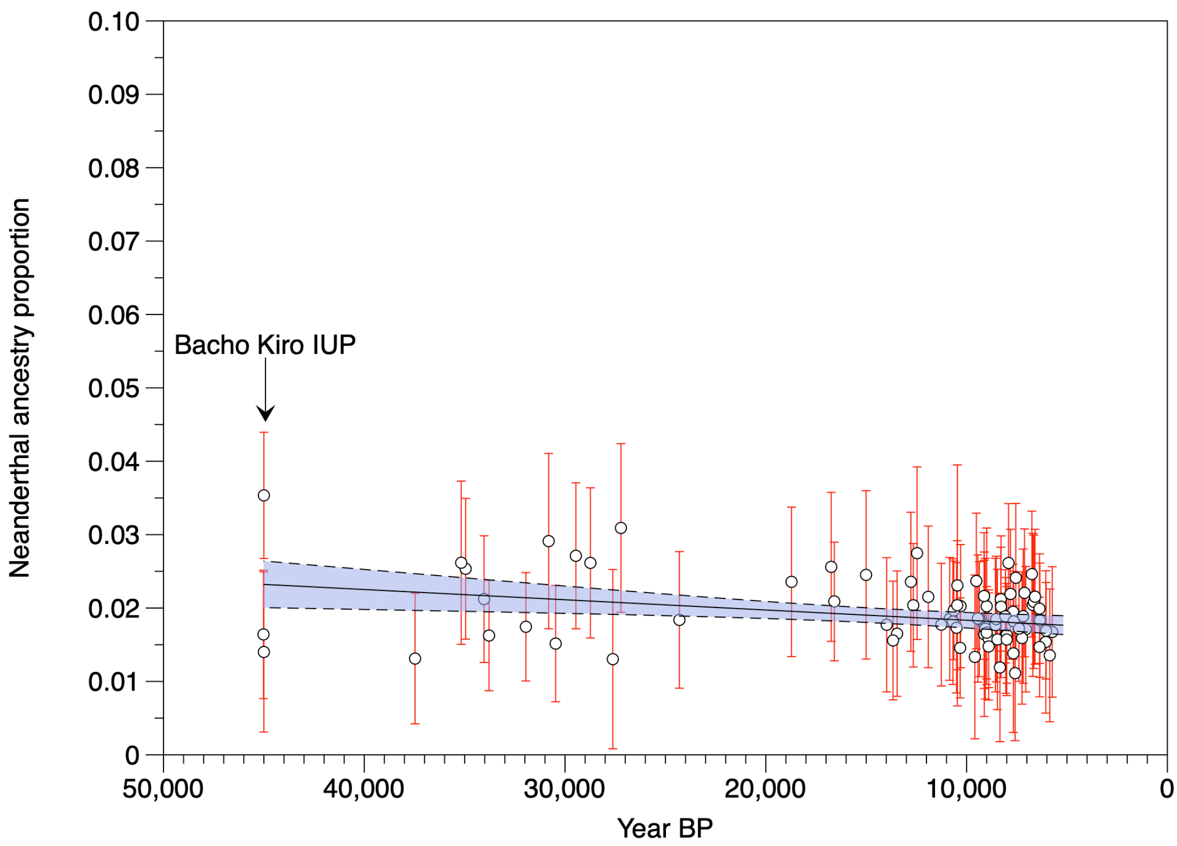

Figure S17. The Neanderthal ancestry proportion in European hunter-gatherers.
The x-axis shows the age of tested individuals/populations (> 300k SNPs covered) in years before present (BP) and the y-axis shows the Neanderthal ancestry proportion (α). The dots and error bars show the point estimation and 2*SD based on jackknife resampling on 5cM block SNPs, which corresponds to 95% CI of the estimation. The horizontal line shows the X-Y linear fitting and the shade with dashed outline shows the SD of the fitting function.

Section 7: East Asian affinity in Goyet Q116-1-related populations

A previous study reported an extra affinity between the 35k-year-old Goyet Q116-1 individual and a 40k-year-old East Asian individual Tianyuan, which was not present in any other West Eurasian individuals sequenced until then [35]. Such affinity was later also detected in the Bacho Kiro IUP population (46-43 kya), which was modelled as contributing to both Goyet Q116-1 and Tianyuan, thus drawing genetically together the two latter individuals [14].

In this study, we describe the *Fournol* ancestry which was present in Gravettian- and Solutrean-associated individuals from western and southwestern Europe, as a sister lineage of the *GoyetQ116-1* ancestry (Fig. 2, Data S2.B). This raises the question of whether the affinity to Tianyuan could also be detected in the *Fournol* cluster individuals.

We therefore explore the Tianyuan-related affinity in newly reported and published European hunter-gatherers, with f_4-statistics in the form of f_4(Mbuti, Tianyuan; Kostenki 14, Testpop) (Data S2.P). We find that the Fournol 85 individual also shows significantly higher affinity to Tianyuan compared to Kostenki 14 (Z=3.596), as observed for Goyet Q116-1. The statistics is not significant in other *Fournol* cluster populations, like the Serinyà group (Z=2.037), potentially due to the relatively low SNP coverage of these individuals. The Tianyuan-related affinity in Fournol and Goyet Q116-1 is also on a similar level, as revealed by f_4(Mbuti, Tianyuan; Goyet Q116-1, Fournol) ~ 0 (Z=-0.074). However, as the genetic affinity between Tianyuan and Goyet Q116-1 has been described as a shared genetic legacy derived from the Bacho Kiro IUP population [14], we also test the affinity to Bacho Kiro IUP in the European hunter-gatherers using f_4(Mbuti, Bacho Kiro IUP; Kostenki 14, Testpop). As expected, we find significantly higher affinity to Bacho Kiro IUP in Goyet Q116-1, as well as several *Fournol* cluster populations, including Fournol 85, the Serinyà group and Ormesson 2988 (Data S2.P). Overall, our finding confirms that the *Fournol* ancestry, which largely survives through the LGM in southwestern and western Europe, shares the genetic affinity to Bacho Kiro IUP and Tianyuan that was observed in the Aurignacian-associated Goyet Q116-1 individual. This is consistent with the admixture graph modelling among these ancestries (Fig. S21), where Fournol 85 is a sister lineage of Goyet Q116-1 and shares a genetic contribution from a common Bacho Kiro IUP-related lineage, whereas Tianyuan carries a genetic contribution from another Bacho Kiro IUP-related lineage.

This suggests that the Bacho Kiro IUP-related ancestry indeed left genetic traces in some post-40ka populations from Europe as it did in Asia, but it does not constitute the main ancestry component found in those groups being modelled as a basal lineage that diverged before European and Asian groups split from each other (Fig. S21).

Section 8: Near Eastern affinity in European hunter-gatherers

An affinity between the *Villabruna* ancestry and ancient and modern-day Near Eastern populations has been described in previous studies, suggesting a potential bi-directional genetic influence between the Near East and Europe as early as 15,000 years ago [36,37]. In this study, we also test for the presence of the Near Eastern affinity in the newly reported European hunter-gatherers, with a special focus on Epigravettian-associated individuals from Italy that belong to the *Villabruna* cluster.
With f_4-statistics in the form of f_4(Mbuti, Natufian/Pinarbasi/Jew_Iraqi; Kostenki 14, Test), we confirm that the Near Eastern-related affinity in European hunter-gatherers started to increase after the LGM corresponding to the expansion of the *Villabruna* and *Oberkassel* ancestries, first in southern and southwestern Europe and later in the rest of Europe (Fig. S18, Data S2.O). The ~19k-year-old El Mirón individual and the ~17k-year-old Tagliente 2 individual both carry the Near Eastern affinity. We also detect the Near Eastern-related affinity in an 18k-year-old individual from La Marche and the 15k-year-old Goyet Q-2, which could be explained by our previous observation that the *GoyetQ2* cluster also carries *Villabruna* ancestry, though in a lesser proportion than in El Mirón. Pairwise comparisons between different Epigravettian-associated individuals (f_4(Mbuti, Natufian/Pinarbasi/Jew_Iraqi; Epigravettian1, Epigravettian2)) also show that all these individuals share an affinity with ancient and modern-day Near Easterners in roughly equal intensity (Data S2.F). This confirms that the Near Eastern affinity carried by the *Villabruna* ancestry was already present before its spread into southern Europe.
Interestingly, we also detect minor Near Eastern affinities in pre-LGM populations associated with the *Věstonice* cluster, like the Věstonice group and Paglicci 12 (Fig. S18, Data S2.O). This could be explained by a portion of shared genetic ancestry between the *Villabruna* and *Věstonice* ancestries as described in Fu et al., 2016 [36]. Moreover, considering that both Gravettian and Epigravettian cultures were also found in the Balkans, and that the 35k-year-old Bacho Kiro 1653 individual from Bulgaria has been proposed to contribute genetically to the Věstonice population [14], we speculate that this signal is most likely related to a deep genetic link between the Near East and the Balkans. We specifically test this hypothesis using *qpGraph* modelling as described in Supplementary section 10. Notably, this link has also been suggested in previous studies, based on the Near Eastern affinity found in Mesolithic hunter-gatherers from the Iron Gates in the Balkans [28,37].

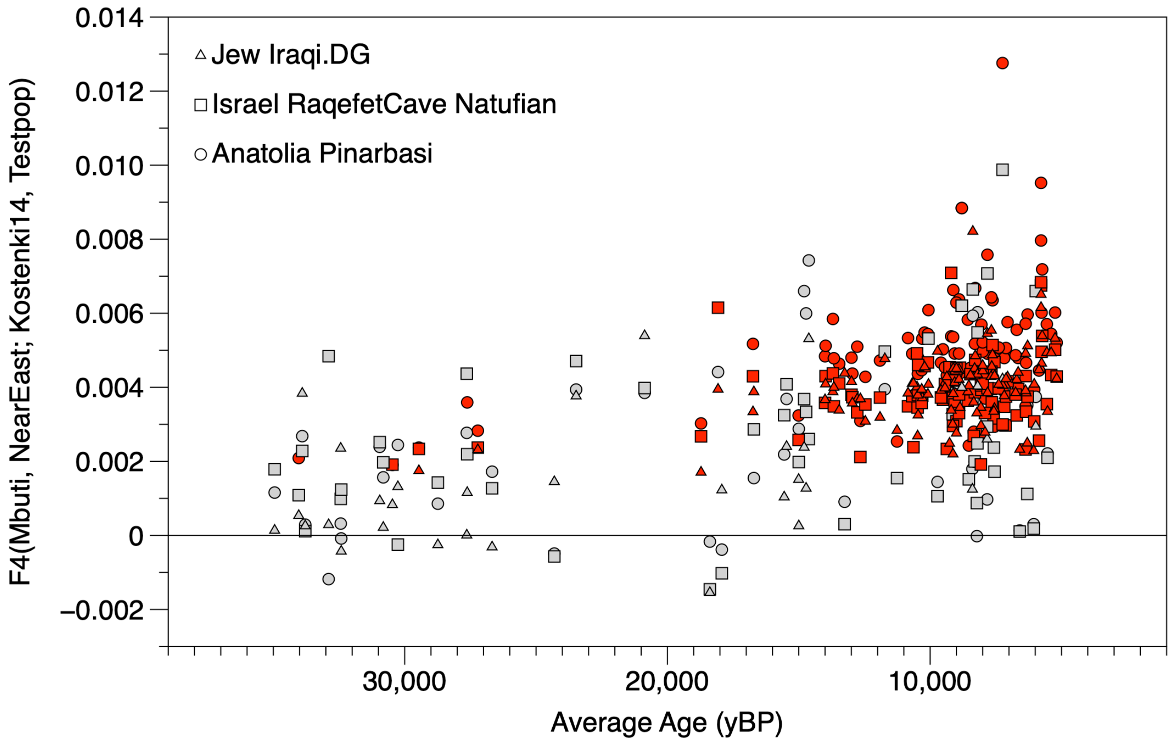

Figure S18. The Near Eastern affinity in European hunter-gatherers after 35,000 BP.
This figure shows the affinity to different Near Eastern populations in European hunter-gatherers. The x-axis shows the average age of each tested population, and the y-axis shows the f_4 values of each test. The shape of the dots shows the Near Eastern population used to test the affinity, and the color indicates if the result is significantly deviated from 0. Red dots are tests with $|Z|>3$ and grey dots are tests with $|Z|<3$.

Section 9: Run of homozygosity (ROH) in European hunter-gatherers

We detect run of homozygosity (ROH) segments in all newly reported and published hunter-gatherer individuals with over 400k SNPs covered on the 1240K panel, following the suggested cutoff in HapROH [4] (Extended Data Fig. 5).

After filtering for individual SNP coverage, we run HapROH on 21 pre-LGM individuals, 10 Epigravettian-associated, two Magdalenian-associated and 148 Mesolithic to Neolithic individuals. We also include two Epigravettian-associated individuals (Pradis 1 and Oriente C) and one Magdalenian-associated individual (Goyet Q-2) with 200-400k SNPs in the analysis to get a more complete picture of ROH distribution in individuals associated with these cultures, which are under-represented in the dataset.

We generally find that there is a limited number of hunter-gatherer individuals carrying recent inbreeding signals, as reflected by a limited amount of long ROH segments (>20 cM). The pre-40k individuals (Zlatý kůň, Ust'Ishim, Bacho Kiro IUP) show similar amounts of short ROHs, which reflect their effective population sizes (that can greatly differ from the census size) consistent to be around 400 individuals. The Zlatý kůň individual also shows a recent inbreeding signal. In contrast, we observe a large variation of cumulative ROH amounts among individuals dated to 40-32k BP. Goyet Q116-1 carries extremely large amounts of ROH, compared to individuals from the Balkans (Bacho Kiro 1653, Muierii 1) and Russia (Kostenki 14), suggesting a strong bottleneck during the spread of the Goyet Q116-1-related population into central Europe. The Sunghir individuals also show increased ROHs compared to Kostenki 14, but not as substantial as in Goyet Q116-1. It has been argued that this is obtained through a social organization with limited intra-group relatedness in a larger mating network [38]. The Gravettian-associated individuals from different regions also carry high amounts of ROHs, intermediate between Goyet Q116-1 and the Sunghir individuals. Interestingly, we do not observe strong bottleneck signals in the ~32k-year-old Yana individuals from northeastern Siberia, suggesting that these Siberian hunter-gatherers might have maintained a similar population size as Gravettian-associated individuals from Europe.

The amounts of cumulative ROH segments in Epigravettian-associated individuals are highly correlated with their geographic distribution. The individuals from northeastern Italy (Group1, as described in Fig. 3) and southwestern coast (Group2) show similar amounts of ROH segments, while the Sicilian individuals (Group3) carry the largest amount of ROHs with the total ROH lengths around 400 cM. This suggests an extremely small population size and a strong bottleneck during the Epigravettian spreading into Sicily, correlating well with the population diversity decline and phylogenetic relationship among Epigravettian groups (Fig. 3).

We also estimate their effective population sizes (N_e) based on maximum likelihood fitting, using individuals without recent inbreeding (cumulative length of long ROHs (>20cM) less than 50cM), and their ROH segments between 4 cM and 20 cM. The Ne of Epigravettian Group1 and Group2 are both estimated to ~200-250 individuals, while Group3 shows a significant decrease to only ~70 individuals (Table S2). The estimation confirms a strong bottleneck in Sicilian Epigravettians (and Early Mesolithic), and suggests that the hunter-gatherer population in Sicily during the Upper Paleolithic was restricted to a very small size, potentially due to the settlement process of this Mediterranean island and/or to the local environment. It is also interesting that, despite the small population size, these hunter-gatherer populations managed to avoid recent inbreeding as indicated by the relatively low number of large ROHs.

The two Magdalenian-associated individuals carry similar amounts of short ROHs with roughly contemporaneous Epigravettian-associated individuals from Group1 and Group2, with the 19k-year-old El Mirón individual from present-day Spain also showing an excess of long ROHs indicating

recent inbreeding. Therefore, the N_e of Magdalenian-associated populations is currently based on the Goyet Q-2 individual and estimated to be ~250 individuals, slightly higher than the Epigravettians from Group1 and Group2. However, since the current N_e value is based on a single individual, we are not able to confidently compare population sizes between Magdalenian- and Epigravettian-associated populations.

Table S2. Effective population sizes estimated based on ROH segments in Epigravettian and Magdalenian populations.

Population	Coeff (2N)	0.025	0.975	Individual N	Ne
Epigravettian_all	260	231	294	8	130
Group1	436	326	599	2	218
Group2	480	375	629	3	240
Group3	143	121	170	3	72
Magdalenian	511	331	830	1	256

Mesolithic as well as Neolithic individuals generally carry a lower amount of ROHs compared to Upper Paleolithic individuals, suggesting an increase in hunter-gatherer population size during these later periods. We also notice that individuals from the Baltics (Baltic Mesolithic, Nava and Middle Neolithic), British Isles (British Mesolithic and Ireland Mesolithic) and Doggerland show relatively higher amounts of ROHs compared to contemporaneous individuals from the other regions. This indicates bottleneck events during the post-LGM expansions of hunter-gatherer populations into northern European areas. Overall, recent consanguinity is rarely found in the hunter-gatherer individuals studied here, even in groups with extremely small population sizes, like Epigravettian-associated populations from the Italian peninsula.

Section 10: Reconstructing the admixture graph of major pre-LGM genetic ancestries

We apply qpGraph to reconstruct the admixture graph of major pre-LGM ancestries described in this study, aiming to resolve 1) the relationship between the Bacho Kiro IUP individuals and Zlatý kůň, the oldest modern human individuals sequenced from Europe to date [13,14], and 2) the phylogenetic position of different Gravettian-associated lineages.

Starting from a scaffold tree (Chimp, (Vindija Neanderthal, Mbuti)), we first iteratively add Zlatý kůň, Sunghir (n=4) and Bacho Kiro IUP (n=3). For each new lineage, we test all the possible graphs with the new lineage fitted as either a sister lineage, or a two-way admixture of existing lineages. The best fitted model is selected based on the worst Z-score among all f_4 combinations and the final likelihood scores.

As the Bacho Kiro IUP individuals have been reported to carry a higher Neanderthal ancestry than most other European hunter-gatherers [14], we add Zlatý kůň and Sunghir first, and then fit Bacho Kiro IUP on the graph. Zlatý kůň and Sunghir can be fitted as resulting from a single gene flow from the Vindija Neanderthal genome, while Bacho Kiro IUP is best fitted as the sister lineage of Sunghir, with an additional gene flow from Vindija (Fig. S19a). This further confirms the basal positioning of the Zlatý kůň genome among all known modern human lineages out of Africa [13].

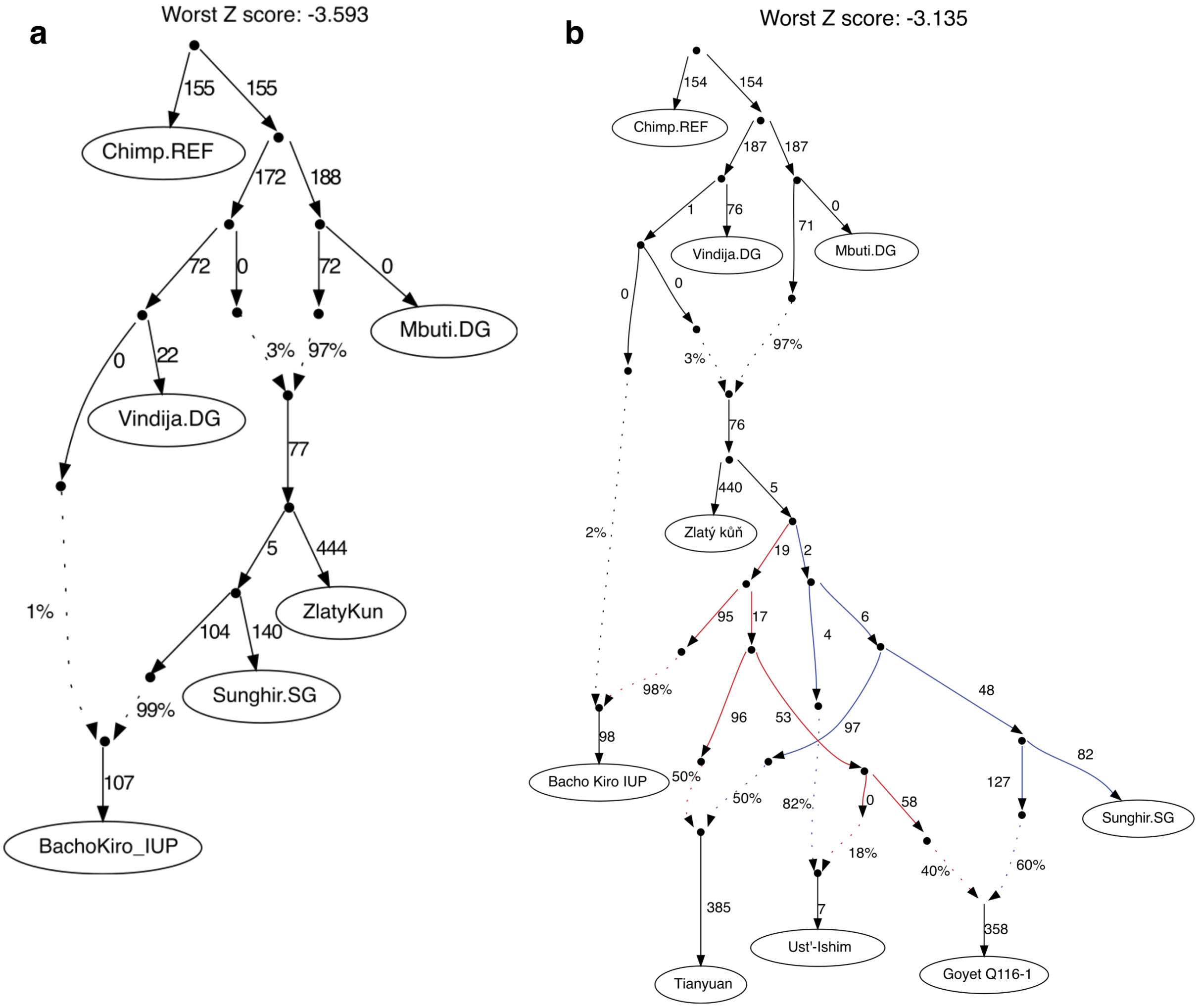

Figure S19. The admixture graph modelling of early modern human lineages out of Africa. (a) the Zlatý kůň is fitted as the basal lineage of modern human out of Africa and (**b**) the Bacho Kiro IUP group contributes to Tianyuan, Ust'Ishim and Goyet Q116-1 (indicated with red lines), but not to the Sunghir group (graph reported also as Extended Data Fig. 4).

We further add the Ust'Ishim, Tianyuan and Goyet Q116-1 genomes on the graph, and confirm that all three lineages carried dual ancestries related to both the Sunghir and Bacho Kiro IUP populations (Fig. S19b). This admixture graph is slightly different from the published graph [14], since the Bacho Kiro IUP-related lineages contributing to these three populations branch off after the additional Neanderthal gene flow into Bacho Kiro IUP. Considering that the Neanderthal introgression in Bacho Kiro IUP was estimated to be a recent event [14], and that Ust'Ishim, Tianyuan and Goyet Q116-1 all carry similar Neanderthal ancestry proportions as most other non-African populations, we think the scenario suggested in our model is more likely, i.e., that the genetic contributions are from Bacho Kiro IUP-related populations that did not carry the extra Neanderthal ancestry found in Bacho Kiro IUP (Fig. S19b).

In the next step, we add different Gravettian-associated lineages on this 9-population graph. We choose Fournol 85 to represent the *Fournol* ancestry and the Věstonice group to represent the *Věstonice* ancestry (Fig. 2B, Data S2.B). Fournol 85 is fitted as a sister lineage of Goyet Q116-1, then Věstonice is fitted as the mixture between the Goyet Q116-1/Fournol 85 lineage and the Sunghir-related lineage (Fig. S20). This supports the genetic affinity between the *Fournol* ancestry and Goyet Q116-1, and between the *Věstonice* ancestry and Sunghir, as revealed by f_4-statistics (Fig. 2B, Extended Data Fig. 3). We further add Paglicci 12 on this graph to represent Gravettian-associated individuals from Italy, which is best fitted as a sister lineage of the Věstonice group, confirming that a similar *Věstonice* ancestry was distributed in central-eastern and southern Europe in Gravettian-associated populations (Fig. S21).

However, this graph still does not fully represent the distinct genetic affinity between the Věstonice and Sunghir lineages in comparison to Goyet Q116-1, as revealed by a series of f_4-statistics and previously shown [14] (Data S2.B). To resolve the trifurcation among the lineages contributing to Goyet Q116-1, Věstonice and Sunghir we simplify the graph by removing both non-European early modern humans (Ust'Ishim and Tianyuan) and include Kostenki 14. Since we consistently detect extra affinity between Kostenki 14 and Mbuti - suggesting unresolved interactions among their associated deep lineages - we replace Chimp with the high-coverage Denisova 3 individual. Following the graph published in Hajdinjak *et al.* [14], we model Denisova 3 as the mixture between a super-archaic and a Neanderthal-related lineages (these admixture proportions are not to be considered). In the resulting model, the previously observed trifurcation is resolved with Věstonice deriving from the mixture between a lineage closely related to Sunghir, and another lineage that splits basally to the Goyet Q116-1/Fournol 85 clade (Fig. 2c).

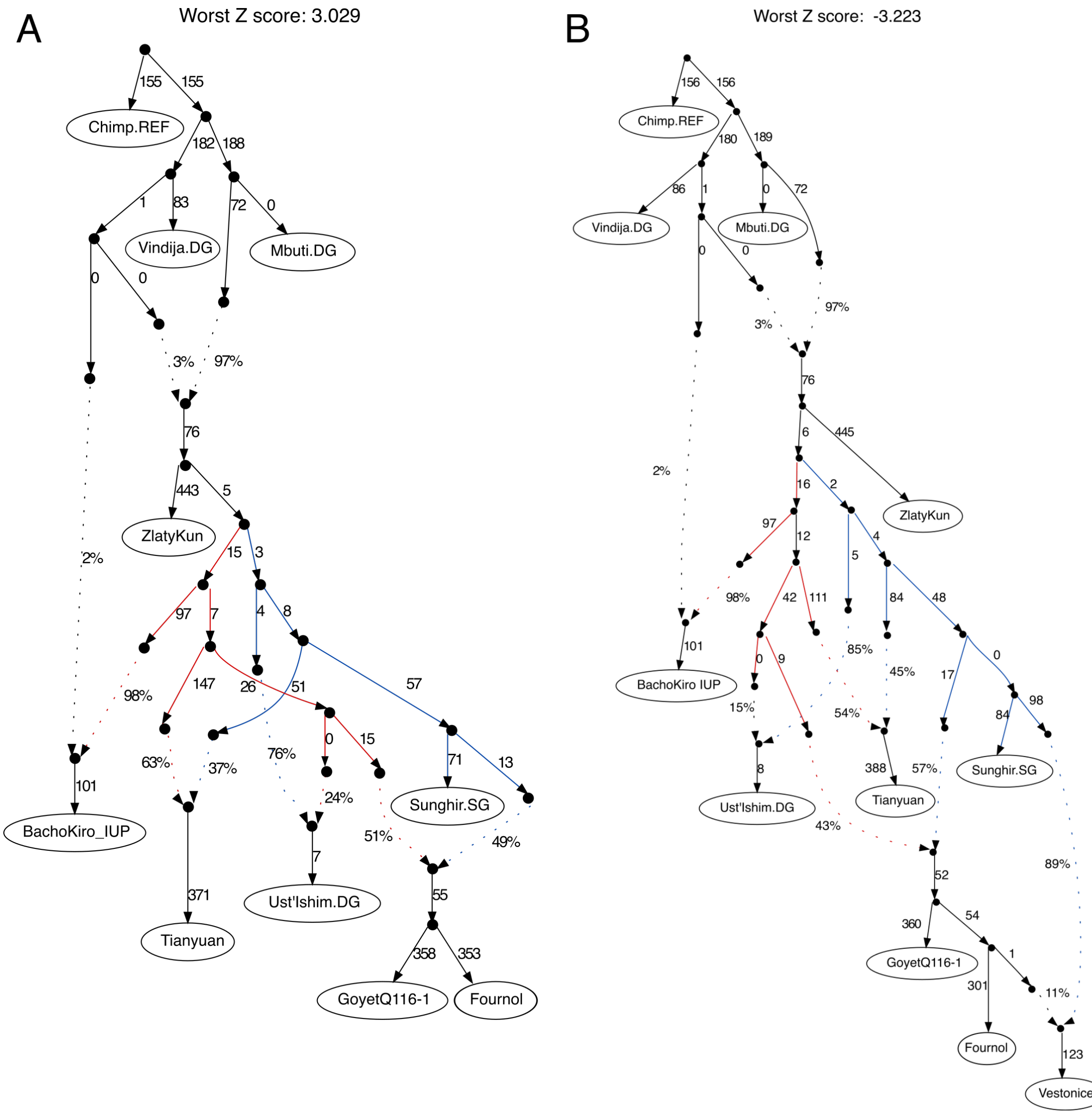

Figure S20. Admixture graph modelling of *Věstonice* and *Fournol* ancestries. (A) The Fournol 85 lineage is bested fitted as a sister lineage of Goyet Q116-1. **(B)** The Věstonice lineage is best fitted as a two-way admixture between Fournol-related and Sunghir-related lineages. The red branches indicate genetic contribution from Bacho Kiro IUP-related lineages and the blue branches indicate genetic contribution from a Sunghir-related lineage.

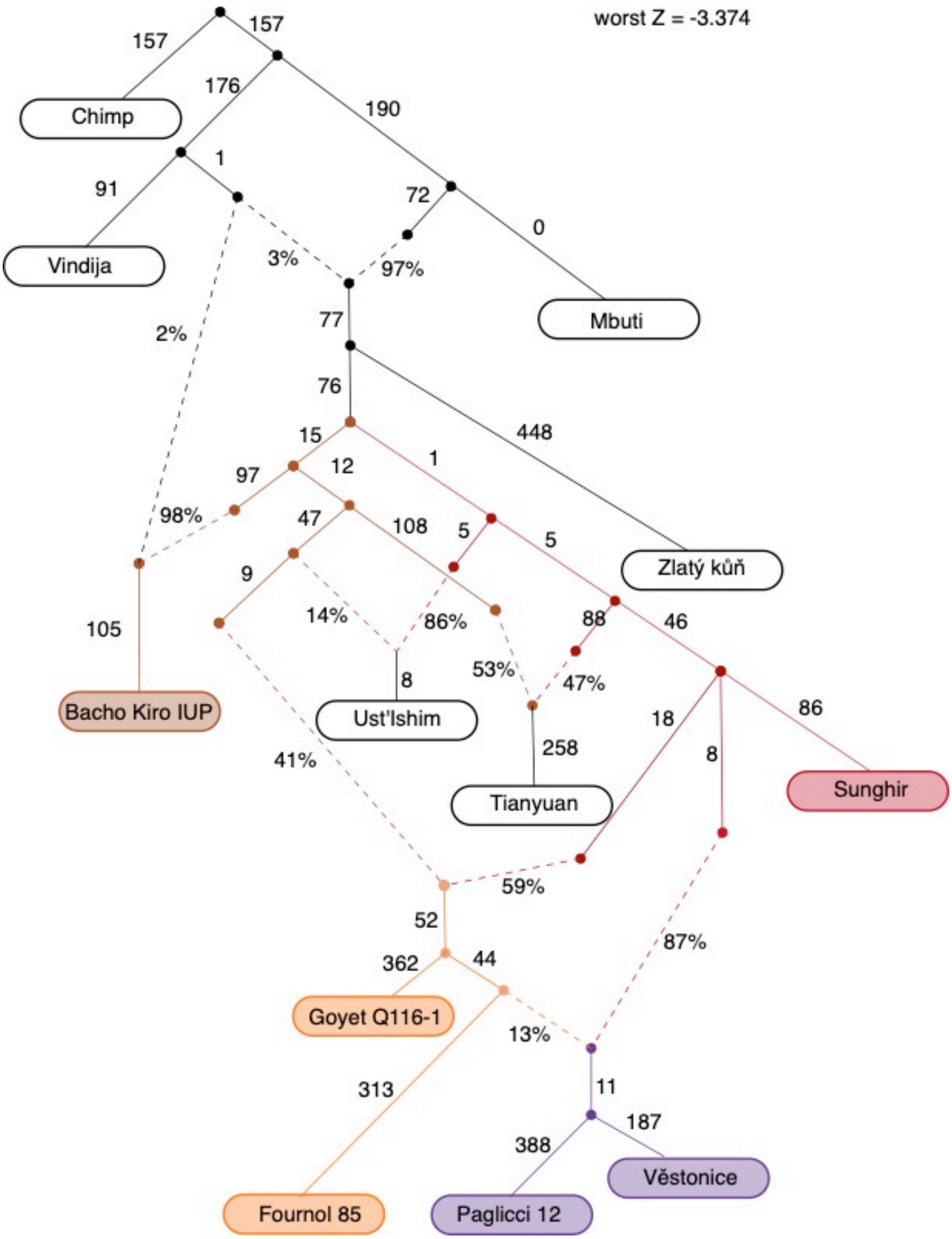

Figure S21. Admixture graph modelling of pre-LGM Eurasian hunter-gatherer lineages. In this admixture graph, the lineage related to Zlatý kůň splits more basally than the Bacho Kiro IUP group, which contributes to Tianyuan, Ust'Ishim and Goyet Q116-1 (indicated with brown lines), but not to the Sunghir group. Among the Gravettian-associated individuals, Fournol 85 is modeled as a sister clade of Goyet Q116-1 whereas Paglicci 12 and the Věstonice group derive most of their ancestry from a distinct lineage.

Investigating the Bacho Kiro 1653 genetic ancestry with qpgraph

The ~35k-year-old Bacho Kiro 1653 individual (BK-1653) from present-day Bulgaria, published in [14], was suggested to be a sister lineage of its contemporaneous Goyet Q116-1 individual from central-western Europe. Interestingly, this individual showed a genetic connection with Věstonice 16, indicating a potential contribution from a population related to Bacho Kiro 1653 in the formation of the *Věstonice* ancestry. We validate the relationship between BK-1653 and the *Věstonice* ancestry with f_4-statistics in the form of f_4(Mbuti, Testpop; Goyet Q116-1, BK-1653), which is significantly positive for *Věstonice* cluster populations like the Věstonice group and Paglicci 12, and significantly negative for *Fournol* cluster populations like Fournol 85 and the Serinyà group (Data S2.B).

We further try to fit BK-1653 on the admixture graph to better understand its relationship with different Gravettian-associated ancestries. On the graph shown in Fig. S22A we find the best fitted model of BK-1653 deriving from a two-way admixture between a lineage closely related with Goyet Q116-1, although without the Bacho Kiro IUP-related contribution, and an ancestral lineage diverging more basally than all other European hunter-gatherer lineages, including the Zlatý kůň one. This is partially in line with the published graph which also revealed a contribution to BK-1653 from a deep lineage that is not related to Bacho Kiro IUP [14] .

We then fit Věstonice on the graph including BK-1653, and find it is best modelled as a two-way admixture of the BK-1653 and Sunghir lineages, as reported in Hajdinjak et al., 2021 [14] (Fig. S22B). This supports the observations from f_4-statistics that Věstonice is more closely related to BK-1653 than Goyet Q116-1, further suggesting different genetic origins of the *Věstonice* and *Fournol* ancestries. However, it is worth noting that the best fitted model with BK-1653 shows the worst Z-score of 3.659, over the 3.5-threshold previously used to assess the robustness of complex qpGraph models e.g., [39,40]. The f_4-statistics outlier in this admixture graph shows an un-modelled genetic affinity between the present-day African Mbuti populations and BK-1653, suggesting that BK-1653 may carry a more complex genetic origin that cannot be fully resolved with the lineages know so far. Therefore, further investigations into pre-LGM Balkan hunter-gatherers would be crucial to better understand the formation of this unique genetic ancestry, as well as of the derived *Věstonice* ancestry.

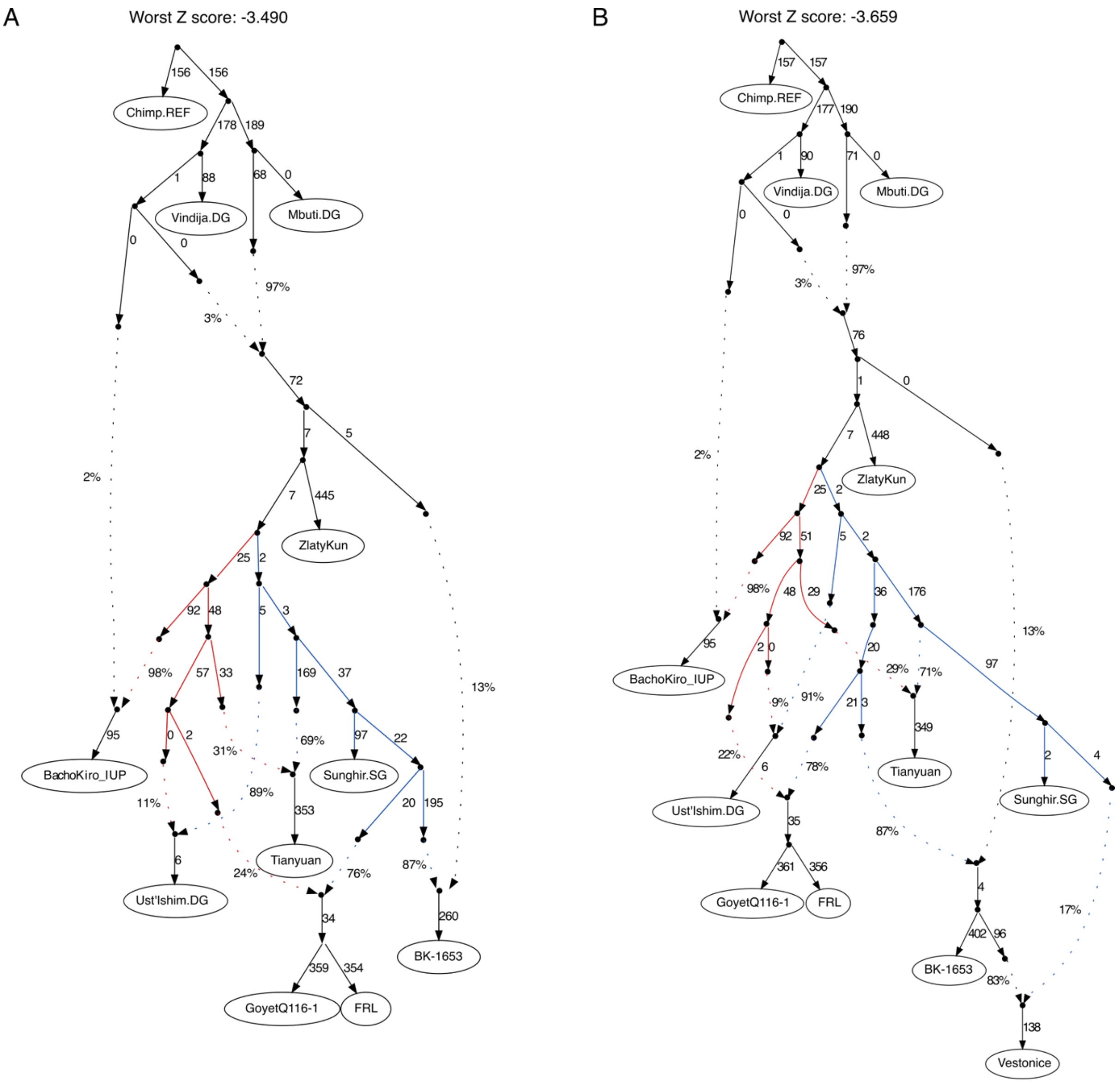

Figure S22. The admixture graph modelling of 35k-year-old BK-1653.
(A) BK-1653 is best fitted as a two-way admixture between a Sunghir-related lineage and a ghost lineage ancestral to all known European hunter-gatherer lineages including Zlatý kůň. **(B)** Věstonice is further modelled as a two-way admixture between Sunghir- and BK-1653-related lineages, instead of from a Fournol-related lineage as reported in Fig. 2B. FRL = Fournol.

Genetic affinity between *Věstonice* and *Villabruna* ancestries
A genetic contribution from the *Villabruna* ancestry into the *Věstonice* ancestry has been previously proposed [36], while in a later study the *Věstonice* ancestry was modelled as an admixture between BK-1653 and Sunghir [14] and confirmed here in Fig. S22. However, none of the previous studies have modelled BK-1653, Věstonice and Villabruna together. We therefore continue to add Villabruna onto our working model and try to disentangle the relationships between these three types of ancestries. As described above, BK-1653 may carry a more complex genetic background associated with deeply divergent lineages. To reduce the impact of this signal and focus more on younger lineages, we start from another skeleton graph without the Chimp- and Neanderthal-related lineages. From (Mbuti,(Zlatý kůň, Kostenki 14)), we iteratively add Ust'Ishim, Tianyuan, Goyet Q116-1, Sunghir, and BK-1653. The best fitted models reproduce the relationships among these lineages shown in the previous qpGraph models (Fig. S23).

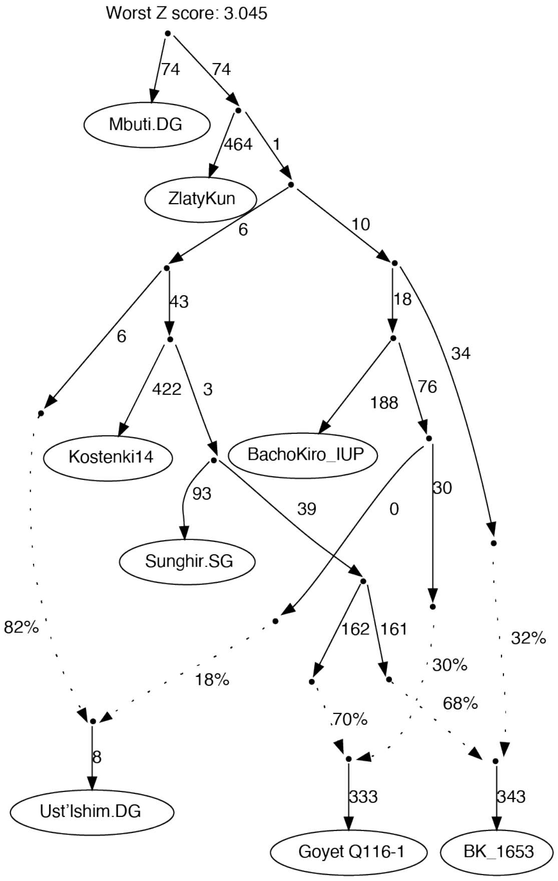

Figure S23. The admixture graph modelling of BK-1653 with Mbuti as the outgroup.

We further add the two Gravettian-associated lineages, Věstonice and Fournol, on the graph. As previous models revealed, Fournol 85 fits as the sister lineage of Goyet Q116-1, and the Věstonice group as the admixture between BK-1653 and Sunghir (Fig. S24).

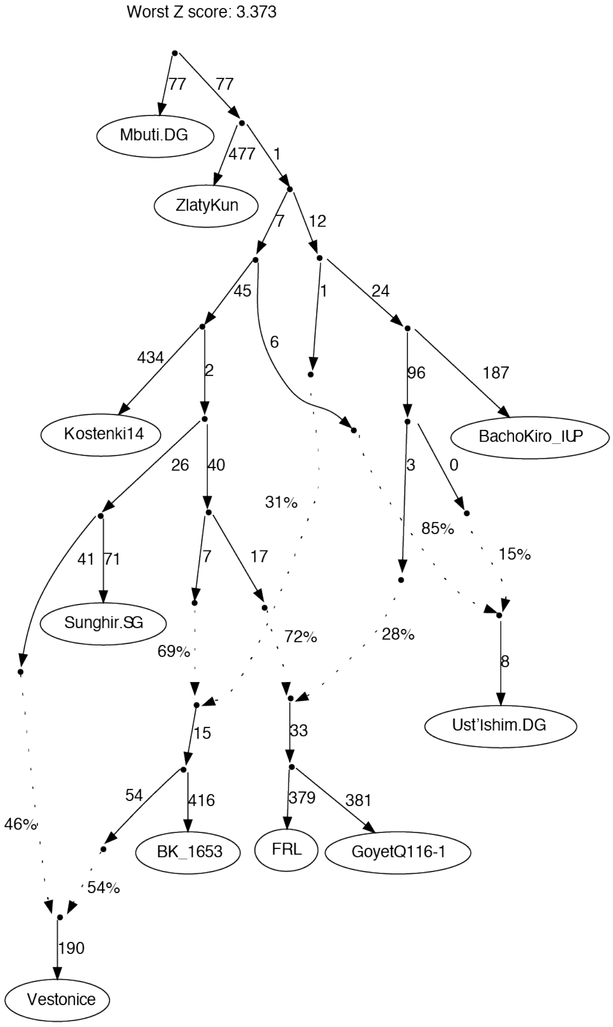

Figure S24. Modelling of the Věstonice and Fournol lineages on the graph with Mbuti as the outgroup. FRL = Fournol 85.

We then fit Villabruna on this graph. Interestingly, we find that the best fitted model suggests Villabruna to result from the admixture between a Věstonice-related lineage and a branch that is basal to the split between Kostenki 14 and Goyet Q116-1 genomes (Fig. S25). The shared part of ancestry between Věstonice and Villabruna is related to BK-1653 and could potentially explain the affinity between these two ancestries observed in Fu et al., 2016 with qpGraph modelling [36]. This suggests that the previously identified shared genetic ancestry in the *Věstonice* and *Villabruna* clusters might trace back to pre-30k populations from the Balkans.

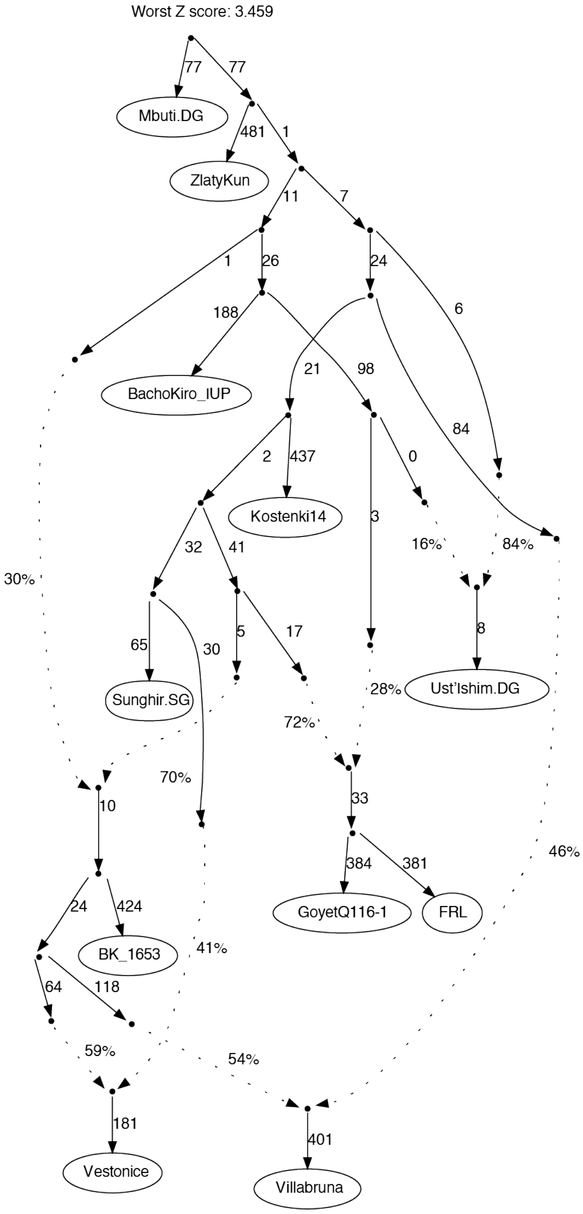

Figure S25. Graphic modelling of Věstonice and Villabruna pointing to a shared Balkan-related ancestry. FRL = Fournol 85.

Section 11: Genetic ancestry modelling of post-LGM hunter-gatherers

Genetic ancestry of Magdalenian-associated populations

Previous studies have reported two major types of genetic ancestries in Magdalenian-associated hunter-gatherers, represented by the 15k-year-old Goyet Q-2 individual from Belgium and 19k-year-old El Mirón individual from Spain, with the latter carrying a significant amount of *Villabruna* ancestry [36,41]. We apply qpAdm, which is based on *f*-statistics, to model the genetic ancestry of Magdalenian-associated populations, as well as post-14ka hunter-gatherers from Iberia and a subset of *Oberkassel* cluster individuals from (central-)western Europe. We use the Fournol 85 individual to represent the *Fournol* ancestry, and Arene Candide 16 to represent the *Villabruna* ancestry.

We compile an outgroup set of 10 populations (OG10), including all major genetic ancestries that could help to differentiate European hunter-gatherers' ancestries: Mbuti.DG (N=4), Mota.SG (4,500 BP Mota individual from Ethiopia), Zlatý kůň, Ust'Ishim.DG, Tianyuan, Kostenki 14, Goyet Q116-1, Věstonice (N=5), Israel Raqefet Cave Natufian (Epipaleolithic hunter-gatherers from the Levant, N=6), and Villabruna.

We find that most of the tested populations can be modelled as two-way admixtures of Fournol 85 and Arene Candide 16, with a p-value over 0.05 (Data S3.C). All the Magdalenian-associated populations except for Rigney 1 show a good fit for the two-way model, with the contribution from Arene Candide 16 estimated to 43.1% in El Mirón from Spain, and 18.7-29.3% in the other Magdalenian-associated individuals dated between 18k and 14.6 ka. The Arene Candide 16 ancestry is dominant in post-14 ka populations from western and central Europe, consistent with ~88% (81.7-90.3%) (Fig. 4). As described in a previous study [41], in post-14 ka Iberian populations, the *Fournol* ancestry is preserved at higher levels, ranging between 20.7-50.7%. This is also validated by f_4-statistics of the form f_4(Mbuti, Arene Candide 16; Goyet Q-2, Magdalenian-associated individuals), which is significantly positive only for El Mirón while all other tested individuals are consistent to be equally related to Goyet Q-2 with respect to Arene Candide 16 (Fig. S26, Data S2.H).

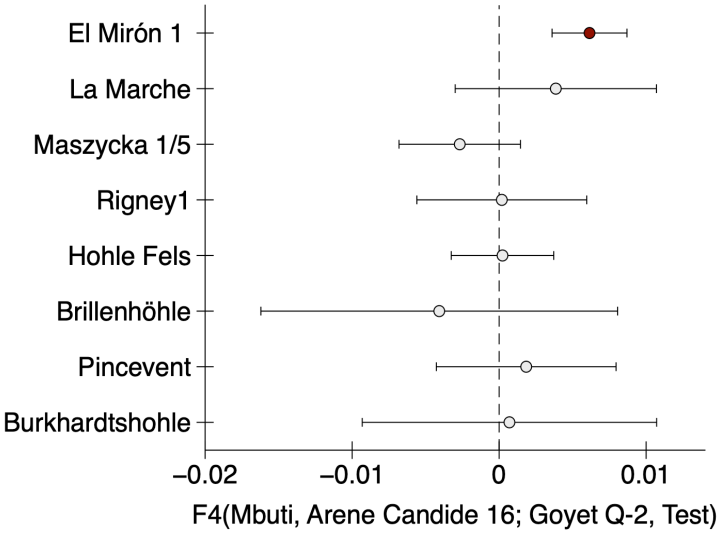

Figure S26. Relative affinity to *Villabruna* ancestry in Magdalenian-associated individuals. The Arene Candide 16 genome represents the *Villabruna* ancestry, while Goyet Q-2 and Test are Magdalenian-associated individuals. The X-axis reports the f_4-values as center of dots and the error bar shows 3*SD estimated using 5cM block jackknife resampling. The only test with significant deviation from zero is plotted in red.

Admixture between Mesolithic-Neolithic hunter-gatherers

To obtain a comprehensive overview on the genetic ancestry dynamics of European hunter-gatherers from the Mesolithic to the Neolithic, we apply qpAdm modelling for 75 hunter-gatherer populations across Europe, after filtering for a coverage of at least 50,000 SNPs (Data S3.E). We choose the Oberkassel group (N=2) to represent the *Oberkassel* ancestry, Yuzhny Oleni Ostrov population (N=19) to represent the *Sidelkino* ancestry, and Anatolian Neolithic farmers (N=30) [20,32] to represent farmer-related ancestry. As the populations from the Iberian peninsula have been suggested to carry extra Magdalenian-related ancestry compared to *Oberkassel*-related populations, we model the eight Iberian hunter-gatherer groups with additional *GoyetQ2* ancestry modeled by the Goyet Q-2 individual. We use an OG11 outgroup set, which is OG10 described above with the addition of MA1.SG representing the ANE ancestry, for all modelling analyses.

Firstly, we model the 66 Mesolithic to Neolithic populations except the ones from Iberia as three-way admixtures of Oberkassel + Yuzhny Oleni Ostrov + Neolithic farmers from present-day Turkey, and checked the p-value for nested models to favor the simplest fitting models. The simplest model (model requiring the least number of source populations) with both a tail probability and p-value for nested model over 0.01 was considered as the best fitting model for a population. Based on these criteria, we found six populations consistent with one-way *Sidelkino* ancestry, and 20 populations consistent with one-way *Oberkassel* ancestry. This was also confirmed by qpWave modelling of those populations being consistent with deriving from the same ancestral wave of either Oberkassel or Yuzhny Oleni Ostrov groups (Data S3.D).

There are 14 populations best fitted as a mixture between *Oberkassel* and *Sidelkino*, and four populations as a mixture between *Oberkassel* and Anatolia Neolithic farmer ancestries. The modelling result is further confirmed by two-way admixture modelling of Oberkassel + Yuzhny Oleni Ostrov or Oberkassel + Neolithic farmers from present-day Turkey. The *Oberkassel*/*Sidelkino* admixed populations are mostly dated to around or after 8,000 BP, except for the two oldest *Sidelkino* populations (Minino A, Peschanitsa) from Russia, dated to before 10,000 BP. The individual from Wöllersdorf, Austria, dated to 8,800 BP is also modeled as *Oberkassel* ancestry with a minor *Sidelkino* component. However, we estimated ~6-8% human DNA contamination in this genome (Data S1.D), which may affect the qpAdm modelling since the PMD-filtered genotype of this individual requires only *Oberkassel* ancestry. We therefore cautiously exclude this individual from the plot in Fig. 5A.

The four *Oberkassel*/Anatolia Neolithic farmer admixed populations include one ~7,600 BP population from Koros in Hungary, where hunter-gatherers were found to co-exist with Neolithic farmers and recent admixture was detected [42]. The other three populations were all from Middle Neolithic Germany after 6,000 BP, including one published individual from Blatterhole and two newly reported individuals from Wehye-Dreye and Ostorf.

The remaining 21 populations are fitted as three-way admixtures, including most hunter-gatherer populations from the Balkans (Iron Gates), Ukraine, Scandinavia and the Baltics. Considering the age of many Iron Gates, Ukrainian and Scandinavian individuals being older than 8,000 BP, it is unlikely that the early European Neolithic farmers reached those regions by then. Also, the three-way model does not fit well with some of these populations, like Romania_IronGates and Ukraine_N. Therefore, we speculate that either Oberkassel or Yuzhny Oleni Ostrov are not suitable source populations for the ancestry found in groups from these areas. Previous studies have revealed an affinity between Epigravettian-related ancestry and Near Eastern populations [36,37]. The affinity to the Anatolia Neolithic farmer ancestry observed in groups predating the Neolithic farming expansion in Europe might also be explained by an unsampled hunter-gatherer genetic component distributed in southeastern or eastern Europe, which contributed to the Mesolithic/Neolithic hunter-gatherers from

southeastern, eastern and northeastern Europe. Currently, the genetic make-up of Epigravettian-associated hunter-gatherers from the Balkans is still unknown and, therefore, future genomic studies are necessary to delineate the genetic history of those regions.

There are also three populations younger than ~6,500BP from central and eastern Europe that were modelled as three-way admixtures, namely the Murzihinskiy population from the Russian Republic of Tatarsta, and the youngest Ostorf 2 and Ostorf 3 individuals (OST002, OST003) from Germany. Those groups attest both to the expansion of farmer-related ancestry across Europe and a higher interaction between European hunter-gatherer populations in the 7th-6th millennium BP.

The nine Iberian populations are modelled as four-way admixtures of Arene Candide 16 + Goyet Q-2 + Yuzhny Oleni Ostrov + Neolithic farmers from present-day Turkey. After checking for nested models, five of them could be modelled as Arene Candide 16 + Goyet Q-2, and the other four youngest populations as Arene Candide 16 + Goyet Q-2 + Yuzhny Oleni Ostrov. This revealed the arrival of the *Sidelkino* ancestry in Iberia as early as ~7,800 BP. These nested models were further confirmed with two-way or three-way models, respectively.

Based on the above analyses, we plot the *Oberkassel*/*Sidelkino*/Anatolia Neolithic farmer ancestry proportions for all populations as pie charts with geographic locations and sample dates (Fig. 5). For the Iberian populations, we plot the Goyet Q-2 proportions in addition to the Oberkassel ancestry. We use the estimations from the simplest two-way or three-way models as the ancestry proportions for plotting. The sample date for each population is the average sample date of all individuals in this group. For genetic populations including samples from more than one geographic location, the same ancestry proportion is plotted for all the different sampling sites. The dates, geographic coordinates and ancestry proportions are summarized in Data S3.F.

Exploring the formation of the *Sidelkino* ancestry

In the previously described ancestry modelling, we find the two oldest *Sidelkino* cluster groups (>10k BP) from the Upper Volga region in western Russia showing extra affinity to the *Oberkassel* ancestry compared to other *Sidelkino* cluster populations. Those are the previously published ~12,600 BP individual from the Peschanitsa site (PES) [43], and the newly reported Minino A population including the six oldest individuals from the Minino I-II sites dated to ~10,600 BP (Fig. 1A). However, another almost contemporaneous individual from the Samara region (Sidelkino dated to ~11,300 BP) does not show such extra affinity.

The *Sidelkino* ancestry profile was reported to be associated with both the Ancient North Eurasian (ANE) and *Oberkassel* (WHG)/*Villabruna* ancestries [44]. We thus use f_4-statistics, qpAdm and DATES to explore the formation of *Sidelkino* ancestry, and test if there is a recent admixture signal between the ANE and *Oberkassel* ancestries in the three pre-10k *Sidelkino*-related individuals/groups (Peschanitsa, Sidelkino and Minino A).

With f_4-statistics in the form f_4(Mbuti, *Sidelkino*; *Villabruna*, *Oberkassel*), we find that individuals/groups in the *Sidelkino* cluster are equally related to the *Villabruna* and *Oberkassel* ancestries (Data S2.K). We therefore model the *Sidelkino* individuals/groups using either the Villabruna individual or the Oberkassel group as one source, and the Afontova Gora 3 individual as the other source. We use an outgroup set of ten populations (OG10_2) after removing Villabruna from the OG11 list. Only individuals/groups older than 8 ka are involved in this analysis, thus predating the post-8 ka contacts between the *Oberkassel* and *Sidelkino* clusters reported in this study.

We find that most populations are estimated to carry ~30% *Villabruna/Oberkassel* ancestry and 70% ANE ancestry, similar to previous studies [44], with Peschanitsa and Minino A carrying a slightly higher *Villabruna/Oberkassel* ancestry estimated to ~37-38% (Data S3.E). However, the modelling fails for almost all source and population combinations ($p << 0.05$), suggesting that neither *Villabruna* nor

Oberkassel appropriately represent the genetic ancestry that contributed to the *Sidelkino* ancestry. We speculate that the expansion of a yet unsampled Epigravettian-related population might have influenced the genetic profile of eastern European hunter-gatherers. While in western and central Europe we observe a large-scale genetic replacement with the arrival of the *Oberkassel* ancestry, in eastern Europe there was possibly a substantial admixture with the ANE ancestry resulting in the formation of the *Sidelkino* cluster.

For the DATES analyses, we pool the populations carrying either *Oberkassel*, *Villabruna* or ANE ancestries to increase the resolution as follows:

Oberkassel_pool: 11 *Oberkassel* cluster individuals older than 10,000 BP, including Bichon, Ranchot 88, Rochedane, Abri des Autours 3, Achères 2002, Farman, Malonne Petit Ri 1, Oberkassel (N=2), Waulsort Cave X (N=2);

Villabruna_pool: 11 *Villabruna* cluster individuals, including Villabruna 1, Continenza R7, R5, R11, Uzzo 50-54, Uzzo 96, Oriente C, San Teodoro 2, Arene Candide 16, Pradis 1, Tagliente 2;

ANE_pool: Three ANE cluster individuals, including Mal'ta1, Afontova Gora 3, Afontova Gora 2.

The admixture event in these three populations is dated to around 70-110 generations before the date of the tested populations, with both Oberkassel_pool + ANE_pool, or Villabruna_pool + ANE_pool (Table S3). Using a generation time of 29 years and the average dates of the tested populations, the admixture events are estimated to have happened in a similar time range for all tested groups, that is between 15 ka and 13 ka (Extended Data Fig. 7). Interestingly, this time range coincides with the arrival of the *Oberkassel* ancestry in central Europe, suggesting that the *Sidelkino* and *Oberkassel* ancestries might have formed around the same time. These two highly distinct ancestries remained largely separated in their respective geographic regions until as late as ~8 ka, when we start observing east-west admixture events (Figure 5A).

Table S3. Dating of the admixture in the earliest hunter-gatherers carrying *Sidelkino* ancestry.

Ancestry	Pop	Overall (gen)	jmean (gen)	SD (gen)	jmean (year)	SD (year)	Sample Age (yBP)	Admix Date (yBP)
Oberkassel+ ANE	Peschanitsa	84	75	27	2179	786	12656	14834
	Sidelkino	86	76	36	2209	1036	11259	13468
	Minino A	110	109	15	3160	441	10637	13797
Villabruna+ ANE	Peschanitsa	93	87	27	2516	12656	15172	789
	Sidelkino	99	90	33	2599	11259	13858	963
	Minino A	111	113	37	3268	10637	13905	1083

Section 12: Phenotypic analysis of European hunter-gatherers

We estimate the allele frequencies of phenotype-associated SNPs in ancient European hunter-gatherers to investigate the phenotype variations among them. As the sequencing coverage for ancient individuals is mostly not enough to reliably call diploid genotypes, we apply the strategy used in [27]. We count the reads mapped to different alleles for each individual and estimate the population-based maximum likelihood allele frequencies.

We consider 334 individuals in the analysis, including 102 newly reported and 232 published individuals, and divide them into different groups based on previously attested genetic ancestries (Data S1.J). The bam files of the published individuals are downloaded from public repositories for re-genotyping. We select eleven phenotypic SNPs for this analysis, which were previously described in Mathieson et al., 2015 [20] as showing strong selection signals in European populations. Details of the selected SNPs are listed in Data S3.G.

We pile-up the sequencing reads on selected positions using samtools mpileup, with parameters "-q30 -Q30" to filter out reads with mapping quality and base quality below 30. Then the numbers of forward and reverse reads mapping to both REF (reference) and ALT (alternative) alleles are extracted using BCFtools 1.3 [45]. To reduce the impact of post-mortem damage typical of ancient DNA, we apply different filters for bam files produced with different library protocols. For double-stranded libraries with partial-UDG treatment (ds_halfUDG), we trim 2 bp from both sides of the reads before pile-up. For double-stranded libraries without UDG treatment, we use untrimmed bam files for mpileup and only consider the reads mapped to transversion sites. For single-stranded libraries, we use untrimmed bam files for mpileup and remove the forward reads mapped to C/T variants, and reverse reads mapped to G/A variants. For individuals with more than one library, we calculate the individual read counts as the sum of read counts from all libraries.

After obtaining read counts on REF/ALT alleles from all individuals, we calculate the likelihood *L(p)* of a population with *N* individuals, with given population alternative allele frequency p, as:

$$L(p) = \prod_{i=1}^{N} \left\{ \binom{j_i+k_i}{k_i} p^{k_i} (1-p)^{j_i} \right\}$$

where for individual i, we observe j_i reads with the reference allele and k_i reads with the alternative allele. The maximum likelihood estimation of allele frequency is calculated for each population, and the 95% confidence interval (CI) is estimated as the allele frequencies with log-likelihood $l(p) = l_{max} - 1.92$.

We estimate the allele frequencies in eight hunter-gatherer groups: pre-LGM (N=42), *Villabruna* (N=9), El Mirón/*GoyetQ2* (N=11), *Oberkassel* (N=78), *Sidelkino* (N=43), Iron Gates HG (N=45), Ukraine HG (N=42), Baltic HG/Scandinavian HG (SHG) (N=53). The ANE and Caucasus HG groups are not included in this analysis due to the limited sample size, and neither are seven *Oberkassel* cluster individuals carrying admixture with the Anatolia Neolithic farmer ancestry, namely two ~7,600 BP individuals from Koros, Hungary, and five individuals from Germany younger than 6,000 BP (Supplementary section 11, Data S3.E).

Fig. S27 shows the likelihood curves of each hunter-gatherer group on the SNP rs12913832 located in the gene *HERC2*, as an example of how the maximum likelihood estimation and 95% CI are calculated. The alternative allele on this SNP is associated with the blue/green eye color phenotype present in high frequency in modern-day northern Europeans [46,47]. The summary of this estimation is also presented in Fig. 5B. Based on the group-based analysis, we suggest that the blue-eye phenotype first appeared in Europe in Epigravettian-associated populations carrying *Villabruna* ancestry, with an almost fixed alternative allele frequency at 92.6% (95% CI 78.8-98.7%). This phenotype was

inherited by hunter-gatherers with *Oberkassel* (WHG) ancestry and is also present in high frequency among Baltic HG and SHG who carry a mixed ancestry between *Oberkassel* and *Sidelkino*.

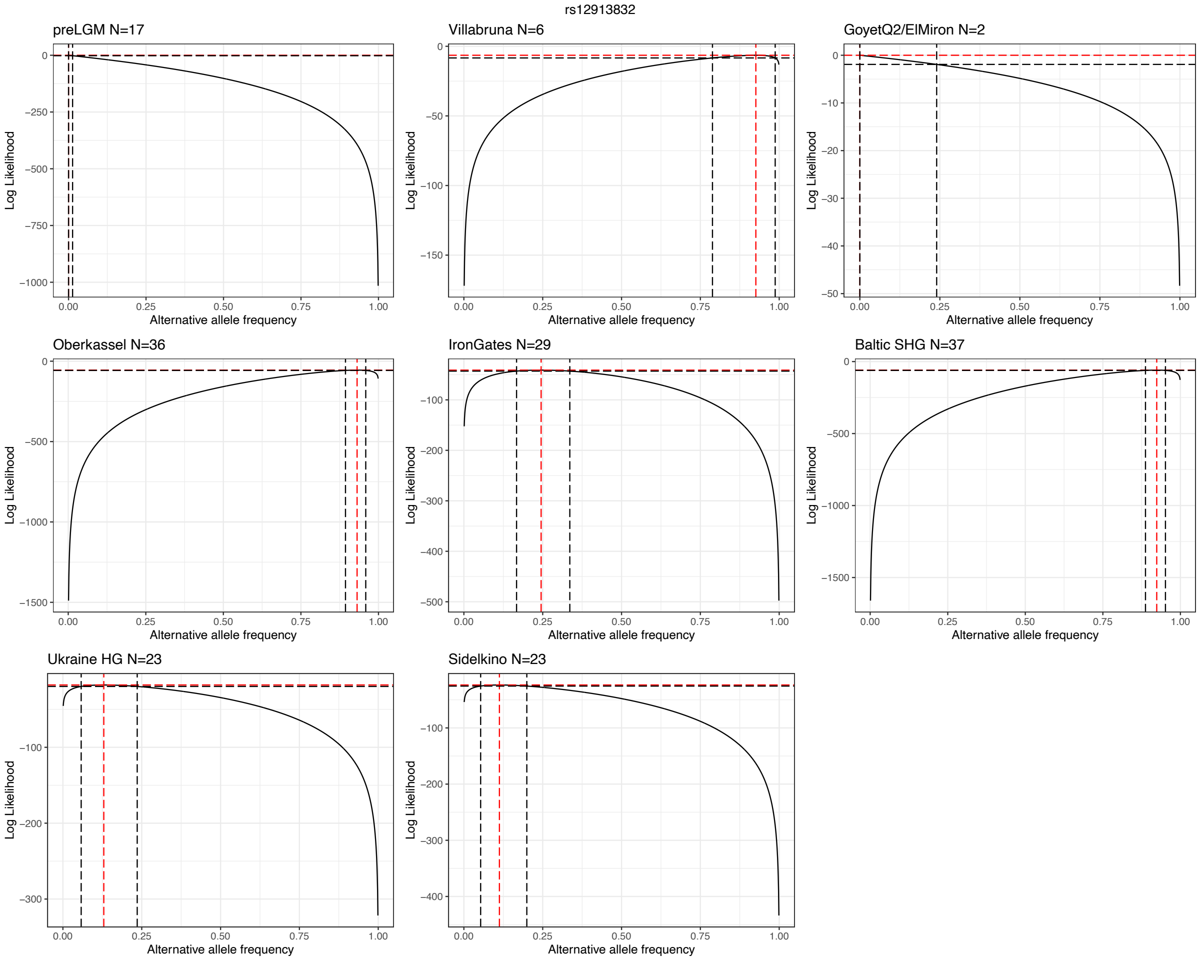

Figure S27. Likelihood curves of the alternative allele frequency on rs12913832, calculated in different hunter-gatherer groups. The x-axis shows the alternative allele frequency and the y-axis shows the log likelihood. The individual number with reads that covered the site in each group is shown in the upper-left corner. The red dashed line shows the allele frequency with the highest likelihood and the black dashed lines show the allele frequencies corresponding to the 95% CI.

We summarize the estimations of three additional SNPs related to skin color (two SNPs) and lactase persistence (one SNP) in Fig. 5B. We find that none of the hunter-gatherer groups carries the derived alleles on rs4988235 on the *LCT* gene related to lactase persistence, confirming that the emergence and selection on this SNP did not take place in European hunter-gatherers. On the contrary, we find a marked frequency discrepancy in both skin color-related SNPs (rs16891982 and rs1426654) between the *Oberkassel* and *Sidelkino* groups, suggesting different phenotypes between these roughly contemporaneous groups.

The estimations of the seven other SNPs investigated in Mathieson et al., 2015 [20] are summarized in Fig. S28. These SNPs are related to various functions including fatty acid metabolism (FADS1/2, rs174546), immunity (TLR, rs4833103 and MHC, rs2269424), vitamin D metabolism (DHCR7, rs7944926), coeliac desease (ATXN2/SH2B3, rs653178), ergothioneine transport (SLC22A4, rs272872) and skin pigmentation (GRM5, rs7119749). Among those SNPs, a marked difference in allele frequencies between *Villabruna/Oberkassel* and *Sidelkino* groups is observed at the GRM5 gene confirming the results from other two skin color-related SNPs and for the FADS1/2 gene, which is known to be related to diet.

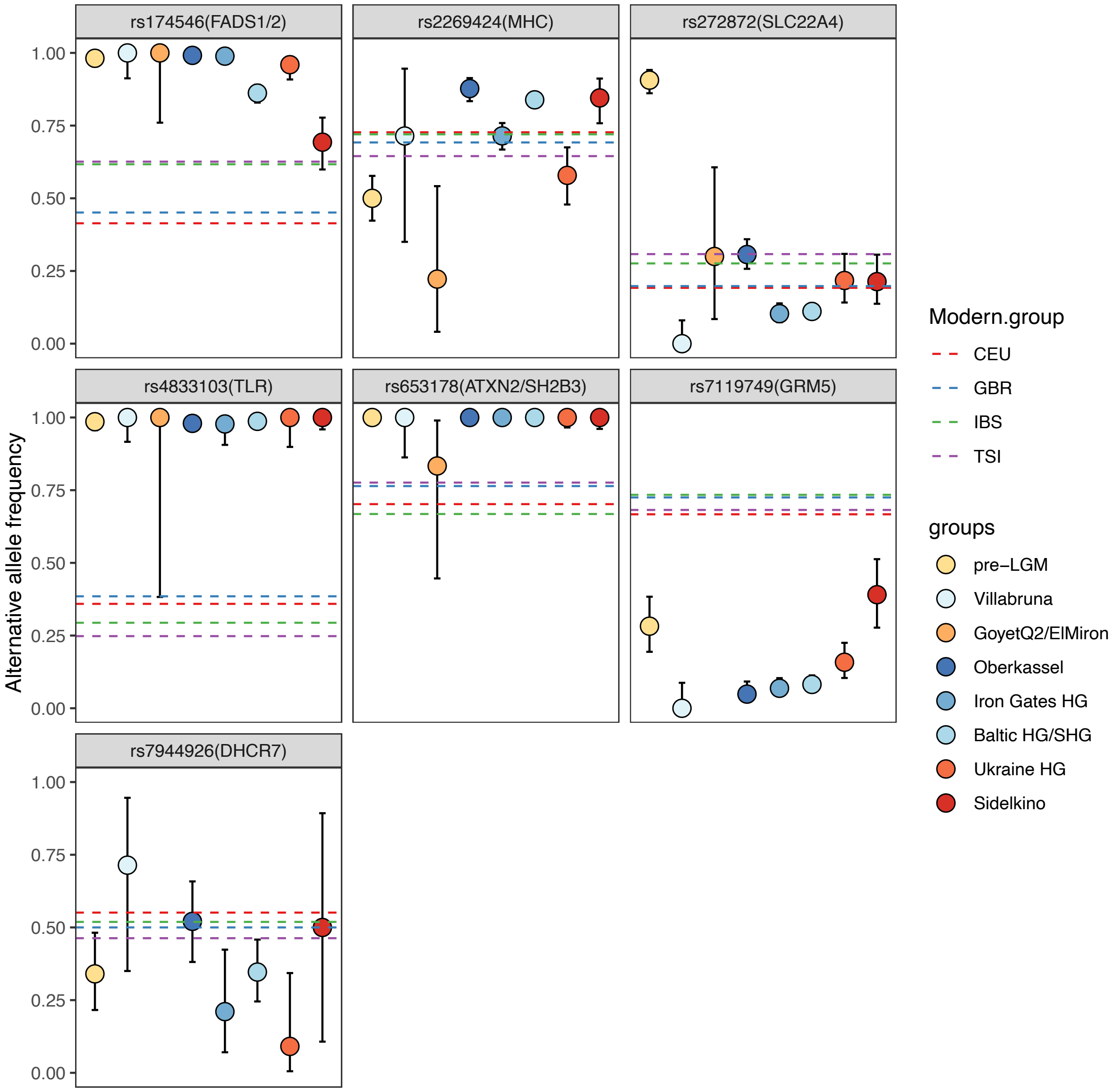

Figure S28. Allele frequencies on selected SNPs in different hunter-gatherer populations. This figure shows the allele frequency distribution of the analyzed phenotypic SNPs not included in Fig. 5. The dots and error bars show the maximum likelihood estimates and 95 % confidence intervals of the alternative allele frequencies (n=number of individuals in each group provided in Data S3.G).

Section 13: Mortuary practices of genetically analyzed Upper Paleolithic humans

In order to investigate if the differences in genetic ancestry affinities observed between the analyzed Upper Paleolithic humans from Europe are related to their cultural identities, we looked at their mortuary practices since they may reflect the social organisation of human populations [48]. We summarized the contextual information for the genetically analyzed specimens in this study (both newly reported and previously published Upper Paleolithic individuals from Europe), and conducted a principal component analysis (PCA) on the variables related to mortuary practices.

Specimens older than 40 ka from Bacho Kiro, Zlatý kůň, and the Peştera cu Oase were not included in the analysis because they represent basal non-African lineages not specifically related to each other [13,14]. Moreover, the pre-30 ka specimens Bacho Kiro 1653, Muierii 2, Cioclovina 1, and Paglicci 133 were not included in the analysis since they do not share distinctive affinities to the two main pre-30 ka genetic clusters (*GoyetQ116-1* and *Kostenki*).

Two other specimens were not included in the analysis: the Gravettian deciduous tooth from Ormesson and the Epigravettian deciduous tooth from Pradis. The two deciduous teeth appear to have been lost during the life of the individuals they belonged to [49,50], hence they do not come from mortuary contexts.

Additionally, contextual information was not secure enough to unambiguously assign four more specimens to the categories of mortuary practices we defined and they were excluded from our analysis. These were the Early Upper Paleolithic femoral diaphysis Sunghir 4, the Gravettian femoral fragment Dolní Věstonice 43, the Epigravettian (or Early Mesolithic) teeth Uzzo 50-54, and the Epigravettian hemimandible Tagliente 2 [51-54].

All of the other specimens (n = 48) dating from ~40 ka to ~12 ka (with the exception of individuals from present-day Italy dated until 9 ka from Continenza and Uzzo) and associated to the pre-14 ka genetic clusters discussed in this study (*GoyetQ116-1*, *Kostenki*, *Věstonice*, *Věstonice/Fournol*, *Fournol*, *Fournol/ElMirón*, *GoyetQ2*, ElMirón, and *Villabruna*) are included in the PCA.

The following variables pertaining to mortuary practices were coded following Duday's [55] terminology as either present (1) or absent (0) for each specimen analyzed based on a review of the available literature [14,21,31,53,56-85]: Cave burial (B-Cave), Open-air burial (B-Open), Grave goods and/or personal ornaments (GGO), Ochre (O), Cave anthropic deposit (A-Cave), Open-air anthropic deposit (A-Open), Anthropogenic marks (AG-mark), Evidence of cannibalism (Cannibalism).

The principal component analysis was computed based on the correlation matrix with Statistica 14.0 (StatSoft Inc., 2020). The resulting matrix is reported in Data S3.H, while the factor coordinates of the individuals included in the analysis are reported in Table S4 (PC1 and PC3) and Data S3.I (PC1 through PC7).

Table S4. Factor coordinates of each individual included in the mortuary practices PCA for the first and third principal components.

Specimens	Genetic cluster	Geographical origin	PC1	PC3
Goyet Q116-1	*GoyetQ116-1*	CWE	1.47679	0.13488
Goyet Q376-3	*GoyetQ116-1*	CWE	1.47679	0.13488
Kostenki 12	*Kostenki*	EE	-0.2929	1.57488
Kostenki 14	*Kostenki*	EE	-1.22304	1.51838
Sunghir 1	*Kostenki*	EE	-2.13735	1.60096
Sunghir 2	*Kostenki*	EE	-2.13735	1.60096
Sunghir 3	*Kostenki*	EE	-2.13735	1.60096

Dolní Věstonice 13	*Věstonice*	CEE	-2.13735	1.60096
Dolní Věstonice 14	*Věstonice*	CEE	-2.13735	1.60096
Dolní Věstonice 15	*Věstonice*	CEE	-2.13735	1.60096
Dolní Věstonice 16	*Věstonice*	CEE	-2.13735	1.60096
Krems-Wachtberg ind3	*Věstonice*	CEE	-2.13735	1.60096
Ostuni 1	*Věstonice*	SE	-2.08374	-1.45971
Paglicci 12	*Věstonice*	SE	-2.08374	-1.45971
Pavlov 1	*Věstonice*	CEE	-1.20721	1.65746
Goyet 2878-15	*Věstonice/Fournol*	CWE	1.47679	0.13488
Goyet 2878-18	*Věstonice/Fournol*	CWE	2.15665	-0.37565
Goyet Q-1	*Věstonice/Fournol*	CWE	2.15665	-0.37565
Goyet Q100-8	*Věstonice/Fournol*	CWE	1.47679	0.13488
Goyet Q376-19	*Věstonice/Fournol*	CWE	2.15665	-0.37565
Goyet Q53-1	*Věstonice/Fournol*	CWE	1.47679	0.13488
Goyet Q56-16	*Věstonice/Fournol*	CWE	1.47679	0.13488
Fournol 85	*Fournol*	WE	2.15665	-0.37565
La Rochette	*Fournol*	WE	1.47679	0.13488
Le Piage II	*Fournol*	WE	1.47679	0.13488
Mollet III GER-33	*Fournol*	SWE	-1.1536	-1.4032
Reclau Viver GER-8	*Fournol*	SWE	0.56249	0.21746
Brillenhöhle	*GoyetQ2*	CWE	2.24069	0.75893
Burkhardtshöhle	*GoyetQ2*	CWE	1.47679	0.13488
Goyet Q-2	*GoyetQ2*	CWE	0.54665	0.07838
Hohle Fels 10	*GoyetQ2*	CWE	2.24069	0.75893
Hohle Fels 49	*GoyetQ2*	CWE	2.24069	0.75893
Hohle Fels 79	*GoyetQ2*	CWE	2.24069	0.75893
La Marche LMR-B113-115	*GoyetQ2*	WE	2.15665	-0.37565
Maszycka 1/1	*GoyetQ2*	CEE	1.47679	0.13488
Maszycka 1/5	*GoyetQ2*	CEE	2.15665	-0.37565
Pincevent	*GoyetQ2*	CWE	0.30301	-1.81891
Rigney 1	*GoyetQ2*	CWE	1.22652	-0.43215
El Mirón 1	ElMirón	SWE	-1.16943	-1.54229
Arene Candide 16	*Villabruna*	SE	-1.16943	-1.54229
Grotta Continenza R11	*Villabruna*	SE	-2.08374	-1.45971
Grotta Continenza R15	*Villabruna*	SE	-2.08374	-1.45971
Grotta Continenza R7	*Villabruna*	SE	-2.08374	-1.45971
Oriente C	*Villabruna*	SE	-2.08374	-1.45971
San Teodoro 2	*Villabruna*	SE	-1.16943	-1.54229
Uzzo 96	*Villabruna*	SE	-0.23929	-1.48579
Villabruna 1	*Villabruna*	SE	-2.08374	-1.45971

The bivariate projection of the first and third principal components explains 56.7 % of the total variance (Fig. S29). As illustrated by the projection of the variables on the correlation circle, which allows to assess the weight of each variable on the two PCs, the first principal component is positively correlated with A-Cave, AG-mark and Cannibalism, and negatively correlated with O, GGO and burial contexts (B-Cave and B-Open) (Fig. S30). The third PC is mainly influenced by burial contexts (positive correlation with B-Open and negative correlation with B-Cave).

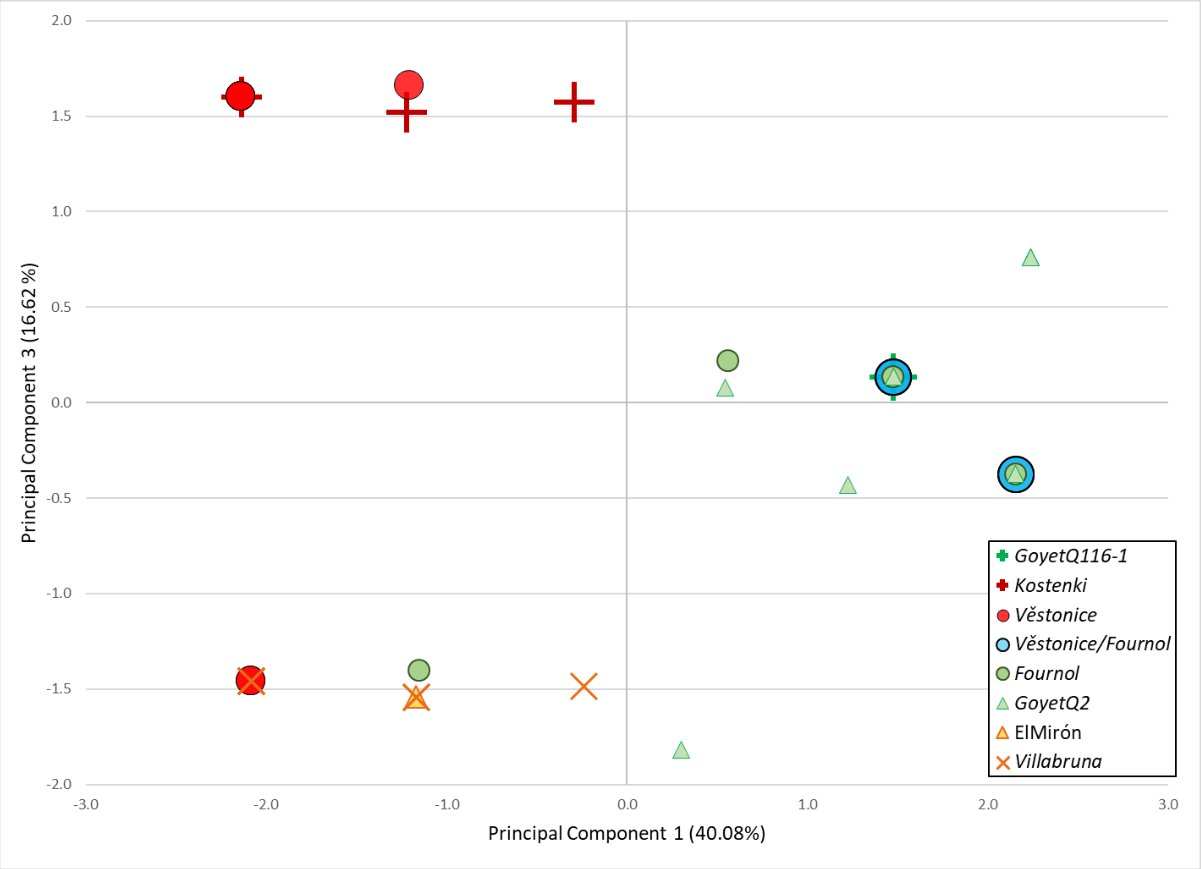

Figure S29. Projection on PC1 and PC3 of the genetically analyzed Upper Paleolithic humans coded by their genetic clusters.

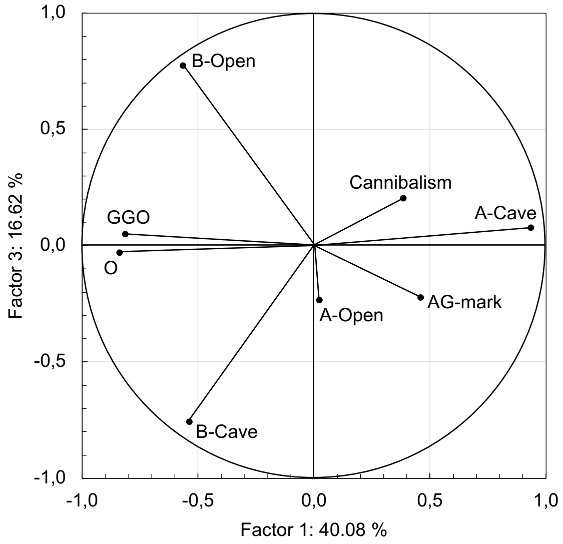

Figure S30. Correlations between the mortuary practices variables and PC1 and PC3 of the Principal Component Analysis.

The projection of the specimens on the two principal components shows a clear separation along the first axis (PC1) between individuals related to the two main pre-30 ka genetic ancestries discussed in this study and coded using green and red colors (Fig. S29). For instance, the *GoyetQ116-1* ancestry is in green, like other subsequent clusters that exhibit a higher affinity to this group (namely, *Fournol*

and *GoyetQ2*). Similarly, the *Kostenki* ancestry is represented in red, like the subsequent *Věstonice* cluster that shows genetic similarity with this group.
Each specimen was identified *a posteriori* according to their genetic cluster, and specimens coming from the same site and having been subjected to similar mortuary practices are represented by a single point on the graph.
The correlation between PC1 and genetic clustering is further illustrated in Fig. S31. Specimens carrying *GoyetQ116-1*-related ancestries, including the *GoyetQ116-1*, *Fournol*, and *GoyetQ2* clusters, tend to show higher factor coordinates on PC1, which correspond mostly to intentional cave deposits often associated to anthropogenic marks and evidence of cannibalism. The specimens carrying *Kostenki*- and *Villabruna*-related ancestries, including the *Věstonice* cluster (which carries a high proportion of *Kostenki* ancestry) and the El Mirón individual (which can be modelled as a mixture between *Villabruna* and *Fournol* ancestries), show negative factor coordinates on PC1 and come from burial contexts. Furthermore, they are subdivided into two groups along the second axis (PC3) in relation to either their cave or open-air context.
In order to add another level of interpretation, we used the same projection of PC1 and PC3 but identified the specimens based on their geographical origin only (Fig. S32). We divided the European continent in the same six zones as on the geographical map of Fig. 1: southwestern (SWE), western (WE), central-western (CWE), southern (SE), central-eastern (CEE), and eastern (EE) Europe.
The individuals associated with the *Villabruna* cluster (coming from southern Europe), whose genetic origins are not well understood, plot in the negative values of PC1, like the *Kostenki* and *Věstonice* groups, and more specifically with the *Věstonice* cluster individuals from southern Europe. However, we are unable to assess if the similarities in mortuary practices are related to a portion of shared genetic ancestry or to the transmission of cultural practices in a specific geographic area.

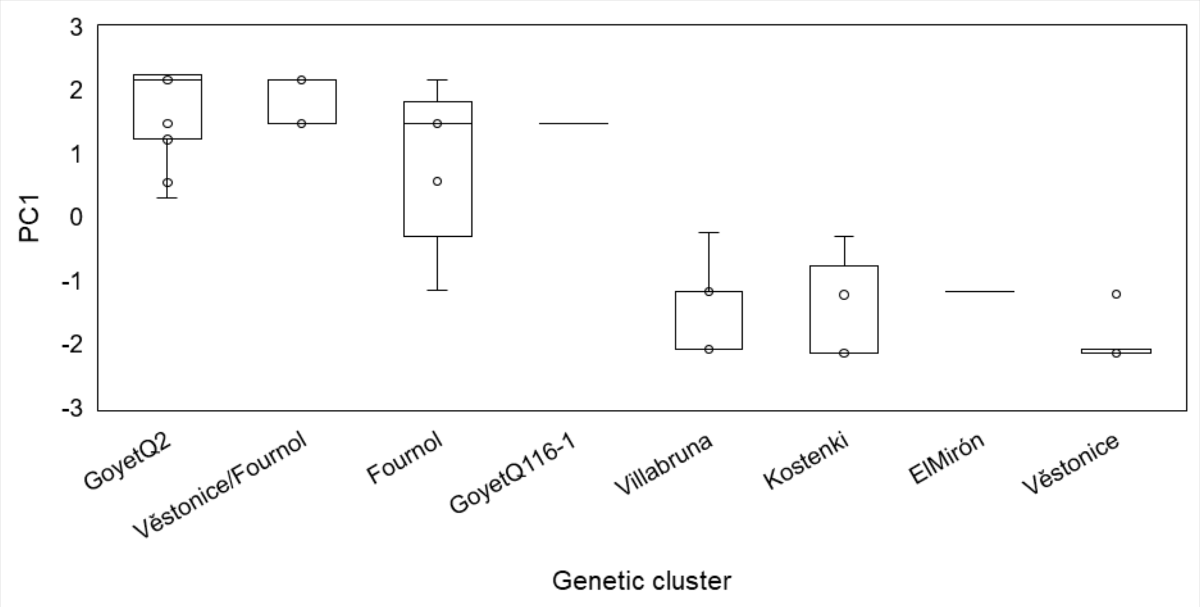

Figure S31. Boxplots of the PC1 factor coordinates of the individuals included in the mortuary practices PCA and grouped according to their genetic clusters. The box plot shows the median as the box center, interquartile range (IQR, defined by upper quartile [Q3] and lower quartile [Q1]) as the box bounds, maximum and minimum excluding outliers as the whisker bounds, and the sample site (n) being the individual number in each group.

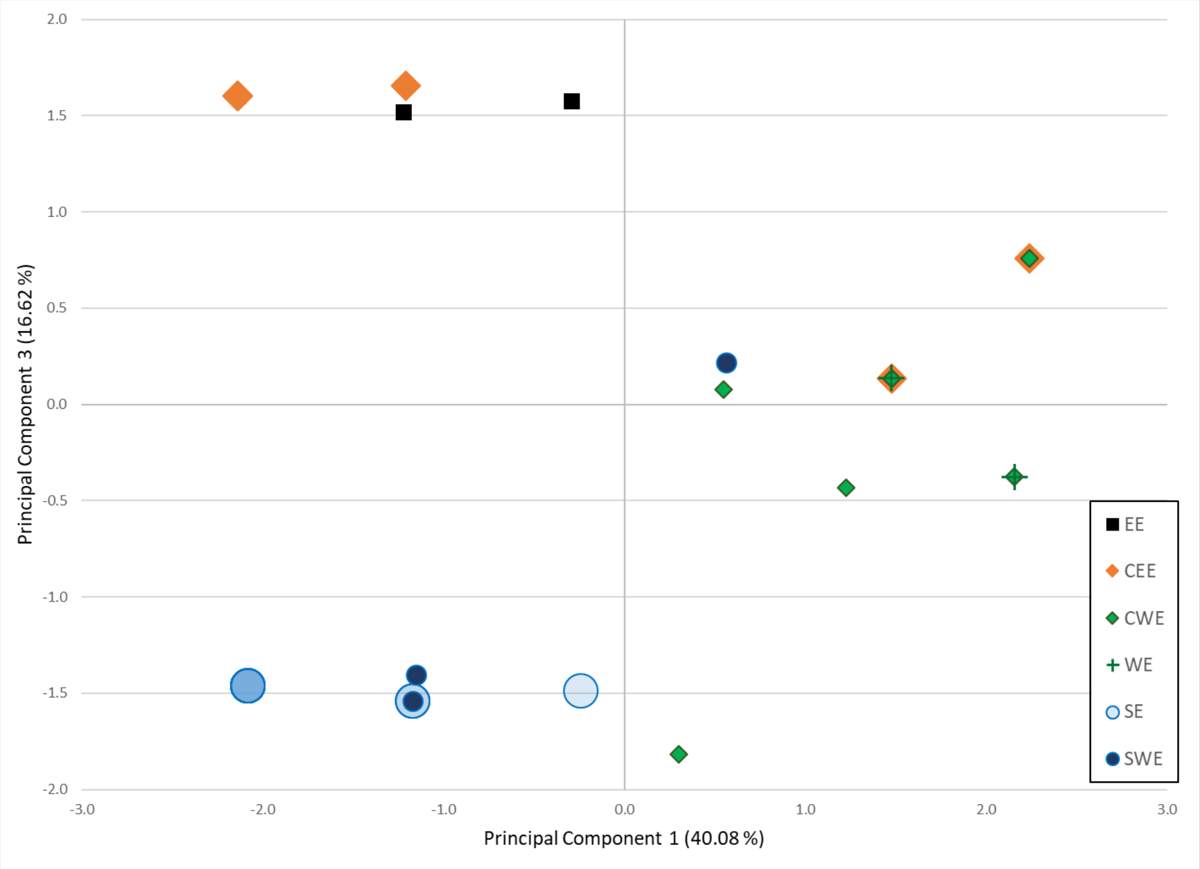

Figure S32. Projection on PC1 and PC3 of the genetically analyzed Upper Paleolithic humans coded by their geographical origins.

Focusing on the Gravettian-associated individuals, we see that the identified ancestry differences between the *Věstonice* and *Fournol* clusters are mirrored by cultural distinctions related to mortuary practices. We note that the individuals admixed between the *Věstonice/Fournol* clusters (namely the Goyet Gravettian specimens) are located in the PCA projection with the *Fournol* group rather than the *Věstonice* group. The geographical proximity between these Goyet individuals (from central-western Europe) and the specimens from the *Fournol* group (found in western and central-western Europe) could indicate that, despite admixture with incoming *Věstonice*-related ancestry populations, the Goyet Gravettian population retained local mortuary practices traditions related to deeper local ancestry linked to the *GoyetQ116-1* cluster. The only specimen from the *Fournol* cluster that plots in the negative part of PC1, close to the *Věstonice* cluster individuals from southern Europe, is Mollet III (Spain) (Fig. S29). This individual together with the specimen from Reclau Viver (which instead plots in the positive part of PC1) represent the only two Gravettian specimens genetically sequenced to date located in southwestern Europe. Secure contextual information that would allow an attribution of the Reclau Viver specimen to the B-Cave category is currently missing (Soler Subils, pers. comm.), which led us to keep it in the A-Cave group. Therefore, more skeletal remains are necessary to understand if the particular position of Mollet III may be related to a specific pattern. Instead, the mortuary practices of most *GoyetQ116-1* and *Fournol* cluster individuals show similarities with Madgalenian-associated individuals in the *GoyetQ2* cluster from across Europe, as observed at a genetic level.

The buried Gravettian-associated individuals belonging to the *Věstonice* cluster, which are related genetically to the *Kostenki* cluster are all found in the negative part of PC1, indicating similarities in mortuary practices. Moreover, they are divided into two groups along PC3 based on them being

buried in open-air sites or in cave contexts. Interestingly, this distinction follows a geographical pattern with the central-eastern and eastern European burial sites found in open-air contexts and southwestern and southern European burial grounds related to cave systems. Various biases and/or constraints may explain this observation, in particular geological constraints (*e.g.* the existence of cave systems, the nature of the soil) as well as other factors (*e.g.* historical, archaeological). However, multivariate analyses of the nature of grave goods associated with Gravettian burials from southern Europe on one hand, and central-eastern and eastern Europe on the other, did find significant cultural variation between the two groups, which could not be related to raw material availability [86], thus supporting the hypothesis of geographical funerary variability.
In addition, continuity through time regarding burial practices is also highlighted in southern and southwestern Europe since specimens from these regions are mainly positioned in the lower left quadrant of the PCA projection. For southern Europe, the mortuary practices affinities between the *Věstonice* and *Villabruna* clusters individuals contrast with the turnover observed at a genetic level between Gravettian- and Epigravettian-associated groups.
Accessing certain funerary treatments is conditioned by the identity of the deceased, whether biological and/or cultural, as perceived by the community in charge of the funeral [87]. As illustrated by Beau et al. [88], there are examples from Prehistoric times in which biological affinities were determinant criteria. Here, our results appear to indicate that the way people treated their dead in the European Upper Paleolithic may have transcended some aspects of their material cultures. Continuity in mortuary treatment sometimes aligns with genetic affinities through time and space, suggesting that some cultural beliefs of these hunter-gatherer groups may have persisted in some biologically related groups. Therefore, regardless of material culture changes and/or continuity, genetic affinities appear to not always be limited to biological transmission alone.
It is important to stress again that the analysis presented in this section is based only on genetically analyzed human remains. A broader analysis is warranted to assess the variety of mortuary practices in the European Upper Paleolithic, which are not necessarily captured in their entire complexity in the present investigation.

Supplementary References

1. Rasmussen, M. *et al.* An Aboriginal Australian Genome Reveals Separate Human Dispersals into Asia. *Science* **334**, 94–98 (2011).
2. Moreno-Mayar, J. V. *et al.* A likelihood method for estimating present-day human contamination in ancient male samples using low-depth X-chromosome data. *Bioinformatics* **36**, 828–841 (2020).
3. Huang, Y. & Ringbauer, H. hapCon: Estimating contamination of ancient genomes by copying from reference haplotypes. *bioRxiv* 2021.12.20.473429 (2021). doi:10.1101/2021.12.20.473429
4. Ringbauer, H., Novembre, J. & Steinrücken, M. Parental relatedness through time revealed by runs of homozygosity in ancient DNA. *Nat. Commun.* **12**, 5425 (2021).
5. Li, N. & Stephens, M. Modeling Linkage Disequilibrium and Identifying Recombination Hotspots Using Single-Nucleotide Polymorphism Data. *Genetics* **165**, 2213–2233 (2003).
6. Zhu, C., Byrd, R. H., Lu, P. & Nocedal, J. Algorithm 778: L-BFGS-B: Fortran Subroutines for Large-Scale Bound-Constrained Optimization. *ACM Trans. Math. Softw.* **23**, 550–560 (1997).
7. Virtanen, P. *et al.* SciPy 1.0: fundamental algorithms for scientific computing in Python. *Nat. Methods* **17**, 261–272 (2020).
8. Auton, A. *et al.* A global reference for human genetic variation. *Nature* **526**, 68–74 (2015).
9. Olalde, I. *et al.* The genomic history of the Iberian Peninsula over the past 8000 years. *Science* **363**, 1230–1234 (2019).
10. Huang, Y. & Ringbauer, H. hapCon: Estimating contamination of ancient genomes by copying from reference haplotypes. *Bioinformatics* **38**, 3768–3777 (2022).
11. Jónsson, H., Ginolhac, A., Schubert, M., Johnson, P. L. F. & Orlando, L. MapDamage2.0: Fast approximate Bayesian estimates of ancient DNA damage parameters. *Bioinformatics* **29**, 1682–1684 (2013).
12. Lazaridis, I. *et al.* Ancient human genomes suggest three ancestral populations for present-day Europeans. *Nature* **513**, 409–413 (2014).
13. Prüfer, K. *et al.* A genome sequence from a modern human skull over 45,000 years old from Zlatý kůň in Czechia. *Nat. Ecol. Evol.* **5**, 820–825 (2021).
14. Hajdinjak, M. *et al.* Initial Upper Palaeolithic humans in Europe had recent Neanderthal ancestry. *Nature* **592**, 253–257 (2021).
15. Mallick, S. *et al.* The Simons Genome Diversity Project: 300 genomes from 142 diverse populations. *Nature* **538**, 201–206 (2016).
16. Renaud, G., Hanghøj, K., Willerslev, E. & Orlando, L. gargammel: a sequence simulator for ancient DNA. *Bioinformatics* **33**, 577–579 (2017).
17. Li, H. Aligning sequence reads, clone sequences and assembly contigs with BWA-MEM. *arXiv* arXiv:1303.3997 (2013). doi:10.48550/ARXIV.1303.3997
18. Nakatsuka, N. *et al.* ContamLD: estimation of ancient nuclear DNA contamination using breakdown of linkage disequilibrium. *Genome Biol.* **21**, 199 (2020).
19. Korneliussen, T. S., Albrechtsen, A. & Nielsen, R. ANGSD: Analysis of Next Generation Sequencing Data. *BMC Bioinformatics* **15**, 1–13 (2014).
20. Renaud, G., Slon, V., Duggan, A. T. & Kelso, J. Schmutzi: Estimation of contamination and endogenous mitochondrial consensus calling for ancient DNA. *Genome Biol.* **16**, 1–18 (2015).
21. Peltzer, A. *et al.* EAGER: efficient ancient genome reconstruction. *Genome Biol.* **17**, 60 (2016).
22. Posth, C. *et al.* Pleistocene Mitochondrial Genomes Suggest a Single Major Dispersal of Non-Africans and a Late Glacial Population Turnover in Europe. *Curr. Biol.* **26**, 827–833 (2016).
23. Fowler, C. *et al.* A high-resolution picture of kinship practices in an Early Neolithic tomb. *Nature* **601**, 584–587 (2022).
24. Staubwasser, M. *et al.* Impact of climate change on the transition of Neanderthals to modern humans in Europe. *Proc. Natl. Acad. Sci. U. S. A.* **115**, 9116–9121 (2018).
25. Cooper, A. *et al.* A global environmental crisis 42,000 years ago. *Science* **371**, 811–818 (2021).

26. Haak, W. *et al.* Massive migration from the steppe was a source for Indo-European languages in Europe. *Nature* **522**, 207–211 (2015).
27. Mathieson, I. *et al.* Genome-wide patterns of selection in 230 ancient Eurasians. *Nature* **528**, 499–503 (2015).
28. Mathieson, I. *et al.* The genomic history of southeastern Europe. *Nature* **555**, 197–203 (2018).
29. Green, R. E. *et al.* A draft sequence of the Neandertal genome. *Science* **328**, 710–722 (2010).
30. Sankararaman, S. *et al.* The genomic landscape of Neanderthal ancestry in present-day humans. *Nature* **507**, 354–357 (2014).
31. Yang, M. A. & Fu, Q. Insights into Modern Human Prehistory Using Ancient Genomes. *Trends Genet.* **34**, 184–196 (2018).
32. Petr, M., Pääbo, S., Kelso, J. & Vernot, B. Limits of long-term selection against Neandertal introgression. *Proc. Natl. Acad. Sci. U. S. A.* **116**, 1639–1644 (2019).
33. Patterson, N. *et al.* Ancient admixture in human history. *Genetics* **192**, 1065–1093 (2012).
34. Fu, Q. *et al.* An early modern human from Romania with a recent Neanderthal ancestor. *Nature* **524**, 216–219 (2015).
35. Yang, M. A. *et al.* 40,000-Year-Old Individual from Asia Provides Insight into Early Population Structure in Eurasia. *Curr. Biol.* **27**, 3202-3208.e9 (2017).
36. Fu, Q. *et al.* The genetic history of Ice Age Europe. *Nature* **534**, 200–205 (2016).
37. Feldman, M. *et al.* Late Pleistocene human genome suggests a local origin for the first farmers of central Anatolia. *Nat. Commun.* **10**, 1–10 (2019).
38. Sikora, M. *et al.* Ancient genomes show social and reproductive behavior of early Upper Paleolithic foragers. *Science* **358**, 659–662 (2017).
39. Fernandes, D. M. *et al.* A genetic history of the pre-contact Caribbean. 1–42 (2020).
40. Posth, C. *et al.* Reconstructing the Deep Population History of Central and South America. *Cell* **175**, 1185-1197.e22 (2018).
41. Villalba-Mouco, V. *et al.* Survival of Late Pleistocene Hunter-Gatherer Ancestry in the Iberian Peninsula. *Curr. Biol.* **29**, 1169-1177.e7 (2019).
42. Lipson, M. *et al.* Parallel palaeogenomic transects reveal complex genetic history of early European farmers. *Nature* **551**, 368–372 (2017).
43. Saag, L. *et al.* Genetic ancestry changes in Stone to Bronze Age transition in the East European plain. *Sci. Adv.* **7**, eabd6535 (2021).
44. Lazaridis, I. *et al.* Genomic insights into the origin of farming in the ancient Near East. *Nature* **536**, 419–424 (2016).
45. Li, H. *et al.* The sequence alignment/map format and SAMtools. *Bioinformatics* **25**, 2078–2079 (2009).
46. Eiberg, H. *et al.* Blue eye color in humans may be caused by a perfectly associated founder mutation in a regulatory element located within the HERC2 gene inhibiting OCA2 expression. *Hum. Genet.* **123**, 177–187 (2008).
47. Sturm, R. A. *et al.* A single SNP in an evolutionary conserved region within intron 86 of the HERC2 gene determines human blue-brown eye color. *Am. J. Hum. Genet.* **82**, 424–431 (2008).
48. Pearson, M. P. *The Archaeology of Death and Burial* (Sutton, 2003).
49. Bodu, P. et al., in *Préhistoire de l'Europe du Nord-Ouest : Mobilités, Climats et Entités Culturelles. Volume 2 : Paléolithique Supérieur Ancien, Paléolithique Final - Mésolithique* (dir. Montoya, C., Fagnart, J.-P. & Locht, J.-L.) 231–261 (Actes du XXVIIIe Congrès Préhistorique de France, Société Préhistorique Française, 2019).
50. Lugli, F. et al., Tracing the mobility of a Late Epigravettian (~ 13 ka) male infant from Grotte di Pradis (Northeastern Italian Prealps) at high-temporal resolution. *Sci. Rep.* **12**, 8104 (2022).
51. Trinkaus, E. & Buzhilova, A. P. Diversity and differential disposal of the dead at Sunghir. *Antiquity* **92**, 7–21 (2018).
52. Trinkaus, E. et al., Human remains from the Moravian Gravettian: Morphology and Taphonomy of isolated elements from the Dolní Věstonice II site. *J. Archaeol. Sci.* **27**, 1115–1132 (2000).

53. Yu, H., van de Loosdrecht, M.S., Mannino, M.A., Talamo, S., Rohrlach, A.B., Childebayeva, A., Villalba-Mouco, V., Aron, F., Brandt, G., Burri, M., et al. Genomic and dietary discontinuities during the Mesolithic and Neolithic in Sicily. *iScience* **25**, 104244. (2022).
54. Bortolini, E. et al., Early Alpine occupation backdates westward human migration in Late Glacial Europe. *Curr. Biol.* **31**, 1–10 (2021).
55. Duday, H. *The Archaeology of the Dead: Lectures in Archaeothanatology* (Oxbow Books, 2009).
56. Alciati, G., Pesce Delfino, V. & Vacca, E. (eds.) *Catalogue of Italian Fossil Human Remains from the Palaeolithic to the Mesolithic* (Istituto Italiano di Antropologia, 2005).
57. Antonio, M. L. et al., Ancient Rome: A genetic crossroads of Europe and the Mediterranean. *Science* **366**, 708–714 (2019).
58. Bignon, O. (pers. comm.)
59. Borgognini Tarli, S., Canci, A., Piperno, M. & Repetto, E. Dati archeologici e antropologici sulle sepolture mesolitiche della Grotta dell'Uzzo (Trapani). *Bullettino di Paletnologia Italiana* **84, n.s. II**, 85–179 (1993).
60. Coppola, D. (ed.) *Il Riparo di Agnano nel Paleolitico Superiore. La Sepoltura Ostuni 1 ed i suoi Simboli* (Università di Roma Tor Vergata, 2012).
61. Cupillard, C. et al., Changes in ecosystems, climate and societies in the Jura Mountains between 40 and 8 ka cal BP. *Quat. Int.* **378**, 40–72 (2015).
62. Dinnis, R. et al., New data for the Early Upper Paleolithic of Kostenki (Russia). *J. Hum. Evol.* **127**, 21–40 (2019).
63. Gambier, D., Valladas, H., Tisnérat-Laborde, N., Arnold, M. & Bresson, F. Datation de vestiges humains présumés du Paléolithique supérieur par la méthode du Carbone 14 en spectrométrie de masse par accélérateur. *Paléo* **12**, 201–212 (2000).
64. Gazzoni, V. & Fontana, F. Quelle mort ? Quelle vie ? Pratiques funéraires et organisation sociale des chasseurs-cueilleurs de la péninsule italienne. *Bull. Mém. Soc. Anthropol. Paris* **23**, 52–69 (2011).
65. Graziosi, P. Gli uomini paleolitici della Grotta di S. Teodoro (Messina). *Riv. Sci. Preist.* **2**, 123–223 (1947).
66. Grifoni Cremonesi, R., Serradimigni, M. & Usala, M. in *Il Fucino e le Aree Limitrofe nell'Antichità* 27–39 (Atti del III Convegno di Archeologia in ricordo di Walter Cianciusi, Archeoclub d'Italia - Sezione della Marsica, 2011).
67. Henry-Gambier, D. in *Préhistoire entre Vienne et Charente : Hommes et Sociétés du Paléolithique* (eds. Buisson-Catil, J. & Primault, J.) 25–43 (Association des publications chauvinoises, 2010).
68. Klaatsch, H. & Lustig, W. Morphologie der paläolithisches skelettreste des mittleren Aurignacien des grotte von La Rochette, Dordogne. *Archiv für Anthropologie* **41**, 81–126 (1914).
69. Le Mort, F. & Gambier, D. in *Le Peuplement Magdalénien, Paléogéographie Physique et Humaine* (eds. Rigaud, J.-P., Laville, H. & Vandermeersch, B.) 29–40 (Editions du C.T.H.S., 1992).
70. Marom, A., McCullagh, J. S. O., Higham, T. F. G., Sinitsyn, A. A. & Hedges, R. E. M. Single amino acid radiocarbon dating of Upper Paleolithic modern humans. *Proc. Natl. Acad. Sci. U.S.A.* **109**, 6878–6881 (2012).
71. Mezzena, F. & Palma di Cesnola, A. Scoperta di una sepoltura gravettiana nelle Grotta Paglicci (Rignano Garganico). *Riv. Sci. Preist.* **27**, 27–50 (1972).
72. Orschiedt, J. Secondary burial in the Magdalenian: The Brillenhöhle (Blauberen, Southwest Germany). *Paléo* **14**, 241–256 (2002).

73. Orschiedt, J. et al., Human remains from Maszycka Cave (Woj. Malopolskie / PL): The treatment of human bodies in the Magdalenian. *Archäologisches Korrespondenzblatt* **47**, 423–439 (2017).
74. Riel-Salvatore, J. & Gravel-Miguel, C. in *The Oxford Handbook of the Archaeology of Death and Burial* (eds. Tarlow, S. & Nilsson Stutz, L.) 303–346 (Oxford University Press, 2003).
75. Rougier, H. et al., The first directly dated human fossil from the Solutrean in Southwestern Europe (Le Piage, Fajoles, SW France). *J. Hum. Evol.* (in prep.).
76. Rufí, I., Coromina, N., Soler, J., Solés, A. & Agustí, B. Les restes humanes gravetianes de Mollet III. Identificació i descoberta de les restes d'*Homo sapiens* més antigues de Catalunya. *Tribuna d'Arqueologia* **2016-2017**, 103–116 (2017).
77. Sala, N. & Conard, N. Taphonomic analysis of the hominin remains from Swabian Jura and their implications for the mortuary practices during the Upper Paleolithic. *Quat. Sci. Rev.* **150**, 278–300 (2016).
78. Saladié, P. & Rodríguez-Hidalgo, A. Archaeological Evidence for Cannibalism in Prehistoric Western Europe: from *Homo antecessor* to the Bronze Age. *J. Archaeol. Method Theory* **24**, 1034–1071 (2017).
79. Sinitsyn, A. A. in *La Spiritualité* (dir. Otte, M.) 237–244 (Actes du Colloque de la Commission 8 de l'UISPP (Paléolithique supérieur), ERAUL 106, 2004).
80. Sparacello, V. S. et al., New insights on Final Epigravettian funerary behavior at Arene Candide Cave (Western Liguria, Italy). *J. Anthropol. Sci.* **96**, 161–184 (2018).
81. Straus, L. G., Gonzalez Morales, M. R. & Carretero, J. M. (eds.) 'The Red Lady of El Mirón Cave': Lower Magdalenian Human Burial in Cantabrian Spain. *J. Archaeol. Sci.* **60**, 1–137 (2015).
82. Teschler-Nicola, M. et al., Ancient DNA reveals monozygotic newborn twins from the Upper Palaeolithic. *Commun. Biol.* **3**, 650 (2020).
83. Trinkaus, E. & Svoboda, J. (eds.) *Early Modern Human Evolution in Central Europe. The People of Dolní Věstonice and Pavlov* (The Dolní Věstonice Studies Volume 12, Oxford Univ. Press, 2006).
84. Vercellotti, G., Alciati, G., Richards, M. P. & Formicola, V. The Late Upper Paleolithic skeleton Villabruna 1 (Italy): a source of data on biology and behavior of a 14.000 year-old hunter. *J. Anthropol. Sci.* **86**, 143–163 (2008).
85. Villotte, S. et al., Evidence for previously unknown mortuary practices in the Southwest of France (Fournol, Lot) during the Gravettian. *J. Archaeol. Sci. Rep.* **27**, 101959 (2019).
86. d'Errico, F. & Vanhaeren, M. in Death Rituals, Social Order and the Archaeology of Immortality in the Ancient World (eds. Renfrew, C., Boyd, M. J. & Morley, I.) 45–62 (Cambridge University Press, 2015).
87. Schmitt, A., Déderix, S. & Crevecoeur, I. (eds.) *Gathered in Death. Archaeological and Ethnological Perspectives on Collective Burial and Social Organisation* (Actes de Colloques, Aegis 14, Presses Universitaires de Louvain, 2018).
88. Beau, A. et al., Multi-scale ancient DNA analyses confirm the western origin of Michelsberg farmers and document probable practices of human sacrifice. *PLoS ONE* **12**, e0179742 (2017).
