# Supplementary material for: Palaeogenomics of Upper Palaeolithic to Neolithic European hunter-gatherers
Source: Nature. 2023 Mar 1;615(7950):117–26. doi: 10.1038/s41586-023-05726-0 (PMC9977688; doi:10.1038/s41586-023-05726-0)
Supplement: Supplementary file 2 — Reporting Summary [file 41586_2023_5726_MOESM2_ESM.pdf]

nature research

Corresponding author(s): Cosimo Posth, He Yu, Johannes Krause

Last updated by author(s): 16/12/22

Reporting Summary

Nature Research wishes to improve the reproducibility of the work that we publish. This form provides structure for consistency and transparency in reporting. For further information on Nature Research policies, see our Editorial Policies and the Editorial Policy Checklist.

Statistics

For all statistical analyses, confirm that the following items are present in the figure legend, table legend, main text, or Methods section.

n/a	Confirmed	
☐	☒	The exact sample size (n) for each experimental group/condition, given as a discrete number and unit of measurement
☐	☒	A statement on whether measurements were taken from distinct samples or whether the same sample was measured repeatedly
☐	☒	The statistical test(s) used AND whether they are one- or two-sided<br>*Only common tests should be described solely by name; describe more complex techniques in the Methods section.*
☐	☒	A description of all covariates tested
☐	☒	A description of any assumptions or corrections, such as tests of normality and adjustment for multiple comparisons
☐	☒	A full description of the statistical parameters including central tendency (e.g. means) or other basic estimates (e.g. regression coefficient) AND variation (e.g. standard deviation) or associated estimates of uncertainty (e.g. confidence intervals)
☐	☒	For null hypothesis testing, the test statistic (e.g. F, t, r) with confidence intervals, effect sizes, degrees of freedom and P value noted<br>*Give P values as exact values whenever suitable.*
☒	☐	For Bayesian analysis, information on the choice of priors and Markov chain Monte Carlo settings
☒	☐	For hierarchical and complex designs, identification of the appropriate level for tests and full reporting of outcomes
☒	☐	Estimates of effect sizes (e.g. Cohen's d, Pearson's r), indicating how they were calculated

Our web collection on statistics for biologists contains articles on many of the points above.

Software and code

Policy information about availability of computer code

Data collection	AADR v42.4 (https://reich.hms.harvard.edu/allen-ancient-dna-resource-aadr-downloadable-genotypes-present-day-and-ancient-dna-data", version 42.4)<br>EAGER v.1.92.55<br>BWA v.0.7.12<br>circularmapper v.1.93.5<br>AdapterRemoval v.2.2.0<br>dedup v.0.12.1<br>mapDamage v.2.0.6 and 2.0.9<br>samtools v.1.3<br>pileupCaller v.1.4.0.2<br>PMDtools v.0.6<br>ANGSD v.0.934<br>hapCon v.0.1<br>contamLD v.0.1<br>newly developed code for contamination estimate (https://github.com/hyl317/hapROH and https://zenodo.org/record/7421149#.Y5Q7WuyZOWg)
Data analysis	OxCal v.4.4<br>schmutzi 1.0<br>HaploGrep2<br>EIGENSOFT v.6.0.1 (smartpca)<br>AdmixTools 5.1 (qp3Pop, qpDstat, qpF4Ratio, qpWave, qpAdm, qpGraph) and 7.0.2 (qpfstats)<br>hapROH 0.1<br>DATES 753

R 3.5.1
MUSCLE v.3
HaploGrep v.2
MEGA10
pathPhynder v1.a

For manuscripts utilizing custom algorithms or software that are central to the research but not yet described in published literature, software must be made available to editors and reviewers. We strongly encourage code deposition in a community repository (e.g. GitHub). See the Nature Research guidelines for submitting code & software for further information.

Data

Policy information about availability of data

All manuscripts must include a data availability statement. This statement should provide the following information, where applicable:

- Accession codes, unique identifiers, or web links for publicly available datasets
- A list of figures that have associated raw data
- A description of any restrictions on data availability

The human reference genome used in this study is version hs37d5 and the accession of human mitochondrial genome reference is NC_012920.1. The aligned sequences of all individuals with new genomic data reported in this study is available at the European Nucleotide Archive (ENA) under the study accession number PRJEB51862. The compiled genotype files used for analyses, including re-genotyped published genomes, is uploaded at the Edmond Data Repository of the Max Planck Society (https://edmond.mpdl.mpg.de/dataset.xhtml?persistentId=doi:10.17617/3.Y1KJMF).

Field-specific reporting

Please select the one below that is the best fit for your research. If you are not sure, read the appropriate sections before making your selection.

☒ Life sciences ☐ Behavioural & social sciences ☐ Ecological, evolutionary & environmental sciences

For a reference copy of the document with all sections, see nature.com/documents/nr-reporting-summary-flat.pdf

Life sciences study design

All studies must disclose on these points even when the disclosure is negative.

Sample size	No statistical methods were used to determine ancient DNA sample size a priori. The number of genomes analyzed in this study depends on available human remains associated with hunter-gatherer individuals with signature of preserved ancient DNA. Those specimens are very limited because of the scarce availability and poor molecular preservation of human remains from that period.
Data exclusions	For ancient DNA screening analysis, libraries with less than ~0.1% human DNA and/or with no ancient DNA damage pattern are not carried on for SNPs capture. For alignment, sequencing reads with fragment length <30bp and mapping quality/base quality <30 are excluded from genotyping. For ancient DNA authentication, libraries with indication of substantial contamination levels or individually-analyzed libraries with marginal contamination levels are filtered to analyze only sequencing reads that carry signs of post-mortem DNA damage. For whole genome analysis, individuals with less than 6,000 usable SNPs are excluded.
Replication	Multiple libraries (1-8) are prepared for a subset of samples as replication. The genotypes from different libraries (both single stranded and double stranded) are merged for downstream analysis after confirming of similar statistical behaviour. Each sample is analyzed for up to 1.24 Million markers across the human genome that represent an internal replication of the findings.
Randomization	Randomization is not relevant to this study. Samples are grouped based on sampling locations, dates and genetic affinities.
Blinding	Blinding is not applicable for ancient specimens as the sampling locations and dates are known as prior. In downstream data analysis blinding is also not relevant since the newly generated ancient genomes are co-analyzed with previously published present-day and ancient human genomes.

Reporting for specific materials, systems and methods

We require information from authors about some types of materials, experimental systems and methods used in many studies. Here, indicate whether each material, system or method listed is relevant to your study. If you are not sure if a list item applies to your research, read the appropriate section before selecting a response.

Materials & experimental systems

n/a	Involved in the study
☒	☐ Antibodies
☒	☐ Eukaryotic cell lines
☐	☒ Palaeontology and archaeology
☒	☐ Animals and other organisms
☒	☐ Human research participants
☒	☐ Clinical data
☒	☐ Dual use research of concern

Methods

n/a	Involved in the study
☒	☐ ChIP-seq
☒	☐ Flow cytometry
☒	☐ MRI-based neuroimaging

Palaeontology and Archaeology

Specimen provenance	Specimen provenance is described in Supplementary section 1 and Data S1.A. The skeletal remains analyzed in this study derive from multiple collections and museums. All specimens were sampled with the approval of the appropriate institutions for the handling of archaeological samples and/or in collaboration with local scientists and curators listed among the authors of this study.
Specimen deposition	Contact persons for each archaeological site/sample are listed in Supplementary Information section 1. The genetic libraries generated from DNA extracted from the skeletal remains are stored at the Max Planck Institute for Evolutionary Anthropology (MPI-EVA) in Jena and Leipzig, Germany.
Dating methods	In this study we report 47 new Accelerator Mass Spectrometer (AMS) radiocarbon dates from skeleton remains of 40 individuals, performed by the Curt-Engelhorn-Zentrum Archaeometrie in Mannheim (MAMS, n=29), Center for Isotope Research, University of Groningen (GrA and GrM, n=5), University of Aarhus (AAR, n=3), Beta Analytics (Beta, n=2), Zürich (ETH, n=3), International Chemical Analysis (ICA, n=2), Natural History Museum in Paris (Echo Lab, n=1) and Vilnium (FTMC. n=2). Conventional radiocarbon ages were calibrated using the OxCal 4.4 program and the IntCal20 calibration curve, with uncertainties reported at 95.4% confidence interval. Details of the radiocarbon dating methods are provided in Supplementary section 1.

☒ Tick this box to confirm that the raw and calibrated dates are available in the paper or in Supplementary Information.

Ethics oversight	No ethical approval or guidance was required as the research did not involve human participants.

Note that full information on the approval of the study protocol must also be provided in the manuscript.
